# The DEK oncoprotein binds to highly and ubiquitously expressed genes with a dual role in their transcriptional regulation

**DOI:** 10.1186/1476-4598-13-215

**Published:** 2014-09-12

**Authors:** Carl Sandén, Linnea Järvstråt, Andreas Lennartsson, Per Ludvik Brattås, Björn Nilsson, Urban Gullberg

**Affiliations:** Department of Hematology, Lund University, BMC B13, Klinikgatan 26, 221 84 Lund, Sweden; Center for Biosciences, Department of Biosciences and Nutrition, Karolinska Institute, Novum, 141 83 Huddinge, Sweden

**Keywords:** DEK, DEK-NUP214, ChIP-seq, shRNA, Gene expression, Euchromatin, Heterochromatin, Histone modifications, RNA polymerase II

## Abstract

**Background:**

The DEK gene is highly expressed in a wide range of cancer cells, and a recurrent translocation partner in acute myeloid leukemia. While DEK has been identified as one of the most abundant proteins in human chromatin, its function and binding properties are not fully understood.

**Methods:**

We performed ChIP-seq analysis in the myeloid cell line U937 and coupled it with epigenetic and gene expression analysis to explore the genome-wide binding pattern of DEK and its role in gene regulation.

**Results:**

We show that DEK preferentially binds to open chromatin, with a low degree of DNA methylation and scarce in the heterochromatin marker H3K9me^3^ but rich in the euchromatin marks H3K4me^2/3^, H3K27ac and H3K9ac. More specifically, DEK binding is predominantly located at the transcription start sites of highly transcribed genes and a comparative analysis with previously established transcription factor binding patterns shows a similarity with that of RNA polymerase II. Further bioinformatic analysis demonstrates that DEK mainly binds to genes that are ubiquitously expressed across tissues. The functional significance of DEK binding was demonstrated by knockdown of DEK by shRNA, resulting in both significant upregulation and downregulation of DEK-bound genes.

**Conclusions:**

We find that DEK binds to transcription start sites with a dual role in activation and repression of highly and ubiquitously expressed genes.

**Electronic supplementary material:**

The online version of this article (doi:10.1186/1476-4598-13-215) contains supplementary material, which is available to authorized users.

## Introduction

The DEK oncogene is highly expressed in many types of cancers, including breast, ovarian, bladder, colon, and skin cancer as well as acute myeloid leukemia [1–3]. High DEK expression is also associated with advanced disease and poor prognosis [4–6]. No mutations have been reported and upregulation may occur through copy gains [7] or transcriptional activation by upstream regulators such as E2F-1 [1], NF-Y [8], YY-1 [8] and ERα [9]. The DEK gene is also part of the t(6;9) chromosomal translocation resulting in the DEK-NUP214 fusion gene, which is found in 1% of acute myeloid leukemias and promotes cellular proliferation and transformation [10, 11].

What is known of the role of DEK in cancer biology is multifaceted. The expression is generally high in rapidly proliferating cells and knockdown of DEK by shRNA reduces the proliferation of cell lines from several tissues [1, 12]. Inhibition of DEK is sufficient to drive melanoma cells into senescence whereas overexpression prolongs cellular lifespan [13, 14]. In several cell types, DEK expression is reduced during cellular differentiation and depletion of DEK promotes the differentiation of both cell lines and primary cells [15, 16]. Conversely, overexpression of DEK causes a shift in keratinocytes from a differentiated to a proliferative state [16]. Many studies have also implicated DEK in apoptosis, although with differing roles depending on the cellular context. In HeLa cells, DEK depletion leads to apoptosis through p53 stabilization, whereas knockdown of DEK in melanoma cells causes downregulation of the anti-apoptotic protein MCL-1 [2, 17]. Reduced expression of DEK has also been shown to increase the sensitivity to apoptotic agents [18]. DEK is thus implicated in several essential oncogenic mechanisms, including both proliferation, differentiation and apoptosis.

Consistent with a role in these processes, DEK contributes to cellular transformation. This has been most strikingly demonstrated in keratinocytes, where cells overexpressing DEK in addition to the *HRAS*, HPV E6 and E7 oncogenes display increased potential to form colonies in soft agar and tumors when transplanted into mice. The transformed cells are more sensitive to depletion of DEK than the surrounding normal tissue, raising the possibility of oncogene addiction and DEK as a drug target. This notion is further supported by the finding that DEK knockout mice are less prone to develop tumors when challenged with carcinogens [19].

DEK is a structurally unique and highly conserved protein with emerging roles in epigenetic and transcriptional regulation. The DEK protein changes chromatin topology by introducing positive supercoils and assembles DNA and histones into chromatin [20, 21]. It has also been shown to sustain the levels of the repressive histone mark H3K9me^3^ and inhibit several activating histone acetyl transferases [22, 23]. Concordantly, DEK has been deemed essential for the preservation of transcriptionally inactive heterochromatin [22]. However, immunofluorescent imaging as well as immunoprecipitation shows accumulation of DEK in regions of transcriptionally active euchromatin [20, 24]. The reported roles of DEK in transcriptional regulation are similarly paradoxical. DEK counteracts transcriptional activation by SET, NFκB, P/CAF and p300 and is found in a repression complex with Daxx [23, 25–27]. But it is also a coactivator of U2AF and the *Drosophila* ecdysone receptor, enhances the transcriptional activity of AP-2α and C/EBPα, and accumulates during transcriptional activation of the CR2 gene [20, 28–31]. To investigate the seemingly conflicting reports on the geography of DEK binding and its role in gene regulation, we performed a genome-wide analysis of global DEK binding by ChIP-seq and knocked down DEK with shRNA to analyze changes in gene expression. We find that DEK binds to transcription start sites of highly and ubiquitously transcribed genes and that DEK binding can serve to either promote or repress transcription.

## Results

### DEK binds close to the transcription start site

**Figure 1:**
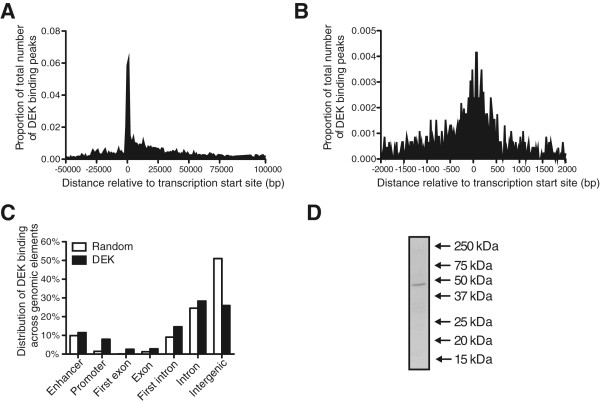
**DEK binding accumulates at transcription start sites.** Distribution of DEK binding peaks based on the distance to the transcription start sites of the bound genes, across 150000 bp **(A)** and 4000 bp **(B)**. **(C)** The types of genomic elements bound by DEK, with random genomic sequences as control. **(D)** Specificity of the ChIP-seq antibody, as demonstrated by Western blot of U937 cell lysate showing a distinct band of the expected size of the DEK protein, 43 kDa.

For a genome-wide analysis of DEK binding, we performed chromatin immunoprecipitation of DEK in the U937 cell line followed by high-throughput sequencing (ChIP-seq). We identified 4581 peaks of DEK binding, determined as overlapping peaks in two independent precipitations (Additional file 1: Table S1). The specificity of the immunoprecipitating antibody was verified by Western blot (Figure 1D). To determine the dispersion pattern of DEK throughout the genome, we calculated the distance from the middle of each DEK peak to the nearest transcription start site (TSS). The result shows a strong accumulation of DEK binding around the TSS (Figure 1A-B). This was confirmed by analysis with the Nebula software, showing that the highest enrichment of binding occurs in promoter regions (Figure 1C).

### DEK binds to highly and commonly expressed genes

**Figure 2:**
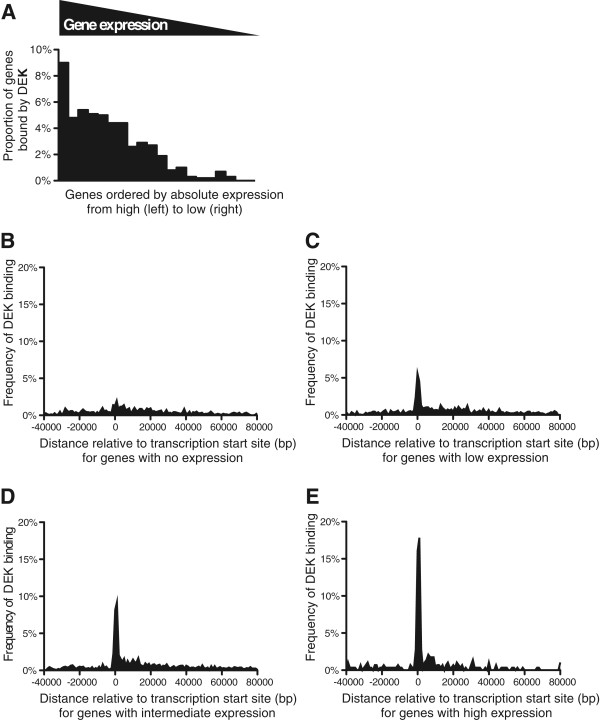
**DEK binds to highly expressed genes.** Frequency of DEK binding to genes, based on their expression. Absolute expression levels in the U937 cell line for all genes in the genome were determined by Cap Analysis of Gene Expression (CAGE). The genes were then ordered based on their expression and grouped into bins of one thousand genes, with the thousand genes with the highest expression in the leftmost column and the ones with the lowest expression in the rightmost column. For each group, we calculated the proportion of the genes that were found to be bound by DEK, showing that DEK binds most commonly to highly transcribed genes **(A)**. Distribution of DEK peaks relative to the transcription start sites of genes with no **(B)**, low **(C)**, intermediate **(D)** and high **(E)** expression.

**Figure 3:**
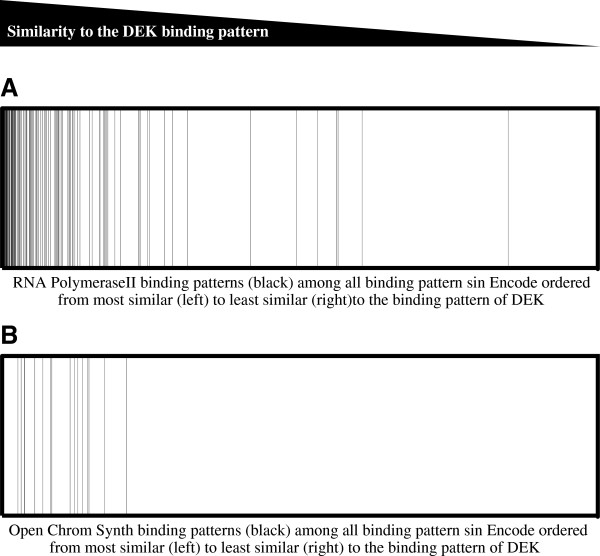
**DEK binding correlates with binding by RNA polymerase II and an open chromatin structure.** Distribution of the 2642 ChIP-seq experiments in the Encode database based on their similarity with the result of the DEK ChIP-seq. The binding pattern of every Encode experiment was compared to the binding pattern of DEK and subsequently ordered from left to right based on their similarity, with the most similar binding pattern furthest to the left and the least similar pattern furthest to the right. **(A)** Each black line represents a Chip-seq experiment with RNA polymerase II, showing a strong enrichment of these binding patterns among the ones most similar to that of DEK (p < 3×10^−6^). **(B)** Each black line represents a Chip-seq experiment marking open chromatin, by synthesis of results from ChIP-seq, FAIRE-seq and DNAse hypersensitivity analysis, showing strong enrichment among the binding patterns most similar to that of DEK (p < 4×10^−5^).

To characterize the genes bound by DEK, we stratified them based on their expression levels. All the genes in the genome were assembled into groups of one thousand, from the most to the least expressed genes based on previously established expression levels in the U937 cell line, as determined by CAGE analysis [32]. For each group, we then calculated the proportion of genes that were bound by DEK. To exclude DEK binding which is less likely to be relevant for gene regulation, we selected the genes to which DEK binds within 1 kb from the transcription start site but similar results were obtained using other or no boundaries. The result clearly demonstrates that DEK mainly binds to highly transcribed genes (Figure 2A). Furthermore, we divided all the DEK-bound genes into four groups based on their expression; no (CAGE 0), low (CAGE 0–10), intermediate (CAGE 10–100) and high (CAGE >100) expression. We then calculated the distances from each DEK binding site to the nearest transcription start site. Interestingly, the genes that were not expressed lacked the characteristic accumulation of DEK at the transcription start sites (Figure 2B). And for the expressed genes, higher expression correlated with higher accumulation of DEK (Figure 2C-E). The correlation between DEK binding and gene transcription was further substantiated by comparing the DEK binding pattern to those of previously characterized factors. We constructed an algorithm that scored and ranked the binding patterns from all 2642 ChIP-seq experiments in the Encode database based on their similarity with the DEK binding pattern (Additional file 2: Table S2). Strikingly, many of the most similar binding patterns represented ChIP-seq analyses of RNA polymerase II (POL2) binding, indicating active gene transcription. This was visualized by ranking the ChIP-seq experiments in Encode from most to least similar to DEK, with POL2 experiments represented by black bars (Figure 3A). The analysis clearly shows accumulation of POL2 experiments among the patterns most similar to that of DEK, with half of the 100 most similar binding patterns belonging to POL2. The same result was obtained when the analysis was conducted only with Encode binding patterns established in myeloid cells (Additional file 3: Table S3). In all four myeloid datasets, the POL2 binding patterns were among the very most similar to the DEK binding pattern (Additional file 4: Table S4). The Encode database contains experiments from a wide range of cells and tissues. Thus, the striking similarity between DEK and POL2 binding across the entirety of the database, as seen in Figure 3A, shows that DEK binding is associated with genes that are commonly transcribed across different cell types rather than tissue- or lineage-specific genes. To confirm this notion, we constructed figures as the one in Figure 3A for each transcription factor in the Encode database. We then calculated the similarity between the binding pattern of each factor and the complete collection of POL2 experiments, as the rank of the median POL2 experiment. Finally, we compared these scores with that of DEK. Out of the 1172 analyzed binding patterns, only 39 had a higher correlation with overall POL2 binding than DEK did (Additional file 5: Table S5). With few exceptions, the precipitated factor in these cases was POL2 itself. DEK even ranked higher than 66% of the POL2 binding patterns. This confirms that DEK binds to genes that are not only highly expressed but also ubiquitously expressed across different cell types.

### DEK binds to open chromatin

**Figure 4:**
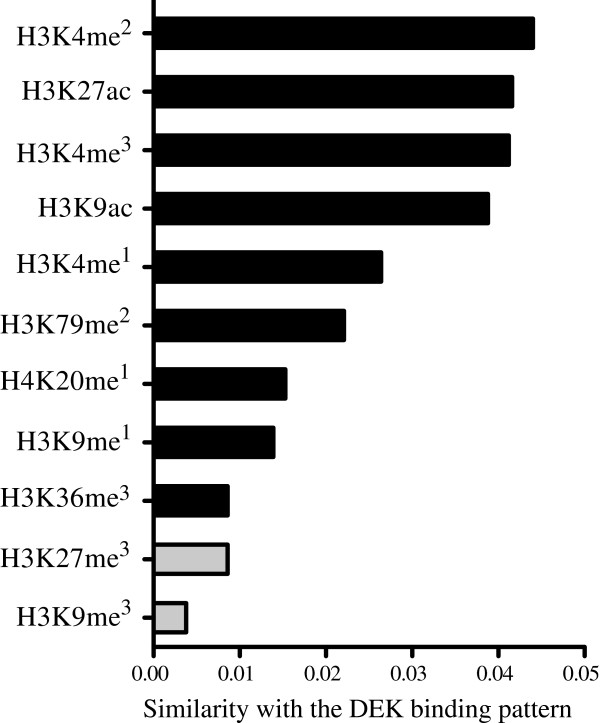
**DEK binding correlates with activating histone marks.** Similarity between the DEK binding pattern and those of ChIP-seq experiments from the Broad Histone K562 dataset in Encode, showing the highest similarity with activating histone marks (black bars) and the lowest similarity with the two repressive histone marks (grey bars).

**Figure 5:**
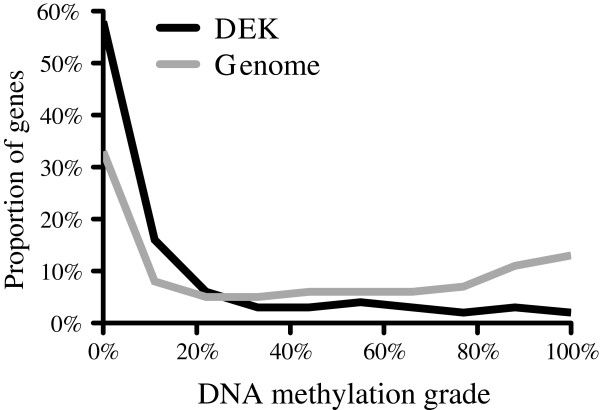
**DEK binds to lowly methylated DNA.** DNA methylation grade for the genes bound by DEK (black line) and the complete genome (grey line), showing lower methylation for the DEK-bound genes, as determined by DNA methylation array analysis of U937 cells.

Given the binding of DEK to highly expressed genes and the implication of DEK in epigenetic regulation, we wanted to characterize the epigenetic landscape of DEK binding, To this end, we compared the DEK binding pattern with the Encode experiments termed OpenChromSynth, which indicate regions of open chromatin, as determined by a combination of ChIP-seq, FAIRE-seq and DNAse hypersensitivity analysis. The result shows high similarity between these genomic regions and the DEK binding pattern (Figure 3B), suggesting extensive overlap between DEK binding and regions of open chromatin. The same result was obtained when the analysis was restricted to the myeloid datasets (data not shown). To determine the histone modifications associated with DEK binding, we calculated the similarity between the DEK binding pattern and those of the histone modifications analyzed in the BroadHistone K562 dataset in Encode (Figure 4). In concordance with binding to highly transcribed genes, DEK showed the highest degree of overlap with the active TSS marker H3K4me^3^, the active enhancer marker H3K27ac and the active promoter markers H3K4me^2^ and H3K9ac [33–36]. Conversely, the two repressive histone modifications H3K27me^3^ and H3K9me^3^ (grey bars) were the ones with the least overlap [37]. The association between DEK and active chromatin was further demonstrated by performing a DNA methylation array to calculate the methylation grade for each gene. The methylation of the genes bound by DEK was then compared to the global gene methylation. The result shows markedly lower methylation of the genes to which DEK binds (Figure 5). Thus, consistent with the binding to the transcription start sites of highly and commonly transcribed genes, we also find that DEK binds to regions of open chromatin with low DNA methylation and which are rich in activating histone marks.

### DEK binding is enriched among certain sets of genes

The results above show that DEK binds to genes commonly expressed in different cell types and tissues. This was further confirmed by gene ontology analysis of the genes to which DEK binds within 1000 bp of the transcription start site. The significantly enriched categories mainly represent basic metabolic processes that are shared by all cells (Table 1). Several are however frequently deregulated in cancer and essential for cancer cell proliferation, such as protein synthesis and cell cycle regulation. Notably, the category with the highest enrichment of DEK-bound genes is “Nucleosome assembly”, a process in which DEK itself is directly and functionally implicated [38]. This suggests that DEK could contribute to epigenetic regulation not only directly but also by binding and regulating other genes involved in the process. Gene ontology analysis of the genes deregulated by knockdown of DEK in U937 cells by shRNA shows that many of the genes whose expression is affected by DEK are involved in the same processes, such as cell cycle and chromatin regulation (Additional file 6: Table S6). Since no binding motif has been defined for the DEK protein, we analyzed the DEK-bound sequences for enriched motifs (Table 2). The identified motif with the highest degree of significance was found to be a binding motif for the transcription factor PU.1, a key regulator of gene expression during myeloid cell development and highly expressed in these cells. The other known motifs belong to the transcription factors SP1, RUNX1 and USF1, all of which are also expressed in these cells. Although we also identified several motifs not corresponding to known transcription factors, it is unlikely that any of these represent genuine DEK motifs, since none of them predict DEK binding as well as the PU.1 motif.

**Table 1:** **DEK binds to genes involved in multiple cellular functions**

Gene ontology term	Fold enrichment	P value
Nucleosome assembly	5.96	2.7 × 10^−6^
mRNA metabolic process	2.95	1.8 × 10^−8^
RNA splicing	2.82	5.3 × 10^−3^
Ubiquitin-dependent protein catabolic process	2.59	4.9 × 10^−3^
Cellular macromolecular complex assembly	2.58	5.0 × 10^−5^
Viral process	2.34	7.2 × 10^−4^
Interspecies interaction between organisms	2.20	2.8 × 10^−3^
Protein complex biogenesis	2.16	7.9 × 10^−5^
Cell cycle	1.84	4.7 × 10^−4^
Cellular catabolic process	1.70	2.5 × 10^−3^
Gene expression	1.41	5.0 × 10^−4^

Gene ontology analysis of the DEK-bound genes, showing enrichment in many categories that are central for cancer biology.

**Table 2:** **DEK binds to DNA with motifs for major transcriptional regulators**

Motif	Motif e value	Match	Match P value
AGGAARH	1.5 × 10^−41^	PU.1	0.000013
DAAATR	5.6 × 10^−22^	-	-
CMCMGCC	9.3 × 10^−20^	SP1	0.000019
ACACASR	1.3 × 10^−18^	-	-
KTTTCY	1.5 × 10^−13^	-	-
CHGCMGCC	6.7 × 10^−12^	-	-
CTTCCBS	3.0 × 10^−9^	PU.1	0.000006
CAGCKCC	1.0 × 10^−8^	-	-
TWTAWA	3.4 × 10^−8^	-	-
MTGTGGY	6.9 × 10^−6^	RUNX1	0.000017
CACGTG	4.5 × 10^−5^	USF1	0.000001
CCCCACCC	3.5 × 10^−3^	SP1	0.000044
CTGTCACY	7.6 × 10^−3^	-	-

Motif analysis of the sequences bound by DEK, identifying the most common motif not as a unique DEK motif but as that of the transcription factor PU.1. The motif E value denotes the statistical significance of the enrichment of the motif among the DEK-bound sequences. The match p value denotes the statistical significance of the match between the enriched motif and that of the matching transcription factor. Nucleotide sequences are described using standard IUPAC nomenclature, where B denotes C/G/T, D denotes A/G/T, H denotes A/C/T, K denotes G/T, M denotes A/C, R denotes A/G, S denotes C/G, W denotes A/T and Y denotes C/T.

### DEK binding correlates with gene expression

**Figure 6:**
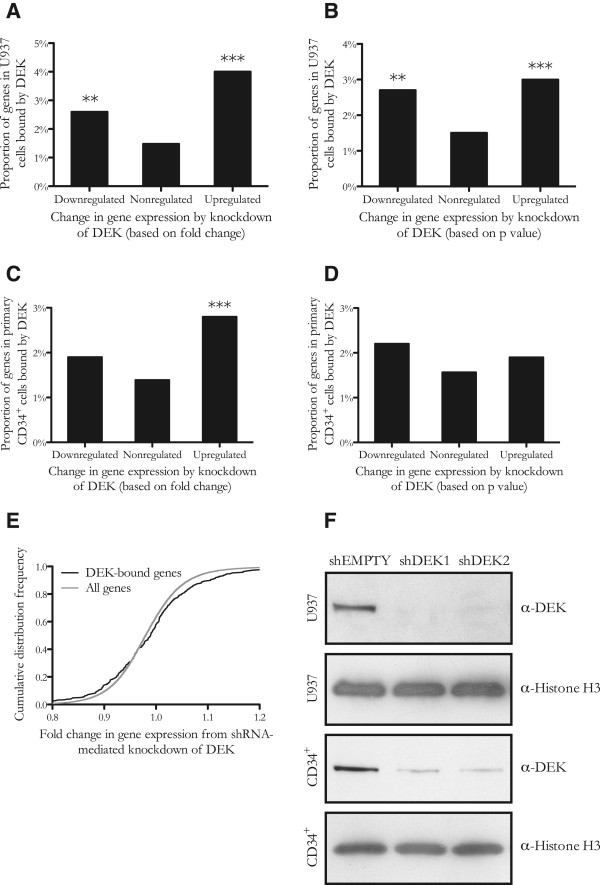
**Genes bound by DEK are both up- and downregulated by the knockdown of DEK.** Proportion of genes that are bound by DEK among the genes that were upregulated, downregulated and not affected by the knockdown of DEK. Both the downregulated and the upregulated genes are enriched for DEK-bound genes, showing that DEK binding is associated with transcriptional regulation and may contribute to both promotion and repression of gene transcription. The downregulated and upregulated categories were defined as the thousand most up- and downregulated genes based on either fold change **(A and C)** or p value **(B and D)**, as determined by gene expression microarray analysis of U937 **(A and B)** and primary CD34^+^ cord blood cells **(C and D)**. **(E)** Cumulative distribution frequency of genes based on their fold change in gene expression upon knockdown of DEK in U937 cells, showing enrichment of DEK-bound genes both among the genes most upregulated and the genes most downregulated by DEK depletion. **(F)** The efficiency of the shRNA-mediated knockdown of DEK in U937 and primary CD34^+^ cord blood cells, as determined by Western blot. Histone H3 was used as an equal loading control.

The role of DEK in gene regulation is far from understood. Previous studies have determined both activating and repressive effects on the expression of single genes. To study the effect on a genome-wide scale, we combined the results of the ChIP-seq analysis with gene expression microarray analysis following knockdown of DEK by shRNA. Based on the microarray analysis, we characterized the DEK-bound genes as either upregulated, downregulated or unaffected by the knock-down of DEK (Additional file 7: Table S7). To only include the DEK binding most likely to influence gene expression, the analysis was limited to genes where DEK binds less than 1000 bp either upstream or downstream from the transcription start site. Compared to the complete genome, significantly more genes were found to be either upregulated or downregulated by DEK depletion than unaffected. The same result was obtained when either fold change (Figure 6A, E) or statistical significance (Figure 6B) were used as the measure of change in gene expression. The same pattern was observed when DEK was knocked down in primary CD34^+^ cells isolated from human umbilical cord blood (Figure 6C-D). That the correlation is not as strong for the primary cells is expected, given the discrepancy in gene expression between these cells and the U937 cell line in which the ChIP-seq analysis was performed. These findings emphasize the role of DEK as a gene regulatory protein and indicate that DEK serves as both an activator and a repressor of transcription in the same cell under the same conditions. To validate the association of DEK binding with gene expression, we performed a network analysis of a collection of publicly available microarray datasets from patients with acute myeloid leukemia. For all of the 1246 analyzed transcription factors, we calculated the number of genes whose expression correlated with that of each transcription factor (Table 3). Interestingly, DEK was found to be the factor with the third highest number of correlated genes. The analysis was then repeated using microarray data from the Microarray Innovations in Leukemia (MILE) study [39]. In this analysis, DEK ranked in the 11^th^ percentile and out of the 30 factors with the most connections in the original dataset, DEK was the one with the most connections in the MILE dataset. Taken together, the network analyses underscore the importance of DEK in gene regulation.

**Table 3:** **DEK expression correlates with the expression of other genes**

Transcription factor	Correlations in the AML dataset	Correlations in the MILE dataset
ZNF593	1582	141
FOXP2	1437	0
DEK	1380	198
ZNF479	1235	4
NKX1-1	1135	0
TRIM28	1083	178
BARHL1	1045	0
ESR1	1042	1
USF2	1037	110
ZFN396	1020	0

Network analysis of gene expression in publicly available datasets, showing DEK as one of the transcription factors whose expression has the most correlations to the expression levels of other genes. The ten transcriptions factors with the most correlations in the AML network are shown.

## Discussion

This study provides a genome-wide map of DEK binding in myeloid cells. We show that DEK does not bind uniformly to long stretches of the genome, as previously suggested by the high amounts of DEK bound to chromatin [21]. Instead, we demonstrate that DEK binding is highly distinct and centered around transcription start sites. We continue to show that DEK mainly binds to highly expressed genes and that the accumulation of binding around the transcription start site positively correlates with the transcription of the gene. These findings are in concert with a previous study of a single gene locus, which found that DEK binding to the promoter of the complement receptor 2 gene is 2–3 fold higher in a cell line expressing the gene than in a cell line without expression and in which DEK was shown to be recruited to the promoter upon induction of gene expression [29]. Furthermore, our findings are consistent with previous reports that DEK contributes to positive supercoiling of the DNA structure, which opens up the chromatin to allow access to the transcriptional machinery and is a characteristic of highly transcribed genes [21, 40]. The correlation between DEK binding and gene expression is further underlined by our finding that out of the 2642 binding patterns in the Encode database, the DEK binding pattern is most similar to that of RNA polymerase II.

Interestingly, we found that the DEK binding pattern not only resembles that of RNA polymerase II in hematopoietic cells but also in highly different cell types. In a comparison with the complete collection of POL2 bindings patterns in Encode, DEK was one of the factors with the highest degree of similarity. The DEK binding pattern actually scored higher than most POL2 binding patterns in terms of similarity with overall POL2 binding. This shows that DEK binds to genes that are commonly expressed across cell types, which could explain the ubiquitous expression of DEK in human tissues. Genes that are expressed in very different cell types generally contribute to common functions such as cellular organization and metabolism. Gene ontology analysis confirmed that genes bound by DEK are involved in basic cellular functions such as catabolism, biogenesis and chromatin organization. Many of these processes are not only fundamental to normal cells but must also be deregulated in order for cancer cells to produce the macromolecules and the energy needed for their high proliferation. Accelerating basic cellular functions could thus be a way by which DEK contributes to carcinogenesis. This notion would be compatible with the previous observation that DEK is essential for tumor cells but dispensable for their normal counterparts [19]. We also show that DEK binds to genes involved in cell cycle regulation and gene expression, processes with obvious implications for cancer biology. Since the binding patterns collected in the Encode database are mainly derived from transformed cell lines, some of the commonly expressed genes encode the proteins conferring the cancer phenotype. We show that the genes bound by DEK are also enriched for genes involved in cell cycle regulation and gene expression, which could mediate the oncogenic function of DEK.

Previous studies have provided contradictory indications regarding the association of DEK with euchromatin and heterochromatin. Based on immunofluorescence imaging, DEK co-localizes with regions of open chromatin containing acetylated histone H4 [24]. It also co-precipitates with the activating histone marks H3K4me^2^ and H3K4me^3^ [20]. Contrarily, other reports have indicated DEK as essential for the maintenance of heterochromatin by strengthening the binding between heterochromatin protein 1α and the heterochromatin marker H3K9me^3^ [22]. Here, we show that DEK binding overlaps with histone marks found in euchromatin and with genes carrying a low degree of DNA methylation. We thus conclude that DEK preferentially binds to euchromatin and more specifically to transcription start sites of euchromatic genes.

DEK has been shown to bind to chromatin in a manner dependent on the structure rather than the sequence of the DNA, based on the finding that DEK accumulates at sites of supercoiled and four-way junction DNA [41]. However, sequence-specific binding to the peri-*ets* site of the HIV-2 enhancer has been demonstrated [42]. To determine the sequence-specificity of the genome-wide DEK binding, we performed motif analysis of the bound sequences. The analysis identified the most significantly enriched motif as that of the hematopoietic transcription factor PU.1. However, given that PU.1 is a major transcription factor in these cells, many of its target genes coincide with the highly expressed genes bound by RNA polymerase II. Since the comparison with the Encode experiments shows that the binding pattern of DEK is more similar to the binding pattern of RNA polymerase II than to that of PU.1, it is more likely that the binding to genes with PU.1 motifs is a consequence of their high expression than that PU.1 would be the major determinant of DEK binding. We also identified several previously uncharacterized motifs. However, it is unlikely that any of these is a common DEK motif as none of them were nearly as significantly enriched as the PU.1 motif. Thus, we find that DNA sequence does not predict DEK binding as well as gene expression.

The role of DEK binding in gene expression is still ambiguous, with reports of contributions to either activation or repression of single genes under different conditions. Our finding that knockdown of DEK leads to both upregulation and downregulation of DEK-bound genes suggests that DEK has a dual role in gene regulation in that it can either promote or repress transcription of different genes in the same cellular context. The determinants of the effect of DEK on transcription are still unknown but could potentially include phosphorylation by casein kinase 2, which has been shown to alter but not abolish the association between DEK and chromatin [43]. Another possible model is one where DEK contributes to either activation or repression depending on the cofactors that bind at the gene regulatory site.

To further examine the importance of DEK in gene regulation in primary leukemic cells, we constructed a network model of gene expression and found that out of the 1246 analyzed factors, DEK was the factor with the third highest number of correlated genes. This suggests that DEK may have a broad set of targets, consistent with our findings that DEK binds to many highly and commonly expressed genes. Furthermore, it strongly suggests that DEK is important for gene regulation and may play a major role in the gene regulatory pathways that govern cancer cells. Characterizing the precise mechanistics of DEK-mediated gene regulation will be an important challenge for future research and a key to understanding the role of DEK in cancer biology and its potential as a therapeutic target.

## Materials and methods

### Cell culture

The U937 cell line (ATCC, Manassas, VA, USA) was cultured in RPMI 1640 medium (Life Technologies, Carlsbad, CA, USA) supplemented with 10% fetal bovine serum (Life Technologies). Primary CD34^+^ cells were obtained from human umbilical cord blood collected at Skåne University Hospital. The mononuclear cell population was isolated by separation on Lymphoprep (Axis-Shield PoC AS, Oslo, Norway) and CD34^+^ cells were subsequently selected using the Indirect CD34 MicroBead Kit (Miltenyi Biotec, Bergisch Gladbach, Germany). The cells were grown in StemSpan SFEM medium (Stemcell Technologies, Vancouver, Canada) supplemented by 20% fetal bovine serum and the CC100 cytokine cocktail (Stemcell Technologies).

### ChIP-seq

Chromatin immunoprecipitation was performed on U937 cells using the Magna-ChIP A/G Chromatin Immunoprecipitation Kit (Merck Millipore, Billerica, MA, USA) after crosslinking with 1% formaldehyde for 15 min. Chromatin shearing was achieved by sonication in a Bioruptor UCD-200 (Diagenode, Liège, Belgium). Immunoprecipitation was performed with 10 μg of DEK antibody (Abcam, Cambridge, UK; product code ab74975) per million cells. Immunoprecipitated DNA was sequenced by the Science for Life Laboratory in Stockholm, Sweden with an Illumina HiSeq 2000 as paired-end reads to 100 bp with a minimum of 18 million reads per sample. The Illumina OLB v1.9 was used for base conversion, the Bowtie 2 software [44] was used for the alignment of reads to the hg19 reference genome and peak calling was performed with the MACS software v1.4.1 [45]. Default parameters were used throughout the process. Two independent ChIP-seq experiments were performed under identical conditions and the peaks found in both experiments were used for all subsequent analyses. Non-precipitated chromatin was used as negative control. Validation was performed by real-time PCR analysis of DNA immunoprecipitated with the DEK antibody, showing enrichment of DNA corresponding to the predicted binding sites in the *S100A9* (fold enrichment 2.4) and *VIM* (fold enrichment 1.8) genes and a lack of enrichment of DNA corresponding to the *IRF8* gene (fold enrichment -3.1), which was determined by the ChIP-seq analysis to not be bound by DEK. The raw data from the ChIP-seq analysis is available through the Gene Expression Omnibus data repository, with the accession number “GSE60692”.

### Binding to genomic elements

The “Genomic annotation of ChIP-seq peaks” tool in the Nebula software package [46] was used with default parameters to find the closest gene to each DEK peak and calculate the distance from the transcription start site to the middle of the peak. The same tool was used to determine the genomic elements to which DEK binds, using default settings where promoters are designated as the 2.000 bp upstream of the transcription start site and enhancers are designated as the 30.000 bp upstream of the TSS. As control, random genomic sequences were generated by a random draw with replacement from a square-distribution across all genomic positions.

### Cap analysis of gene expression

Absolute gene expression levels for all genes in the U937 cell line were determined by Cap Analysis of Gene Expression (CAGE) in a study by the FANTOM5 consortium [32]. All genes were ordered by absolute expression and divided into bins of one thousand, for which we calculated the number of genes where DEK binds within 1000 bp from the transcription start site. For further analysis, all DEK-bound genes were divided into four categories according to their expression; no (0), low (0–10), intermediate (10–100) or high (>100) expression. For each category, the distances to the nearest transcription start sites were calculated as described above.

### Correlation with existing ChIP-seq binding patterns

The determined DEK binding pattern was compared to the binding patterns previously established for transcription factors and histone modifications as well as the genome-wide distributions of characteristics such as DNAse hypersensitivity. To this end, we constructed an algorithm which analyzed all of the 2642 such patterns in the Encode database and scored them based on their similarity with the binding pattern of the DEK protein. For each Encode binding pattern, the weight of each base was calculated based on the number of sequence tags at that position in the genome. The raw correlation between this sequence tag distribution and that of DEK was then calculated as the sum of the products of the weights of each matching base, corrected for track length using the inverse square of the total weight of each track. Formally,


where weight1_i_ and weight2_i_ are the score assigned basepair i in the two tracks compared, respectively, and n_basepairs_ is the total number of basepairs in the hg19 assembly of the human genome. DEK binding was correlated with open chromatin status by comparison of the DEK binding pattern with the Encode tracks designated OpenChromSynth, each containing a synthesis of results from ChIP-seq, FAIRE-seq and DNAse hypersensitivity analysis in the examined cell type. The binding patterns of different histone marks in the K562 cell line were obtained from the Broad Histone dataset in Encode. Statistical testing of the enrichment of tracks representing either RNA polymerase II or OpenChromSynth among the most similar binding patterns to that of DEK was performed using the RenderCat software, as previously described [47].

### DNA methylation array

Microarray-based DNA methylation analysis was performed with the Infinium HumanMethylation27 BeadChip (Illumina) by the BEA core facility at Karolinska Institute. Genomic DNA from U937 cells was extracted using the QIAamp DNA mini kit (Qiagen) and bisulfite converted using the Zymo EZ DNA methylation Kit (Zymo Research, Irvine, CA). Subsequently, the DNA was subjected to the Illumina Infinium HD Methylation assay including whole genome amplification and enzymatic fragmentation before hybridization to the BeadChip. Arrays were scanned and the signals processed in Genome Studio module 1.8. The methylation grade for each gene was calculated as the average of the corresponding probes. The methylation of the genes to which DEK binds within 1000 bp of the transcription start site was then compared to the methylation of the entire genome. The raw data from the DNA methylation analysis is available through the Gene Expression Omnibus data repository, with the accession number “GSE60734”.

### Knockdown of DEK by shRNA

Two shRNAs in H1 lentiviral vectors targeting the DEK transcript were a kind gift from Dr David Markovitz [2]. Lentiviral particles were harvested after calcium phosphate transfection of 293 T cells (ATCC) with the respective shRNA constructs, *gag-pol* and the RD114 envelope gene. For lentiviral transduction, non-tissue culture coated plates were coated with retronectin (Takara, Otsu, Japan) and blocked with 2% bovine serum albumin (Sigma-Aldrich, St. Louis, MO, USA) for 30 min at room temperature. Subsequently, virus-containing medium was added and the plates were centrifuged at 1000 × g for 60 min at 4°C before the cells were added and incubated at 37°C for 48 h, after which they were sorted by FACS based on the expression of the GFP marker. Transduction efficiencies were similar for all constructs and consistently above 40%. The efficiency of the knockdown was verified by Western blot of cell lysates obtained 4 days (U937 cells) or 10 days (primary cells) after sorting, with a primary DEK antibody (BD Transduction Laboratories, San Jose, CA, USA). The raw data from the gene expression analysis is available through the Gene Expression Omnibus data repository, with the accession number “GSE60734”.

### Gene expression microarray

RNA was isolated with the RNeasy Mini Kit (Qiagen, Hilden, Germany) from cells harvested 4 days (U937) or 10 days (primary cells) after sorting. Microarray analysis was performed in triplicates with the HumanHT-12 v4 Expression BeadChip (Illumina, San Diego, CA, USA) by the Swegene Centre for Integrative Biology at Lund University (SCIBLU). Designated as upregulated or downregulated were the genes found to be among the thousand most strongly upregulated or downregulated based on either the average fold change or the combined p value from statistical testing of the two shRNA constructs by the student’s t test. Subsequently, the number of genes to which DEK binds within 1000 bp from the transcription start site was calculated for each category. The gene expression microarray was validated by quantitative real-time PCR analysis of two genes found to be upregulated (CSF2RA; fold change 1.3, p = 0.004 and CSF3R; fold change 1.3, p = 0.04) and two genes found to be downregulated (CHMP2B; fold change −1.3, p = 0.03 and ICAM2; fold change −1.4, p = 0.002) upon knockdown of DEK in the U937 cell line. For this analysis, RNA was reverse-transcribed to cDNA with the High Capacity cDNA Reverse Transcription Kit (Life Technologies) and real-time PCR was performed using the TaqMan Gene Expression Assay (Life Technologies) and the StepOne Plus Real-Time PCR System (Life Technologies).

### Gene ontology analysis

Gene ontology analysis of the genes bound by DEK was performed with the GO::TermFinder software [48], using gene ontology associations based on the UniProt reference proteome. The analysis was performed on the genes to which DEK binds within 1000 bp from the transcription start site, with the default p value threshold of 0.01. Fold enrichment was calculated as the percentage of the DEK-bound genes associated with a certain term divided by the percentage of the total genome associated with the same term. The result was filtered for redundant terms and terms containing more than 5000 genes. Gene ontology analysis of the genes deregulated by the knockdown of DEK was performed by Gene Set Enrichment Analysis (GSEA), using a p value threshold of 0.01 and a false discovery rate threshold of 0.05 [49].

### Motif analysis

Motif analysis was performed with the Discriminative Regular Expression Motif Elicitation (DREME) software [50], using as input the sequences from 150 bp upstream to 150 bp downstream of the middle of all DEK peaks. The p value threshold was set to 0.01. Identified motifs were subsequently matched with known motifs using the TOMTOM software [51] with a false discovery rate threshold of 5%.

### Network analysis

The genome-wide AML network of gene expression was constructed by LASSO regression modeling of gene expression correlations in 3013 samples obtained by merging samples from studies based on the Affymetrix HG-U133 Plus 2.0 GeneChips (GPL570) platform (see Additional file 8 Methods for a complete list of datasets). The MILE network was constructed in the same manner but instead based on the results from stage one of the Microarray Innovations in Leukemia study [39]. To reduce the false discovery rate, the correlation threshold of the network models was set to a level at which they did not find any correlations in randomized data. The list of transcription factors used in the analysis was manually curated after its original development as part of the Differentiation Map project [52].

## Electronic supplementary material

Additional file 1: Table S1: DEK Binding Sites. Complete list of significant peaks of DEK binding, as determined by ChIP-seq analysis in the U937 cell line. (DOCX 391 KB)

Additional file 2: Table S2: Correlation between the binding pattern of DEK and those of other factors. Complete list of all 2642 ChIP-seq experiments in the Encode database, ordered from that with the most to that with the least similar binding pattern to that of DEK. (DOCX 152 KB)

Additional file 3: Table S3: Correlation between the binding pattern of DEK and those of other factors in myeloid cells. Complete list of the 201 ChIP-seq experiments in the Encode database that were performed in myeloid cells, ordered from that with the most to that with the least similar binding pattern to that of DEK. (DOCX 23 KB)

Additional file 4: Table S4: DEK binding correlates with RNA polymerase II binding in hematopoietic datasets. All ChIP-seq experiments in the four hematopoietic datasets in the Encode database were scored and ranked based on their similarity with the DEK binding pattern. In all four datasets, the binding patterns of RNA polymerase II were among the most similar to that of DEK. (DOCX 14 KB)

Additional file 5: Table S5: DEK binds to commonly expressed genes. Complete list of the 1172 analyzed transcription factors, based on their binding to genes that are commonly expressed across different tissues. For each transcription factor, the overlap with each of the other binding patterns in the Encode database was calculated. The resulting ranks of the POL2 binding patterns were extracted and the median rank was calculated, representing the similarity between the binding pattern of the transcription factor and that of POL2 across all the different cell types represented in Encode. DEK was found to be one of the transcription factors with the highest similarity, demonstrating that DEK binds to genes that are commonly expressed across different cell types. (DOCX 61 KB)

Additional file 6: Table S6: DEK affects the expression of genes involved in multiple cellular functions. Gene set enrichment analysis of the changes in gene expression following knockdown of DEK, showing that the genes deregulated upon DEK knockdown are involved in multiple cellular processes that are also enriched for genes shown in Table 1 to be bound by DEK. Positive enrichment scores correspond to enrichment among the genes downregulated by DEK knockdown and negative enrichment scores correspond to enrichment among the genes upregulated by DEK knockdown. FDR denotes the false discovery rate. (DOCX 15 KB)

Additional file 7: Table S7: DEK affects gene expression. Complete list of changes in gene expression upon knockdown of DEK by shRNA in the U937 cell line, as determined by microarray analysis. (XLS 2 MB)

Additional file 8: **Datasets used for the network analysis.** (DOCX 34 KB)
