# Supplementary material for: The DEK oncoprotein binds to highly and ubiquitously expressed genes with a dual role in their transcriptional regulation
Source: Mol Cancer. 2014 Sep 12;13:215. doi: 10.1186/1476-4598-13-215 (PMC4175287; doi:10.1186/1476-4598-13-215)
Supplement: Supplementary file 1 — Additional file 1: Table S1: DEK Binding Sites. Complete list of significant peaks of DEK binding, as determined by ChIP-seq analysis in the U937 cell line. (DOCX 391 KB) [file 12943_2014_1416_MOESM1_ESM.docx]

**Additional file 1: Table S1.** **DEK Binding Sites.** Complete list of significant peaks of DEK binding, as determined by ChIP-seq analysis in the U937 cell line.

| CHROMOSOME | START POSITION | END POSITION | GENE NAME | SCORE |
| --- | --- | --- | --- | --- |

|  |  |  |  |
| --- | --- | --- | --- |
|  |  |  |  |

| 22 | 30194798 | 30195556 | ASCC2 | 2031 |
| --- | --- | --- | --- | --- |
| 8 | 17738928 | 17739395 | FGL1 | 1874 |
| 1 | 149821787 | 149825604 | HIST2H3C | 1739 |
| 8 | 3563258 | 3564107 | CSMD1 | 1575 |
| 1 | 149856911 | 149859720 | HIST2H2AC | 1569 |
| 13 | 31233835 | 31234751 | USPL1 | 1461 |
| 1 | 149811337 | 149815072 | HIST2H3C | 1450 |
| 7 | 158123399 | 158124347 | PTPRN2 | 1359 |
| 1 | 33237166 | 33240203 | KIAA1522 | 1337 |
| 2 | 8821931 | 8823212 | ID2 | 1206 |
| 12 | 4351671 | 4353538 | CCND2 | 1192 |
| 3 | 10593533 | 10594386 | ATP2B2 | 1182 |
| 1 | 31229126 | 31230827 | LAPTM5 | 1153 |
| 8 | 8653828 | 8655298 | MFHAS1 | 1128 |
| 8 | 6124910 | 6125609 | MCPH1 | 1123 |
| 14 | 36092754 | 36093395 | RALGAPA1 | 1112 |
| 2 | 8665511 | 8666452 | ID2 | 1106 |
| 6 | 42906658 | 42907504 | GNMT | 1098 |
| 15 | 23196967 | 23197377 | NIPA1 | 1087 |
| 18 | 9541641 | 9542569 | RALBP1 | 1075 |
| 21 | 45145788 | 45146457 | PDXK | 1018 |
| 4 | 11552604 | 11553254 | HS3ST1 | 1001 |
| 9 | 71180545 | 71181048 | C9orf71 | 988 |
| 10 | 26726710 | 26728272 | APBB1IP | 954 |
| 4 | 1403972 | 1405218 | CRIPAK | 951 |
| X | 12992473 | 12994544 | TMSB4X | 950 |
| 20 | 2765046 | 2765902 | CPXM1 | 950 |
| 1 | 21660670 | 21661611 | ECE1 | 944 |
| 10 | 25296176 | 25296715 | ENKUR | 938 |
| 13 | 110052987 | 110053220 | IRS2 | 930 |
| 14 | 67785237 | 67785908 | MPP5 | 927 |
| 17 | 42187832 | 42189010 | HDAC5 | 904 |
| 2 | 26677966 | 26679391 | CCDC164 | 899 |
| 5 | 61739695 | 61740385 | IPO11 | 897 |
| 12 | 12007671 | 12008228 | ETV6 | 896 |
| 5 | 36547200 | 36547768 | SLC1A3 | 878 |
| 11 | 22506449 | 22506887 | FANCF | 870 |
| 1 | 208096807 | 208097323 | CD34 | 870 |
| 5 | 76812390 | 76812959 | WDR41 | 863 |
| 16 | 87498288 | 87500113 | ZCCHC14 | 860 |
| 20 | 5986096 | 5986872 | CRLS1 | 859 |
| 14 | 23024680 | 23025787 | DAD1 | 823 |
| 15 | 40816059 | 40816872 | C15orf57 | 816 |
| 2 | 48101434 | 48101466 | FBXO11 | 795 |
| 8 | 3572692 | 3573510 | CSMD1 | 791 |
| 3 | 11594994 | 11595963 | VGLL4 | 784 |
| 3 | 11594994 | 11595963 | ATG7 | 784 |
| 16 | 87497441 | 87497742 | ZCCHC14 | 778 |
| 5 | 64894201 | 64894630 | TRIM23 | 777 |
| 19 | 1605504 | 1606876 | UQCR11 | 772 |
| 8 | 4488424 | 4488961 | CSMD1 | 765 |
| 16 | 87501070 | 87501740 | ZCCHC14 | 749 |
| 20 | 2653668 | 2654131 | EBF4 | 748 |
| 18 | 20839520 | 20840938 | CABLES1 | 747 |
| 8 | 3485437 | 3486444 | CSMD1 | 740 |
| 4 | 38358723 | 38359192 | KLF3 | 739 |
| 12 | 42300482 | 42301568 | GXYLT1 | 720 |
| 17 | 437145 | 437488 | VPS53 | 679 |
| 12 | 1900436 | 1901286 | CACNA2D4 | 674 |
| 12 | 1900436 | 1901286 | LRTM2 | 674 |
| 20 | 484197 | 485096 | CSNK2A1 | 672 |
| 8 | 144536424 | 144536942 | ZC3H3 | 671 |
| 3 | 196149734 | 196150599 | UBXN7 | 660 |
| 3 | 197794620 | 197795418 | FAM157A | 659 |
| 19 | 1382914 | 1384552 | NDUFS7 | 657 |
| 2 | 8929178 | 8929822 | KIDINS220 | 656 |
| 7 | 30325270 | 30325817 | ZNRF2 | 654 |
| 7 | 851874 | 855385 | SUN1 | 647 |
| 6 | 42989617 | 42991701 | RRP36 | 647 |
| 21 | 34407159 | 34407319 | OLIG2 | 646 |
| 6 | 26021716 | 26022325 | HIST1H4A | 629 |
| 17 | 77801357 | 77801574 | CBX4 | 629 |
| 7 | 42000313 | 42001073 | GLI3 | 626 |
| X | 49229858 | 49230570 | GAGE8 | 618 |
| X | 49229858 | 49230570 | GAGE2C | 618 |
| X | 49229858 | 49230570 | GAGE2A | 618 |
| X | 49229858 | 49230570 | GAGE12F | 618 |
| X | 49229858 | 49230570 | GAGE2D | 618 |
| 4 | 40054890 | 40056463 | N4BP2 | 612 |
| 7 | 113859662 | 113860267 | FOXP2 | 589 |
| 14 | 50051529 | 50053619 | RPS29 | 587 |
| 7 | 149473282 | 149474288 | SSPO | 586 |
| 7 | 30066146 | 30066745 | PLEKHA8 | 584 |
| 10 | 69644705 | 69645472 | SIRT1 | 583 |
| 2 | 3381092 | 3382423 | TTC15 | 581 |
| 3 | 15805391 | 15805863 | ANKRD28 | 578 |
| 21 | 34633819 | 34634782 | IFNAR2 | 574 |
| 4 | 40044519 | 40045941 | N4BP2 | 571 |
| 14 | 20997037 | 20997571 | RNASE10 | 566 |
| 20 | 640622 | 641477 | SRXN1 | 566 |
| 21 | 20163982 | 20164565 | TMPRSS15 | 559 |
| 5 | 11344187 | 11344555 | CTNND2 | 558 |
| 17 | 65865603 | 65866231 | BPTF | 557 |
| 3 | 9821585 | 9822508 | ARPC4 | 555 |
| 3 | 9821585 | 9822508 | TADA3 | 555 |
| 3 | 9821585 | 9822508 | ARPC4-TTLL3 | 555 |
| 17 | 34362821 | 34363551 | CCL18 | 554 |
| 8 | 22571288 | 22571932 | PEBP4 | 544 |
| 17 | 71006977 | 71008100 | SLC39A11 | 541 |
| 10 | 34808338 | 34808746 | PARD3 | 541 |
| 19 | 36399587 | 36401125 | TYROBP | 533 |
| 12 | 124990513 | 124991779 | NCOR2 | 532 |
| X | 49186006 | 49187120 | GAGE2E | 529 |
| X | 49186006 | 49187120 | GAGE13 | 529 |
| 8 | 3572528 | 3572603 | CSMD1 | 525 |
| 19 | 12905235 | 12905562 | HOOK2 | 522 |
| 5 | 149059635 | 149060967 | PPARGC1B | 521 |
| 12 | 2403483 | 2404385 | CACNA1C | 521 |
| 3 | 42801707 | 42802318 | CCDC13 | 520 |
| 12 | 68758471 | 68759227 | MDM1 | 518 |
| 22 | 29189260 | 29190032 | XBP1 | 516 |
| X | 24072103 | 24073492 | EIF2S3 | 516 |
| 5 | 14942847 | 14943506 | ANKH | 514 |
| 8 | 7906111 | 7906781 | LOC100132396 | 513 |
| 6 | 34572538 | 34573470 | C6orf106 | 513 |
| 7 | 27909473 | 27910388 | JAZF1 | 513 |
| 18 | 9614922 | 9615439 | PPP4R1 | 513 |
| 10 | 6192611 | 6195303 | PFKFB3 | 513 |
| 1 | 235098860 | 235099268 | TOMM20 | 508 |
| 18 | 11818773 | 11819182 | GNAL | 508 |
| 2 | 10683365 | 10684186 | ODC1 | 503 |
| 19 | 12900634 | 12901416 | JUNB | 501 |
| 8 | 41908290 | 41908869 | KAT6A | 500 |
| 19 | 12901956 | 12905109 | JUNB | 497 |
| 13 | 110306807 | 110307483 | IRS2 | 496 |
| 15 | 59973265 | 59974009 | BNIP2 | 495 |
| 3 | 193905263 | 193906595 | HES1 | 492 |
| 15 | 20557018 | 20558454 | GOLGA6L6 | 487 |
| 15 | 30691116 | 30691706 | CHRFAM7A | 486 |
| 12 | 2462955 | 2463668 | CACNA1C | 486 |
| 17 | 4837219 | 4837925 | GP1BA | 484 |
| 16 | 8943999 | 8944943 | CARHSP1 | 483 |
| 4 | 32514383 | 32514976 | PCDH7 | 482 |
| 4 | 39621398 | 39622221 | C4orf34 | 478 |
| 2 | 43358123 | 43358894 | ZFP36L2 | 478 |
| 3 | 111392967 | 111394190 | PLCXD2 | 477 |
| 9 | 115479920 | 115480596 | C9orf80 | 476 |
| 1 | 42266873 | 42267659 | HIVEP3 | 476 |
| 4 | 6097458 | 6097989 | JAKMIP1 | 473 |
| 18 | 73503087 | 73503586 | C18orf62 | 473 |
| 9 | 106855766 | 106856407 | SMC2 | 472 |
| 1 | 9135585 | 9136335 | SLC2A5 | 472 |
| 9 | 95881790 | 95882629 | NINJ1 | 472 |
| 6 | 5997505 | 5998250 | NRN1 | 470 |
| 1 | 42268448 | 42269210 | HIVEP3 | 470 |
| 6 | 36410373 | 36411378 | KCTD20 | 469 |
| 6 | 36410373 | 36411378 | PXT1 | 469 |
| 5 | 72860729 | 72861819 | UTP15 | 468 |
| 6 | 160239859 | 160243918 | PNLDC1 | 467 |
| 9 | 97411629 | 97412554 | FBP1 | 465 |
| 11 | 96007065 | 96007615 | MAML2 | 464 |
| 9 | 74908965 | 74909576 | ZFAND5 | 464 |
| 2 | 239431795 | 239432129 | ASB1 | 463 |
| 3 | 37102402 | 37103160 | LRRFIP2 | 463 |
| 2 | 8683581 | 8686407 | ID2 | 460 |
| 1 | 39975839 | 39975946 | BMP8A | 458 |
| 10 | 28572333 | 28572725 | MPP7 | 457 |
| 4 | 35693829 | 35694308 | ARAP2 | 456 |
| 1 | 160807194 | 160808000 | CD244 | 454 |
| 2 | 218264276 | 218264341 | TNP1 | 453 |
| 3 | 183798571 | 183798713 | HTR3E | 453 |
| 7 | 105524805 | 105525363 | ATXN7L1 | 452 |
| 1 | 38454525 | 38456812 | SF3A3 | 452 |
| 20 | 43275680 | 43276134 | ADA | 452 |
| 10 | 1530921 | 1531844 | ADARB2 | 452 |
| 1 | 42204579 | 42205320 | HIVEP3 | 451 |
| 1 | 39974682 | 39975725 | BMP8A | 450 |
| 2 | 64463520 | 64463746 | PELI1 | 449 |
| 11 | 106570273 | 106570948 | GUCY1A2 | 448 |
| 14 | 59828019 | 59828586 | DAAM1 | 448 |
| 12 | 1920317 | 1921159 | CACNA2D4 | 444 |
| 12 | 1920317 | 1921159 | LRTM2 | 444 |
| 17 | 43373318 | 43374073 | MAP3K14 | 444 |
| 16 | 15107609 | 15108198 | PDXDC1 | 444 |
| 8 | 37593788 | 37594862 | ERLIN2 | 441 |
| 11 | 93146301 | 93146784 | CCDC67 | 439 |
| 3 | 19997087 | 19997793 | RAB5A | 439 |
| 18 | 77733349 | 77733514 | TXNL4A | 439 |
| 17 | 8982780 | 8983633 | NTN1 | 437 |
| 1 | 149783059 | 149783896 | HIST2H2BF | 436 |
| 1 | 9486030 | 9487331 | SLC25A33 | 436 |
| 9 | 21172721 | 21173085 | IFNA21 | 435 |
| 3 | 123476075 | 123476462 | MYLK | 434 |
| 8 | 580779 | 581341 | C8orf42 | 433 |
| 7 | 6064943 | 6066399 | EIF2AK1 | 433 |
| 7 | 6064943 | 6066399 | AIMP2 | 433 |
| 8 | 9687318 | 9687695 | MSRA | 433 |
| 10 | 69644039 | 69644635 | SIRT1 | 433 |
| 6 | 167001474 | 167002530 | RPS6KA2 | 432 |
| 8 | 145132848 | 145134156 | EXOSC4 | 432 |
| 16 | 85709933 | 85710725 | GINS2 | 432 |
| 22 | 46683984 | 46684843 | TTC38 | 432 |
| 4 | 32319489 | 32320029 | PCDH7 | 431 |
| 19 | 39321890 | 39322816 | ECH1 | 431 |
| 1 | 244998695 | 244999368 | FAM36A | 430 |
| 2 | 58425723 | 58426306 | FANCL | 428 |
| 11 | 104603512 | 104603977 | CASP12 | 428 |
| 3 | 183797197 | 183798053 | HTR3E | 427 |
| 1 | 1710039 | 1712540 | NADK | 427 |
| 9 | 6919308 | 6920108 | KDM4C | 427 |
| 12 | 6444326 | 6446760 | TNFRSF1A | 423 |
| 15 | 28729966 | 28730463 | HERC2 | 423 |
| 14 | 75372029 | 75372817 | RPS6KL1 | 422 |
| 10 | 125795211 | 125796092 | CHST15 | 421 |
| 6 | 5108482 | 5108974 | LYRM4 | 421 |
| 2 | 63025685 | 63026248 | EHBP1 | 419 |
| 16 | 89601059 | 89601188 | SPG7 | 418 |
| 1 | 24517012 | 24517484 | IL28RA | 417 |
| 17 | 1480202 | 1481520 | SLC43A2 | 416 |
| 8 | 29630836 | 29631528 | TMEM66 | 416 |
| 3 | 125033375 | 125033977 | ZNF148 | 416 |
| 18 | 9349694 | 9350301 | TWSG1 | 416 |
| 7 | 5276025 | 5276141 | WIPI2 | 415 |
| 10 | 104576214 | 104576971 | C10orf26 | 412 |
| 7 | 11952561 | 11953149 | THSD7A | 412 |
| 12 | 4397509 | 4398087 | CCND2 | 411 |
| 4 | 41282337 | 41282978 | UCHL1 | 410 |
| 1 | 40240792 | 40242453 | BMP8B | 409 |
| 1 | 40240792 | 40242453 | OXCT2 | 409 |
| 17 | 66254852 | 66255677 | ARSG | 409 |
| 12 | 25444512 | 25445113 | KRAS | 408 |
| 1 | 2221089 | 2222880 | SKI | 408 |
| 2 | 242294308 | 242295079 | FARP2 | 408 |
| 2 | 70350518 | 70352769 | PCBP1 | 408 |
| 19 | 57149188 | 57149858 | LOC147670 | 407 |
| X | 10134447 | 10134733 | CLCN4 | 406 |
| 5 | 143568868 | 143569358 | KCTD16 | 406 |
| 1 | 26866895 | 26870134 | RPS6KA1 | 405 |
| 2 | 218263335 | 218263965 | TNP1 | 405 |
| 6 | 27106787 | 27107751 | HIST1H2BK | 403 |
| 6 | 27106787 | 27107751 | HIST1H4I | 403 |
| 1 | 229033700 | 229034766 | RHOU | 401 |
| 15 | 23452200 | 23452762 | MKRN3 | 401 |
| 17 | 73309007 | 73311096 | SLC25A19 | 401 |
| 10 | 34511495 | 34512233 | PARD3 | 401 |
| 16 | 11763331 | 11764299 | SNN | 400 |
| 8 | 8206504 | 8207218 | SGK223 | 400 |
| 15 | 72832059 | 72832716 | ARIH1 | 398 |
| 3 | 15502587 | 15503276 | COLQ | 397 |
| 7 | 18061116 | 18061828 | PRPS1L1 | 396 |
| 11 | 33182789 | 33183621 | CSTF3 | 395 |
| 18 | 60251465 | 60252419 | ZCCHC2 | 395 |
| 22 | 46770200 | 46770907 | CELSR1 | 395 |
| 15 | 67811989 | 67812722 | C15orf61 | 393 |
| 10 | 49892534 | 49893554 | WDFY4 | 393 |
| 5 | 75697628 | 75699535 | IQGAP2 | 392 |
| 13 | 107145177 | 107145658 | EFNB2 | 391 |
| 12 | 13253926 | 13254823 | GSG1 | 391 |
| 7 | 83645470 | 83645993 | SEMA3A | 391 |
| 8 | 144407956 | 144408811 | TOP1MT | 391 |
| 2 | 47948014 | 47948766 | MSH6 | 389 |
| 7 | 105751880 | 105752434 | SYPL1 | 389 |
| 3 | 5862813 | 5863315 | EDEM1 | 388 |
| 7 | 119159529 | 119160061 | KCND2 | 387 |
| 17 | 55952424 | 55954187 | CUEDC1 | 387 |
| 16 | 30119969 | 30120965 | GDPD3 | 386 |
| 5 | 17593326 | 17596256 | BASP1 | 386 |
| 10 | 104574791 | 104575504 | C10orf26 | 386 |
| 5 | 65220513 | 65221437 | ERBB2IP | 386 |
| 1 | 231829967 | 231830962 | DISC1 | 386 |
| 4 | 84401833 | 84402339 | FAM175A | 385 |
| 19 | 3141297 | 3142956 | GNA15 | 385 |
| 20 | 2654319 | 2654685 | EBF4 | 384 |
| 19 | 39322971 | 39324124 | ECH1 | 383 |
| 8 | 109194123 | 109194648 | EIF3E | 383 |
| 19 | 11378455 | 11380045 | TSPAN16 | 382 |
| 16 | 54316525 | 54317245 | IRX3 | 382 |
| 2 | 75872590 | 75873089 | MRPL19 | 382 |
| 17 | 56065777 | 56066457 | VEZF1 | 381 |
| 18 | 32620832 | 32621819 | MAPRE2 | 381 |
| 19 | 7267831 | 7268644 | INSR | 380 |
| 19 | 11380207 | 11381872 | TSPAN16 | 380 |
| 16 | 81591625 | 81592912 | CMIP | 380 |
| 2 | 33531126 | 33532034 | LTBP1 | 380 |
| 4 | 7648973 | 7652530 | SORCS2 | 378 |
| 18 | 74718086 | 74719260 | MBP | 377 |
| 21 | 22858136 | 22858679 | NCAM2 | 377 |
| 15 | 45004503 | 45004547 | B2M | 377 |
| 3 | 5261838 | 5262891 | EDEM1 | 377 |
| 3 | 13337129 | 13338463 | NUP210 | 377 |
| 2 | 85117877 | 85118350 | TMSB10 | 376 |
| 1 | 2187600 | 2187961 | SKI | 376 |
| 15 | 51058336 | 51059266 | SPPL2A | 376 |
| 6 | 12364061 | 12364508 | EDN1 | 375 |
| 1 | 9487652 | 9489317 | SLC25A33 | 374 |
| 1 | 150131338 | 150131798 | PLEKHO1 | 372 |
| 5 | 118842917 | 118843588 | HSD17B4 | 372 |
| 5 | 39575116 | 39575971 | DAB2 | 372 |
| 1 | 149803359 | 149804681 | HIST2H4A | 372 |
| 13 | 88874629 | 88875322 | SLITRK5 | 371 |
| 3 | 123234790 | 123235487 | PTPLB | 370 |
| 3 | 197601530 | 197602430 | LRCH3 | 369 |
| 16 | 84979588 | 84982779 | ZDHHC7 | 369 |
| 9 | 92079336 | 92079913 | SEMA4D | 368 |
| 21 | 14439385 | 14439931 | POTED | 368 |
| 21 | 47935255 | 47935817 | DIP2A | 367 |
| 2 | 43333995 | 43335103 | ZFP36L2 | 367 |
| 4 | 28608399 | 28608852 | STIM2 | 367 |
| 12 | 1921560 | 1922136 | CACNA2D4 | 365 |
| 12 | 1921560 | 1922136 | LRTM2 | 365 |
| 15 | 23014458 | 23015032 | NIPA2 | 365 |
| 13 | 31735261 | 31736651 | HSPH1 | 365 |
| 9 | 31953216 | 31953853 | ACO1 | 364 |
| 1 | 182583259 | 182584678 | RNASEL | 364 |
| 5 | 1313319 | 1314036 | TERT | 364 |
| 5 | 80735287 | 80735787 | SSBP2 | 363 |
| 3 | 42113130 | 42113860 | TRAK1 | 363 |
| 11 | 75961694 | 75961707 | WNT11 | 362 |
| 21 | 24343513 | 24343890 | NCAM2 | 361 |
| 3 | 150833036 | 150833966 | MED12L | 361 |
| 16 | 376387 | 377807 | AXIN1 | 361 |
| 19 | 45812500 | 45814812 | CKM | 361 |
| 19 | 57148631 | 57149119 | LOC147670 | 361 |
| 15 | 20936244 | 20936947 | POTEB | 360 |
| 10 | 665999 | 666092 | DIP2C | 359 |
| 8 | 8850045 | 8850912 | ERI1 | 359 |
| 2 | 24256716 | 24257713 | C2orf44 | 359 |
| 3 | 112554531 | 112555342 | CD200R1L | 358 |
| 21 | 30407677 | 30408304 | USP16 | 356 |
| 3 | 14480487 | 14481660 | SLC6A6 | 356 |
| 18 | 20651881 | 20652363 | CABLES1 | 356 |
| 4 | 164067529 | 164068014 | NAF1 | 355 |
| 17 | 55970183 | 55971933 | CUEDC1 | 354 |
| 15 | 29246638 | 29247389 | APBA2 | 353 |
| 1 | 26865203 | 26866756 | RPS6KA1 | 353 |
| 3 | 197824649 | 197825218 | FAM157A | 353 |
| 12 | 28547040 | 28547525 | CCDC91 | 353 |
| 6 | 131948827 | 131949898 | MED23 | 353 |
| 14 | 35821514 | 35822432 | NFKBIA | 352 |
| 6 | 5108126 | 5108480 | LYRM4 | 352 |
| 1 | 6085188 | 6087831 | KCNAB2 | 352 |
| 8 | 12344932 | 12345603 | FAM86B2 | 352 |
| 9 | 32069673 | 32070368 | ACO1 | 351 |
| 21 | 48117762 | 48118648 | PRMT2 | 351 |
| 5 | 65221573 | 65222028 | ERBB2IP | 351 |
| 13 | 78947270 | 78947834 | POU4F1 | 351 |
| 17 | 75865138 | 75866722 | TNRC6C | 350 |
| 17 | 36747366 | 36748779 | SRCIN1 | 349 |
| 9 | 125675628 | 125676808 | ZBTB6 | 349 |
| 11 | 116643133 | 116644144 | BUD13 | 348 |
| 1 | 42192379 | 42193771 | HIVEP3 | 347 |
| X | 71847454 | 71848057 | PHKA1 | 347 |
| 12 | 66569112 | 66569152 | IRAK3 | 347 |
| 4 | 103747539 | 103749269 | UBE2D3 | 347 |
| 3 | 10231236 | 10231961 | IRAK2 | 347 |
| 21 | 45665172 | 45666305 | ICOSLG | 346 |
| 3 | 180537519 | 180538178 | FXR1 | 345 |
| 6 | 34192584 | 34193491 | HMGA1 | 345 |
| 7 | 72625112 | 72629320 | NSUN5 | 345 |
| 7 | 26720591 | 26721116 | SKAP2 | 344 |
| 18 | 60428478 | 60428990 | PHLPP1 | 343 |
| 19 | 14896449 | 14898239 | EMR2 | 342 |
| 1 | 42205507 | 42207049 | HIVEP3 | 342 |
| 1 | 17539002 | 17539678 | PADI1 | 342 |
| 1 | 27647678 | 27649437 | TMEM222 | 342 |
| 15 | 50800974 | 50801967 | USP50 | 341 |
| 8 | 10832596 | 10833408 | XKR6 | 341 |
| 19 | 14886119 | 14888265 | EMR2 | 341 |
| 1 | 229125448 | 229126504 | RHOU | 341 |
| 17 | 3819209 | 3820112 | P2RX1 | 341 |
| 10 | 34494196 | 34495203 | PARD3 | 340 |
| 2 | 8593806 | 8594461 | ID2 | 340 |
| 4 | 2943086 | 2944460 | NOP14 | 340 |
| 6 | 85297387 | 85297590 | TBX18 | 340 |
| 3 | 160922907 | 160923802 | NMD3 | 339 |
| 3 | 183964300 | 183966185 | ALG3 | 339 |
| 1 | 54146492 | 54148212 | GLIS1 | 339 |
| 6 | 23913453 | 23914145 | NRSN1 | 339 |
| 1 | 51761705 | 51763212 | TTC39A | 339 |
| 7 | 5267691 | 5269613 | WIPI2 | 339 |
| 6 | 55986846 | 55987371 | COL21A1 | 339 |
| 7 | 150064918 | 150065761 | REPIN1 | 339 |
| 1 | 2186523 | 2186543 | SKI | 338 |
| 21 | 34897856 | 34898847 | GART | 338 |
| 9 | 27572638 | 27573522 | C9orf72 | 338 |
| 6 | 149196432 | 149197065 | UST | 338 |
| 3 | 153576355 | 153576956 | ARHGEF26 | 338 |
| 5 | 148619462 | 148620706 | ABLIM3 | 337 |
| 12 | 22661611 | 22662383 | KIAA0528 | 337 |
| 5 | 990704 | 991165 | LOC100506688 | 336 |
| 13 | 79290123 | 79290844 | RNF219 | 336 |
| 15 | 45314248 | 45315797 | SORD | 336 |
| 12 | 2548583 | 2549596 | CACNA1C | 336 |
| 10 | 109216675 | 109217099 | SORCS1 | 336 |
| 3 | 13268843 | 13270087 | IQSEC1 | 336 |
| 11 | 57988716 | 57989284 | OR1S2 | 335 |
| 6 | 100906382 | 100906781 | SIM1 | 335 |
| 15 | 31801838 | 31801843 | OTUD7A | 335 |
| 19 | 183250 | 183981 | OR4F17 | 334 |
| 19 | 33689871 | 33691242 | LRP3 | 334 |
| 18 | 23938199 | 23938763 | TAF4B | 333 |
| 7 | 73633069 | 73633714 | LAT2 | 333 |
| X | 49221122 | 49222078 | GAGE8 | 331 |
| X | 49221122 | 49222078 | GAGE2C | 331 |
| X | 49221122 | 49222078 | GAGE2A | 331 |
| X | 49221122 | 49222078 | GAGE5 | 331 |
| X | 49221122 | 49222078 | GAGE12F | 331 |
| X | 49221122 | 49222078 | GAGE12I | 331 |
| X | 49221122 | 49222078 | GAGE4 | 331 |
| X | 49221122 | 49222078 | GAGE7 | 331 |
| X | 49221122 | 49222078 | GAGE2D | 331 |
| 1 | 110090838 | 110091665 | GNAI3 | 331 |
| 21 | 40530232 | 40530842 | PSMG1 | 330 |
| 5 | 10635785 | 10636296 | ANKRD33B | 330 |
| 1 | 100817004 | 100819052 | CDC14A | 330 |
| 14 | 52931747 | 52932484 | TXNDC16 | 330 |
| 16 | 87051043 | 87051579 | C16orf95 | 329 |
| 12 | 783847 | 784759 | NINJ2 | 329 |
| 1 | 40723095 | 40724848 | ZMPSTE24 | 329 |
| 20 | 23122870 | 23123496 | CD93 | 329 |
| 2 | 99374081 | 99374950 | MGAT4A | 327 |
| 13 | 84904987 | 84905987 | SLITRK1 | 326 |
| 6 | 27065550 | 27066275 | HIST1H2BJ | 326 |
| 4 | 88359874 | 88360424 | NUDT9 | 326 |
| 19 | 56747185 | 56747199 | ZSCAN5A | 326 |
| 8 | 27683816 | 27684479 | PBK | 326 |
| 17 | 55949089 | 55952252 | CUEDC1 | 325 |
| 7 | 105493676 | 105494847 | ATXN7L1 | 325 |
| 6 | 27777597 | 27778531 | HIST1H3H | 325 |
| 6 | 117090726 | 117091156 | FAM162B | 325 |
| 15 | 91203668 | 91204402 | BLM | 325 |
| 3 | 181666491 | 181667099 | SOX2 | 325 |
| 13 | 87132319 | 87133275 | SLITRK6 | 324 |
| 1 | 149784368 | 149785351 | HIST2H2BF | 324 |
| 8 | 3611533 | 3612609 | CSMD1 | 324 |
| 2 | 9370190 | 9372104 | ASAP2 | 323 |
| 15 | 67157988 | 67158980 | SMAD6 | 323 |
| 5 | 145562455 | 145562874 | LARS | 323 |
| 8 | 9467082 | 9467762 | TNKS | 323 |
| 10 | 43914960 | 43916592 | HNRNPF | 322 |
| 10 | 1104768 | 1106169 | WDR37 | 322 |
| 5 | 112876674 | 112877156 | YTHDC2 | 322 |
| 14 | 55573513 | 55574296 | LGALS3 | 322 |
| 10 | 88769001 | 88769469 | AGAP11 | 320 |
| 19 | 21225316 | 21225707 | ZNF430 | 320 |
| 15 | 67366855 | 67367278 | SMAD3 | 320 |
| 17 | 18218053 | 18219147 | TOP3A | 320 |
| 3 | 58181767 | 58181802 | DNASE1L3 | 319 |
| 9 | 93955267 | 93956566 | AUH | 319 |
| 1 | 149222059 | 149223683 | HIST2H2BF | 319 |
| 6 | 67741105 | 67741280 | EYS | 319 |
| 6 | 122803564 | 122803918 | PKIB | 318 |
| 13 | 108915456 | 108915981 | TNFSF13B | 318 |
| 6 | 35264866 | 35266521 | DEF6 | 318 |
| 6 | 97711347 | 97711974 | MMS22L | 317 |
| 5 | 43033778 | 43034850 | C5orf39 | 317 |
| 9 | 134459913 | 134460163 | RAPGEF1 | 317 |
| 11 | 10644692 | 10645558 | MRVI1 | 317 |
| 13 | 29211331 | 29211373 | POMP | 317 |
| 1 | 7186776 | 7187331 | CAMTA1 | 317 |
| 1 | 173568051 | 173568877 | SLC9A11 | 315 |
| 21 | 46571993 | 46572704 | ADARB1 | 315 |
| 2 | 24956119 | 24956535 | NCOA1 | 315 |
| 2 | 9539308 | 9540186 | ASAP2 | 315 |
| 1 | 27018718 | 27020077 | ARID1A | 314 |
| 19 | 8880041 | 8881111 | OR2Z1 | 314 |
| 2 | 113119702 | 113120367 | RGPD5 | 314 |
| 22 | 39146912 | 39148708 | SUN2 | 314 |
| 2 | 54900373 | 54901654 | SPTBN1 | 314 |
| 13 | 77891513 | 77892986 | MYCBP2 | 314 |
| 8 | 62749124 | 62749668 | ASPH | 313 |
| 21 | 38629132 | 38631234 | DSCR3 | 313 |
| 12 | 20568176 | 20568636 | PDE3A | 313 |
| 11 | 118229560 | 118230456 | UBE4A | 313 |
| 14 | 104072832 | 104074595 | KLC1 | 313 |
| 3 | 100026950 | 100027443 | TBC1D23 | 313 |
| 12 | 46123471 | 46124162 | ARID2 | 312 |
| 7 | 157460122 | 157463365 | PTPRN2 | 312 |
| 20 | 656673 | 657855 | SCRT2 | 312 |
| 3 | 127452661 | 127453456 | MGLL | 312 |
| 5 | 60704392 | 60704964 | ZSWIM6 | 312 |
| 4 | 10438065 | 10438630 | ZNF518B | 312 |
| 7 | 18977588 | 18978354 | HDAC9 | 311 |
| 5 | 39575069 | 39575101 | DAB2 | 311 |
| 4 | 21440365 | 21440884 | KCNIP4 | 311 |
| 11 | 128564871 | 128565247 | FLI1 | 311 |
| 17 | 1667373 | 1668215 | SERPINF1 | 310 |
| 5 | 178053506 | 178054219 | CLK4 | 310 |
| 1 | 161135543 | 161136532 | PPOX | 310 |
| 3 | 195257162 | 195257951 | PPP1R2 | 309 |
| 16 | 17437958 | 17438060 | XYLT1 | 309 |
| 7 | 22084238 | 22084793 | CDCA7L | 309 |
| 20 | 1471392 | 1472222 | SIRPB2 | 309 |
| 18 | 40209278 | 40209892 | RIT2 | 309 |
| 2 | 46475947 | 46476572 | EPAS1 | 308 |
| 5 | 100146339 | 100147087 | ST8SIA4 | 308 |
| 3 | 142423225 | 142423896 | PLS1 | 308 |
| 4 | 183729958 | 183730851 | DCTD | 308 |
| 8 | 142097272 | 142097280 | DENND3 | 308 |
| 18 | 13464654 | 13465168 | C18orf1 | 308 |
| 5 | 171839921 | 171840389 | SH3PXD2B | 308 |
| 9 | 95538218 | 95538374 | BICD2 | 308 |
| 3 | 150946924 | 150948215 | P2RY14 | 308 |
| 3 | 150946924 | 150948215 | MED12L | 308 |
| 15 | 42198545 | 42199503 | EHD4 | 307 |
| 2 | 70087781 | 70088323 | GMCL1 | 307 |
| 22 | 18632319 | 18633112 | USP18 | 307 |
| 15 | 32871849 | 32872989 | ARHGAP11A | 306 |
| 6 | 26031631 | 26032379 | HIST1H3B | 306 |
| 15 | 90436713 | 90438224 | AP3S2 | 306 |
| 15 | 90436713 | 90438224 | C15orf38-AP3S2 | 306 |
| 19 | 54900452 | 54901236 | TTYH1 | 306 |
| 18 | 12838122 | 12838876 | PTPN2 | 306 |
| 17 | 76413245 | 76414303 | PGS1 | 306 |
| 11 | 75961898 | 75962670 | WNT11 | 306 |
| 8 | 25202786 | 25203669 | DOCK5 | 306 |
| 5 | 131801934 | 131802910 | IRF1 | 306 |
| 17 | 30602565 | 30603191 | RHBDL3 | 305 |
| 7 | 5269695 | 5275008 | WIPI2 | 305 |
| 6 | 34231846 | 34232714 | C6orf1 | 305 |
| 2 | 37259920 | 37260494 | HEATR5B | 305 |
| 3 | 167464985 | 167465669 | SERPINI1 | 304 |
| 20 | 31252732 | 31253534 | C20orf203 | 304 |
| 7 | 73640918 | 73642353 | LAT2 | 304 |
| 6 | 149081856 | 149083008 | UST | 304 |
| 17 | 17654752 | 17655474 | RAI1 | 304 |
| 1 | 145022305 | 145023374 | PDE4DIP | 303 |
| 8 | 19623942 | 19624272 | INTS10 | 303 |
| 1 | 26948447 | 26950119 | ARID1A | 303 |
| 1 | 112049831 | 112050941 | ADORA3 | 302 |
| 10 | 72125377 | 72125426 | LRRC20 | 302 |
| 6 | 3139511 | 3139977 | BPHL | 302 |
| 12 | 123458838 | 123460300 | OGFOD2 | 301 |
| 8 | 22572586 | 22574040 | PEBP4 | 301 |
| 10 | 73505862 | 73508300 | C10orf54 | 301 |
| 10 | 73505862 | 73508300 | C10orf105 | 301 |
| 10 | 73505862 | 73508300 | CDH23 | 301 |
| 8 | 145051873 | 145052658 | PLEC | 301 |
| 1 | 115604955 | 115605713 | TSPAN2 | 301 |
| 15 | 69859017 | 69859613 | RPLP1 | 301 |
| 17 | 34739570 | 34740564 | TBC1D3H | 301 |
| 1 | 15103319 | 15104151 | KAZN | 301 |
| 3 | 194364571 | 194365311 | LSG1 | 300 |
| 8 | 4507407 | 4508154 | CSMD1 | 300 |
| 6 | 37158823 | 37160427 | PIM1 | 300 |
| 22 | 31957114 | 31957695 | SFI1 | 300 |
| 8 | 7913044 | 7914295 | LOC100132396 | 300 |
| 10 | 73426565 | 73427139 | CDH23 | 300 |
| 14 | 102001671 | 102002627 | DIO3 | 300 |
| 7 | 50423066 | 50423663 | IKZF1 | 299 |
| 7 | 152104260 | 152105942 | MLL3 | 299 |
| 6 | 118110862 | 118111443 | NUS1 | 299 |
| 7 | 44573874 | 44574480 | NPC1L1 | 299 |
| 5 | 59995215 | 59996359 | DEPDC1B | 299 |
| 6 | 170585835 | 170587455 | FAM120B | 299 |
| 5 | 145421085 | 145421478 | SH3RF2 | 299 |
| 20 | 52510161 | 52510212 | BCAS1 | 298 |
| 13 | 84363140 | 84364142 | SLITRK1 | 298 |
| 10 | 12380359 | 12380758 | CAMK1D | 298 |
| 11 | 111492861 | 111493521 | SIK2 | 298 |
| 1 | 236117749 | 236118510 | LYST | 298 |
| 3 | 151671054 | 151671669 | SUCNR1 | 298 |
| 7 | 74515987 | 74516183 | GTF2IRD2B | 298 |
| 7 | 104652285 | 104654240 | MLL5 | 297 |
| 21 | 36180174 | 36180893 | RUNX1 | 297 |
| 6 | 158400520 | 158401079 | SYNJ2 | 297 |
| 1 | 93645174 | 93647591 | TMED5 | 297 |
| 19 | 56747202 | 56747915 | ZSCAN5A | 297 |
| 11 | 34175573 | 34176372 | ABTB2 | 297 |
| 13 | 89504236 | 89505105 | SLITRK5 | 297 |
| X | 135606268 | 135606818 | VGLL1 | 297 |
| 9 | 69755577 | 69756039 | ANKRD20A4 | 297 |
| 3 | 183364412 | 183365909 | KLHL24 | 297 |
| 18 | 77385058 | 77387285 | CTDP1 | 296 |
| 12 | 2609158 | 2611591 | CACNA1C | 296 |
| 9 | 13087572 | 13088233 | MPDZ | 296 |
| 3 | 58522481 | 58522614 | ACOX2 | 296 |
| 19 | 30171015 | 30172213 | PLEKHF1 | 296 |
| 3 | 41733236 | 41733358 | ULK4 | 296 |
| 1 | 153329637 | 153330469 | S100A9 | 295 |
| 6 | 38410294 | 38410417 | BTBD9 | 295 |
| 13 | 89807006 | 89807934 | SLITRK5 | 295 |
| 10 | 13759842 | 13760678 | FRMD4A | 295 |
| 21 | 34545517 | 34546156 | IFNAR2 | 295 |
| 3 | 181614779 | 181616047 | SOX2 | 295 |
| 16 | 48569869 | 48571183 | N4BP1 | 295 |
| 5 | 61675316 | 61675561 | KIF2A | 295 |
| 17 | 72595000 | 72595813 | CD300LD | 295 |
| 15 | 43529946 | 43531651 | TGM5 | 294 |
| 21 | 45899214 | 45899278 | LRRC3 | 294 |
| 2 | 58393926 | 58394479 | FANCL | 294 |
| 2 | 112718620 | 112718962 | MERTK | 294 |
| 12 | 125424071 | 125424825 | UBC | 294 |
| 13 | 76062802 | 76063943 | TBC1D4 | 293 |
| 4 | 31376952 | 31377431 | PCDH7 | 293 |
| 1 | 229265063 | 229265703 | RAB4A | 293 |
| 1 | 144613919 | 144614668 | NBPF9 | 293 |
| 7 | 26332343 | 26333433 | SNX10 | 293 |
| 7 | 102539206 | 102539488 | FBXL13 | 292 |
| 7 | 102539206 | 102539488 | LRRC17 | 292 |
| 4 | 47002975 | 47003522 | GABRA4 | 292 |
| 20 | 57580057 | 57580549 | CTSZ | 292 |
| 8 | 17739422 | 17740001 | FGL1 | 292 |
| 15 | 51368654 | 51369864 | TNFAIP8L3 | 292 |
| 7 | 107268768 | 107270085 | SLC26A4 | 292 |
| 6 | 21704784 | 21705383 | SOX4 | 291 |
| 16 | 87051869 | 87053038 | C16orf95 | 291 |
| 7 | 12696088 | 12696648 | SCIN | 290 |
| 15 | 43397700 | 43398477 | UBR1 | 290 |
| 6 | 158974795 | 158975675 | TMEM181 | 290 |
| 1 | 149831977 | 149833482 | HIST2H4A | 290 |
| 3 | 128321947 | 128323510 | C3orf27 | 290 |
| 8 | 94892739 | 94893302 | PDP1 | 289 |
| 11 | 111957016 | 111957819 | SDHD | 289 |
| 7 | 75148624 | 75148941 | SPDYE5 | 289 |
| 17 | 58180367 | 58180965 | HEATR6 | 289 |
| 14 | 50703011 | 50703626 | SOS2 | 288 |
| 3 | 197426389 | 197427183 | KIAA0226 | 288 |
| 10 | 134007536 | 134008301 | DPYSL4 | 288 |
| 13 | 79263186 | 79264016 | RNF219 | 287 |
| 3 | 39770455 | 39771009 | MYRIP | 287 |
| 20 | 35806545 | 35807105 | RPN2 | 287 |
| 20 | 35806545 | 35807105 | C20orf132 | 287 |
| 17 | 45144426 | 45145004 | RPRML | 286 |
| 3 | 186264016 | 186264917 | TBCCD1 | 286 |
| 1 | 25348922 | 25349881 | RUNX3 | 286 |
| 19 | 35503858 | 35504171 | GRAMD1A | 286 |
| 9 | 69498144 | 69498832 | ANKRD20A4 | 286 |
| 2 | 16872386 | 16872862 | FAM49A | 285 |
| 20 | 43594647 | 43595515 | STK4 | 285 |
| 6 | 42301003 | 42301430 | TRERF1 | 285 |
| 19 | 39155152 | 39156133 | ACTN4 | 285 |
| 1 | 55006314 | 55008811 | ACOT11 | 285 |
| 4 | 3287174 | 3289381 | RGS12 | 284 |
| 11 | 102574163 | 102574523 | MMP27 | 284 |
| 8 | 48513717 | 48513974 | KIAA0146 | 284 |
| 8 | 7598731 | 7599890 | FAM90A10 | 284 |
| 15 | 93721513 | 93722247 | RGMA | 284 |
| 22 | 50628551 | 50629897 | TRABD | 283 |
| 15 | 75080964 | 75083336 | CSK | 283 |
| 15 | 75079006 | 75080539 | CSK | 283 |
| 15 | 31652270 | 31654176 | KLF13 | 283 |
| 19 | 6674391 | 6675309 | TNFSF14 | 283 |
| 18 | 57567248 | 57568047 | PMAIP1 | 283 |
| 11 | 8709888 | 8710873 | RPL27A | 283 |
| 6 | 82461222 | 82461990 | FAM46A | 282 |
| 15 | 74726117 | 74727887 | SEMA7A | 282 |
| 5 | 132065691 | 132066470 | KIF3A | 282 |
| 18 | 58352269 | 58352730 | MC4R | 282 |
| 21 | 26980280 | 26980293 | MRPL39 | 282 |
| 19 | 53026597 | 53027192 | ZNF808 | 281 |
| 12 | 7062266 | 7064093 | PTPN6 | 281 |
| 3 | 183390255 | 183391092 | KLHL24 | 281 |
| 14 | 45719745 | 45720336 | MIS18BP1 | 281 |
| 14 | 61201189 | 61201885 | MNAT1 | 281 |
| 7 | 16036251 | 16036848 | MEOX2 | 281 |
| 13 | 74381045 | 74381488 | KLF12 | 280 |
| 6 | 38762789 | 38763517 | DNAH8 | 280 |
| 6 | 26188291 | 26189449 | HIST1H2AE | 280 |
| 5 | 158689850 | 158690902 | UBLCP1 | 279 |
| 4 | 1096174 | 1096878 | RNF212 | 279 |
| 15 | 59430082 | 59430838 | MYO1E | 279 |
| 2 | 26226456 | 26227255 | RAB10 | 279 |
| 3 | 8699805 | 8700449 | C3orf32 | 278 |
| 1 | 25359263 | 25361286 | RUNX3 | 278 |
| 6 | 42628269 | 42629108 | UBR2 | 278 |
| 7 | 106301123 | 106301828 | C7orf74 | 278 |
| 4 | 16032209 | 16032779 | PROM1 | 277 |
| 14 | 24804587 | 24804954 | ADCY4 | 277 |
| 8 | 10993523 | 10994059 | XKR6 | 277 |
| 6 | 20320096 | 20320881 | E2F3 | 277 |
| 21 | 19102325 | 19102888 | C21orf91 | 277 |
| 9 | 93926195 | 93927583 | AUH | 277 |
| 15 | 50606173 | 50606931 | GABPB1 | 277 |
| 8 | 6148064 | 6148486 | MCPH1 | 277 |
| 8 | 12756124 | 12756664 | KIAA1456 | 277 |
| 13 | 78948096 | 78948684 | POU4F1 | 277 |
| 6 | 26045542 | 26046109 | HIST1H2BB | 276 |
| 9 | 17566652 | 17567221 | SH3GL2 | 276 |
| 16 | 4566425 | 4566459 | C16orf5 | 276 |
| 15 | 90436601 | 90436611 | AP3S2 | 276 |
| 15 | 90436601 | 90436611 | C15orf38-AP3S2 | 276 |
| 12 | 132646941 | 132648355 | DDX51 | 276 |
| 13 | 85455738 | 85456475 | SLITRK6 | 275 |
| 15 | 32907472 | 32908539 | ARHGAP11A | 275 |
| 17 | 43370200 | 43371449 | MAP3K14 | 275 |
| 13 | 74794909 | 74795571 | KLF12 | 274 |
| 3 | 182774309 | 182775186 | MCCC1 | 274 |
| 19 | 14486064 | 14488014 | CD97 | 274 |
| 16 | 81593237 | 81594463 | CMIP | 274 |
| 10 | 52381516 | 52382444 | SGMS1 | 274 |
| 16 | 33039463 | 33039472 | TP53TG3 | 273 |
| 13 | 110052403 | 110052937 | IRS2 | 273 |
| 6 | 15283434 | 15284376 | JARID2 | 273 |
| 16 | 84983819 | 84984249 | ZDHHC7 | 273 |
| 1 | 947994 | 949408 | ISG15 | 273 |
| 11 | 114030555 | 114031053 | ZBTB16 | 273 |
| 3 | 185629060 | 185630771 | TRA2B | 273 |
| 19 | 14466690 | 14467941 | CD97 | 273 |
| 12 | 718593 | 720140 | NINJ2 | 273 |
| 5 | 61985515 | 61986050 | LRRC70 | 273 |
| 4 | 185425474 | 185425882 | IRF2 | 273 |
| 1 | 116213296 | 116214302 | VANGL1 | 273 |
| 18 | 3149912 | 3150458 | MYOM1 | 273 |
| 5 | 142260332 | 142260854 | ARHGAP26 | 272 |
| 10 | 6316906 | 6317923 | PFKFB3 | 272 |
| 1 | 112050947 | 112051845 | ADORA3 | 272 |
| 6 | 15299684 | 15300103 | JARID2 | 272 |
| 1 | 8164887 | 8165605 | ERRFI1 | 272 |
| 7 | 47537209 | 47538072 | TNS3 | 272 |
| 3 | 9523378 | 9524004 | SETD5 | 272 |
| X | 129225839 | 129226498 | ELF4 | 272 |
| 15 | 35331509 | 35332117 | ZNF770 | 272 |
| 16 | 86997047 | 86998321 | C16orf95 | 272 |
| 15 | 75416616 | 75417377 | C15orf39 | 272 |
| 8 | 7305379 | 7306810 | SPAG11B | 272 |
| 4 | 83271274 | 83271725 | HNRNPD | 272 |
| 13 | 76380725 | 76380822 | LMO7 | 271 |
| 10 | 73648352 | 73649004 | PSAP | 271 |
| 11 | 126337633 | 126338075 | KIRREL3 | 271 |
| X | 54435074 | 54435559 | TSR2 | 271 |
| 10 | 82258329 | 82260839 | TSPAN14 | 271 |
| 8 | 98858382 | 98858816 | LAPTM4B | 271 |
| 1 | 156095735 | 156096665 | LMNA | 271 |
| 14 | 51088883 | 51089523 | ATL1 | 271 |
| 1 | 97190687 | 97192279 | PTBP2 | 271 |
| 6 | 143166605 | 143167539 | HIVEP2 | 271 |
| 5 | 93196146 | 93196274 | FAM172A | 270 |
| 19 | 42375163 | 42378321 | LYPD4 | 270 |
| 2 | 74006630 | 74007408 | DUSP11 | 270 |
| 15 | 20560600 | 20560603 | GOLGA6L6 | 270 |
| 16 | 33038412 | 33039390 | TP53TG3 | 270 |
| 13 | 92078523 | 92078969 | GPC5 | 270 |
| 15 | 94924442 | 94924912 | MCTP2 | 270 |
| 3 | 4794541 | 4794923 | ITPR1 | 270 |
| 5 | 120197487 | 120198154 | PRR16 | 270 |
| 12 | 50794419 | 50795099 | LARP4 | 270 |
| X | 53710750 | 53711566 | HUWE1 | 270 |
| 10 | 75624859 | 75626617 | CAMK2G | 270 |
| 3 | 15368443 | 15369035 | SH3BP5 | 269 |
| 10 | 134045346 | 134046868 | STK32C | 269 |
| 1 | 32532508 | 32533744 | TMEM39B | 269 |
| 21 | 38592521 | 38594011 | DSCR3 | 269 |
| 3 | 127496789 | 127497519 | MGLL | 269 |
| 10 | 72123981 | 72125277 | LRRC20 | 269 |
| 3 | 72149719 | 72150347 | PROK2 | 268 |
| 12 | 2871855 | 2872273 | FKBP4 | 268 |
| 1 | 22108918 | 22109409 | USP48 | 268 |
| 15 | 31506782 | 31507856 | LOC283710 | 268 |
| 11 | 59577297 | 59578551 | MRPL16 | 267 |
| 12 | 111618476 | 111619645 | CUX2 | 267 |
| 6 | 27781599 | 27784226 | HIST1H2AJ | 267 |
| 12 | 62653620 | 62654519 | USP15 | 267 |
| 11 | 58932371 | 58933025 | DTX4 | 267 |
| 9 | 130624190 | 130625190 | ENG | 267 |
| 2 | 112761460 | 112761659 | MERTK | 267 |
| 9 | 3803165 | 3803695 | RFX3 | 267 |
| 15 | 63789202 | 63789832 | USP3 | 266 |
| 6 | 158687141 | 158687541 | TULP4 | 266 |
| 11 | 94965080 | 94965757 | SESN3 | 266 |
| 17 | 48556772 | 48556940 | RSAD1 | 266 |
| 15 | 93722770 | 93722992 | RGMA | 266 |
| 16 | 66641864 | 66642838 | CMTM3 | 266 |
| 1 | 181103106 | 181104516 | IER5 | 266 |
| 19 | 14898353 | 14898779 | EMR2 | 266 |
| 15 | 65242961 | 65243392 | ANKDD1A | 266 |
| 1 | 16840493 | 16841211 | NECAP2 | 266 |
| 5 | 148004934 | 148005732 | HTR4 | 266 |
| 17 | 46638265 | 46638579 | HOXB3 | 266 |
| 2 | 10549071 | 10550137 | HPCAL1 | 265 |
| 22 | 22296692 | 22297520 | PPM1F | 265 |
| 17 | 65252705 | 65253504 | HELZ | 265 |
| 19 | 39154884 | 39154939 | ACTN4 | 265 |
| 19 | 35503034 | 35503829 | GRAMD1A | 265 |
| 5 | 69789883 | 69791344 | SERF1A | 265 |
| 10 | 111693426 | 111693637 | XPNPEP1 | 264 |
| 11 | 134455462 | 134456021 | B3GAT1 | 264 |
| 11 | 65608411 | 65609293 | SNX32 | 264 |
| 1 | 25425415 | 25425434 | SYF2 | 264 |
| 20 | 39637382 | 39637958 | TOP1 | 264 |
| 18 | 74767551 | 74768244 | MBP | 264 |
| 1 | 39995504 | 39996558 | BMP8A | 263 |
| 7 | 75038475 | 75040432 | TRIM74 | 263 |
| 6 | 155053617 | 155055469 | SCAF8 | 263 |
| 19 | 8881527 | 8883327 | OR2Z1 | 263 |
| 1 | 35641960 | 35642761 | SFPQ | 263 |
| 21 | 34304797 | 34305757 | OLIG2 | 263 |
| 10 | 121128604 | 121129915 | GRK5 | 263 |
| 1 | 180895493 | 180896038 | KIAA1614 | 263 |
| 10 | 3598254 | 3599003 | KLF6 | 262 |
| 3 | 101395235 | 101396177 | ZBTB11 | 262 |
| 7 | 4991507 | 4992514 | MMD2 | 262 |
| 6 | 68272316 | 68273054 | BAI3 | 262 |
| 18 | 13426182 | 13426206 | C18orf1 | 262 |
| 1 | 7958230 | 7958756 | TNFRSF9 | 261 |
| 3 | 190294243 | 190295117 | IL1RAP | 261 |
| 7 | 119530491 | 119531049 | KCND2 | 261 |
| 1 | 41009003 | 41009519 | ZNF684 | 261 |
| X | 129216032 | 129216751 | ELF4 | 261 |
| 1 | 54186376 | 54187417 | GLIS1 | 261 |
| 8 | 124168926 | 124170243 | FAM83A | 261 |
| 14 | 65099350 | 65100631 | PLEKHG3 | 261 |
| 17 | 75878805 | 75879007 | TNRC6C | 261 |
| 3 | 107723116 | 107723764 | CD47 | 261 |
| 1 | 77824369 | 77825017 | AK5 | 260 |
| 4 | 21095281 | 21095741 | KCNIP4 | 260 |
| 17 | 34412723 | 34413243 | CCL4 | 260 |
| 16 | 84660540 | 84661582 | KLHL36 | 260 |
| 8 | 82470380 | 82470500 | FABP12 | 260 |
| 19 | 1384736 | 1384840 | NDUFS7 | 260 |
| 2 | 100602145 | 100602762 | AFF3 | 260 |
| 12 | 51983957 | 51984417 | SCN8A | 260 |
| 6 | 70043384 | 70043895 | BAI3 | 259 |
| 7 | 130915444 | 130916130 | MKLN1 | 259 |
| 1 | 35875785 | 35877732 | ZMYM4 | 259 |
| 15 | 58602765 | 58603065 | LIPC | 259 |
| 16 | 84664041 | 84664669 | KLHL36 | 259 |
| 16 | 12740598 | 12741068 | CPPED1 | 258 |
| 8 | 104079092 | 104080059 | ATP6V1C1 | 258 |
| 7 | 75040537 | 75040577 | TRIM74 | 258 |
| 18 | 32869920 | 32870669 | ZSCAN30 | 258 |
| 13 | 114895059 | 114895113 | RASA3 | 258 |
| 19 | 34941382 | 34941872 | UBA2 | 258 |
| 18 | 20520943 | 20521544 | RBBP8 | 258 |
| 2 | 87005150 | 87006014 | RMND5A | 258 |
| 6 | 123126650 | 123127031 | SMPDL3A | 258 |
| 8 | 17940744 | 17942583 | ASAH1 | 258 |
| 3 | 166962697 | 166963210 | ZBBX | 258 |
| 1 | 94560254 | 94561598 | ABCA4 | 258 |
| 10 | 126276323 | 126278154 | LHPP | 258 |
| 5 | 39454733 | 39455367 | DAB2 | 257 |
| 6 | 110953581 | 110954637 | CDK19 | 257 |
| 6 | 26195630 | 26197568 | HIST1H3E | 257 |
| 6 | 91321600 | 91322277 | MAP3K7 | 257 |
| 6 | 148800444 | 148801260 | SASH1 | 257 |
| 8 | 107707389 | 107708078 | OXR1 | 257 |
| 22 | 50630004 | 50630961 | TRABD | 257 |
| 5 | 17591810 | 17592549 | BASP1 | 257 |
| 1 | 108720380 | 108721490 | SLC25A24 | 257 |
| 16 | 11146185 | 11146831 | CLEC16A | 257 |
| 17 | 76411606 | 76411901 | PGS1 | 257 |
| 6 | 35016061 | 35016187 | ANKS1A | 256 |
| 3 | 182780001 | 182781084 | MCCC1 | 256 |
| 15 | 23212266 | 23213025 | NIPA1 | 256 |
| 13 | 79333009 | 79333726 | RNF219 | 256 |
| 18 | 47908034 | 47908380 | SKA1 | 256 |
| 10 | 22102590 | 22102763 | DNAJC1 | 256 |
| 20 | 2930801 | 2931398 | PTPRA | 256 |
| 14 | 93702111 | 93702687 | C14orf142 | 256 |
| 3 | 153179524 | 153180220 | C3orf79 | 256 |
| 8 | 7583337 | 7584557 | FAM90A19 | 256 |
| 10 | 97507644 | 97508070 | ENTPD1 | 256 |
| 1 | 234742729 | 234743602 | IRF2BP2 | 255 |
| 15 | 85800482 | 85801505 | AKAP13 | 255 |
| 7 | 155153094 | 155153795 | INSIG1 | 255 |
| 11 | 128968064 | 128968536 | ARHGAP32 | 255 |
| 15 | 99127969 | 99128883 | IGF1R | 255 |
| 6 | 26568237 | 26568748 | ABT1 | 255 |
| 3 | 151693103 | 151693709 | SUCNR1 | 255 |
| 15 | 28910649 | 28911401 | APBA2 | 254 |
| 1 | 71747152 | 71747718 | ZRANB2 | 254 |
| 16 | 80857128 | 80857319 | CDYL2 | 254 |
| 4 | 31203387 | 31204006 | PCDH7 | 254 |
| 7 | 73642375 | 73643431 | LAT2 | 254 |
| 2 | 98912685 | 98913322 | VWA3B | 254 |
| 15 | 20545310 | 20548198 | GOLGA6L6 | 254 |
| 1 | 227150990 | 227151665 | ADCK3 | 254 |
| 15 | 70558470 | 70559942 | TLE3 | 254 |
| 5 | 74100996 | 74101579 | FAM169A | 254 |
| 17 | 54877285 | 54877818 | C17orf67 | 254 |
| X | 30596119 | 30596400 | CXorf21 | 254 |
| 3 | 171711506 | 171711955 | FNDC3B | 254 |
| 7 | 47535329 | 47536624 | TNS3 | 254 |
| 1 | 118147748 | 118149757 | FAM46C | 254 |
| 11 | 75488478 | 75489140 | DGAT2 | 253 |
| 6 | 160113119 | 160113775 | SOD2 | 253 |
| 2 | 87729918 | 87730547 | PLGLB2 | 253 |
| 19 | 2274373 | 2274554 | JSRP1 | 253 |
| 12 | 46120053 | 46122027 | ARID2 | 253 |
| 1 | 22073986 | 22074512 | USP48 | 253 |
| 4 | 49288213 | 49289369 | CWH43 | 253 |
| 10 | 101723639 | 101723789 | DNMBP | 253 |
| 5 | 37386857 | 37387263 | WDR70 | 252 |
| 3 | 12254846 | 12255419 | TIMP4 | 252 |
| 10 | 64348000 | 64348507 | ZNF365 | 252 |
| 17 | 74190646 | 74190849 | RNF157 | 252 |
| 19 | 2270941 | 2274262 | OAZ1 | 252 |
| 9 | 95860436 | 95861602 | C9orf89 | 252 |
| 1 | 92941345 | 92942181 | GFI1 | 252 |
| 4 | 36218638 | 36219188 | ARAP2 | 252 |
| 1 | 1440097 | 1440734 | ATAD3A | 252 |
| 14 | 50704459 | 50705169 | SOS2 | 252 |
| 12 | 46122227 | 46123401 | ARID2 | 251 |
| 11 | 64528556 | 64530089 | PYGM | 251 |
| 10 | 116530458 | 116531000 | FAM160B1 | 251 |
| 2 | 42863691 | 42864163 | MTA3 | 251 |
| 4 | 3558067 | 3559550 | LRPAP1 | 251 |
| 7 | 2847332 | 2848472 | GNA12 | 250 |
| 21 | 40356937 | 40357631 | ETS2 | 250 |
| 12 | 50418877 | 50419500 | RACGAP1 | 250 |
| 12 | 122124593 | 122125699 | TMEM120B | 250 |
| 18 | 13222185 | 13223003 | C18orf1 | 250 |
| 6 | 65090494 | 65090980 | EYS | 250 |
| 14 | 92973390 | 92973794 | RIN3 | 250 |
| 11 | 66188701 | 66189773 | NPAS4 | 250 |
| 12 | 40402023 | 40402658 | SLC2A13 | 249 |
| 1 | 27787186 | 27788434 | WASF2 | 249 |
| 5 | 81062835 | 81063283 | SSBP2 | 249 |
| 3 | 177711703 | 177712434 | KCNMB2 | 249 |
| 10 | 88607461 | 88608068 | BMPR1A | 249 |
| 1 | 46805519 | 46806746 | NSUN4 | 249 |
| 7 | 35335300 | 35335854 | TBX20 | 249 |
| 6 | 155272047 | 155272901 | TIAM2 | 249 |
| 2 | 73748352 | 73749025 | ALMS1 | 249 |
| 4 | 38141336 | 38141774 | TBC1D1 | 249 |
| 7 | 5256735 | 5257966 | WIPI2 | 249 |
| 9 | 134391883 | 134392176 | POMT1 | 249 |
| 19 | 16437924 | 16438636 | KLF2 | 248 |
| 10 | 126276105 | 126276256 | LHPP | 248 |
| 16 | 76008638 | 76009270 | CNTNAP4 | 248 |
| 15 | 31508442 | 31509101 | LOC283710 | 248 |
| 4 | 170213950 | 170214530 | SH3RF1 | 248 |
| 12 | 54349862 | 54350943 | HOXC10 | 247 |
| 2 | 25154214 | 25154878 | ADCY3 | 247 |
| 1 | 15741918 | 15743132 | EFHD2 | 247 |
| 1 | 109641345 | 109644353 | TAF13 | 247 |
| 5 | 161463014 | 161463922 | GABRG2 | 247 |
| 8 | 105433207 | 105434080 | DPYS | 247 |
| 8 | 26447102 | 26448193 | DPYSL2 | 247 |
| 4 | 17037383 | 17038125 | LDB2 | 247 |
| 12 | 20481381 | 20482088 | PDE3A | 247 |
| 3 | 14347088 | 14347696 | SLC6A6 | 247 |
| 13 | 78699040 | 78699658 | EDNRB | 247 |
| 6 | 137112318 | 137113241 | MAP3K5 | 247 |
| 18 | 74766669 | 74767475 | MBP | 246 |
| 22 | 36007634 | 36007967 | MB | 246 |
| 17 | 61911217 | 61912104 | SMARCD2 | 246 |
| 2 | 220109902 | 220110717 | GLB1L | 246 |
| 3 | 157501000 | 157501609 | C3orf55 | 246 |
| 12 | 12660485 | 12660947 | DUSP16 | 246 |
| 17 | 61725742 | 61726543 | MAP3K3 | 246 |
| 14 | 105842297 | 105842877 | PACS2 | 246 |
| 7 | 131601424 | 131602148 | PODXL | 246 |
| 4 | 42096875 | 42097534 | BEND4 | 246 |
| 3 | 180706124 | 180706855 | DNAJC19 | 246 |
| 17 | 74368770 | 74369999 | PRPSAP1 | 246 |
| 18 | 37295406 | 37296055 | CELF4 | 245 |
| 1 | 199836401 | 199836822 | NR5A2 | 245 |
| 8 | 70138770 | 70139312 | SULF1 | 245 |
| 10 | 49679011 | 49679032 | ARHGAP22 | 245 |
| 17 | 76339339 | 76340374 | SOCS3 | 245 |
| X | 33051411 | 33051904 | DMD | 245 |
| 5 | 88144487 | 88145054 | MEF2C | 245 |
| 1 | 206645720 | 206646527 | IKBKE | 245 |
| 8 | 104431656 | 104431726 | SLC25A32 | 245 |
| 8 | 104431656 | 104431726 | DCAF13 | 245 |
| 12 | 38807611 | 38808386 | ALG10B | 245 |
| 13 | 88300028 | 88301234 | SLITRK5 | 244 |
| 18 | 11918212 | 11919089 | MPPE1 | 244 |
| 1 | 109704470 | 109704793 | KIAA1324 | 244 |
| 17 | 7017789 | 7018491 | ASGR2 | 244 |
| 10 | 13628477 | 13629976 | PRPF18 | 244 |
| X | 103401340 | 103401847 | MCART6 | 244 |
| 14 | 105114155 | 105115144 | INF2 | 244 |
| 6 | 7254974 | 7255824 | RREB1 | 244 |
| 13 | 41703621 | 41704190 | KBTBD6 | 244 |
| 8 | 6841809 | 6842736 | DEFA1 | 244 |
| 18 | 720894 | 721730 | ENOSF1 | 244 |
| 15 | 35261273 | 35262493 | AQR | 244 |
| 8 | 57470370 | 57470846 | PENK | 244 |
| 1 | 35280186 | 35280928 | GJA4 | 243 |
| 11 | 1865240 | 1866108 | LSP1 | 243 |
| 15 | 91808931 | 91809448 | SV2B | 243 |
| 1 | 181104684 | 181104734 | IER5 | 243 |
| 1 | 9300676 | 9300796 | H6PD | 243 |
| 2 | 61306771 | 61307799 | KIAA1841 | 243 |
| 16 | 48571335 | 48571689 | N4BP1 | 243 |
| 13 | 86082705 | 86083540 | SLITRK6 | 243 |
| 6 | 170178768 | 170179300 | C6orf70 | 243 |
| 1 | 165362095 | 165362581 | LMX1A | 243 |
| 6 | 5243012 | 5243581 | LYRM4 | 242 |
| 1 | 26615327 | 26617857 | UBXN11 | 242 |
| 21 | 40618526 | 40619115 | BRWD1 | 242 |
| 12 | 1837118 | 1837964 | ADIPOR2 | 242 |
| X | 1655842 | 1656301 | P2RY8 | 242 |
| 16 | 82190269 | 82190832 | MPHOSPH6 | 242 |
| 13 | 45914479 | 45915159 | TPT1 | 242 |
| 17 | 17306611 | 17307995 | SMCR9 | 241 |
| 3 | 125977170 | 125977737 | ALDH1L1 | 241 |
| 7 | 2710208 | 2711211 | AMZ1 | 241 |
| 1 | 32529701 | 32531493 | TMEM39B | 241 |
| 20 | 2812650 | 2813352 | VPS16 | 241 |
| 10 | 116303601 | 116304507 | ABLIM1 | 241 |
| 7 | 151094150 | 151095200 | WDR86 | 240 |
| 13 | 48072746 | 48073125 | SUCLA2 | 240 |
| 16 | 11402248 | 11402414 | PRM1 | 240 |
| 13 | 37071969 | 37072460 | CCNA1 | 240 |
| 1 | 174968298 | 174968996 | CACYBP | 240 |
| 7 | 152160955 | 152162468 | MLL3 | 240 |
| 7 | 158504926 | 158505760 | NCAPG2 | 240 |
| 8 | 110603546 | 110604297 | SYBU | 240 |
| 10 | 97889375 | 97890545 | ZNF518A | 240 |
| 3 | 162475798 | 162476291 | OTOL1 | 240 |
| 11 | 75597840 | 75598872 | UVRAG | 240 |
| 15 | 101582777 | 101583385 | LRRK1 | 240 |
| 1 | 145381257 | 145382111 | HFE2 | 240 |
| 15 | 50731416 | 50732050 | USP8 | 240 |
| 6 | 145486621 | 145487273 | EPM2A | 240 |
| 7 | 68008865 | 68009200 | AUTS2 | 239 |
| 20 | 4792108 | 4792917 | RASSF2 | 239 |
| 2 | 86757061 | 86757480 | CHMP3 | 239 |
| 2 | 86757061 | 86757480 | RNF103-CHMP3 | 239 |
| 4 | 15889223 | 15889993 | FGFBP1 | 239 |
| 20 | 22077465 | 22078066 | PAX1 | 239 |
| 9 | 140223776 | 140224341 | EXD3 | 239 |
| 1 | 753066 | 753620 | SAMD11 | 239 |
| 7 | 47344169 | 47345014 | TNS3 | 239 |
| 9 | 34118533 | 34118597 | DCAF12 | 239 |
| 13 | 86081278 | 86082661 | SLITRK6 | 239 |
| 4 | 6888923 | 6890719 | TBC1D14 | 239 |
| 17 | 46217006 | 46217072 | SKAP1 | 238 |
| 22 | 47018399 | 47018866 | GRAMD4 | 238 |
| 3 | 159789263 | 159790332 | IL12A | 238 |
| 19 | 34708510 | 34709291 | LSM14A | 238 |
| 6 | 152431846 | 152432863 | ESR1 | 238 |
| 7 | 73506991 | 73508015 | LIMK1 | 238 |
| 8 | 81376416 | 81376942 | ZBTB10 | 238 |
| 4 | 6167788 | 6168392 | JAKMIP1 | 238 |
| 3 | 17548086 | 17548640 | TBC1D5 | 237 |
| 11 | 1884542 | 1885314 | LSP1 | 237 |
| 6 | 135504381 | 135504998 | MYB | 237 |
| 7 | 13105137 | 13105864 | ARL4A | 237 |
| 10 | 80891642 | 80892860 | ZMIZ1 | 237 |
| 10 | 122790901 | 122791555 | WDR11 | 237 |
| 2 | 68479403 | 68480594 | PPP3R1 | 237 |
| 4 | 6925948 | 6928144 | TBC1D14 | 237 |
| 6 | 26026862 | 26027585 | HIST1H4B | 237 |
| 2 | 96955625 | 96956152 | SNRNP200 | 237 |
| 15 | 79053323 | 79054560 | ADAMTS7 | 237 |
| 1 | 54561856 | 54562327 | TCEANC2 | 237 |
| 6 | 25033019 | 25033562 | FAM65B | 237 |
| 15 | 40625878 | 40627178 | C15orf52 | 237 |
| 7 | 11201586 | 11202101 | PHF14 | 236 |
| 2 | 43353860 | 43355640 | ZFP36L2 | 236 |
| 15 | 77296220 | 77297248 | PSTPIP1 | 236 |
| 6 | 37143529 | 37144455 | PIM1 | 236 |
| 3 | 196389871 | 196394568 | LRRC33 | 236 |
| 15 | 90607709 | 90609083 | ZNF710 | 236 |
| 6 | 149703285 | 149703806 | SUMO4 | 236 |
| 6 | 149703285 | 149703806 | TAB2 | 236 |
| 11 | 120079373 | 120080361 | OAF | 236 |
| 2 | 47283920 | 47285147 | TTC7A | 236 |
| 15 | 57590891 | 57592902 | CGNL1 | 236 |
| 1 | 41326066 | 41327269 | CITED4 | 235 |
| 1 | 33298552 | 33299375 | S100PBP | 235 |
| 1 | 33298552 | 33299375 | YARS | 235 |
| 6 | 159980874 | 159981553 | SOD2 | 235 |
| 12 | 10001815 | 10002759 | CLEC2B | 235 |
| 9 | 140365218 | 140365611 | PNPLA7 | 235 |
| 13 | 31191248 | 31192479 | USPL1 | 235 |
| 4 | 3306846 | 3308117 | RGS12 | 235 |
| 1 | 45179580 | 45180630 | C1orf228 | 235 |
| 11 | 10566233 | 10567021 | RNF141 | 235 |
| 2 | 219367880 | 219368562 | USP37 | 235 |
| 10 | 111613820 | 111614340 | XPNPEP1 | 235 |
| 16 | 86415539 | 86416857 | FOXF1 | 234 |
| 10 | 98415472 | 98415920 | PIK3AP1 | 234 |
| 11 | 74972347 | 74973440 | ARRB1 | 234 |
| 7 | 19443544 | 19444332 | FERD3L | 234 |
| 2 | 169927102 | 169927657 | DHRS9 | 234 |
| 17 | 70715176 | 70716730 | SLC39A11 | 234 |
| 19 | 17831650 | 17833486 | MAP1S | 234 |
| 6 | 119205943 | 119206466 | MCM9 | 234 |
| 6 | 119205943 | 119206466 | ASF1A | 234 |
| 17 | 27798206 | 27798694 | TAOK1 | 234 |
| 5 | 32310836 | 32311487 | MTMR12 | 234 |
| 16 | 4323198 | 4324426 | TFAP4 | 234 |
| 7 | 155179223 | 155180374 | EN2 | 234 |
| 6 | 26570162 | 26572023 | ABT1 | 233 |
| 10 | 94458180 | 94458957 | HHEX | 233 |
| 8 | 6034119 | 6034878 | MCPH1 | 233 |
| 1 | 12492678 | 12493605 | VPS13D | 233 |
| 10 | 35838143 | 35838929 | CCNY | 233 |
| 11 | 72839806 | 72841387 | FCHSD2 | 233 |
| X | 40012053 | 40013167 | BCOR | 233 |
| 17 | 60951466 | 60951817 | MARCH10 | 233 |
| 14 | 107034395 | 107035031 | TMEM121 | 233 |
| 14 | 101367188 | 101367774 | RTL1 | 233 |
| 13 | 114895955 | 114897075 | RASA3 | 233 |
| 3 | 32187357 | 32187804 | GPD1L | 233 |
| 19 | 42700558 | 42701275 | ZNF526 | 233 |
| 1 | 26946623 | 26948201 | RPS6KA1 | 233 |
| 6 | 3813266 | 3813744 | FAM50B | 232 |
| 11 | 93861365 | 93862887 | PANX1 | 232 |
| 1 | 226830584 | 226831632 | ITPKB | 232 |
| 4 | 46623270 | 46623755 | GABRA2 | 232 |
| 9 | 19379644 | 19380970 | RPS6 | 232 |
| 1 | 110948958 | 110949545 | HBXIP | 232 |
| 11 | 9268364 | 9268849 | DENND5A | 232 |
| 6 | 12010858 | 12011677 | HIVEP1 | 231 |
| 16 | 80546655 | 80547049 | DYNLRB2 | 231 |
| 4 | 140340010 | 140340525 | RAB33B | 231 |
| 10 | 116219848 | 116220782 | ABLIM1 | 231 |
| 1 | 109481800 | 109482410 | CLCC1 | 231 |
| 16 | 11516890 | 11517294 | RMI2 | 231 |
| 10 | 81045467 | 81047194 | ZMIZ1 | 231 |
| 19 | 54710945 | 54711883 | RPS9 | 231 |
| 10 | 94439578 | 94440382 | HHEX | 231 |
| 14 | 61220835 | 61221273 | MNAT1 | 231 |
| 18 | 13426791 | 13427366 | C18orf1 | 230 |
| 6 | 28180167 | 28180826 | ZNF193 | 230 |
| 5 | 116603942 | 116604448 | SEMA6A | 230 |
| 7 | 45979292 | 45979622 | IGFBP3 | 230 |
| 13 | 91743261 | 91744184 | GPC5 | 230 |
| 6 | 82157950 | 82158556 | FAM46A | 230 |
| 1 | 100652863 | 100653334 | DBT | 230 |
| 6 | 24441049 | 24441502 | GPLD1 | 230 |
| 17 | 80544542 | 80545950 | FOXK2 | 230 |
| 4 | 185735011 | 185735560 | ACSL1 | 230 |
| 1 | 207378038 | 207378631 | C4BPA | 230 |
| 2 | 43446362 | 43447596 | ZFP36L2 | 230 |
| 14 | 65510871 | 65511646 | FNTB | 229 |
| 14 | 65510871 | 65511646 | MAX | 229 |
| 14 | 65510871 | 65511646 | CHURC1-FNTB | 229 |
| 2 | 102844175 | 102844639 | IL1RL2 | 229 |
| 4 | 88343177 | 88344277 | NUDT9 | 229 |
| X | 119378430 | 119379405 | ZBTB33 | 229 |
| 8 | 144286262 | 144287313 | GPIHBP1 | 229 |
| 14 | 68397326 | 68398114 | RAD51B | 229 |
| 8 | 11882698 | 11883205 | DEFB134 | 229 |
| 10 | 14281104 | 14281743 | FRMD4A | 229 |
| 7 | 106300019 | 106300925 | C7orf74 | 229 |
| 3 | 56706621 | 56707392 | FAM208A | 229 |
| 1 | 54409757 | 54411567 | LRRC42 | 229 |
| 20 | 1098931 | 1099866 | PSMF1 | 228 |
| 8 | 11719214 | 11719829 | CTSB | 228 |
| 8 | 59896682 | 59897470 | TOX | 228 |
| 3 | 141515737 | 141517587 | GRK7 | 228 |
| 13 | 85720983 | 85721717 | SLITRK6 | 228 |
| 3 | 196415556 | 196417026 | PIGX | 228 |
| 10 | 76407968 | 76408709 | ADK | 228 |
| 12 | 89433594 | 89434214 | DUSP6 | 228 |
| 19 | 28325170 | 28325549 | UQCRFS1 | 228 |
| 17 | 55944855 | 55944990 | CUEDC1 | 228 |
| 6 | 26283754 | 26286180 | HIST1H3G | 228 |
| 19 | 35939645 | 35940761 | FFAR2 | 228 |
| 15 | 44068998 | 44070377 | SERF2 | 228 |
| 15 | 44068998 | 44070377 | ELL3 | 228 |
| 2 | 3224592 | 3225493 | TSSC1 | 228 |
| 15 | 44068998 | 44070377 | PDIA3 | 228 |
| 10 | 27046506 | 27047100 | ABI1 | 228 |
| 19 | 35529533 | 35530843 | HPN | 227 |
| 6 | 138167755 | 138168365 | TNFAIP3 | 227 |
| 1 | 51985066 | 51985334 | EPS15 | 227 |
| 4 | 45430267 | 45430644 | GABRG1 | 227 |
| 16 | 30469743 | 30470770 | DCTPP1 | 227 |
| 3 | 150445518 | 150446401 | FAM194A | 227 |
| 17 | 49011815 | 49013397 | TOB1 | 227 |
| 20 | 29617594 | 29619188 | DEFB115 | 227 |
| 2 | 37624923 | 37626056 | QPCT | 227 |
| 9 | 69031326 | 69032090 | FOXD4L6 | 227 |
| 1 | 145399973 | 145400057 | HFE2 | 227 |
| 22 | 25796250 | 25797602 | LRP5L | 227 |
| 5 | 103020642 | 103021147 | NUDT12 | 227 |
| 12 | 123849507 | 123850298 | SETD8 | 227 |
| 6 | 157824837 | 157825217 | ZDHHC14 | 227 |
| 17 | 43200997 | 43202010 | PLCD3 | 227 |
| 3 | 31310574 | 31311144 | STT3B | 227 |
| 2 | 182321177 | 182322632 | ITGA4 | 227 |
| 8 | 22562007 | 22562968 | EGR3 | 227 |
| 9 | 140430200 | 140431327 | PNPLA7 | 226 |
| 11 | 12088145 | 12088874 | MICAL2 | 226 |
| 6 | 27719597 | 27721077 | HIST1H2BL | 226 |
| 18 | 60629404 | 60630027 | PHLPP1 | 226 |
| 15 | 75221860 | 75223125 | COX5A | 226 |
| 6 | 23019439 | 23020230 | HDGFL1 | 226 |
| 4 | 43929110 | 43929746 | KCTD8 | 226 |
| 15 | 90602227 | 90603278 | ZNF710 | 226 |
| 10 | 47333057 | 47333670 | AGAP9 | 226 |
| 6 | 99968603 | 99969471 | LOC100130890 | 226 |
| 13 | 52768192 | 52769145 | NEK3 | 226 |
| 16 | 68117995 | 68119016 | NFATC3 | 226 |
| 17 | 72565404 | 72566501 | CD300C | 226 |
| 2 | 240174047 | 240175085 | HDAC4 | 226 |
| 15 | 78384637 | 78385142 | SH2D7 | 226 |
| 5 | 158381702 | 158381832 | EBF1 | 226 |
| 19 | 7159078 | 7159290 | INSR | 226 |
| 20 | 4793276 | 4796052 | RASSF2 | 226 |
| 19 | 54712114 | 54712932 | MBOAT7 | 226 |
| 11 | 14913171 | 14914141 | CYP2R1 | 226 |
| 3 | 170178719 | 170179161 | SLC7A14 | 225 |
| 16 | 85587887 | 85588420 | KIAA0182 | 225 |
| 17 | 78315439 | 78316216 | RNF213 | 225 |
| 4 | 40005868 | 40006770 | PDS5A | 225 |
| 1 | 158921540 | 158922180 | PYHIN1 | 225 |
| 17 | 75400472 | 75400477 | SEPT9 | 225 |
| 5 | 131380954 | 131381351 | CSF2 | 225 |
| 6 | 109010293 | 109011058 | FOXO3 | 225 |
| 10 | 101946506 | 101947030 | ERLIN1 | 225 |
| 14 | 81686717 | 81687934 | GTF2A1 | 225 |
| 5 | 13730652 | 13731419 | DNAH5 | 225 |
| 1 | 114264226 | 114265445 | PHTF1 | 225 |
| 11 | 124940382 | 124941212 | SLC37A2 | 225 |
| 19 | 6800267 | 6802117 | VAV1 | 225 |
| 4 | 47559678 | 47560221 | ATP10D | 225 |
| 3 | 196682170 | 196683135 | PIGZ | 225 |
| 11 | 62657641 | 62658605 | SLC3A2 | 225 |
| 3 | 185040861 | 185042238 | MAP3K13 | 225 |
| 14 | 65510206 | 65510650 | FNTB | 224 |
| 14 | 65510206 | 65510650 | MAX | 224 |
| 14 | 65510206 | 65510650 | CHURC1-FNTB | 224 |
| 8 | 14567406 | 14568063 | SGCZ | 224 |
| 3 | 183863649 | 183864702 | AP2M1 | 224 |
| 10 | 116608365 | 116608901 | FAM160B1 | 224 |
| 1 | 19831884 | 19832395 | CAPZB | 224 |
| 21 | 34726129 | 34726734 | IFNAR1 | 224 |
| 8 | 108653234 | 108653847 | ANGPT1 | 224 |
| 4 | 3565901 | 3567343 | LRPAP1 | 224 |
| X | 17756161 | 17756765 | SCML1 | 224 |
| 12 | 109231992 | 109232663 | SSH1 | 224 |
| 17 | 6984497 | 6985262 | CLEC10A | 224 |
| 8 | 68661733 | 68662381 | CPA6 | 223 |
| 16 | 33237074 | 33241352 | TP53TG3 | 223 |
| 16 | 33237074 | 33241352 | TP53TG3B | 223 |
| 21 | 43886897 | 43886990 | RSPH1 | 223 |
| 6 | 27774278 | 27776588 | HIST1H2AI | 223 |
| 5 | 143660132 | 143660827 | KCTD16 | 223 |
| 14 | 72726168 | 72726805 | RGS6 | 223 |
| 3 | 162140255 | 162140794 | OTOL1 | 223 |
| 3 | 49591432 | 49591489 | BSN | 223 |
| 21 | 46208754 | 46209506 | UBE2G2 | 223 |
| 22 | 46894544 | 46895095 | CELSR1 | 223 |
| 1 | 246945163 | 246946282 | SCCPDH | 223 |
| 10 | 94901198 | 94901759 | CYP26A1 | 223 |
| 7 | 40571065 | 40571689 | C7orf10 | 223 |
| 8 | 20896671 | 20898176 | GFRA2 | 223 |
| 1 | 67425479 | 67425884 | MIER1 | 222 |
| 13 | 91055320 | 91056437 | GPC5 | 222 |
| 21 | 11089136 | 11090600 | BAGE5 | 222 |
| 21 | 11089136 | 11090600 | BAGE | 222 |
| 11 | 121247902 | 121248382 | SORL1 | 222 |
| 11 | 60837501 | 60838054 | CD5 | 222 |
| 19 | 14518822 | 14521294 | DDX39A | 222 |
| 6 | 111302440 | 111303617 | RPF2 | 222 |
| 6 | 141569627 | 141570276 | NMBR | 222 |
| 1 | 145382228 | 145382730 | HFE2 | 222 |
| 19 | 21356279 | 21356838 | ZNF431 | 222 |
| 3 | 182741498 | 182742212 | MCCC1 | 222 |
| 18 | 43923589 | 43924377 | RNF165 | 222 |
| 18 | 9803721 | 9804332 | RAB31 | 222 |
| 11 | 119445002 | 119445340 | THY1 | 222 |
| 11 | 118798506 | 118799293 | UPK2 | 222 |
| 13 | 78594046 | 78594779 | EDNRB | 221 |
| X | 39753231 | 39753680 | BCOR | 221 |
| 5 | 118884282 | 118884818 | FAM170A | 221 |
| 14 | 55737703 | 55738250 | FBXO34 | 221 |
| 4 | 37003340 | 37004051 | KIAA1239 | 221 |
| 1 | 3499559 | 3500568 | MEGF6 | 221 |
| 20 | 61201079 | 61201638 | C20orf166 | 221 |
| 3 | 187387450 | 187388037 | SST | 221 |
| 7 | 1155110 | 1157218 | C7orf50 | 221 |
| 6 | 26322115 | 26323140 | HIST1H4H | 221 |
| 16 | 83847847 | 83848478 | HSBP1 | 221 |
| 13 | 114899602 | 114900443 | RASA3 | 221 |
| 9 | 139432702 | 139434862 | NOTCH1 | 221 |
| 2 | 181978351 | 181978739 | UBE2E3 | 221 |
| 8 | 14584844 | 14585486 | SGCZ | 221 |
| 19 | 24216140 | 24217062 | ZNF254 | 220 |
| 6 | 102207584 | 102208182 | GRIK2 | 220 |
| 10 | 64290887 | 64291425 | ZNF365 | 220 |
| 1 | 78148884 | 78149504 | ZZZ3 | 220 |
| 9 | 130281138 | 130282220 | FAM129B | 220 |
| 2 | 24368443 | 24368823 | LOC375190 | 220 |
| 18 | 13614651 | 13615309 | C18orf1 | 220 |
| 21 | 24594661 | 24595141 | NCAM2 | 220 |
| 6 | 14274966 | 14276141 | CD83 | 220 |
| 4 | 47869877 | 47870523 | NFXL1 | 220 |
| 5 | 180230127 | 180231467 | MGAT1 | 220 |
| 12 | 7066977 | 7069005 | PTPN6 | 219 |
| 6 | 84569102 | 84569756 | CYB5R4 | 219 |
| 7 | 7899962 | 7900486 | GLCCI1 | 219 |
| 14 | 21945149 | 21945545 | RAB2B | 219 |
| 6 | 37137982 | 37139574 | PIM1 | 219 |
| 5 | 24563494 | 24564018 | CDH10 | 219 |
| 2 | 63493346 | 63493990 | WDPCP | 219 |
| 1 | 10571949 | 10572088 | PEX14 | 219 |
| 6 | 135502804 | 135504231 | MYB | 219 |
| 3 | 150125523 | 150128985 | TSC22D2 | 219 |
| 5 | 10735869 | 10737254 | DAP | 219 |
| 10 | 95053949 | 95054697 | MYOF | 219 |
| 17 | 27709911 | 27710472 | TAOK1 | 219 |
| 7 | 128405694 | 128406262 | CALU | 219 |
| 3 | 196764462 | 196765069 | MFI2 | 219 |
| 1 | 94057229 | 94058173 | BCAR3 | 219 |
| 8 | 145746819 | 145748521 | LRRC14 | 219 |
| 8 | 145746819 | 145748521 | LRRC24 | 219 |
| 2 | 82641081 | 82641603 | SUCLG1 | 219 |
| 17 | 67369850 | 67371494 | MAP2K6 | 219 |
| 2 | 87893983 | 87895296 | PLGLB2 | 219 |
| 2 | 241504701 | 241505891 | CAPN10 | 218 |
| 13 | 79214373 | 79214998 | RNF219 | 218 |
| 10 | 111916193 | 111917121 | MXI1 | 218 |
| 5 | 131518929 | 131519537 | P4HA2 | 218 |
| 13 | 48573350 | 48573905 | SUCLA2 | 218 |
| 21 | 25186012 | 25186693 | MRPL39 | 218 |
| 3 | 151670251 | 151670917 | SUCNR1 | 218 |
| 18 | 74768282 | 74769080 | MBP | 218 |
| 2 | 85977740 | 85978355 | ATOH8 | 218 |
| 13 | 106099450 | 106099875 | DAOA | 218 |
| 10 | 80886436 | 80886684 | ZMIZ1 | 218 |
| 3 | 14186220 | 14186701 | CHCHD4 | 218 |
| 17 | 72457595 | 72459886 | CD300A | 218 |
| 9 | 20691394 | 20692026 | KIAA1797 | 218 |
| 10 | 45135690 | 45136247 | CXCL12 | 218 |
| 3 | 118291057 | 118291354 | IGSF11 | 218 |
| 18 | 53498677 | 53499348 | TCF4 | 217 |
| 5 | 176300297 | 176300407 | UNC5A | 217 |
| 11 | 62606280 | 62608883 | WDR74 | 217 |
| 21 | 45507270 | 45508186 | TRAPPC10 | 217 |
| 21 | 34772792 | 34773971 | IFNGR2 | 217 |
| 7 | 150118119 | 150119328 | GIMAP8 | 217 |
| 7 | 18976375 | 18977008 | HDAC9 | 217 |
| 9 | 47169035 | 47169257 | CNTNAP3B | 217 |
| 5 | 145656984 | 145657443 | RBM27 | 217 |
| 6 | 21567189 | 21567899 | SOX4 | 217 |
| 12 | 117500595 | 117501854 | TESC | 217 |
| 10 | 29433543 | 29434101 | LYZL1 | 217 |
| 18 | 13649188 | 13650054 | C18orf1 | 217 |
| 6 | 26033265 | 26034194 | HIST1H3B | 217 |
| 7 | 2684406 | 2685680 | TTYH3 | 217 |
| 20 | 56078770 | 56079047 | CTCFL | 217 |
| 4 | 2895958 | 2896888 | ADD1 | 217 |
| 13 | 42613750 | 42613811 | DGKH | 217 |
| 5 | 51118536 | 51119134 | ISL1 | 217 |
| 7 | 6211908 | 6213257 | CYTH3 | 216 |
| 11 | 9381949 | 9383005 | IPO7 | 216 |
| 2 | 62382663 | 62383664 | B3GNT2 | 216 |
| 5 | 176734131 | 176735177 | MXD3 | 216 |
| 1 | 4045101 | 4045227 | C1orf174 | 216 |
| 1 | 148596700 | 148597381 | NBPF16 | 216 |
| 1 | 148596700 | 148597381 | NBPF15 | 216 |
| 3 | 88114742 | 88115845 | CGGBP1 | 216 |
| 1 | 221052854 | 221053253 | HLX | 215 |
| 17 | 39723312 | 39724066 | KRT9 | 215 |
| 2 | 55329581 | 55330170 | RTN4 | 215 |
| 4 | 25089775 | 25090797 | LGI2 | 215 |
| 8 | 86300370 | 86300454 | CA1 | 215 |
| 19 | 35453800 | 35454058 | ZNF792 | 215 |
| 18 | 46422782 | 46424149 | SMAD7 | 215 |
| 12 | 44356032 | 44356940 | TMEM117 | 215 |
| 7 | 18517410 | 18518126 | HDAC9 | 215 |
| 8 | 108524301 | 108524840 | ANGPT1 | 215 |
| 2 | 33636160 | 33636907 | RASGRP3 | 215 |
| 8 | 17300359 | 17300975 | MTMR7 | 215 |
| 22 | 36889446 | 36889919 | FOXRED2 | 215 |
| 19 | 8566424 | 8567188 | PRAM1 | 215 |
| 10 | 33268649 | 33270216 | ITGB1 | 215 |
| 19 | 16393977 | 16395923 | KLF2 | 215 |
| 3 | 151506537 | 151507130 | AADAC | 215 |
| 3 | 119795076 | 119795771 | GSK3B | 215 |
| 19 | 4568356 | 4569547 | LRG1 | 215 |
| 15 | 101689801 | 101691364 | CHSY1 | 215 |
| 3 | 195959945 | 195961377 | ZDHHC19 | 215 |
| 3 | 11684965 | 11685633 | VGLL4 | 215 |
| 7 | 5369782 | 5370706 | TNRC18 | 215 |
| 9 | 130469092 | 130469592 | C9orf117 | 215 |
| 12 | 48171481 | 48172358 | SLC48A1 | 214 |
| 12 | 48171481 | 48172358 | HDAC7 | 214 |
| 1 | 234859779 | 234861106 | IRF2BP2 | 214 |
| 8 | 7394194 | 7395308 | FAM90A7 | 214 |
| 5 | 95194395 | 95194980 | GLRX | 214 |
| 14 | 102965966 | 102966995 | TECPR2 | 214 |
| 18 | 65885054 | 65885613 | TMX3 | 214 |
| 5 | 65225856 | 65226506 | ERBB2IP | 214 |
| 2 | 178089266 | 178089741 | HNRNPA3 | 214 |
| 8 | 33411239 | 33411534 | RNF122 | 214 |
| 7 | 18593689 | 18594285 | HDAC9 | 214 |
| 19 | 54715056 | 54715852 | MBOAT7 | 214 |
| 6 | 3045506 | 3046126 | RIPK1 | 214 |
| 8 | 101732686 | 101733598 | PABPC1 | 214 |
| 10 | 73508321 | 73511060 | C10orf54 | 214 |
| 10 | 73508321 | 73511060 | CDH23 | 214 |
| 17 | 67187992 | 67188509 | ABCA10 | 214 |
| 7 | 142143105 | 142144531 | PRSS58 | 214 |
| 10 | 81004649 | 81005372 | ZMIZ1 | 214 |
| 8 | 142011987 | 142012338 | PTK2 | 214 |
| 4 | 29419442 | 29419869 | PCDH7 | 214 |
| 7 | 25934546 | 25935536 | NFE2L3 | 214 |
| 2 | 137180776 | 137180836 | CXCR4 | 214 |
| 9 | 92444374 | 92444904 | GADD45G | 214 |
| 1 | 235805118 | 235805976 | GNG4 | 214 |
| 14 | 104125022 | 104125738 | KLC1 | 214 |
| 18 | 67872536 | 67873426 | RTTN | 214 |
| 14 | 35024344 | 35025119 | EAPP | 214 |
| 13 | 114814493 | 114815174 | RASA3 | 214 |
| 3 | 157646616 | 157647228 | SHOX2 | 214 |
| 15 | 48483341 | 48483575 | CTXN2 | 213 |
| 2 | 160390928 | 160391557 | BAZ2B | 213 |
| 20 | 10413715 | 10415094 | MKKS | 213 |
| 3 | 32950625 | 32950819 | CCR4 | 213 |
| 7 | 65680037 | 65681144 | TPST1 | 213 |
| 4 | 55888625 | 55889173 | KDR | 213 |
| 2 | 28542253 | 28542901 | BRE | 213 |
| 18 | 59854528 | 59855275 | PIGN | 213 |
| 14 | 99645360 | 99645845 | BCL11B | 213 |
| 12 | 45228127 | 45229720 | NELL2 | 213 |
| 3 | 101231809 | 101232293 | SENP7 | 213 |
| 12 | 131246364 | 131247031 | STX2 | 213 |
| 12 | 45286218 | 45286954 | NELL2 | 213 |
| 10 | 80712416 | 80713415 | ZMIZ1 | 213 |
| 3 | 88198331 | 88199726 | C3orf38 | 213 |
| 3 | 88198331 | 88199726 | CGGBP1 | 213 |
| 12 | 27396638 | 27397846 | STK38L | 212 |
| 8 | 7883740 | 7888244 | LOC100132396 | 212 |
| 1 | 226187088 | 226187688 | C1orf55 | 212 |
| 3 | 102235626 | 102236188 | ZPLD1 | 212 |
| 2 | 65384400 | 65385223 | RAB1A | 212 |
| 12 | 46089573 | 46091776 | ARID2 | 212 |
| 14 | 77498843 | 77500923 | IRF2BPL | 212 |
| 6 | 27798294 | 27799498 | HIST1H2BL | 212 |
| 5 | 55353135 | 55354010 | IL6ST | 212 |
| 7 | 2439550 | 2443096 | CHST12 | 212 |
| 19 | 8851427 | 8852436 | OR2Z1 | 212 |
| 14 | 101158408 | 101159984 | DLK1 | 212 |
| 6 | 3337881 | 3338085 | SLC22A23 | 212 |
| 10 | 73563294 | 73563402 | CDH23 | 212 |
| 17 | 73340996 | 73341506 | GRB2 | 211 |
| 7 | 25559112 | 25559606 | NPVF | 211 |
| 8 | 6484119 | 6486255 | MCPH1 | 211 |
| 9 | 94660784 | 94661604 | ROR2 | 211 |
| 16 | 85862659 | 85864435 | COX4I1 | 211 |
| 3 | 183723585 | 183724074 | ABCC5 | 211 |
| 7 | 21945772 | 21946179 | CDCA7L | 211 |
| 7 | 21945772 | 21946179 | DNAH11 | 211 |
| 13 | 85594647 | 85595662 | SLITRK6 | 211 |
| 20 | 49434650 | 49435396 | BCAS4 | 211 |
| 11 | 36734768 | 36735018 | RAG2 | 211 |
| 10 | 120450878 | 120451461 | C10orf46 | 211 |
| 3 | 167176593 | 167177202 | SERPINI2 | 211 |
| 10 | 49288286 | 49288791 | FAM25B | 211 |
| 21 | 36398621 | 36399617 | RUNX1 | 211 |
| 17 | 56649614 | 56650187 | TEX14 | 211 |
| 3 | 147288598 | 147289301 | ZIC1 | 211 |
| 6 | 79811625 | 79812166 | PHIP | 211 |
| 10 | 129845133 | 129846190 | PTPRE | 210 |
| 2 | 69513676 | 69513909 | GFPT1 | 210 |
| 14 | 24911503 | 24912193 | KHNYN | 210 |
| 14 | 24911503 | 24912193 | SDR39U1 | 210 |
| 18 | 9396596 | 9397126 | TWSG1 | 210 |
| 6 | 106533817 | 106534666 | PRDM1 | 210 |
| 2 | 26138542 | 26138987 | ASXL2 | 210 |
| 13 | 100153208 | 100154180 | TM9SF2 | 210 |
| 3 | 148799679 | 148800335 | HLTF | 210 |
| 1 | 31481593 | 31482313 | PUM1 | 210 |
| 3 | 188665841 | 188666414 | TPRG1 | 210 |
| 15 | 99826067 | 99826754 | LRRC28 | 210 |
| X | 128910942 | 128911775 | SASH3 | 210 |
| 4 | 87927657 | 87928759 | AFF1 | 209 |
| 5 | 180869121 | 180869141 | OR4F29 | 209 |
| 10 | 35479388 | 35480007 | CREM | 209 |
| 17 | 19091342 | 19093114 | EPN2 | 209 |
| 3 | 39537369 | 39538148 | MOBP | 209 |
| 21 | 43885817 | 43886411 | RSPH1 | 209 |
| 3 | 125977953 | 125978722 | ALDH1L1 | 209 |
| 6 | 87901155 | 87901645 | ZNF292 | 209 |
| 2 | 135810231 | 135810783 | RAB3GAP1 | 209 |
| 10 | 121065923 | 121066452 | GRK5 | 209 |
| 13 | 74477525 | 74478019 | KLF12 | 209 |
| 11 | 118395850 | 118396475 | MLL | 209 |
| 18 | 74874183 | 74874880 | MBP | 209 |
| 21 | 46529304 | 46529557 | ADARB1 | 209 |
| 2 | 101863501 | 101864045 | C2orf29 | 209 |
| 15 | 41127029 | 41127704 | DNAJC17 | 209 |
| 2 | 241505982 | 241506014 | CAPN10 | 209 |
| 11 | 8279725 | 8280373 | LMO1 | 208 |
| 1 | 45187895 | 45188842 | C1orf228 | 208 |
| 2 | 82953715 | 82954323 | SUCLG1 | 208 |
| 6 | 87321338 | 87321859 | HTR1E | 208 |
| 5 | 90142945 | 90143022 | GPR98 | 208 |
| 3 | 29462153 | 29462682 | RBMS3 | 208 |
| 19 | 44066602 | 44067748 | XRCC1 | 208 |
| 4 | 26585268 | 26586259 | TBC1D19 | 208 |
| 17 | 78864787 | 78866525 | RPTOR | 208 |
| 8 | 104431081 | 104431509 | SLC25A32 | 208 |
| 8 | 104431081 | 104431509 | DCAF13 | 208 |
| 10 | 71097302 | 71097772 | HK1 | 208 |
| 3 | 192498642 | 192499421 | FGF12 | 208 |
| 8 | 86545669 | 86546215 | REXO1L1 | 208 |
| 17 | 34528566 | 34529287 | TBC1D3B | 208 |
| 2 | 153467889 | 153467970 | FMNL2 | 208 |
| 10 | 28863020 | 28863657 | WAC | 207 |
| 15 | 41456746 | 41457388 | INO80 | 207 |
| 18 | 32557603 | 32557679 | MAPRE2 | 207 |
| 3 | 182043879 | 182044281 | ATP11B | 207 |
| 6 | 37014615 | 37016280 | FGD2 | 207 |
| 6 | 68828582 | 68829023 | BAI3 | 207 |
| 11 | 76067570 | 76068201 | PRKRIR | 207 |
| 11 | 134453584 | 134455288 | B3GAT1 | 207 |
| 7 | 74142136 | 74142728 | GTF2I | 207 |
| 1 | 26186368 | 26187281 | C1orf135 | 207 |
| 10 | 22117943 | 22118680 | DNAJC1 | 207 |
| 6 | 134617831 | 134617884 | SGK1 | 207 |
| 1 | 152020187 | 152020986 | S100A11 | 207 |
| 8 | 101300744 | 101301434 | RNF19A | 207 |
| 2 | 61107955 | 61108488 | REL | 207 |
| 13 | 85534888 | 85537095 | SLITRK6 | 207 |
| 2 | 41384940 | 41385460 | SLC8A1 | 207 |
| 5 | 100072504 | 100073202 | ST8SIA4 | 207 |
| 1 | 8694662 | 8695209 | RERE | 207 |
| 3 | 57193433 | 57194047 | IL17RD | 207 |
| X | 55026106 | 55026977 | APEX2 | 207 |
| 10 | 50814863 | 50815647 | SLC18A3 | 207 |
| 10 | 50814863 | 50815647 | CHAT | 207 |
| 13 | 80942218 | 80943056 | SPRY2 | 206 |
| 6 | 26569064 | 26569775 | ABT1 | 206 |
| 5 | 990144 | 990660 | LOC100506688 | 206 |
| 5 | 95170183 | 95171235 | GLRX | 206 |
| 15 | 32828679 | 32829378 | ARHGAP11A | 206 |
| 2 | 239977143 | 239978316 | HDAC4 | 206 |
| 3 | 152783510 | 152784075 | RAP2B | 206 |
| 17 | 61523844 | 61525429 | CYB561 | 206 |
| 6 | 37099850 | 37100773 | PIM1 | 206 |
| 17 | 47921121 | 47922310 | TAC4 | 206 |
| 17 | 47921121 | 47922310 | FLJ45513 | 206 |
| 3 | 63796757 | 63797265 | THOC7 | 206 |
| 7 | 6739434 | 6740014 | ZNF12 | 206 |
| 19 | 18588675 | 18591099 | ELL | 206 |
| 15 | 67315985 | 67316599 | SMAD3 | 206 |
| 3 | 195691808 | 195692416 | TNK2 | 206 |
| 1 | 205289185 | 205289222 | NUAK2 | 206 |
| 15 | 49912734 | 49913565 | DTWD1 | 206 |
| 12 | 131464134 | 131465304 | GPR133 | 206 |
| 9 | 113018854 | 113019528 | TXN | 205 |
| 4 | 48369920 | 48370477 | SLAIN2 | 205 |
| 1 | 173544508 | 173545132 | SLC9A11 | 205 |
| 14 | 91317204 | 91317889 | TTC7B | 205 |
| 18 | 59996337 | 59996928 | TNFRSF11A | 205 |
| 13 | 48611521 | 48612671 | NUDT15 | 205 |
| 15 | 55488473 | 55489637 | RSL24D1 | 205 |
| 17 | 19512712 | 19513621 | ALDH3A2 | 205 |
| 3 | 187802621 | 187803076 | LPP | 205 |
| 9 | 126138542 | 126138857 | CRB2 | 205 |
| 3 | 117028484 | 117029200 | LSAMP | 205 |
| 3 | 192733973 | 192734520 | MB21D2 | 205 |
| 2 | 66661075 | 66662635 | MEIS1 | 205 |
| 15 | 32901199 | 32901624 | ARHGAP11A | 205 |
| 16 | 74594620 | 74595300 | GLG1 | 205 |
| 10 | 127373790 | 127374601 | C10orf122 | 205 |
| 17 | 36003133 | 36003825 | DDX52 | 205 |
| 10 | 12061652 | 12062484 | UPF2 | 205 |
| 1 | 246996783 | 246996858 | AHCTF1 | 205 |
| 9 | 69262159 | 69262899 | CBWD6 | 204 |
| 6 | 73329546 | 73331108 | KCNQ5 | 204 |
| 1 | 62738638 | 62738713 | KANK4 | 204 |
| 2 | 30808791 | 30809226 | LCLAT1 | 204 |
| 4 | 46866313 | 46866991 | COX7B2 | 204 |
| 2 | 129491707 | 129492474 | HS6ST1 | 204 |
| 17 | 80200120 | 80203467 | CSNK1D | 204 |
| 17 | 53614270 | 53615004 | MMD | 204 |
| 5 | 172198917 | 172199839 | DUSP1 | 204 |
| 17 | 75400630 | 75402503 | SEPT9 | 204 |
| 3 | 185825011 | 185826781 | ETV5 | 204 |
| 10 | 15379401 | 15379803 | FAM171A1 | 204 |
| 7 | 140532500 | 140533122 | BRAF | 204 |
| 21 | 45897880 | 45899057 | LRRC3 | 204 |
| 18 | 13137446 | 13137885 | C18orf1 | 204 |
| 13 | 81630155 | 81630926 | SPRY2 | 204 |
| 17 | 62153765 | 62154327 | ERN1 | 204 |
| 18 | 36395453 | 36395896 | CELF4 | 204 |
| 2 | 85788415 | 85790701 | GGCX | 204 |
| 2 | 43444654 | 43445619 | ZFP36L2 | 204 |
| 1 | 67323501 | 67324339 | WDR78 | 204 |
| 15 | 64179496 | 64180468 | HERC1 | 203 |
| 2 | 102091563 | 102092347 | RFX8 | 203 |
| 11 | 105948348 | 105948937 | KBTBD3 | 203 |
| 11 | 105948348 | 105948937 | AASDHPPT | 203 |
| 3 | 124547918 | 124548390 | ITGB5 | 203 |
| 5 | 36875275 | 36876811 | NIPBL | 203 |
| 1 | 26095892 | 26097135 | MAN1C1 | 203 |
| 13 | 75875228 | 75876083 | TBC1D4 | 203 |
| 1 | 149338708 | 149339245 | HIST2H2BF | 203 |
| 19 | 45256259 | 45256967 | BCL3 | 203 |
| 3 | 195031728 | 195032718 | ACAP2 | 203 |
| X | 128914187 | 128914428 | SASH3 | 203 |
| 12 | 40605388 | 40606159 | LRRK2 | 203 |
| 6 | 111767368 | 111767956 | REV3L | 203 |
| 10 | 36806179 | 36806614 | ANKRD30A | 203 |
| 12 | 37959386 | 37960666 | ALG10B | 203 |
| 2 | 91654464 | 91655081 | RPIA | 203 |
| 3 | 161194741 | 161195450 | OTOL1 | 202 |
| 5 | 95171318 | 95171875 | GLRX | 202 |
| 3 | 160126449 | 160127386 | SMC4 | 202 |
| 1 | 161476004 | 161476540 | FCGR2A | 202 |
| 6 | 67844890 | 67845449 | EYS | 202 |
| 21 | 25828634 | 25829229 | MRPL39 | 202 |
| 15 | 100014000 | 100015861 | MEF2A | 202 |
| 2 | 98656350 | 98656698 | TMEM131 | 202 |
| 13 | 80119707 | 80120260 | NDFIP2 | 202 |
| 3 | 136144813 | 136146102 | STAG1 | 202 |
| 17 | 55124005 | 55124786 | AKAP1 | 202 |
| 6 | 151695841 | 151696359 | ZBTB2 | 202 |
| 3 | 170105161 | 170105811 | SKIL | 202 |
| 6 | 35384822 | 35386696 | PPARD | 202 |
| 9 | 136988166 | 136988989 | WDR5 | 202 |
| 13 | 114832566 | 114832875 | RASA3 | 202 |
| X | 40025600 | 40026372 | BCOR | 202 |
| 3 | 148829401 | 148830026 | HLTF | 202 |
| 15 | 58601182 | 58601924 | LIPC | 201 |
| 7 | 19151780 | 19152447 | TWIST1 | 201 |
| 20 | 38671197 | 38671730 | MAFB | 201 |
| 7 | 144532727 | 144533768 | TPK1 | 201 |
| 15 | 82578119 | 82578729 | EFTUD1 | 201 |
| 7 | 143125455 | 143126252 | EPHA1 | 201 |
| 6 | 163802183 | 163802695 | QKI | 201 |
| 1 | 12587285 | 12588742 | DHRS3 | 200 |
| 8 | 33412057 | 33412667 | RNF122 | 200 |
| 10 | 12784478 | 12784610 | CAMK1D | 200 |
| 19 | 3132471 | 3134057 | GNA15 | 200 |
| 7 | 5761425 | 5762564 | RNF216 | 200 |
| 4 | 33675001 | 33675710 | ARAP2 | 200 |
| 8 | 27197940 | 27198701 | PTK2B | 200 |
| 2 | 27303705 | 27304441 | EMILIN1 | 200 |
| 8 | 6668612 | 6669758 | XKR5 | 200 |
| 5 | 40797976 | 40799033 | PRKAA1 | 200 |
| 11 | 125948460 | 125949209 | CDON | 200 |
| 3 | 48013970 | 48014747 | MAP4 | 200 |
| 3 | 136276803 | 136277363 | STAG1 | 200 |
| 15 | 93351888 | 93353013 | CHD2 | 199 |
| 16 | 86774068 | 86774614 | FOXL1 | 199 |
| 7 | 22603423 | 22603723 | STEAP1B | 199 |
| 12 | 47608902 | 47609433 | FAM113B | 199 |
| 1 | 243418211 | 243419715 | SDCCAG8 | 199 |
| 14 | 52327106 | 52327762 | GNG2 | 199 |
| 4 | 45340555 | 45341303 | GNPDA2 | 199 |
| 3 | 49517556 | 49518796 | DAG1 | 199 |
| 4 | 1193317 | 1196791 | SPON2 | 199 |
| 12 | 107713872 | 107715061 | BTBD11 | 199 |
| 1 | 117074552 | 117075053 | CD58 | 199 |
| 18 | 56554646 | 56555405 | ZNF532 | 199 |
| 6 | 26198642 | 26200346 | HIST1H2BF | 199 |
| 1 | 234735199 | 234736208 | IRF2BP2 | 199 |
| 4 | 6893597 | 6895687 | TBC1D14 | 199 |
| 5 | 169705599 | 169706384 | LCP2 | 199 |
| 7 | 45016239 | 45017916 | MYO1G | 199 |
| 16 | 87441190 | 87441969 | ZCCHC14 | 199 |
| 17 | 36673808 | 36674464 | SRCIN1 | 199 |
| 2 | 11414778 | 11415457 | ROCK2 | 198 |
| 14 | 100218534 | 100218991 | EML1 | 198 |
| X | 52949565 | 52951320 | FAM156B | 198 |
| 12 | 12878615 | 12879356 | APOLD1 | 198 |
| 12 | 82751802 | 82752604 | C12orf26 | 198 |
| 1 | 100586122 | 100586803 | SASS6 | 198 |
| 15 | 74987946 | 74988815 | EDC3 | 198 |
| 14 | 71451005 | 71451548 | PCNX | 198 |
| 20 | 36618785 | 36619337 | TTI1 | 198 |
| 13 | 114543999 | 114544244 | GAS6 | 198 |
| 14 | 94425512 | 94426462 | ASB2 | 198 |
| 12 | 111115773 | 111116135 | HVCN1 | 198 |
| 7 | 130882170 | 130882229 | MKLN1 | 198 |
| 8 | 106384145 | 106384685 | ZFPM2 | 198 |
| 2 | 166650605 | 166650652 | GALNT3 | 198 |
| 7 | 130882310 | 130882847 | MKLN1 | 197 |
| 20 | 61358750 | 61360339 | NTSR1 | 197 |
| 13 | 114893989 | 114894754 | RASA3 | 197 |
| 6 | 26020506 | 26021515 | HIST1H4A | 197 |
| 14 | 75547107 | 75548220 | MLH3 | 197 |
| 2 | 80067674 | 80068261 | CTNNA2 | 197 |
| 5 | 175122888 | 175124069 | HRH2 | 197 |
| 11 | 47736425 | 47737440 | AGBL2 | 197 |
| 4 | 17628985 | 17629249 | MED28 | 197 |
| 3 | 197440456 | 197441074 | KIAA0226 | 197 |
| 3 | 13526251 | 13527486 | HDAC11 | 197 |
| 9 | 114800480 | 114800609 | SUSD1 | 197 |
| 15 | 31801967 | 31802608 | OTUD7A | 197 |
| 19 | 8852924 | 8854526 | OR2Z1 | 197 |
| 12 | 54430358 | 54431096 | HOXC4 | 197 |
| 12 | 54430358 | 54431096 | HOXC5 | 197 |
| 2 | 69405192 | 69405804 | ANTXR1 | 197 |
| 18 | 62259780 | 62260347 | SERPINB8 | 196 |
| 3 | 185670028 | 185672280 | TRA2B | 196 |
| 12 | 75723691 | 75724357 | CAPS2 | 196 |
| 19 | 54714122 | 54714703 | MBOAT7 | 196 |
| 16 | 85821299 | 85822026 | COX4NB | 196 |
| 4 | 184364823 | 184366585 | CDKN2AIP | 196 |
| 6 | 116694040 | 116694576 | DSE | 196 |
| 3 | 172428433 | 172429089 | NCEH1 | 196 |
| 9 | 4066543 | 4067111 | GLIS3 | 196 |
| 8 | 12278580 | 12280187 | FAM86B2 | 196 |
| 17 | 48555500 | 48556618 | RSAD1 | 196 |
| X | 49022398 | 49023990 | MAGIX | 196 |
| 9 | 132650432 | 132651144 | FNBP1 | 196 |
| 14 | 77923343 | 77925307 | C14orf133 | 196 |
| 17 | 19088238 | 19088881 | EPN2 | 196 |
| 19 | 49468333 | 49470371 | FTL | 195 |
| 9 | 135472024 | 135473430 | DDX31 | 195 |
| 19 | 33862051 | 33864524 | CEBPG | 195 |
| 6 | 139498220 | 139498334 | HECA | 195 |
| 6 | 126386210 | 126386638 | TRMT11 | 195 |
| 12 | 44439240 | 44439722 | TMEM117 | 195 |
| 2 | 96223179 | 96223799 | TRIM43 | 195 |
| 6 | 149080140 | 149080442 | UST | 195 |
| 18 | 60584651 | 60585500 | PHLPP1 | 195 |
| 8 | 98300732 | 98301352 | TSPYL5 | 195 |
| 5 | 131761843 | 131763259 | SLC22A5 | 195 |
| 7 | 72634241 | 72635447 | NSUN5 | 195 |
| 6 | 34723661 | 34725679 | SNRPC | 195 |
| 3 | 156891001 | 156891548 | CCNL1 | 195 |
| 2 | 69034291 | 69034791 | ARHGAP25 | 195 |
| 12 | 49891064 | 49891540 | SPATS2 | 195 |
| 11 | 72523851 | 72526449 | ATG16L2 | 195 |
| 2 | 85925012 | 85925914 | GNLY | 195 |
| 20 | 5943316 | 5943438 | MCM8 | 194 |
| 1 | 27694274 | 27696272 | MAP3K6 | 194 |
| 10 | 3762446 | 3762524 | KLF6 | 194 |
| 1 | 43397330 | 43398616 | SLC2A1 | 194 |
| 1 | 113242712 | 113244231 | FAM19A3 | 194 |
| 4 | 43134160 | 43134839 | GRXCR1 | 194 |
| 16 | 81534606 | 81536310 | CMIP | 194 |
| 10 | 33540979 | 33541570 | NRP1 | 194 |
| 12 | 46748964 | 46749709 | SLC38A2 | 194 |
| 14 | 107154804 | 107155312 | TMEM121 | 194 |
| 9 | 22131247 | 22131923 | CDKN2B | 194 |
| 15 | 29020040 | 29020725 | APBA2 | 194 |
| 6 | 111196652 | 111197752 | AMD1 | 194 |
| 3 | 25728191 | 25728800 | TOP2B | 194 |
| 17 | 19088988 | 19090521 | EPN2 | 194 |
| 9 | 119311281 | 119312050 | ASTN2 | 194 |
| X | 39959263 | 39959990 | BCOR | 194 |
| 3 | 141241088 | 141241565 | RASA2 | 193 |
| 3 | 72396698 | 72397409 | RYBP | 193 |
| 22 | 24638302 | 24639330 | GGT5 | 193 |
| 1 | 186958148 | 186958735 | PLA2G4A | 193 |
| 10 | 134826353 | 134827811 | C10orf93 | 193 |
| 2 | 25896572 | 25897799 | DTNB | 193 |
| 3 | 156893308 | 156893950 | CCNL1 | 193 |
| 2 | 233948750 | 233949153 | INPP5D | 193 |
| 3 | 128691985 | 128692720 | KIAA1257 | 193 |
| 12 | 8925862 | 8926502 | RIMKLB | 193 |
| 5 | 6766212 | 6767536 | PAPD7 | 193 |
| 6 | 157319172 | 157319904 | ARID1B | 193 |
| 3 | 157737416 | 157737957 | SHOX2 | 193 |
| 20 | 61584939 | 61586537 | SLC17A9 | 193 |
| 3 | 195693857 | 195695444 | TNK2 | 193 |
| 16 | 86857808 | 86858938 | FOXL1 | 193 |
| 1 | 114301271 | 114302638 | PHTF1 | 193 |
| 20 | 34541364 | 34542993 | SCAND1 | 193 |
| 19 | 4916212 | 4917931 | UHRF1 | 193 |
| 7 | 45007189 | 45007731 | MYO1G | 193 |
| 12 | 49109937 | 49111292 | CCNT1 | 193 |
| 2 | 66604264 | 66604791 | MEIS1 | 193 |
| 3 | 148575934 | 148576388 | CPB1 | 193 |
| 6 | 15522807 | 15523718 | DTNBP1 | 193 |
| 1 | 63019588 | 63020291 | DOCK7 | 193 |
| 13 | 88652137 | 88653358 | SLITRK5 | 193 |
| 14 | 100998875 | 101001834 | BEGAIN | 192 |
| 18 | 60618677 | 60618725 | PHLPP1 | 192 |
| 19 | 49155372 | 49156102 | DBP | 192 |
| 8 | 38322835 | 38324213 | FGFR1 | 192 |
| 14 | 53133938 | 53134566 | ERO1L | 192 |
| 3 | 193852163 | 193853362 | HES1 | 192 |
| 13 | 46751213 | 46751915 | LCP1 | 192 |
| 20 | 35224736 | 35224972 | C20orf24 | 192 |
| 20 | 35224736 | 35224972 | TGIF2 | 192 |
| 20 | 35224736 | 35224972 | TGIF2-C20ORF24 | 192 |
| 13 | 84743520 | 84744063 | SLITRK1 | 192 |
| 16 | 25269255 | 25270061 | ZKSCAN2 | 192 |
| 15 | 70739787 | 70741039 | UACA | 192 |
| 18 | 43470879 | 43471421 | EPG5 | 192 |
| 8 | 3359500 | 3360103 | CSMD1 | 192 |
| 15 | 34635759 | 34635866 | NOP10 | 192 |
| 2 | 4610078 | 4610663 | ALLC | 192 |
| 2 | 68561548 | 68562216 | CNRIP1 | 192 |
| 7 | 43660005 | 43660592 | STK17A | 192 |
| 9 | 132625011 | 132625768 | USP20 | 192 |
| 7 | 152085184 | 152085698 | MLL3 | 192 |
| 6 | 89943836 | 89943986 | GABRR1 | 192 |
| 5 | 140985780 | 140986352 | DIAPH1 | 192 |
| 10 | 93652410 | 93652847 | FGFBP3 | 192 |
| 19 | 50701779 | 50701830 | MYH14 | 192 |
| 2 | 8273515 | 8273661 | ID2 | 191 |
| 8 | 28515287 | 28515804 | EXTL3 | 191 |
| 2 | 69055966 | 69056889 | ARHGAP25 | 191 |
| 1 | 145151790 | 145152727 | SEC22B | 191 |
| 19 | 51630216 | 51630867 | SIGLEC9 | 191 |
| 8 | 17128581 | 17129221 | VPS37A | 191 |
| 1 | 37940339 | 37945882 | ZC3H12A | 191 |
| 10 | 439925 | 440955 | DIP2C | 191 |
| 7 | 129709845 | 129710470 | KLHDC10 | 191 |
| 4 | 8262299 | 8264390 | HTRA3 | 191 |
| X | 150565151 | 150565516 | VMA21 | 191 |
| 5 | 139825691 | 139826245 | ANKHD1 | 191 |
| 4 | 26705102 | 26705739 | TBC1D19 | 191 |
| 19 | 42156223 | 42156732 | CEACAM4 | 191 |
| 15 | 50745825 | 50746580 | USP8 | 191 |
| 19 | 16197298 | 16198076 | TPM4 | 191 |
| 2 | 86828761 | 86830301 | RNF103 | 191 |
| 2 | 86828761 | 86830301 | RNF103-CHMP3 | 191 |
| 12 | 32028574 | 32029531 | C12orf35 | 191 |
| 7 | 81890127 | 81890745 | CACNA2D1 | 191 |
| 10 | 88523342 | 88523819 | BMPR1A | 191 |
| 3 | 172118265 | 172119020 | FNDC3B | 191 |
| 6 | 170363475 | 170365763 | C6orf70 | 191 |
| 10 | 29996768 | 29997587 | SVIL | 190 |
| 12 | 42860488 | 42861857 | PRICKLE1 | 190 |
| 1 | 161581897 | 161582473 | FCGR3B | 190 |
| 14 | 101238113 | 101238149 | DLK1 | 190 |
| 12 | 53593864 | 53594274 | ITGB7 | 190 |
| 12 | 52591222 | 52592804 | KRT80 | 190 |
| 19 | 24217181 | 24217380 | ZNF254 | 190 |
| 7 | 94215662 | 94216215 | SGCE | 190 |
| 1 | 212963046 | 212964436 | TATDN3 | 190 |
| 10 | 126299958 | 126301612 | LHPP | 190 |
| 14 | 101909438 | 101910136 | DIO3 | 189 |
| 8 | 99169641 | 99170654 | POP1 | 189 |
| 10 | 7549377 | 7550720 | SFMBT2 | 189 |
| 5 | 34115382 | 34115970 | C1QTNF3 | 189 |
| 12 | 42586485 | 42587524 | YAF2 | 189 |
| 16 | 22618810 | 22618826 | LOC100132247 | 189 |
| 6 | 170267826 | 170269236 | C6orf70 | 189 |
| 3 | 56790300 | 56792310 | ARHGEF3 | 189 |
| 14 | 75749255 | 75749734 | FOS | 189 |
| 9 | 137287594 | 137289483 | RXRA | 189 |
| 6 | 69516909 | 69517584 | BAI3 | 189 |
| 5 | 176299412 | 176300197 | UNC5A | 189 |
| 7 | 158532614 | 158533130 | ESYT2 | 189 |
| 11 | 70243392 | 70244943 | CTTN | 189 |
| 1 | 236101071 | 236101536 | LYST | 189 |
| 12 | 125211580 | 125212658 | SCARB1 | 189 |
| 1 | 21409003 | 21409748 | EIF4G3 | 189 |
| 4 | 40673818 | 40674502 | RBM47 | 189 |
| 17 | 55776766 | 55776828 | MRPS23 | 189 |
| 4 | 6839743 | 6839780 | KIAA0232 | 189 |
| 12 | 76952568 | 76953761 | OSBPL8 | 189 |
| 12 | 7828164 | 7829562 | APOBEC1 | 188 |
| 2 | 240161403 | 240162038 | HDAC4 | 188 |
| 16 | 58162885 | 58164465 | C16orf80 | 188 |
| 11 | 100551462 | 100551664 | ARHGAP42 | 188 |
| 11 | 117686044 | 117688080 | DSCAML1 | 188 |
| 6 | 25991867 | 25993326 | HIST1H4A | 188 |
| 12 | 10338721 | 10339219 | C12orf59 | 188 |
| 3 | 151553169 | 151553610 | AADAC | 188 |
| 19 | 42314486 | 42315856 | CEACAM3 | 188 |
| 3 | 120314636 | 120315778 | NDUFB4 | 188 |
| 10 | 17270161 | 17271093 | VIM | 188 |
| 3 | 180651391 | 180651806 | FXR1 | 188 |
| 17 | 45780418 | 45781125 | TBKBP1 | 188 |
| 3 | 188224436 | 188224880 | LPP | 188 |
| 10 | 69523100 | 69524418 | CTNNA3 | 188 |
| 15 | 28517756 | 28518366 | HERC2 | 188 |
| 8 | 29229956 | 29231379 | DUSP4 | 188 |
| 18 | 33708739 | 33710412 | ELP2 | 188 |
| 10 | 118791673 | 118792134 | KIAA1598 | 188 |
| 19 | 8895548 | 8896055 | ZNF558 | 188 |
| 2 | 11486214 | 11486728 | ROCK2 | 188 |
| 11 | 129099477 | 129100100 | ARHGAP32 | 187 |
| 9 | 127056914 | 127058308 | NEK6 | 187 |
| 11 | 56154776 | 56155292 | OR8U8 | 187 |
| 10 | 30317187 | 30317216 | KIAA1462 | 187 |
| 18 | 32160750 | 32161469 | DTNA | 187 |
| 1 | 50648382 | 50649661 | ELAVL4 | 187 |
| 12 | 7499945 | 7500486 | ACSM4 | 187 |
| 9 | 127961959 | 127963311 | RABEPK | 187 |
| 5 | 32113645 | 32114357 | PDZD2 | 187 |
| 7 | 150062458 | 150062909 | RARRES2 | 187 |
| 16 | 81516984 | 81518430 | CMIP | 187 |
| 7 | 73638478 | 73640890 | LAT2 | 187 |
| 6 | 27162415 | 27163115 | HIST1H2AH | 187 |
| 3 | 187819938 | 187820567 | LPP | 187 |
| 2 | 65064338 | 65065396 | SLC1A4 | 187 |
| 8 | 73977646 | 73978105 | C8orf84 | 187 |
| 2 | 28658984 | 28659905 | FOSL2 | 187 |
| 21 | 44097512 | 44098050 | PDE9A | 187 |
| 7 | 155556192 | 155557680 | RBM33 | 187 |
| 7 | 32529623 | 32530327 | LSM5 | 187 |
| 18 | 46475197 | 46476342 | SMAD7 | 187 |
| 2 | 28268777 | 28269415 | BRE | 187 |
| 7 | 17363942 | 17364485 | AHR | 187 |
| 5 | 115836294 | 115836800 | SEMA6A | 187 |
| 10 | 105670835 | 105671277 | OBFC1 | 186 |
| 3 | 161769620 | 161770146 | OTOL1 | 186 |
| 1 | 147400452 | 147401219 | GPR89C | 186 |
| 1 | 147400452 | 147401219 | GPR89B | 186 |
| 22 | 37638146 | 37639184 | RAC2 | 186 |
| 18 | 72370023 | 72370463 | ZNF407 | 186 |
| 15 | 28742791 | 28744336 | HERC2 | 186 |
| 13 | 89989330 | 89990007 | SLITRK5 | 186 |
| 13 | 92664805 | 92665454 | GPC5 | 186 |
| 1 | 27481111 | 27481203 | SLC9A1 | 186 |
| 12 | 38591167 | 38591952 | ALG10B | 186 |
| 10 | 76701371 | 76701967 | KAT6B | 186 |
| 19 | 56277825 | 56278337 | NLRP9 | 186 |
| 9 | 132651386 | 132651526 | FNBP1 | 186 |
| 1 | 153764162 | 153764911 | SLC27A3 | 186 |
| 6 | 57402303 | 57403160 | PRIM2 | 186 |
| 12 | 31939271 | 31940389 | H3F3C | 186 |
| 22 | 24798220 | 24798757 | SPECC1L | 186 |
| 10 | 72625886 | 72626198 | SGPL1 | 186 |
| 7 | 152084351 | 152085138 | MLL3 | 185 |
| 1 | 150156928 | 150157581 | PLEKHO1 | 185 |
| 6 | 11382212 | 11382718 | NEDD9 | 185 |
| 19 | 17334938 | 17336979 | OCEL1 | 185 |
| 5 | 97912314 | 97912751 | RGMB | 185 |
| 12 | 58335922 | 58336081 | XRCC6BP1 | 185 |
| 4 | 41377368 | 41377885 | LIMCH1 | 185 |
| 2 | 65474092 | 65475044 | ACTR2 | 185 |
| 16 | 81745029 | 81747992 | CMIP | 185 |
| 1 | 43202523 | 43203661 | CLDN19 | 185 |
| 5 | 93677405 | 93678056 | KIAA0825 | 185 |
| 12 | 6646476 | 6650239 | GAPDH | 185 |
| 6 | 24699138 | 24699896 | ACOT13 | 185 |
| 1 | 11024817 | 11025015 | C1orf127 | 185 |
| 10 | 6092417 | 6093667 | IL2RA | 185 |
| 3 | 158034085 | 158034640 | RSRC1 | 185 |
| 10 | 106085256 | 106086492 | ITPRIP | 185 |
| 7 | 130571041 | 130571528 | KLF14 | 185 |
| X | 15872950 | 15873851 | AP1S2 | 185 |
| 3 | 138684373 | 138684827 | FOXL2 | 185 |
| 15 | 55699977 | 55701461 | CCPG1 | 185 |
| 12 | 8130356 | 8130879 | SLC2A3 | 185 |
| 21 | 38616159 | 38616962 | DSCR3 | 184 |
| 8 | 104057769 | 104058284 | ATP6V1C1 | 184 |
| 16 | 27394774 | 27395450 | IL21R | 184 |
| 7 | 74182451 | 74182616 | NCF1 | 184 |
| 15 | 72978024 | 72978817 | BBS4 | 184 |
| 20 | 23636043 | 23637444 | CST3 | 184 |
| 14 | 91716296 | 91716938 | GPR68 | 184 |
| 9 | 69048317 | 69049147 | FOXD4L6 | 184 |
| 13 | 91344651 | 91345485 | GPC5 | 184 |
| 1 | 105742890 | 105743622 | AMY1B | 184 |
| 17 | 29034737 | 29036078 | CRLF3 | 184 |
| 12 | 46294506 | 46295532 | ARID2 | 184 |
| 12 | 110925756 | 110926172 | C12orf24 | 184 |
| 12 | 110925756 | 110926172 | GPN3 | 184 |
| 6 | 139694569 | 139696975 | CITED2 | 184 |
| 1 | 28213792 | 28215386 | C1orf38 | 184 |
| 6 | 34230876 | 34231834 | C6orf1 | 184 |
| 2 | 170589798 | 170589970 | KLHL23 | 184 |
| 21 | 37801691 | 37803041 | CLDN14 | 184 |
| 2 | 170589798 | 170589970 | PHOSPHO2-KLHL23 | 184 |
| 1 | 121097536 | 121097616 | FCGR1B | 184 |
| 22 | 50318995 | 50319889 | CRELD2 | 184 |
| 6 | 52950455 | 52951011 | FBXO9 | 184 |
| 12 | 116843878 | 116844458 | MED13L | 184 |
| 6 | 166850704 | 166851351 | RPS6KA2 | 184 |
| 9 | 92935641 | 92936082 | DIRAS2 | 184 |
| 16 | 50742438 | 50742984 | NOD2 | 184 |
| 1 | 109618238 | 109619151 | TAF13 | 184 |
| 7 | 56131395 | 56133108 | SUMF2 | 184 |
| 10 | 47136553 | 47137405 | ANXA8L1 | 184 |
| 6 | 157308213 | 157308764 | ARID1B | 183 |
| 19 | 47729433 | 47730705 | BBC3 | 183 |
| 11 | 75062925 | 75063811 | ARRB1 | 183 |
| 11 | 100552322 | 100552454 | ARHGAP42 | 183 |
| 7 | 122974598 | 122975069 | SLC13A1 | 183 |
| 3 | 115394961 | 115395634 | GAP43 | 183 |
| 1 | 154154778 | 154156059 | TPM3 | 183 |
| 17 | 26949573 | 26950209 | KIAA0100 | 183 |
| 15 | 44859271 | 44859779 | SPG11 | 183 |
| 15 | 44859271 | 44859779 | EIF3J | 183 |
| 4 | 20694615 | 20695215 | PACRGL | 183 |
| 12 | 40286731 | 40287624 | SLC2A13 | 183 |
| 18 | 33758890 | 33759508 | MOCOS | 183 |
| 13 | 87445628 | 87446864 | SLITRK5 | 183 |
| 19 | 13212981 | 13217321 | LYL1 | 183 |
| 19 | 2474601 | 2477660 | GADD45B | 183 |
| 3 | 52277706 | 52278853 | PPM1M | 183 |
| 10 | 89621420 | 89623016 | PTEN | 183 |
| 10 | 89621420 | 89623016 | KLLN | 183 |
| 19 | 55727443 | 55729300 | PTPRH | 183 |
| 1 | 167570852 | 167571191 | RCSD1 | 183 |
| 11 | 67513691 | 67514001 | ALDH3B2 | 182 |
| 17 | 71175227 | 71175608 | COG1 | 182 |
| 3 | 80688775 | 80689296 | ROBO1 | 182 |
| 18 | 29738279 | 29738830 | MEP1B | 182 |
| 11 | 110333814 | 110334242 | FDX1 | 182 |
| 1 | 77684560 | 77685376 | PIGK | 182 |
| 6 | 35899579 | 35900421 | SRPK1 | 182 |
| 1 | 235530410 | 235531800 | TBCE | 182 |
| 7 | 75056269 | 75056742 | POM121C | 182 |
| 3 | 135913830 | 135914676 | MSL2 | 182 |
| 6 | 26055657 | 26056937 | HIST1H1C | 182 |
| 19 | 36370392 | 36371508 | NPHS1 | 182 |
| 5 | 43017866 | 43019042 | C5orf39 | 182 |
| 19 | 12257543 | 12258146 | ZNF625 | 182 |
| 1 | 249167724 | 249168137 | ZNF692 | 182 |
| 4 | 77522601 | 77523115 | SHROOM3 | 182 |
| 3 | 183044620 | 183045207 | MCF2L2 | 182 |
| 22 | 22294820 | 22295631 | PPM1F | 182 |
| 1 | 26097180 | 26098698 | MAN1C1 | 182 |
| 3 | 195772943 | 195772944 | TFRC | 182 |
| 7 | 152162527 | 152163024 | MLL3 | 182 |
| 6 | 57711302 | 57712297 | PRIM2 | 182 |
| 19 | 28959512 | 28960391 | UQCRFS1 | 182 |
| 7 | 114101789 | 114102630 | FOXP2 | 182 |
| 13 | 88528279 | 88529909 | SLITRK5 | 182 |
| 1 | 34965846 | 34966397 | GJB5 | 182 |
| 3 | 183966301 | 183968239 | ALG3 | 182 |
| 18 | 253749 | 254163 | THOC1 | 182 |
| 9 | 6834855 | 6835613 | KDM4C | 182 |
| 10 | 11379274 | 11380009 | CELF2 | 181 |
| 19 | 11388135 | 11389279 | TSPAN16 | 181 |
| 10 | 104951840 | 104952862 | NT5C2 | 181 |
| 5 | 81262036 | 81262885 | ATG10 | 181 |
| 3 | 97698362 | 97699026 | MINA | 181 |
| 11 | 62323698 | 62324476 | AHNAK | 181 |
| 12 | 6275669 | 6276554 | CD9 | 181 |
| 15 | 99663562 | 99664311 | SYNM | 181 |
| 12 | 9860362 | 9860805 | CLECL1 | 181 |
| 6 | 110092369 | 110092965 | FIG4 | 181 |
| 1 | 94312601 | 94313807 | DNTTIP2 | 181 |
| 14 | 71283877 | 71284417 | MAP3K9 | 181 |
| 7 | 149473060 | 149473166 | SSPO | 181 |
| 1 | 93644435 | 93644964 | CCDC18 | 181 |
| 16 | 23463446 | 23464876 | COG7 | 180 |
| 3 | 183481417 | 183482658 | YEATS2 | 180 |
| 1 | 9434138 | 9434447 | SPSB1 | 180 |
| 17 | 41818815 | 41819180 | SOST | 180 |
| 1 | 36840849 | 36841201 | STK40 | 180 |
| 17 | 30467747 | 30468447 | RHOT1 | 180 |
| 16 | 29329506 | 29329846 | SLX1B | 180 |
| 7 | 1311430 | 1312655 | UNCX | 180 |
| 15 | 70785836 | 70786593 | UACA | 180 |
| 12 | 4398275 | 4399425 | CCND2 | 180 |
| 13 | 81210314 | 81210791 | SPRY2 | 180 |
| 15 | 50741445 | 50742528 | USP8 | 180 |
| 9 | 3755305 | 3755779 | RFX3 | 180 |
| 5 | 150529678 | 150530623 | ANXA6 | 180 |
| 9 | 19017631 | 19017656 | FAM154A | 180 |
| 1 | 235371728 | 235372227 | ARID4B | 180 |
| 11 | 67046385 | 67046604 | ADRBK1 | 180 |
| 5 | 130970284 | 130971363 | RAPGEF6 | 180 |
| 15 | 90577549 | 90579374 | ZNF710 | 180 |
| 10 | 86389967 | 86390445 | FAM190B | 180 |
| 21 | 36244360 | 36245016 | RUNX1 | 180 |
| 6 | 166608556 | 166609680 | T | 180 |
| 1 | 60256502 | 60257045 | HOOK1 | 180 |
| 19 | 6642582 | 6642703 | TNFSF14 | 180 |
| 13 | 90810148 | 90810796 | GPC5 | 179 |
| 1 | 19254556 | 19254821 | IFFO2 | 179 |
| 12 | 9250997 | 9251457 | A2M | 179 |
| 13 | 78925487 | 78926463 | POU4F1 | 179 |
| 12 | 41239833 | 41241294 | CNTN1 | 179 |
| 18 | 74330936 | 74331482 | ZNF516 | 179 |
| 16 | 72821869 | 72822325 | ZFHX3 | 179 |
| 7 | 156715506 | 156716543 | NOM1 | 179 |
| 19 | 12635580 | 12636175 | ZNF564 | 179 |
| 8 | 103854158 | 103855559 | AZIN1 | 179 |
| 10 | 98841922 | 98842158 | SLIT1 | 179 |
| 17 | 77801605 | 77802542 | CBX4 | 179 |
| 3 | 194673427 | 194674399 | FAM43A | 179 |
| 21 | 45333612 | 45335553 | AGPAT3 | 179 |
| 3 | 172658271 | 172658914 | SPATA16 | 179 |
| 3 | 196521820 | 196523311 | PAK2 | 179 |
| 6 | 27429663 | 27430448 | ZNF184 | 179 |
| 12 | 46322656 | 46323548 | SCAF11 | 179 |
| 2 | 47403063 | 47404255 | CALM2 | 179 |
| 13 | 82462802 | 82463772 | SPRY2 | 179 |
| 14 | 102548708 | 102549424 | HSP90AA1 | 179 |
| 6 | 21618285 | 21618904 | SOX4 | 179 |
| 8 | 56756755 | 56757484 | LYN | 179 |
| X | 77393860 | 77394330 | TAF9B | 179 |
| X | 38664625 | 38665356 | MID1IP1 | 179 |
| 12 | 111882326 | 111883638 | SH2B3 | 179 |
| 2 | 201953783 | 201954476 | CFLAR | 179 |
| X | 53803554 | 53804255 | HUWE1 | 179 |
| 6 | 12008519 | 12009395 | HIVEP1 | 179 |
| 17 | 61628852 | 61629570 | DCAF7 | 179 |
| 6 | 26123435 | 26124149 | HIST1H2AC | 179 |
| 17 | 65625260 | 65626085 | PITPNC1 | 179 |
| 17 | 48559217 | 48560082 | RSAD1 | 178 |
| 8 | 30252552 | 30252758 | RBPMS | 178 |
| 5 | 139815752 | 139816230 | ANKHD1 | 178 |
| 6 | 158185435 | 158185886 | SNX9 | 178 |
| 9 | 27635468 | 27636138 | C9orf72 | 178 |
| 10 | 121277263 | 121278121 | RGS10 | 178 |
| 10 | 121064654 | 121064960 | GRK5 | 178 |
| 2 | 65867049 | 65867734 | SPRED2 | 178 |
| 9 | 68379361 | 68381078 | ANKRD20A3 | 178 |
| 5 | 64858637 | 64859424 | PPWD1 | 178 |
| 22 | 18253813 | 18254357 | BID | 178 |
| 20 | 50158405 | 50158489 | NFATC2 | 178 |
| 3 | 196414400 | 196415029 | PIGX | 178 |
| 3 | 128564038 | 128565945 | ACAD9 | 178 |
| 18 | 75652317 | 75652939 | GALR1 | 178 |
| 3 | 114610393 | 114611048 | ZBTB20 | 177 |
| 12 | 110871207 | 110872173 | ARPC3 | 177 |
| 17 | 8151160 | 8152934 | CTC1 | 177 |
| 17 | 73024131 | 73024702 | KCTD2 | 177 |
| 8 | 68644569 | 68645284 | CPA6 | 177 |
| 4 | 3573046 | 3574882 | LRPAP1 | 177 |
| 15 | 42008133 | 42008723 | MGA | 177 |
| 1 | 106930589 | 106931200 | PRMT6 | 177 |
| 11 | 60838456 | 60839261 | CD5 | 177 |
| 8 | 134990620 | 134991181 | ST3GAL1 | 177 |
| 1 | 42046239 | 42047657 | HIVEP3 | 177 |
| 5 | 150179988 | 150180554 | C5orf62 | 177 |
| 1 | 200251547 | 200252132 | FAM58BP | 177 |
| 16 | 29311447 | 29311869 | SLX1B | 177 |
| 2 | 207023626 | 207024793 | NDUFS1 | 177 |
| 22 | 16133834 | 16134270 | POTEH | 177 |
| 7 | 111953195 | 111953588 | ZNF277 | 177 |
| 11 | 82904281 | 82905753 | ANKRD42 | 177 |
| 8 | 84957930 | 84958311 | RALYL | 177 |
| 22 | 45630837 | 45631922 | KIAA0930 | 177 |
| 5 | 118609179 | 118609616 | TNFAIP8 | 177 |
| 9 | 43840984 | 43841488 | CNTNAP3B | 177 |
| 11 | 72526551 | 72527639 | ATG16L2 | 177 |
| 22 | 45060671 | 45061354 | PRR5 | 177 |
| 6 | 27099141 | 27101927 | HIST1H2AG | 177 |
| 2 | 24365741 | 24366318 | LOC375190 | 177 |
| 2 | 46953990 | 46954673 | SOCS5 | 177 |
| 6 | 34726022 | 34726952 | SNRPC | 177 |
| 8 | 110460325 | 110460976 | PKHD1L1 | 177 |
| 8 | 3411948 | 3412989 | CSMD1 | 177 |
| 11 | 69062793 | 69063879 | MYEOV | 177 |
| 2 | 65913413 | 65913976 | SPRED2 | 177 |
| 7 | 25949683 | 25950120 | NFE2L3 | 176 |
| 20 | 47367639 | 47368315 | PREX1 | 176 |
| 4 | 6920367 | 6921806 | TBC1D14 | 176 |
| 4 | 33845818 | 33846823 | ARAP2 | 176 |
| 17 | 62788295 | 62788785 | LRRC37A3 | 176 |
| 14 | 104859729 | 104861058 | C14orf180 | 176 |
| 11 | 125756607 | 125758817 | HYLS1 | 176 |
| 10 | 111900003 | 111900932 | MXI1 | 176 |
| 5 | 10680873 | 10683635 | DAP | 176 |
| 3 | 193720376 | 193723051 | HES1 | 176 |
| 12 | 41373858 | 41374456 | CNTN1 | 176 |
| 15 | 20443689 | 20444340 | GOLGA6L6 | 176 |
| 3 | 13455234 | 13456275 | NUP210 | 176 |
| 12 | 27098293 | 27098957 | FGFR1OP2 | 176 |
| 6 | 143557083 | 143557589 | AIG1 | 176 |
| 2 | 64782988 | 64783527 | AFTPH | 176 |
| 4 | 19457781 | 19458916 | SLIT2 | 176 |
| 2 | 103420030 | 103420638 | TMEM182 | 176 |
| 7 | 9336912 | 9337541 | NXPH1 | 176 |
| 10 | 8124752 | 8126043 | GATA3 | 176 |
| 6 | 138769613 | 138770423 | NHSL1 | 176 |
| 6 | 72082340 | 72082893 | OGFRL1 | 176 |
| 3 | 141280691 | 141281691 | RASA2 | 176 |
| X | 68062458 | 68063393 | EFNB1 | 176 |
| 9 | 30548676 | 30549294 | LINGO2 | 176 |
| 3 | 128693238 | 128693926 | KIAA1257 | 176 |
| 2 | 85536697 | 85537449 | TCF7L1 | 176 |
| 7 | 77211125 | 77212063 | PTPN12 | 176 |
| 3 | 185813391 | 185814026 | ETV5 | 176 |
| 3 | 52086299 | 52088329 | DUSP7 | 176 |
| 3 | 121431636 | 121432088 | GOLGB1 | 175 |
| 16 | 78600924 | 78601463 | WWOX | 175 |
| 17 | 65866684 | 65867618 | BPTF | 175 |
| 9 | 32016130 | 32016915 | ACO1 | 175 |
| 16 | 85083366 | 85084660 | KIAA0513 | 175 |
| 3 | 138839036 | 138840117 | PRR23C | 175 |
| 17 | 74024644 | 74026783 | EVPL | 175 |
| 15 | 90136578 | 90137014 | C15orf42 | 175 |
| 8 | 3579108 | 3580238 | CSMD1 | 175 |
| 5 | 40114909 | 40115444 | PTGER4 | 175 |
| 10 | 34004168 | 34004650 | NRP1 | 175 |
| 6 | 43279979 | 43280667 | CRIP3 | 175 |
| 11 | 62609439 | 62610580 | SLC3A2 | 175 |
| 14 | 101142164 | 101145581 | DLK1 | 175 |
| 3 | 156891878 | 156893120 | CCNL1 | 175 |
| 6 | 37437062 | 37437541 | FTSJD2 | 175 |
| 4 | 36521623 | 36522391 | DTHD1 | 175 |
| 19 | 55764683 | 55766575 | PPP6R1 | 175 |
| 12 | 25404079 | 25404674 | KRAS | 175 |
| 21 | 45663204 | 45664420 | ICOSLG | 175 |
| 10 | 6159203 | 6160136 | PFKFB3 | 175 |
| 14 | 31687883 | 31687991 | HECTD1 | 175 |
| 17 | 36324483 | 36325140 | TBC1D3 | 174 |
| 9 | 98902239 | 98902338 | HSD17B3 | 174 |
| 8 | 61821137 | 61821680 | CHD7 | 174 |
| 21 | 40392441 | 40393418 | PSMG1 | 174 |
| 10 | 94449714 | 94452168 | HHEX | 174 |
| 10 | 134384367 | 134386127 | INPP5A | 174 |
| 9 | 102583570 | 102585239 | NR4A3 | 174 |
| 7 | 8163471 | 8164124 | ICA1 | 174 |
| 2 | 89157378 | 89157874 | RPIA | 174 |
| 19 | 42746812 | 42748485 | GSK3A | 174 |
| 3 | 190671607 | 190672526 | GMNC | 174 |
| 5 | 35002971 | 35003563 | AGXT2 | 174 |
| 18 | 57587229 | 57588083 | PMAIP1 | 174 |
| 1 | 93816895 | 93817907 | DR1 | 174 |
| 3 | 195696538 | 195698203 | TNK2 | 174 |
| 6 | 98386279 | 98386848 | MMS22L | 174 |
| 17 | 1100721 | 1102282 | ABR | 174 |
| 3 | 196518005 | 196518008 | PAK2 | 174 |
| 16 | 19078763 | 19079770 | COQ7 | 174 |
| 22 | 24542111 | 24542770 | CABIN1 | 174 |
| 10 | 38320269 | 38320964 | ZNF33A | 174 |
| 6 | 43692706 | 43693346 | MRPS18A | 174 |
| 8 | 100285205 | 100286018 | VPS13B | 174 |
| 7 | 10898484 | 10898893 | NDUFA4 | 174 |
| 2 | 109833998 | 109834609 | SH3RF3 | 174 |
| 11 | 125298418 | 125299248 | PKNOX2 | 174 |
| 7 | 21983509 | 21986016 | CDCA7L | 174 |
| 12 | 116996804 | 116997627 | MAP1LC3B2 | 174 |
| 20 | 23104347 | 23105202 | CD93 | 174 |
| 17 | 75384043 | 75387078 | SEPT9 | 174 |
| 4 | 185605633 | 185605696 | CCDC111 | 174 |
| 13 | 75109768 | 75111504 | KLF12 | 173 |
| 3 | 186114286 | 186115195 | DGKG | 173 |
| 1 | 100875706 | 100876166 | CDC14A | 173 |
| 7 | 154735577 | 154736200 | PAXIP1 | 173 |
| 4 | 5981592 | 5982279 | CRMP1 | 173 |
| 1 | 16999319 | 17001343 | NBPF1 | 173 |
| X | 1454810 | 1455654 | IL3RA | 173 |
| 14 | 69184139 | 69184824 | ZFP36L1 | 173 |
| 10 | 99896296 | 99896993 | C10orf28 | 173 |
| 17 | 80548537 | 80551025 | FOXK2 | 173 |
| 15 | 35231136 | 35231705 | AQR | 173 |
| 3 | 149382108 | 149382698 | WWTR1 | 173 |
| 16 | 89181229 | 89182324 | ACSF3 | 173 |
| 7 | 131114162 | 131114640 | MKLN1 | 173 |
| 9 | 94668160 | 94668746 | ROR2 | 173 |
| 3 | 188013099 | 188013785 | LPP | 173 |
| 10 | 72335095 | 72335516 | PRF1 | 173 |
| 8 | 96950092 | 96950586 | GDF6 | 173 |
| 6 | 134496096 | 134496884 | SGK1 | 173 |
| 12 | 124866053 | 124866746 | NCOR2 | 173 |
| 10 | 71257328 | 71258360 | TSPAN15 | 173 |
| 7 | 52003868 | 52004555 | COBL | 173 |
| 11 | 128597249 | 128598500 | FLI1 | 173 |
| 8 | 56685159 | 56686550 | TMEM68 | 173 |
| 8 | 56685159 | 56686550 | TGS1 | 173 |
| 5 | 16556084 | 16556593 | FAM134B | 172 |
| 18 | 60617882 | 60618648 | PHLPP1 | 172 |
| 18 | 12488696 | 12489330 | SPIRE1 | 172 |
| 3 | 196388765 | 196389679 | LRRC33 | 172 |
| 19 | 2268957 | 2270789 | OAZ1 | 172 |
| 8 | 97730045 | 97732125 | PGCP | 172 |
| 8 | 6419238 | 6421000 | MCPH1 | 172 |
| 8 | 6419238 | 6421000 | ANGPT2 | 172 |
| 18 | 48888367 | 48888555 | MEX3C | 172 |
| 9 | 26909365 | 26909862 | PLAA | 172 |
| 21 | 36159302 | 36160214 | RUNX1 | 172 |
| 19 | 15056050 | 15056627 | OR7C2 | 172 |
| 1 | 36803695 | 36806576 | FAM176B | 172 |
| 10 | 22292266 | 22292290 | DNAJC1 | 172 |
| 13 | 92633018 | 92634108 | GPC5 | 172 |
| 18 | 2591590 | 2592147 | NDC80 | 172 |
| 7 | 101497974 | 101499613 | CUX1 | 172 |
| 3 | 21109148 | 21109794 | ZNF385D | 172 |
| 11 | 121335063 | 121335640 | SORL1 | 172 |
| 1 | 121272349 | 121272931 | FCGR1B | 172 |
| 7 | 56133120 | 56133869 | SUMF2 | 172 |
| 12 | 53810476 | 53810928 | PRR13 | 172 |
| 6 | 72176648 | 72177164 | OGFRL1 | 172 |
| 13 | 74069580 | 74070415 | KLF5 | 172 |
| 14 | 31688106 | 31688504 | HECTD1 | 172 |
| 7 | 89574337 | 89574866 | STEAP1 | 172 |
| 6 | 144954281 | 144954805 | UTRN | 172 |
| 3 | 189211519 | 189212183 | TP63 | 172 |
| 20 | 30159441 | 30161010 | HM13 | 172 |
| 11 | 46681918 | 46682145 | ATG13 | 172 |
| 7 | 22007865 | 22008144 | CDCA7L | 172 |
| 6 | 149998850 | 149999549 | LATS1 | 172 |
| 1 | 114524818 | 114525819 | OLFML3 | 172 |
| 1 | 120751987 | 120752640 | FAM72B | 172 |
| 8 | 99976939 | 99978218 | OSR2 | 172 |
| 6 | 24169502 | 24170172 | DCDC2 | 172 |
| 20 | 3401533 | 3401696 | C20orf194 | 172 |
| 3 | 134325778 | 134326742 | KY | 171 |
| 2 | 232578114 | 232579674 | PTMA | 171 |
| 15 | 44758563 | 44759230 | CTDSPL2 | 171 |
| 17 | 72460105 | 72461055 | CD300A | 171 |
| 3 | 190142241 | 190142681 | TMEM207 | 171 |
| 3 | 194141881 | 194142534 | ATP13A3 | 171 |
| 3 | 156108268 | 156108736 | KCNAB1 | 171 |
| 1 | 11799687 | 11800248 | AGTRAP | 171 |
| 18 | 77547228 | 77547654 | KCNG2 | 171 |
| 8 | 103236077 | 103236858 | RRM2B | 171 |
| 19 | 9306084 | 9306649 | OR7D2 | 171 |
| 1 | 52498320 | 52499594 | KTI12 | 171 |
| 1 | 52498320 | 52499594 | TXNDC12 | 171 |
| 11 | 120096047 | 120096471 | OAF | 171 |
| 11 | 124102228 | 124102692 | OR8G1 | 171 |
| 21 | 46062235 | 46062908 | KRTAP10-10 | 171 |
| 21 | 46062235 | 46062908 | KRTAP10-11 | 171 |
| 21 | 46062235 | 46062908 | KRTAP12-3 | 171 |
| 21 | 46062235 | 46062908 | TSPEAR | 171 |
| 4 | 26875094 | 26876133 | STIM2 | 171 |
| 21 | 43305064 | 43305798 | C2CD2 | 171 |
| 22 | 23226898 | 23227739 | IGLL5 | 171 |
| 5 | 145561100 | 145561927 | LARS | 171 |
| 17 | 57264664 | 57265303 | PRR11 | 171 |
| 12 | 41241402 | 41242257 | CNTN1 | 171 |
| 2 | 238383258 | 238383900 | MLPH | 171 |
| 13 | 110790861 | 110791364 | COL4A1 | 171 |
| 6 | 36510175 | 36511746 | STK38 | 171 |
| 15 | 66683358 | 66683897 | MAP2K1 | 171 |
| 3 | 194670708 | 194672717 | FAM43A | 171 |
| 15 | 83679626 | 83681128 | C15orf40 | 171 |
| 16 | 29816025 | 29817256 | MAZ | 171 |
| X | 40026412 | 40027809 | BCOR | 171 |
| 8 | 81939586 | 81940041 | PAG1 | 171 |
| 18 | 60427932 | 60428339 | PHLPP1 | 171 |
| 1 | 28975385 | 28976984 | GMEB1 | 171 |
| 1 | 117591937 | 117592454 | TTF2 | 170 |
| 1 | 150184302 | 150186185 | ANP32E | 170 |
| 7 | 72131554 | 72132731 | TYW1B | 170 |
| 3 | 184415045 | 184415980 | MAGEF1 | 170 |
| Y | 233963 | 234835 | GTPBP6 | 170 |
| 12 | 124937506 | 124938844 | NCOR2 | 170 |
| 10 | 1047670 | 1048242 | GTPBP4 | 170 |
| 5 | 10526601 | 10527365 | ANKRD33B | 170 |
| 2 | 39572599 | 39573104 | MAP4K3 | 170 |
| 2 | 99953316 | 99954250 | TXNDC9 | 170 |
| 14 | 104375296 | 104376385 | TDRD9 | 170 |
| 16 | 66906655 | 66907423 | PDP2 | 170 |
| 2 | 240188658 | 240189587 | HDAC4 | 170 |
| 5 | 43041820 | 43043711 | C5orf39 | 170 |
| 10 | 18947540 | 18947846 | ARL5B | 170 |
| 9 | 19642567 | 19643097 | SLC24A2 | 170 |
| 2 | 179344597 | 179345079 | FKBP7 | 170 |
| 10 | 94740423 | 94740864 | EXOC6 | 170 |
| 2 | 72880930 | 72881454 | EXOC6B | 170 |
| 9 | 137159147 | 137160798 | RXRA | 170 |
| 1 | 25875423 | 25876637 | LDLRAP1 | 170 |
| 3 | 195958912 | 195959514 | OSTalpha | 170 |
| 2 | 160587969 | 160588677 | MARCH7 | 170 |
| 10 | 134386311 | 134386542 | INPP5A | 170 |
| 15 | 73989142 | 73989967 | CD276 | 169 |
| 2 | 32625234 | 32625804 | BIRC6 | 169 |
| 14 | 35228326 | 35228858 | BAZ1A | 169 |
| 21 | 15656053 | 15656493 | RBM11 | 169 |
| 1 | 184710016 | 184710599 | EDEM3 | 169 |
| 7 | 41739588 | 41740046 | INHBA | 169 |
| 6 | 33702869 | 33703830 | IP6K3 | 169 |
| 7 | 75957454 | 75958085 | YWHAG | 169 |
| 5 | 159894355 | 159895416 | PTTG1 | 169 |
| 15 | 75991521 | 75991697 | CSPG4 | 169 |
| 11 | 67819267 | 67821059 | TCIRG1 | 169 |
| 2 | 153451426 | 153451465 | FMNL2 | 169 |
| 1 | 178254084 | 178254738 | RASAL2 | 169 |
| 3 | 194023480 | 194024939 | CPN2 | 169 |
| 3 | 185017776 | 185018694 | MAP3K13 | 169 |
| 16 | 4070399 | 4070894 | ADCY9 | 169 |
| 1 | 35865226 | 35866207 | ZMYM4 | 169 |
| 18 | 11850978 | 11852210 | CHMP1B | 169 |
| 18 | 11850978 | 11852210 | GNAL | 169 |
| 14 | 35016091 | 35016849 | EAPP | 169 |
| 1 | 144011356 | 144012119 | FAM72D | 169 |
| 9 | 123658867 | 123659919 | PHF19 | 169 |
| 7 | 3128775 | 3129427 | CARD11 | 169 |
| 17 | 19014280 | 19016561 | GRAPL | 169 |
| 9 | 73222699 | 73222811 | TRPM3 | 169 |
| 14 | 69208411 | 69209311 | ZFP36L1 | 169 |
| 11 | 126186311 | 126187378 | DCPS | 169 |
| 22 | 36847400 | 36848484 | TXN2 | 168 |
| 14 | 32030281 | 32030999 | NUBPL | 168 |
| 20 | 29438898 | 29439584 | DEFB115 | 168 |
| 7 | 72394796 | 72395034 | POM121 | 168 |
| 10 | 49719628 | 49719983 | ARHGAP22 | 168 |
| 2 | 82805024 | 82805677 | SUCLG1 | 168 |
| 3 | 176531434 | 176531906 | TBL1XR1 | 168 |
| 5 | 54468511 | 54469158 | CDC20B | 168 |
| 3 | 177001125 | 177001922 | TBL1XR1 | 168 |
| 10 | 105667364 | 105668509 | OBFC1 | 168 |
| 2 | 112231989 | 112232260 | BCL2L11 | 168 |
| 3 | 126248501 | 126250257 | CHST13 | 168 |
| 10 | 124982523 | 124983033 | BUB3 | 168 |
| 18 | 46973736 | 46974159 | DYM | 168 |
| 9 | 66519521 | 66520863 | FAM75A5 | 168 |
| 5 | 530511 | 531026 | SLC9A3 | 168 |
| 8 | 37757165 | 37757909 | RAB11FIP1 | 168 |
| 13 | 113239944 | 113240622 | TUBGCP3 | 168 |
| 12 | 125398343 | 125399789 | UBC | 168 |
| 4 | 37528984 | 37529688 | C4orf19 | 168 |
| 2 | 10190499 | 10191558 | KLF11 | 168 |
| 1 | 6296100 | 6296863 | ICMT | 168 |
| 19 | 16481825 | 16482936 | EPS15L1 | 168 |
| 1 | 89413301 | 89413933 | CCBL2 | 168 |
| 10 | 104375801 | 104376484 | SUFU | 168 |
| 1 | 26185358 | 26186196 | C1orf135 | 168 |
| 19 | 5120487 | 5122422 | KDM4B | 168 |
| 13 | 42524413 | 42524982 | KIAA0564 | 168 |
| 1 | 33832470 | 33834008 | PHC2 | 167 |
| 5 | 30693813 | 30694349 | CDH6 | 167 |
| 21 | 47305608 | 47306598 | PCBP3 | 167 |
| 15 | 94613322 | 94614193 | MCTP2 | 167 |
| 10 | 43782410 | 43783013 | RASGEF1A | 167 |
| 15 | 70538097 | 70539262 | TLE3 | 167 |
| 18 | 61034451 | 61035501 | KDSR | 167 |
| 18 | 9198370 | 9198817 | ANKRD12 | 167 |
| 1 | 46712301 | 46712361 | RAD54L | 167 |
| 9 | 93737827 | 93738837 | SYK | 167 |
| 3 | 193359432 | 193360135 | OPA1 | 167 |
| 22 | 37880288 | 37881374 | MFNG | 167 |
| 8 | 50370726 | 50371306 | C8orf22 | 167 |
| 4 | 2574691 | 2575597 | FAM193A | 167 |
| 11 | 77368543 | 77369114 | CLNS1A | 167 |
| 19 | 54713164 | 54713972 | MBOAT7 | 167 |
| 8 | 103405479 | 103406654 | UBR5 | 167 |
| 12 | 13310438 | 13311153 | EMP1 | 167 |
| 12 | 1264221 | 1265091 | ERC1 | 167 |
| 1 | 108836713 | 108837296 | NBPF4 | 167 |
| 3 | 13689610 | 13690290 | FBLN2 | 167 |
| 3 | 84190301 | 84190822 | CADM2 | 167 |
| 20 | 61302833 | 61303651 | SLCO4A1 | 166 |
| 5 | 86475942 | 86476784 | RASA1 | 166 |
| 11 | 107713032 | 107713487 | SLC35F2 | 166 |
| 2 | 7147692 | 7149120 | RNF144A | 166 |
| 2 | 99797085 | 99798065 | MITD1 | 166 |
| 3 | 45017734 | 45018727 | ZDHHC3 | 166 |
| 10 | 107993967 | 107994358 | SORCS1 | 166 |
| 2 | 43440751 | 43441796 | ZFP36L2 | 166 |
| 5 | 149828710 | 149830184 | RPS14 | 166 |
| 8 | 125667054 | 125667802 | MTSS1 | 166 |
| 1 | 39490873 | 39491459 | NDUFS5 | 166 |
| 3 | 115912991 | 115913629 | LSAMP | 166 |
| 10 | 97369526 | 97369881 | ALDH18A1 | 166 |
| 10 | 51754379 | 51754779 | AGAP6 | 166 |
| 9 | 38672254 | 38673026 | ANKRD18A | 166 |
| 10 | 74076381 | 74077410 | DNAJB12 | 166 |
| 6 | 159459118 | 159459773 | TAGAP | 166 |
| 5 | 114193364 | 114193962 | TRIM36 | 166 |
| 17 | 73450865 | 73450887 | KIAA0195 | 166 |
| 19 | 58977332 | 58979416 | ZNF324 | 166 |
| 15 | 70717902 | 70718282 | UACA | 166 |
| 1 | 41519353 | 41519883 | SCMH1 | 166 |
| 10 | 116443192 | 116443733 | ABLIM1 | 166 |
| 1 | 203144645 | 203145637 | MYBPH | 166 |
| 15 | 44858871 | 44859166 | SPG11 | 166 |
| 15 | 44858871 | 44859166 | EIF3J | 166 |
| 16 | 377894 | 378198 | AXIN1 | 166 |
| 5 | 69209478 | 69211911 | SERF1A | 166 |
| 13 | 73642491 | 73642988 | KLF5 | 166 |
| 7 | 20842466 | 20843324 | SP8 | 166 |
| 10 | 112734656 | 112735277 | SHOC2 | 166 |
| 7 | 26548430 | 26548613 | C7orf71 | 166 |
| 8 | 3178039 | 3178256 | CSMD1 | 166 |
| 20 | 44601446 | 44601619 | ZNF335 | 166 |
| 12 | 57913276 | 57914124 | MARS | 166 |
| 12 | 57913276 | 57914124 | DDIT3 | 166 |
| 17 | 80527647 | 80528491 | FOXK2 | 166 |
| 15 | 23692335 | 23692967 | MKRN3 | 166 |
| 1 | 17508847 | 17509455 | PADI1 | 165 |
| 7 | 72930654 | 72931533 | BAZ1B | 165 |
| 7 | 26239103 | 26241527 | CBX3 | 165 |
| 6 | 114032609 | 114033484 | MARCKS | 165 |
| 2 | 92026847 | 92027536 | RPIA | 165 |
| 14 | 102938712 | 102939621 | TECPR2 | 165 |
| 4 | 16205759 | 16206532 | TAPT1 | 165 |
| 7 | 117163253 | 117163857 | CFTR | 165 |
| 10 | 32612688 | 32613846 | EPC1 | 165 |
| 7 | 72395268 | 72396062 | POM121 | 165 |
| 8 | 18695741 | 18696426 | PSD3 | 165 |
| 6 | 36878608 | 36879375 | C6orf89 | 165 |
| 6 | 134497122 | 134498363 | SGK1 | 165 |
| 7 | 19969085 | 19969956 | TMEM196 | 165 |
| 13 | 86251611 | 86252297 | SLITRK6 | 165 |
| 10 | 126373670 | 126374497 | FAM53B | 165 |
| 13 | 80852303 | 80853448 | SPRY2 | 165 |
| 16 | 81358002 | 81358818 | GAN | 165 |
| 10 | 11380039 | 11380832 | CELF2 | 165 |
| 10 | 80886730 | 80888054 | ZMIZ1 | 165 |
| 18 | 56095636 | 56096010 | ALPK2 | 165 |
| 2 | 80092692 | 80093270 | CTNNA2 | 165 |
| 12 | 124875024 | 124876088 | NCOR2 | 165 |
| 7 | 90057250 | 90057958 | CLDN12 | 165 |
| 2 | 66672207 | 66674268 | MEIS1 | 165 |
| 8 | 95872349 | 95874712 | INTS8 | 164 |
| 15 | 72214146 | 72214737 | MYO9A | 164 |
| 8 | 67837071 | 67838887 | C8orf45 | 164 |
| 1 | 234743950 | 234744653 | IRF2BP2 | 164 |
| 3 | 48555786 | 48557212 | PFKFB4 | 164 |
| 5 | 36876949 | 36877787 | NIPBL | 164 |
| 2 | 109855056 | 109855409 | SH3RF3 | 164 |
| 11 | 129863924 | 129865114 | PRDM10 | 164 |
| 15 | 74838195 | 74838901 | ARID3B | 164 |
| 3 | 23923728 | 23924195 | UBE2E1 | 164 |
| 2 | 100041938 | 100042891 | REV1 | 164 |
| 7 | 50348094 | 50348904 | IKZF1 | 164 |
| 7 | 138915220 | 138916290 | UBN2 | 164 |
| 22 | 22294573 | 22294646 | PPM1F | 164 |
| 1 | 8033481 | 8034261 | PARK7 | 164 |
| 13 | 20160941 | 20161362 | MPHOSPH8 | 164 |
| 19 | 12942978 | 12943954 | RTBDN | 164 |
| 7 | 24267223 | 24267835 | NPY | 164 |
| 3 | 183272452 | 183273628 | KLHL6 | 164 |
| 7 | 41740516 | 41741327 | INHBA | 164 |
| 17 | 9135798 | 9136966 | NTN1 | 164 |
| 6 | 44220025 | 44221044 | HSP90AB1 | 164 |
| 2 | 46160282 | 46161038 | PRKCE | 164 |
| 16 | 86410895 | 86413493 | FOXF1 | 164 |
| 19 | 14319185 | 14319249 | LPHN1 | 164 |
| 1 | 22107043 | 22108238 | USP48 | 164 |
| 2 | 99081351 | 99081903 | INPP4A | 164 |
| 10 | 76796495 | 76797359 | DUPD1 | 164 |
| 14 | 57116765 | 57117221 | C14orf101 | 163 |
| 20 | 5930795 | 5931638 | MCM8 | 163 |
| 5 | 139314213 | 139314780 | NRG2 | 163 |
| 18 | 60649674 | 60650105 | PHLPP1 | 163 |
| 15 | 78348511 | 78349837 | TBC1D2B | 163 |
| 21 | 45615759 | 45618322 | ICOSLG | 163 |
| 13 | 85594297 | 85594623 | SLITRK6 | 163 |
| 10 | 76702155 | 76702570 | KAT6B | 163 |
| 4 | 88131232 | 88131582 | KLHL8 | 163 |
| 3 | 14611520 | 14612075 | GRIP2 | 163 |
| 6 | 106659022 | 106659521 | ATG5 | 163 |
| 11 | 61600761 | 61602470 | FADS2 | 163 |
| 10 | 47042784 | 47044774 | PPYR1 | 163 |
| 2 | 97698330 | 97699081 | FAM178B | 163 |
| 18 | 70901532 | 70902145 | NETO1 | 163 |
| 10 | 102130594 | 102131468 | SCD | 163 |
| 7 | 2610802 | 2611433 | IQCE | 163 |
| 2 | 25798846 | 25799652 | DTNB | 163 |
| 13 | 74666206 | 74666528 | KLF12 | 163 |
| 6 | 105270025 | 105270691 | HACE1 | 163 |
| 11 | 62358500 | 62359942 | TUT1 | 163 |
| 19 | 54617234 | 54620087 | PRPF31 | 163 |
| 19 | 54617234 | 54620087 | TFPT | 163 |
| 16 | 4576696 | 4577304 | C16orf5 | 163 |
| 19 | 42748675 | 42749536 | DEDD2 | 163 |
| 3 | 32619817 | 32620002 | DYNC1LI1 | 163 |
| 16 | 57723549 | 57723999 | CCDC135 | 163 |
| 5 | 172750889 | 172751458 | STC2 | 163 |
| 5 | 176927649 | 176928753 | DBN1 | 163 |
| 21 | 34959966 | 34961534 | DONSON | 163 |
| 3 | 13343607 | 13343626 | NUP210 | 163 |
| 2 | 63241691 | 63242195 | EHBP1 | 163 |
| X | 74375714 | 74376213 | ABCB7 | 163 |
| 15 | 32934311 | 32934454 | SCG5 | 162 |
| 1 | 90479213 | 90479762 | ZNF326 | 162 |
| 19 | 56630268 | 56631668 | ZNF787 | 162 |
| 19 | 48751973 | 48752597 | CARD8 | 162 |
| 1 | 103471941 | 103472515 | COL11A1 | 162 |
| 10 | 24846199 | 24846304 | ARHGAP21 | 162 |
| 5 | 42720854 | 42721415 | GHR | 162 |
| 11 | 60291989 | 60292580 | MS4A13 | 162 |
| 22 | 39715909 | 39716270 | RPL3 | 162 |
| 16 | 74484653 | 74485632 | GLG1 | 162 |
| 18 | 77545809 | 77546466 | KCNG2 | 162 |
| 3 | 197484537 | 197485240 | FYTTD1 | 162 |
| 6 | 50931191 | 50931692 | TFAP2B | 162 |
| 13 | 113916743 | 113917598 | CUL4A | 162 |
| 4 | 3572522 | 3572935 | LRPAP1 | 162 |
| 12 | 40169583 | 40170369 | SLC2A13 | 162 |
| 5 | 118236044 | 118236761 | DTWD2 | 162 |
| 9 | 134131596 | 134132505 | FAM78A | 162 |
| 7 | 20747248 | 20747857 | ABCB5 | 162 |
| 9 | 46887667 | 46888496 | CNTNAP3B | 162 |
| 1 | 110949658 | 110950642 | HBXIP | 162 |
| 8 | 101962391 | 101963420 | YWHAZ | 162 |
| 11 | 46614967 | 46615729 | ATG13 | 162 |
| 7 | 66740031 | 66740797 | TYW1 | 162 |
| 13 | 91971994 | 91972787 | GPC5 | 162 |
| 6 | 125874671 | 125875148 | HEY2 | 162 |
| 5 | 86563091 | 86564633 | RASA1 | 162 |
| 19 | 11242885 | 11244143 | LDLR | 162 |
| 6 | 63963598 | 63963996 | LGSN | 162 |
| 1 | 108812517 | 108813061 | NBPF4 | 162 |
| 4 | 7582979 | 7583736 | SORCS2 | 162 |
| 15 | 41194184 | 41195722 | VPS18 | 162 |
| 7 | 53246766 | 53247353 | POM121L12 | 162 |
| 4 | 9971952 | 9972607 | SLC2A9 | 162 |
| 5 | 32312042 | 32312047 | MTMR12 | 161 |
| 3 | 151788357 | 151788960 | MBNL1 | 161 |
| 3 | 112370998 | 112371528 | CCDC80 | 161 |
| 15 | 44350562 | 44351108 | FRMD5 | 161 |
| 6 | 27113932 | 27116023 | HIST1H2BK | 161 |
| 3 | 53656764 | 53657377 | CACNA1D | 161 |
| 17 | 66470358 | 66471182 | WIPI1 | 161 |
| 6 | 158186206 | 158186830 | SNX9 | 161 |
| 11 | 57508487 | 57509964 | C11orf31 | 161 |
| 8 | 103119798 | 103120465 | NCALD | 161 |
| 18 | 59853054 | 59854255 | KIAA1468 | 161 |
| 8 | 71550836 | 71551607 | LACTB2 | 161 |
| 15 | 73945632 | 73946157 | NPTN | 161 |
| 18 | 34642319 | 34642892 | KIAA1328 | 161 |
| 12 | 39062235 | 39063166 | CPNE8 | 161 |
| 4 | 26274762 | 26275547 | RBPJ | 161 |
| 20 | 1446695 | 1447825 | NSFL1C | 161 |
| 6 | 37017851 | 37020453 | FGD2 | 161 |
| 11 | 65407105 | 65408942 | SIPA1 | 161 |
| 19 | 45921860 | 45923235 | ERCC1 | 161 |
| 10 | 82251350 | 82252255 | TSPAN14 | 161 |
| 12 | 116985001 | 116985607 | MAP1LC3B2 | 161 |
| 3 | 47249653 | 47250295 | SETD2 | 161 |
| 1 | 179993761 | 179994368 | CEP350 | 161 |
| 6 | 76313971 | 76314764 | SENP6 | 161 |
| 2 | 109335117 | 109336419 | RANBP2 | 161 |
| 14 | 23563623 | 23564998 | C14orf119 | 161 |
| 14 | 23563623 | 23564998 | ACIN1 | 161 |
| 1 | 25161559 | 25162143 | CLIC4 | 161 |
| 3 | 53202867 | 53205055 | PRKCD | 161 |
| 2 | 242908167 | 242908578 | C2orf85 | 161 |
| 10 | 72067454 | 72068656 | LRRC20 | 161 |
| 3 | 185122351 | 185122764 | MAP3K13 | 161 |
| 13 | 92728012 | 92728709 | GPC5 | 161 |
| 4 | 124319743 | 124320890 | SPRY1 | 161 |
| 4 | 15945177 | 15945846 | FGFBP1 | 161 |
| 11 | 61791747 | 61792484 | FTH1 | 161 |
| 11 | 117614822 | 117615854 | DSCAML1 | 161 |
| X | 152848325 | 152848815 | FAM58A | 160 |
| 1 | 44166437 | 44167157 | KDM4A | 160 |
| 3 | 151588530 | 151588966 | SUCNR1 | 160 |
| 1 | 144921058 | 144921628 | PDE4DIP | 160 |
| 7 | 139726649 | 139727179 | PARP12 | 160 |
| 21 | 40376465 | 40376991 | PSMG1 | 160 |
| 21 | 27106869 | 27107910 | ATP5J | 160 |
| 5 | 145875246 | 145875776 | TCERG1 | 160 |
| 21 | 27106869 | 27107910 | GABPA | 160 |
| 2 | 42315865 | 42316934 | PKDCC | 160 |
| 1 | 120604142 | 120604773 | NOTCH2 | 160 |
| 14 | 80656820 | 80657439 | DIO2 | 160 |
| 5 | 87528261 | 87528738 | TMEM161B | 160 |
| 12 | 2030798 | 2030803 | CACNA2D4 | 160 |
| 1 | 186948952 | 186949493 | PLA2G4A | 160 |
| 5 | 96270168 | 96272042 | LNPEP | 160 |
| 11 | 18547956 | 18548758 | TSG101 | 160 |
| 22 | 25784477 | 25785538 | LRP5L | 160 |
| 10 | 47289467 | 47290096 | AGAP9 | 160 |
| 8 | 106026406 | 106026895 | ZFPM2 | 160 |
| 12 | 8014340 | 8015355 | SLC2A14 | 160 |
| 11 | 86777609 | 86778131 | TMEM135 | 160 |
| 5 | 171606171 | 171607499 | STK10 | 160 |
| 10 | 88863560 | 88864374 | FAM35A | 160 |
| 2 | 162137257 | 162139410 | PSMD14 | 160 |
| 1 | 17342934 | 17343929 | ATP13A2 | 160 |
| 12 | 58267803 | 58267814 | CTDSP2 | 160 |
| 17 | 18965189 | 18967376 | GRAP | 160 |
| 9 | 77634948 | 77635655 | C9orf41 | 160 |
| 1 | 19252963 | 19254415 | IFFO2 | 160 |
| 9 | 130341000 | 130342006 | FAM129B | 160 |
| 1 | 159880007 | 159880491 | CCDC19 | 160 |
| 14 | 67721215 | 67722173 | MPP5 | 160 |
| 6 | 38651117 | 38652002 | GLO1 | 160 |
| 13 | 99128445 | 99129292 | STK24 | 160 |
| 11 | 67043282 | 67046320 | ADRBK1 | 159 |
| 7 | 101033854 | 101034910 | EMID2 | 159 |
| 1 | 39491472 | 39493208 | NDUFS5 | 159 |
| 19 | 32895511 | 32896783 | DPY19L3 | 159 |
| 18 | 20429261 | 20429833 | RBBP8 | 159 |
| 5 | 112405093 | 112405645 | MCC | 159 |
| 7 | 13399085 | 13399674 | ETV1 | 159 |
| 5 | 122116011 | 122116647 | SNX2 | 159 |
| 6 | 65361117 | 65361692 | EYS | 159 |
| 7 | 64126141 | 64127150 | ZNF107 | 159 |
| 14 | 50329070 | 50329932 | NEMF | 159 |
| 3 | 5060941 | 5061840 | BHLHE40 | 159 |
| 3 | 140416227 | 140416847 | TRIM42 | 159 |
| 10 | 95461836 | 95462677 | FRA10AC1 | 159 |
| 6 | 149337557 | 149338604 | UST | 159 |
| 5 | 123992781 | 123993423 | ZNF608 | 159 |
| 1 | 114273989 | 114274555 | PHTF1 | 159 |
| 1 | 6087947 | 6088854 | KCNAB2 | 159 |
| 3 | 32442528 | 32444247 | CMTM7 | 159 |
| 18 | 43227926 | 43228669 | SLC14A2 | 159 |
| 8 | 142202178 | 142203050 | DENND3 | 159 |
| 20 | 23132152 | 23133461 | CD93 | 159 |
| 1 | 11874435 | 11875446 | CLCN6 | 159 |
| 12 | 42633047 | 42634177 | YAF2 | 159 |
| 1 | 144991063 | 144991683 | PDE4DIP | 159 |
| 6 | 26233113 | 26235154 | HIST1H2BH | 159 |
| X | 71320689 | 71321478 | NHSL2 | 159 |
| 16 | 87099859 | 87102673 | C16orf95 | 159 |
| 2 | 1994130 | 1994787 | MYT1L | 158 |
| 22 | 24541548 | 24541811 | CABIN1 | 158 |
| 1 | 41706622 | 41707346 | SCMH1 | 158 |
| 16 | 84095438 | 84096900 | MBTPS1 | 158 |
| 13 | 77929703 | 77930232 | MYCBP2 | 158 |
| 5 | 162865501 | 162866280 | CCNG1 | 158 |
| 18 | 77579237 | 77580422 | KCNG2 | 158 |
| 4 | 2414424 | 2415765 | ZFYVE28 | 158 |
| 10 | 120608235 | 120608692 | C10orf46 | 158 |
| 6 | 27859455 | 27862335 | HIST1H2BO | 158 |
| 15 | 50554182 | 50556077 | HDC | 158 |
| 4 | 8036786 | 8038247 | ABLIM2 | 158 |
| 18 | 21843441 | 21844003 | OSBPL1A | 158 |
| 15 | 85523127 | 85525200 | PDE8A | 158 |
| 17 | 61818472 | 61820330 | STRADA | 158 |
| 14 | 35591001 | 35591799 | KIAA0391 | 158 |
| 13 | 76593368 | 76594459 | LMO7 | 158 |
| 12 | 117462667 | 117463382 | FBXW8 | 158 |
| 10 | 22614639 | 22615000 | BMI1 | 158 |
| 10 | 22614639 | 22615000 | COMMD3-BMI1 | 158 |
| 5 | 110478223 | 110478579 | WDR36 | 158 |
| 14 | 91113492 | 91113953 | TTC7B | 158 |
| 6 | 133134713 | 133136029 | RPS12 | 158 |
| 6 | 103224640 | 103225106 | GRIK2 | 158 |
| 7 | 158325411 | 158325723 | PTPRN2 | 158 |
| 2 | 101924363 | 101925837 | RNF149 | 158 |
| 10 | 98518449 | 98519021 | PIK3AP1 | 158 |
| 4 | 41047105 | 41047621 | APBB2 | 158 |
| 6 | 148665717 | 148666280 | SASH1 | 158 |
| 6 | 167017731 | 167018315 | RPS6KA2 | 158 |
| 11 | 9385568 | 9386352 | IPO7 | 158 |
| 1 | 28973795 | 28975007 | GMEB1 | 158 |
| 1 | 101227197 | 101227816 | VCAM1 | 158 |
| 2 | 55214999 | 55215499 | RTN4 | 158 |
| 11 | 116724672 | 116725126 | SIK3 | 157 |
| 8 | 7227388 | 7228009 | ZNF705G | 157 |
| 4 | 16802068 | 16802700 | LDB2 | 157 |
| 3 | 142768778 | 142769361 | U2SURP | 157 |
| 8 | 17288129 | 17288815 | MTMR7 | 157 |
| 5 | 21157373 | 21158011 | CDH18 | 157 |
| 16 | 17087438 | 17087649 | XYLT1 | 157 |
| 1 | 54963140 | 54963831 | ACOT11 | 157 |
| 8 | 96190531 | 96191139 | PLEKHF2 | 157 |
| 10 | 99077086 | 99078538 | FRAT1 | 157 |
| 20 | 45203984 | 45204520 | SLC13A3 | 157 |
| 7 | 155382817 | 155384951 | RBM33 | 157 |
| 1 | 62233735 | 62234385 | INADL | 157 |
| 1 | 114302843 | 114303587 | PHTF1 | 157 |
| 4 | 4319297 | 4320155 | ZBTB49 | 157 |
| 3 | 113233347 | 113234911 | SPICE1 | 157 |
| 15 | 69605509 | 69606406 | PAQR5 | 157 |
| 21 | 48116790 | 48117734 | PRMT2 | 157 |
| 13 | 114830227 | 114831627 | RASA3 | 157 |
| 3 | 19942930 | 19943380 | EFHB | 157 |
| 7 | 5762845 | 5763515 | RNF216 | 157 |
| 1 | 27958138 | 27960034 | FGR | 157 |
| 2 | 87871815 | 87872894 | PLGLB2 | 157 |
| 16 | 3206760 | 3207079 | ZNF213 | 157 |
| 16 | 85137959 | 85139187 | FAM92B | 157 |
| 3 | 105239694 | 105240244 | ALCAM | 157 |
| 9 | 140214829 | 140215858 | EXD3 | 156 |
| 13 | 86153462 | 86155341 | SLITRK6 | 156 |
| 5 | 69751791 | 69752257 | SMN1 | 156 |
| 2 | 43450977 | 43453585 | ZFP36L2 | 156 |
| 11 | 61723059 | 61723098 | BEST1 | 156 |
| 9 | 33819511 | 33820088 | UBE2R2 | 156 |
| 19 | 46293966 | 46294284 | DMWD | 156 |
| 5 | 18902535 | 18903158 | CDH18 | 156 |
| 3 | 113296664 | 113297407 | SIDT1 | 156 |
| 10 | 111887961 | 111888411 | ADD3 | 156 |
| 4 | 25270739 | 25271777 | PI4K2B | 156 |
| 3 | 185353091 | 185354943 | SENP2 | 156 |
| 11 | 68146420 | 68149784 | LRP5 | 156 |
| 3 | 162358446 | 162358470 | OTOL1 | 156 |
| 7 | 96746605 | 96747243 | ACN9 | 156 |
| 7 | 6213418 | 6214566 | CYTH3 | 156 |
| 4 | 6157563 | 6158170 | JAKMIP1 | 156 |
| 22 | 32061610 | 32061638 | PISD | 156 |
| 1 | 151429945 | 151430555 | POGZ | 156 |
| 10 | 126744912 | 126745002 | CTBP2 | 156 |
| 3 | 194674725 | 194675977 | FAM43A | 156 |
| 19 | 45170046 | 45170071 | CEACAM19 | 156 |
| 3 | 193408481 | 193409108 | OPA1 | 156 |
| 19 | 41814037 | 41814304 | CCDC97 | 156 |
| 11 | 3857772 | 3858657 | RHOG | 156 |
| 3 | 14472709 | 14474589 | SLC6A6 | 156 |
| 1 | 206753121 | 206753560 | RASSF5 | 156 |
| 1 | 105064521 | 105064906 | AMY1B | 156 |
| 7 | 129826508 | 129827121 | TMEM209 | 156 |
| 1 | 23874732 | 23875635 | E2F2 | 155 |
| 1 | 51863584 | 51864192 | EPS15 | 155 |
| 20 | 22030744 | 22031522 | PAX1 | 155 |
| 8 | 102255617 | 102256457 | ZNF706 | 155 |
| 17 | 5240486 | 5241041 | RABEP1 | 155 |
| 5 | 171399324 | 171399776 | FBXW11 | 155 |
| 6 | 168099180 | 168100690 | MLLT4 | 155 |
| 2 | 10466266 | 10466289 | HPCAL1 | 155 |
| 6 | 167197044 | 167197744 | RPS6KA2 | 155 |
| 8 | 108995029 | 108995452 | RSPO2 | 155 |
| 6 | 25668790 | 25669466 | SCGN | 155 |
| 13 | 75584933 | 75585464 | TBC1D4 | 155 |
| 13 | 114821056 | 114822946 | RASA3 | 155 |
| 2 | 87798151 | 87798584 | PLGLB2 | 155 |
| 6 | 134338448 | 134339109 | SLC2A12 | 155 |
| X | 48794280 | 48794628 | OTUD5 | 155 |
| 8 | 61429226 | 61430227 | RAB2A | 155 |
| 7 | 64829304 | 64830321 | ZNF92 | 155 |
| 17 | 16118163 | 16119532 | PIGL | 155 |
| 1 | 225964740 | 225966020 | SRP9 | 155 |
| 5 | 40460110 | 40460655 | PTGER4 | 155 |
| 7 | 10979268 | 10980420 | NDUFA4 | 155 |
| 12 | 38758471 | 38759149 | ALG10B | 155 |
| 11 | 105157925 | 105158367 | CARD18 | 155 |
| 13 | 87687994 | 87689661 | SLITRK5 | 155 |
| 10 | 95665892 | 95666197 | SLC35G1 | 155 |
| 15 | 102076678 | 102077776 | PCSK6 | 155 |
| 6 | 170687395 | 170688233 | FAM120B | 155 |
| 12 | 12877501 | 12878531 | APOLD1 | 155 |
| 1 | 52955677 | 52956091 | ZCCHC11 | 155 |
| 2 | 114361239 | 114362109 | RABL2A | 155 |
| 1 | 17230087 | 17231768 | CROCC | 155 |
| 6 | 14840394 | 14840980 | JARID2 | 155 |
| 19 | 42701378 | 42704622 | DEDD2 | 155 |
| 1 | 36839179 | 36840561 | STK40 | 155 |
| 3 | 185401805 | 185403080 | IGF2BP2 | 155 |
| 6 | 86387954 | 86389215 | SYNCRIP | 155 |
| 1 | 245649896 | 245650238 | KIF26B | 155 |
| 15 | 43158940 | 43159560 | TTBK2 | 155 |
| 5 | 79940437 | 79940877 | MSH3 | 155 |
| 5 | 79940437 | 79940877 | DHFR | 155 |
| 2 | 87839658 | 87839893 | PLGLB2 | 155 |
| 5 | 146225715 | 146226348 | PPP2R2B | 155 |
| 4 | 154496792 | 154497568 | KIAA0922 | 155 |
| 4 | 43542069 | 43542470 | GRXCR1 | 155 |
| 17 | 5322235 | 5323875 | NUP88 | 154 |
| 17 | 5322235 | 5323875 | RPAIN | 154 |
| 15 | 32902374 | 32902965 | ARHGAP11A | 154 |
| 4 | 39047920 | 39048304 | KLHL5 | 154 |
| 17 | 47816834 | 47817619 | FAM117A | 154 |
| 14 | 106061279 | 106062226 | TMEM121 | 154 |
| 7 | 27633410 | 27634123 | HIBADH | 154 |
| 12 | 31812512 | 31813188 | METTL20 | 154 |
| 17 | 16767367 | 16768605 | TNFRSF13B | 154 |
| 2 | 100663343 | 100663845 | AFF3 | 154 |
| 12 | 124917590 | 124917695 | NCOR2 | 154 |
| 6 | 23385466 | 23386077 | NRSN1 | 154 |
| 7 | 74587191 | 74588150 | GTF2IRD2B | 154 |
| 15 | 91525408 | 91526013 | PRC1 | 154 |
| 2 | 25966604 | 25966964 | ASXL2 | 154 |
| 6 | 33660199 | 33661378 | ITPR3 | 154 |
| 15 | 45763998 | 45764513 | C15orf48 | 154 |
| 19 | 48624930 | 48626054 | LIG1 | 154 |
| 7 | 80019241 | 80019882 | GNAT3 | 154 |
| 7 | 125888072 | 125888720 | GRM8 | 154 |
| 15 | 93432214 | 93432825 | CHD2 | 154 |
| 4 | 2789128 | 2790264 | SH3BP2 | 154 |
| 7 | 90016237 | 90016768 | GTPBP10 | 154 |
| X | 9501638 | 9502240 | TBL1X | 154 |
| 20 | 56323659 | 56324778 | PMEPA1 | 154 |
| 13 | 86334232 | 86335304 | SLITRK6 | 154 |
| 14 | 83751342 | 83751913 | SEL1L | 154 |
| 3 | 194672744 | 194673265 | FAM43A | 154 |
| 1 | 245473765 | 245474210 | KIF26B | 154 |
| 8 | 21621860 | 21622308 | GFRA2 | 154 |
| 22 | 37702773 | 37703733 | CYTH4 | 154 |
| 9 | 18911222 | 18912097 | ADAMTSL1 | 154 |
| 13 | 84660008 | 84660567 | SLITRK1 | 154 |
| 6 | 41623299 | 41624518 | MDFI | 154 |
| 1 | 55180885 | 55180896 | TTC4 | 154 |
| 3 | 28282540 | 28283671 | CMC1 | 154 |
| 7 | 43479168 | 43479980 | HECW1 | 154 |
| 12 | 95397042 | 95398013 | NDUFA12 | 154 |
| 17 | 10017509 | 10017936 | GAS7 | 154 |
| 4 | 27942123 | 27942713 | STIM2 | 154 |
| 10 | 74069292 | 74069378 | DDIT4 | 153 |
| 4 | 34144343 | 34144834 | ARAP2 | 153 |
| 4 | 38083515 | 38084577 | TBC1D1 | 153 |
| 11 | 116951903 | 116952484 | SIK3 | 153 |
| 3 | 156336483 | 156337077 | TIPARP | 153 |
| 17 | 42218541 | 42218690 | C17orf53 | 153 |
| 20 | 1837618 | 1838394 | SIRPA | 153 |
| 7 | 121620746 | 121621280 | PTPRZ1 | 153 |
| 16 | 86350559 | 86351727 | FOXF1 | 153 |
| 22 | 47069749 | 47070960 | GRAMD4 | 153 |
| 2 | 43385117 | 43386435 | ZFP36L2 | 153 |
| 2 | 58272790 | 58274728 | VRK2 | 153 |
| 11 | 61211666 | 61212310 | SDHAF2 | 153 |
| 3 | 194676064 | 194677583 | FAM43A | 153 |
| 3 | 194040198 | 194042079 | CPN2 | 153 |
| 3 | 32543582 | 32544337 | CMTM6 | 153 |
| 15 | 75960487 | 75961370 | IMP3 | 153 |
| 3 | 196386712 | 196388666 | LRRC33 | 153 |
| 7 | 47615783 | 47618345 | TNS3 | 153 |
| 9 | 42952351 | 42952919 | ANKRD20A3 | 153 |
| 4 | 49228220 | 49229119 | CWH43 | 153 |
| 3 | 190958569 | 190959466 | OSTN | 153 |
| 3 | 48020908 | 48021586 | MAP4 | 153 |
| 20 | 23064388 | 23069339 | CD93 | 153 |
| 7 | 6235468 | 6235476 | CYTH3 | 153 |
| 4 | 122722162 | 122722870 | EXOSC9 | 153 |
| 8 | 61824127 | 61824690 | CHD7 | 153 |
| 12 | 41871598 | 41872351 | PDZRN4 | 153 |
| 15 | 83208663 | 83209433 | RPS17 | 153 |
| 8 | 17041675 | 17041929 | ZDHHC2 | 153 |
| 17 | 29870882 | 29870957 | RAB11FIP4 | 152 |
| 15 | 56485069 | 56485725 | RFX7 | 152 |
| 20 | 45173886 | 45174454 | C20orf123 | 152 |
| 20 | 57225628 | 57226868 | STX16 | 152 |
| 7 | 23386014 | 23386510 | IGF2BP3 | 152 |
| 4 | 46533923 | 46534496 | GABRA2 | 152 |
| 9 | 132539106 | 132540666 | TOR1B | 152 |
| 1 | 17757639 | 17757923 | RCC2 | 152 |
| 19 | 16705545 | 16705557 | MED26 | 152 |
| 10 | 74056036 | 74057604 | DDIT4 | 152 |
| 22 | 37696348 | 37697142 | CYTH4 | 152 |
| 3 | 195589101 | 195593643 | TNK2 | 152 |
| 8 | 56917709 | 56918185 | LYN | 152 |
| 4 | 115068289 | 115068958 | ARSJ | 152 |
| 3 | 53380980 | 53382378 | DCP1A | 152 |
| 3 | 113142798 | 113143217 | WDR52 | 152 |
| 12 | 116723876 | 116724536 | MED13L | 152 |
| 1 | 36803268 | 36803685 | FAM176B | 152 |
| 2 | 61405810 | 61406429 | AHSA2 | 152 |
| 3 | 113911696 | 113912290 | DRD3 | 151 |
| 4 | 178363084 | 178363843 | AGA | 151 |
| 19 | 1253234 | 1253525 | MIDN | 151 |
| 7 | 98270656 | 98271539 | NPTX2 | 151 |
| 5 | 140893132 | 140894252 | PCDHGB5 | 151 |
| 5 | 140893132 | 140894252 | PCDHGB1 | 151 |
| 5 | 140893132 | 140894252 | PCDHGA8 | 151 |
| 5 | 140893132 | 140894252 | PCDHGA12 | 151 |
| 5 | 140893132 | 140894252 | PCDHGA11 | 151 |
| 5 | 140893132 | 140894252 | PCDHGA10 | 151 |
| 5 | 140893132 | 140894252 | PCDHGC3 | 151 |
| 5 | 140893132 | 140894252 | PCDHGA6 | 151 |
| 5 | 140893132 | 140894252 | PCDHGA4 | 151 |
| 5 | 140893132 | 140894252 | PCDHGB6 | 151 |
| 5 | 140893132 | 140894252 | PCDHGB2 | 151 |
| 5 | 140893132 | 140894252 | PCDHGA1 | 151 |
| 5 | 140893132 | 140894252 | PCDHGC5 | 151 |
| 5 | 140893132 | 140894252 | PCDHGA7 | 151 |
| 5 | 140893132 | 140894252 | PCDHGB7 | 151 |
| 5 | 140893132 | 140894252 | PCDHGA9 | 151 |
| 5 | 140893132 | 140894252 | PCDHGA3 | 151 |
| 5 | 140893132 | 140894252 | PCDHGB3 | 151 |
| 5 | 140893132 | 140894252 | PCDHGA2 | 151 |
| 5 | 140893132 | 140894252 | PCDHGA5 | 151 |
| 5 | 140893132 | 140894252 | PCDHGB4 | 151 |
| 5 | 140893132 | 140894252 | PCDHGC4 | 151 |
| 17 | 55945076 | 55946961 | CUEDC1 | 151 |
| 14 | 58711111 | 58711991 | PSMA3 | 151 |
| 19 | 46010228 | 46010996 | VASP | 151 |
| 17 | 15830943 | 15831396 | ADORA2B | 151 |
| 6 | 170451792 | 170454244 | DLL1 | 151 |
| 1 | 17758747 | 17758880 | RCC2 | 151 |
| 5 | 78725370 | 78725976 | HOMER1 | 151 |
| 7 | 43908895 | 43909618 | MRPS24 | 151 |
| 7 | 43908895 | 43909618 | URGCP-MRPS24 | 151 |
| 2 | 32533134 | 32534174 | YIPF4 | 151 |
| 16 | 12416673 | 12417124 | SNX29 | 151 |
| 3 | 134162006 | 134162624 | CEP63 | 151 |
| 2 | 102381531 | 102382063 | MAP4K4 | 151 |
| 17 | 63158470 | 63158776 | RGS9 | 151 |
| 6 | 159413237 | 159413773 | RSPH3 | 151 |
| 15 | 64033077 | 64033701 | HERC1 | 151 |
| 15 | 26327131 | 26328037 | ATP10A | 151 |
| 11 | 6633386 | 6634040 | TAF10 | 151 |
| 11 | 6633386 | 6634040 | ILK | 151 |
| 12 | 46314497 | 46315290 | SCAF11 | 151 |
| 15 | 90562646 | 90564150 | ZNF710 | 151 |
| 16 | 9156194 | 9156319 | C16orf72 | 151 |
| 5 | 132262131 | 132262700 | AFF4 | 151 |
| 18 | 29449567 | 29450128 | TRAPPC8 | 151 |
| 6 | 26124218 | 26124900 | HIST1H2BC | 151 |
| 10 | 105097802 | 105097927 | PCGF6 | 151 |
| 11 | 77914414 | 77915341 | USP35 | 151 |
| 2 | 11316043 | 11316129 | PQLC3 | 151 |
| 22 | 30674879 | 30675742 | OSM | 151 |
| 1 | 40203549 | 40204459 | PPIE | 151 |
| 21 | 45237189 | 45238083 | RRP1 | 151 |
| 12 | 40492396 | 40493183 | SLC2A13 | 151 |
| 15 | 26052141 | 26052367 | ATP10A | 151 |
| 10 | 70702169 | 70702609 | DDX50 | 151 |
| 1 | 70670844 | 70671818 | SRSF11 | 151 |
| 8 | 48431011 | 48431560 | KIAA0146 | 150 |
| 11 | 130160941 | 130161468 | ZBTB44 | 150 |
| 7 | 127234097 | 127234220 | FSCN3 | 150 |
| 1 | 113556245 | 113557185 | SLC16A1 | 150 |
| 22 | 18633356 | 18633790 | USP18 | 150 |
| 2 | 240189710 | 240190152 | HDAC4 | 150 |
| 4 | 47737491 | 47738065 | CORIN | 150 |
| 6 | 148879390 | 148879935 | UST | 150 |
| 14 | 60049453 | 60050014 | C14orf38 | 150 |
| 13 | 77600134 | 77600873 | FBXL3 | 150 |
| 6 | 26924192 | 26925043 | HIST1H2BJ | 150 |
| 14 | 93126414 | 93127090 | RIN3 | 150 |
| 22 | 44464258 | 44465260 | PARVB | 150 |
| 18 | 73003875 | 73004430 | TSHZ1 | 150 |
| 4 | 102711526 | 102712545 | BANK1 | 150 |
| 3 | 135696382 | 135696927 | PPP2R3A | 150 |
| 17 | 80111205 | 80112641 | CCDC57 | 150 |
| 13 | 87626260 | 87627386 | SLITRK5 | 150 |
| 10 | 94808101 | 94808611 | EXOC6 | 150 |
| 2 | 92203828 | 92205084 | RPIA | 150 |
| 19 | 12792256 | 12793669 | DHPS | 150 |
| 13 | 92437999 | 92438884 | GPC5 | 150 |
| 7 | 143124825 | 143125344 | EPHA1 | 150 |
| 2 | 42105672 | 42106408 | LOC400950 | 150 |
| 13 | 89127106 | 89127863 | SLITRK5 | 150 |
| 17 | 164150 | 166089 | RPH3AL | 150 |
| 17 | 164150 | 166089 | LOC100506388 | 150 |
| 10 | 30818146 | 30818865 | MAP3K8 | 150 |
| 2 | 43453614 | 43455506 | ZFP36L2 | 150 |
| 20 | 23107929 | 23108628 | CD93 | 150 |
| 15 | 56538178 | 56538560 | RFX7 | 150 |
| 17 | 43303226 | 43304082 | FMNL1 | 150 |
| 4 | 24422445 | 24423376 | DHX15 | 150 |
| 9 | 16659751 | 16659840 | BNC2 | 150 |
| 7 | 158325772 | 158326491 | PTPRN2 | 150 |
| 21 | 26979291 | 26980205 | MRPL39 | 150 |
| 1 | 52869444 | 52870676 | PRPF38A | 150 |
| 12 | 46308496 | 46309641 | SCAF11 | 150 |
| 12 | 41241374 | 41241400 | CNTN1 | 150 |
| 2 | 23676798 | 23676911 | KLHL29 | 149 |
| 6 | 44526124 | 44526809 | CDC5L | 149 |
| 10 | 22911239 | 22911694 | PIP4K2A | 149 |
| 13 | 27295296 | 27296000 | GPR12 | 149 |
| 10 | 70228603 | 70229221 | DNA2 | 149 |
| 3 | 156493912 | 156493989 | LEKR1 | 149 |
| 3 | 122831784 | 122832333 | PDIA5 | 149 |
| 2 | 37371537 | 37371970 | EIF2AK2 | 149 |
| 7 | 107383502 | 107385259 | CBLL1 | 149 |
| 7 | 106956797 | 106957353 | COG5 | 149 |
| 4 | 113557699 | 113558829 | LARP7 | 149 |
| 10 | 6452566 | 6453166 | PRKCQ | 149 |
| 15 | 65242339 | 65242610 | ANKDD1A | 149 |
| 7 | 115850999 | 115851630 | TES | 149 |
| 5 | 27929931 | 27930502 | CDH9 | 149 |
| 15 | 63942130 | 63942863 | HERC1 | 149 |
| 13 | 79239489 | 79240569 | RNF219 | 149 |
| 5 | 100377487 | 100378282 | ST8SIA4 | 149 |
| 12 | 132917934 | 132918592 | GALNT9 | 149 |
| 14 | 93707326 | 93707912 | BTBD7 | 149 |
| 6 | 152420964 | 152421484 | ESR1 | 149 |
| 7 | 130692636 | 130693211 | MKLN1 | 149 |
| 2 | 198485297 | 198485763 | RFTN2 | 149 |
| 11 | 85693310 | 85693893 | PICALM | 149 |
| 15 | 31296844 | 31297403 | TRPM1 | 149 |
| 7 | 76339933 | 76340792 | POMZP3 | 148 |
| 2 | 69371093 | 69371652 | ANTXR1 | 148 |
| 8 | 102147450 | 102150408 | ZNF706 | 148 |
| 3 | 179901992 | 179902717 | PEX5L | 148 |
| 4 | 26221078 | 26221791 | RBPJ | 148 |
| 19 | 55737968 | 55738770 | TMEM86B | 148 |
| 2 | 25800296 | 25801005 | DTNB | 148 |
| 2 | 87885222 | 87885688 | PLGLB2 | 148 |
| 8 | 95731515 | 95732581 | DPY19L4 | 148 |
| 2 | 59547325 | 59548013 | FANCL | 148 |
| 5 | 131386156 | 131386640 | CSF2 | 148 |
| 7 | 2288190 | 2289646 | NUDT1 | 148 |
| 1 | 36694476 | 36696337 | THRAP3 | 148 |
| 11 | 69550054 | 69551030 | FGF19 | 148 |
| 6 | 74229731 | 74231595 | EEF1A1 | 148 |
| 2 | 218681051 | 218681623 | TNS1 | 148 |
| 19 | 19572265 | 19572487 | GATAD2A | 148 |
| 13 | 85427588 | 85429317 | SLITRK6 | 148 |
| 15 | 64752362 | 64752862 | ZNF609 | 148 |
| 15 | 85349766 | 85350348 | ALPK3 | 148 |
| 3 | 123364500 | 123365044 | MYLK | 148 |
| 2 | 232409701 | 232410159 | NMUR1 | 148 |
| 16 | 3208921 | 3209694 | ZNF213 | 148 |
| 8 | 13141579 | 13142114 | DLC1 | 148 |
| 3 | 49066311 | 49067127 | IMPDH2 | 148 |
| 5 | 57929564 | 57930045 | RAB3C | 148 |
| 15 | 90613827 | 90614787 | ZNF710 | 148 |
| 5 | 21530197 | 21530809 | CDH12 | 148 |
| 14 | 74209881 | 74211391 | C14orf43 | 148 |
| 1 | 224690810 | 224691960 | WDR26 | 148 |
| 16 | 3208776 | 3208871 | ZNF213 | 148 |
| 13 | 97389386 | 97390012 | HS6ST3 | 148 |
| 11 | 116955016 | 116955411 | SIK3 | 148 |
| 10 | 81025730 | 81027316 | ZMIZ1 | 148 |
| 5 | 175960066 | 175961306 | RNF44 | 148 |
| 16 | 75058590 | 75059084 | ZNRF1 | 148 |
| 17 | 42021260 | 42022256 | PPY | 148 |
| 4 | 37788748 | 37789296 | PGM2 | 148 |
| 15 | 83821726 | 83822218 | HDGFRP3 | 148 |
| 10 | 95172324 | 95172899 | MYOF | 148 |
| 3 | 194954426 | 194955029 | XXYLT1 | 148 |
| 4 | 74636359 | 74636916 | IL8 | 148 |
| 3 | 186427311 | 186428139 | KNG1 | 148 |
| 10 | 116694734 | 116695379 | TRUB1 | 147 |
| 19 | 11333441 | 11334020 | DOCK6 | 147 |
| 14 | 93837446 | 93838259 | UNC79 | 147 |
| 19 | 11333441 | 11334020 | C19orf80 | 147 |
| X | 49233251 | 49234384 | GAGE8 | 147 |
| X | 49233251 | 49234384 | GAGE2C | 147 |
| X | 49233251 | 49234384 | GAGE2A | 147 |
| X | 49233251 | 49234384 | GAGE2B | 147 |
| 3 | 196516194 | 196517266 | PAK2 | 147 |
| 8 | 6146979 | 6147624 | MCPH1 | 147 |
| 12 | 10251115 | 10251714 | CLEC1A | 147 |
| 7 | 17799027 | 17799599 | SNX13 | 147 |
| 13 | 71498367 | 71498942 | KLHL1 | 147 |
| 8 | 27183684 | 27184715 | PTK2B | 147 |
| 8 | 15977404 | 15978196 | MSR1 | 147 |
| 4 | 48582846 | 48583433 | FRYL | 147 |
| 1 | 18982399 | 18982833 | PAX7 | 147 |
| 6 | 143771312 | 143772237 | PEX3 | 147 |
| 12 | 33865779 | 33866256 | SYT10 | 147 |
| 13 | 84659079 | 84659877 | SLITRK1 | 147 |
| 3 | 49841553 | 49845568 | UBA7 | 147 |
| 1 | 9129215 | 9129682 | SLC2A5 | 147 |
| 6 | 4889729 | 4890090 | CDYL | 147 |
| 10 | 30314921 | 30317166 | KIAA1462 | 147 |
| 8 | 27183630 | 27183666 | PTK2B | 147 |
| 4 | 84405465 | 84406356 | FAM175A | 147 |
| 3 | 88208372 | 88209005 | C3orf38 | 147 |
| 3 | 88208372 | 88209005 | CGGBP1 | 147 |
| 2 | 62499968 | 62500233 | B3GNT2 | 147 |
| 11 | 108236195 | 108236777 | ATM | 147 |
| 3 | 83641171 | 83641813 | CADM2 | 147 |
| 7 | 50305814 | 50306724 | IKZF1 | 147 |
| 14 | 74243762 | 74244528 | C14orf43 | 147 |
| 10 | 18940344 | 18940837 | NSUN6 | 147 |
| X | 53253832 | 53254948 | KDM5C | 147 |
| 2 | 241551565 | 241552065 | GPR35 | 147 |
| 4 | 1220395 | 1223117 | CTBP1 | 147 |
| 1 | 54610449 | 54610499 | CDCP2 | 147 |
| 16 | 50315387 | 50316547 | ADCY7 | 147 |
| 15 | 32960355 | 32960472 | SCG5 | 147 |
| 5 | 66260122 | 66260546 | MAST4 | 147 |
| 19 | 53239465 | 53240036 | ZNF611 | 147 |
| 12 | 43249456 | 43250138 | PRICKLE1 | 146 |
| 4 | 154435850 | 154436370 | KIAA0922 | 146 |
| 4 | 39879560 | 39879626 | PDS5A | 146 |
| 7 | 150482155 | 150482945 | LOC100128542 | 146 |
| 12 | 6276705 | 6277713 | CD9 | 146 |
| 14 | 97727287 | 97727849 | VRK1 | 146 |
| 1 | 91120932 | 91121586 | BARHL2 | 146 |
| 2 | 99079184 | 99080937 | INPP4A | 146 |
| X | 129220538 | 129221866 | ELF4 | 146 |
| 4 | 102987161 | 102987819 | BANK1 | 146 |
| 10 | 28429003 | 28429559 | MPP7 | 146 |
| 3 | 48970387 | 48971422 | ARIH2 | 146 |
| 3 | 48970387 | 48971422 | C3orf71 | 146 |
| 2 | 70417123 | 70417166 | C2orf42 | 146 |
| 15 | 64938634 | 64939208 | ZNF609 | 146 |
| 11 | 89937348 | 89937952 | CHORDC1 | 146 |
| 17 | 44955034 | 44955146 | WNT9B | 146 |
| 3 | 195955968 | 195956877 | OSTalpha | 146 |
| 8 | 99097097 | 99098152 | C8orf47 | 146 |
| 15 | 68266654 | 68267424 | PIAS1 | 146 |
| 15 | 52049115 | 52050021 | TMOD2 | 146 |
| 6 | 85297905 | 85298494 | TBX18 | 146 |
| 2 | 66645340 | 66645901 | MEIS1 | 146 |
| 17 | 55887912 | 55888737 | MRPS23 | 146 |
| 6 | 26156125 | 26157702 | HIST1H2BD | 146 |
| 5 | 91332830 | 91333463 | ARRDC3 | 146 |
| 2 | 120200918 | 120201555 | SCTR | 146 |
| 4 | 29733088 | 29733602 | PCDH7 | 146 |
| 3 | 53169377 | 53169710 | PRKCD | 146 |
| 9 | 47259718 | 47260405 | CNTNAP3B | 146 |
| 19 | 39926028 | 39926929 | RPS16 | 146 |
| 1 | 89167407 | 89167899 | PKN2 | 146 |
| 14 | 63885404 | 63885829 | PPP2R5E | 146 |
| 1 | 226905002 | 226905806 | ITPKB | 146 |
| 14 | 71455032 | 71455532 | PCNX | 146 |
| 22 | 30184288 | 30185098 | ASCC2 | 146 |
| 11 | 68606353 | 68607470 | CPT1A | 146 |
| 1 | 91370198 | 91370740 | ZNF644 | 146 |
| 22 | 39226830 | 39226881 | NPTXR | 146 |
| 5 | 36872201 | 36872794 | NIPBL | 146 |
| 19 | 23869164 | 23870176 | ZNF675 | 146 |
| 11 | 102187810 | 102188583 | BIRC3 | 145 |
| 2 | 219262498 | 219263246 | CTDSP1 | 145 |
| 12 | 124916452 | 124917345 | NCOR2 | 145 |
| 6 | 63962687 | 63963551 | LGSN | 145 |
| 6 | 157155829 | 157156460 | ARID1B | 145 |
| 8 | 6676067 | 6677339 | XKR5 | 145 |
| 19 | 35933541 | 35933689 | FFAR2 | 145 |
| 1 | 9433309 | 9433614 | SPSB1 | 145 |
| 11 | 69078953 | 69080231 | MYEOV | 145 |
| 6 | 144415930 | 144417646 | SF3B5 | 145 |
| 3 | 18072014 | 18072453 | TBC1D5 | 145 |
| 1 | 12664423 | 12665131 | DHRS3 | 145 |
| 4 | 185654808 | 185655812 | MLF1IP | 145 |
| 11 | 75488188 | 75488323 | DGAT2 | 145 |
| 1 | 27812117 | 27812708 | WASF2 | 145 |
| 6 | 81745194 | 81745747 | FAM46A | 145 |
| 10 | 35853031 | 35853946 | CCNY | 145 |
| 6 | 152002311 | 152003168 | ESR1 | 145 |
| 19 | 11391355 | 11392369 | DOCK6 | 145 |
| 14 | 104176566 | 104176609 | XRCC3 | 145 |
| 3 | 193426715 | 193427709 | OPA1 | 145 |
| 1 | 144991842 | 144992783 | PDE4DIP | 145 |
| 7 | 131495768 | 131496254 | PODXL | 145 |
| 14 | 39901483 | 39901843 | FBXO33 | 145 |
| 12 | 9568876 | 9570285 | KLRB1 | 145 |
| 20 | 17485864 | 17486467 | BFSP1 | 145 |
| 7 | 36232167 | 36232384 | EEPD1 | 144 |
| 10 | 121338494 | 121338938 | TIAL1 | 144 |
| 1 | 8486112 | 8486589 | RERE | 144 |
| 17 | 78711592 | 78712492 | RPTOR | 144 |
| 14 | 90421872 | 90422846 | EFCAB11 | 144 |
| 13 | 77784048 | 77784785 | MYCBP2 | 144 |
| 11 | 93436733 | 93437327 | KIAA1731 | 144 |
| 17 | 75230595 | 75231645 | SEPT9 | 144 |
| 12 | 46048063 | 46049002 | ARID2 | 144 |
| 17 | 55681226 | 55682303 | MSI2 | 144 |
| 8 | 61472705 | 61473100 | RAB2A | 144 |
| 13 | 86702161 | 86702808 | SLITRK6 | 144 |
| 5 | 141618223 | 141618808 | SPRY4 | 144 |
| 21 | 34729514 | 34730265 | IFNAR1 | 144 |
| 8 | 110345603 | 110346990 | ENY2 | 144 |
| 3 | 197441612 | 197441772 | KIAA0226 | 144 |
| 19 | 2164592 | 2169718 | DOT1L | 144 |
| 2 | 243027038 | 243027734 | C2orf85 | 144 |
| 2 | 27304652 | 27304914 | EMILIN1 | 144 |
| 13 | 37574028 | 37575613 | ALG5 | 144 |
| 8 | 96099390 | 96099449 | PLEKHF2 | 144 |
| 13 | 84049874 | 84050783 | SLITRK1 | 144 |
| 8 | 81938389 | 81939317 | PAG1 | 144 |
| 6 | 36736814 | 36737950 | CPNE5 | 144 |
| 10 | 20276247 | 20276877 | PLXDC2 | 144 |
| 3 | 33899592 | 33900365 | PDCD6IP | 144 |
| 19 | 17833597 | 17833618 | MAP1S | 144 |
| 20 | 46356669 | 46356948 | SULF2 | 144 |
| 17 | 25858058 | 25858825 | KSR1 | 144 |
| 7 | 123655050 | 123655563 | TMEM229A | 144 |
| 16 | 89002591 | 89003813 | CBFA2T3 | 144 |
| 3 | 154307535 | 154308290 | GPR149 | 144 |
| 1 | 8878689 | 8879398 | RERE | 144 |
| 10 | 29231658 | 29232236 | BAMBI | 144 |
| 8 | 40841295 | 40842177 | ZMAT4 | 144 |
| 3 | 195039432 | 195040310 | ACAP2 | 144 |
| 12 | 10154362 | 10155466 | CLEC9A | 144 |
| 20 | 56025197 | 56026189 | RBM38 | 144 |
| 1 | 160983311 | 160983836 | F11R | 144 |
| 1 | 154392563 | 154393237 | IL6R | 143 |
| 19 | 2084372 | 2086284 | MOB3A | 143 |
| 8 | 78029512 | 78029987 | PEX2 | 143 |
| 13 | 31247853 | 31248709 | ALOX5AP | 143 |
| 1 | 65457644 | 65458280 | JAK1 | 143 |
| 13 | 84873436 | 84874354 | SLITRK1 | 143 |
| 10 | 135475121 | 135475699 | DUX2 | 143 |
| 10 | 135475121 | 135475699 | DUX4L6 | 143 |
| 8 | 9341924 | 9342525 | TNKS | 143 |
| 5 | 149461202 | 149462195 | CSF1R | 143 |
| 6 | 24467977 | 24468580 | GPLD1 | 143 |
| 13 | 42614059 | 42614630 | DGKH | 143 |
| 2 | 98482084 | 98482801 | TMEM131 | 143 |
| 2 | 55523593 | 55524382 | CCDC88A | 143 |
| 7 | 23681395 | 23682422 | CCDC126 | 143 |
| 15 | 28731973 | 28732022 | HERC2 | 143 |
| 6 | 35338049 | 35338594 | PPARD | 143 |
| 15 | 93841387 | 93842892 | RGMA | 143 |
| 7 | 105172360 | 105173246 | RINT1 | 143 |
| 10 | 135091097 | 135093683 | ADAM8 | 143 |
| 6 | 53223408 | 53224903 | ELOVL5 | 143 |
| 17 | 28033618 | 28034527 | SSH2 | 143 |
| 1 | 150013167 | 150013731 | VPS45 | 143 |
| 1 | 33406607 | 33409513 | RNF19B | 143 |
| 1 | 53136556 | 53137123 | SELRC1 | 143 |
| 9 | 134390534 | 134390715 | POMT1 | 143 |
| 14 | 103811476 | 103812695 | EIF5 | 143 |
| 17 | 78723361 | 78723737 | RPTOR | 143 |
| 17 | 78724132 | 78724624 | RPTOR | 143 |
| 7 | 158502520 | 158503161 | NCAPG2 | 143 |
| 5 | 90575216 | 90576756 | ARRDC3 | 143 |
| 19 | 14317631 | 14319037 | LPHN1 | 143 |
| 10 | 14719173 | 14719962 | FAM107B | 143 |
| 19 | 41832295 | 41835954 | CCDC97 | 143 |
| 14 | 100995164 | 100996389 | WDR25 | 143 |
| 4 | 83294708 | 83295920 | HNRNPD | 143 |
| 7 | 94837622 | 94838086 | PPP1R9A | 143 |
| 14 | 82210604 | 82210986 | SEL1L | 143 |
| 5 | 77557140 | 77558040 | AP3B1 | 143 |
| 15 | 102291301 | 102294960 | TARSL2 | 143 |
| 20 | 49072173 | 49073231 | PTPN1 | 143 |
| 4 | 28075281 | 28075991 | STIM2 | 143 |
| 14 | 54954985 | 54956038 | GMFB | 143 |
| 3 | 58457293 | 58457669 | KCTD6 | 143 |
| 3 | 9404365 | 9405228 | THUMPD3 | 143 |
| 3 | 15864485 | 15865041 | ANKRD28 | 143 |
| 3 | 50296183 | 50298843 | C3orf45 | 143 |
| 6 | 135006302 | 135006886 | ALDH8A1 | 143 |
| 11 | 88689542 | 88690074 | GRM5 | 143 |
| 12 | 20506549 | 20507113 | PDE3A | 142 |
| 14 | 102062128 | 102063670 | DIO3 | 142 |
| 16 | 89831211 | 89832036 | FANCA | 142 |
| 8 | 26446687 | 26446940 | DPYSL2 | 142 |
| 20 | 61957900 | 61957922 | COL20A1 | 142 |
| 16 | 4256329 | 4257418 | SRL | 142 |
| 4 | 56261440 | 56262318 | TMEM165 | 142 |
| 1 | 28670689 | 28671410 | PHACTR4 | 142 |
| 7 | 36406660 | 36407537 | KIAA0895 | 142 |
| 6 | 34949308 | 34950131 | ANKS1A | 142 |
| 8 | 142129750 | 142131695 | DENND3 | 142 |
| 2 | 61404850 | 61405615 | AHSA2 | 142 |
| 7 | 130698146 | 130698903 | MKLN1 | 142 |
| 18 | 24376844 | 24376875 | AQP4 | 142 |
| 19 | 1076510 | 1077791 | HMHA1 | 142 |
| 10 | 75011030 | 75012832 | MRPS16 | 142 |
| 13 | 82599997 | 82600828 | SPRY2 | 142 |
| 17 | 41886774 | 41887353 | MPP3 | 142 |
| 8 | 98709379 | 98709953 | MTDH | 142 |
| 3 | 149414198 | 149414279 | WWTR1 | 142 |
| 17 | 25859107 | 25860029 | KSR1 | 142 |
| 17 | 80943591 | 80944519 | B3GNTL1 | 142 |
| 15 | 86026345 | 86027078 | AKAP13 | 142 |
| 11 | 75862758 | 75864464 | WNT11 | 142 |
| 7 | 74594220 | 74597460 | GTF2IRD2B | 142 |
| 16 | 80567628 | 80568194 | DYNLRB2 | 142 |
| 7 | 138291502 | 138291663 | SVOPL | 142 |
| 9 | 32421203 | 32421937 | ACO1 | 142 |
| 16 | 69459758 | 69460437 | CYB5B | 142 |
| 12 | 104470812 | 104471381 | HCFC2 | 142 |
| 2 | 17943286 | 17943755 | GEN1 | 142 |
| 6 | 137832448 | 137833110 | OLIG3 | 142 |
| 7 | 122828638 | 122829187 | SLC13A1 | 142 |
| 6 | 26568134 | 26568188 | ABT1 | 142 |
| 17 | 78884117 | 78885442 | RPTOR | 142 |
| 11 | 121335904 | 121335907 | SORL1 | 142 |
| 2 | 136873974 | 136875236 | CXCR4 | 142 |
| 1 | 161734484 | 161735106 | ATF6 | 142 |
| 8 | 22461743 | 22463129 | KIAA1967 | 142 |
| 13 | 91559209 | 91559885 | GPC5 | 142 |
| 7 | 140678445 | 140679168 | MRPS33 | 142 |
| 1 | 91522373 | 91523047 | ZNF644 | 142 |
| 6 | 27448780 | 27449795 | ZNF184 | 142 |
| 12 | 37918211 | 37919145 | ALG10B | 142 |
| 15 | 21232969 | 21233721 | POTEB | 142 |
| 2 | 95736868 | 95737263 | MAL | 142 |
| 5 | 83447523 | 83448053 | EDIL3 | 142 |
| 7 | 5367253 | 5368885 | TNRC18 | 142 |
| 11 | 65185897 | 65187305 | FRMD8 | 141 |
| 1 | 39493777 | 39494019 | NDUFS5 | 141 |
| 2 | 24478269 | 24479185 | ITSN2 | 141 |
| 13 | 81583721 | 81584299 | SPRY2 | 141 |
| 7 | 152106148 | 152108362 | MLL3 | 141 |
| 11 | 118460022 | 118460580 | ARCN1 | 141 |
| 2 | 47604898 | 47605802 | EPCAM | 141 |
| 12 | 123850396 | 123850491 | SETD8 | 141 |
| 4 | 6909123 | 6909921 | TBC1D14 | 141 |
| 16 | 31044042 | 31044924 | STX4 | 141 |
| 2 | 61443357 | 61443993 | USP34 | 141 |
| 2 | 10191681 | 10192062 | KLF11 | 141 |
| 8 | 96100844 | 96101646 | PLEKHF2 | 141 |
| 3 | 46331915 | 46332324 | CCR3 | 141 |
| 8 | 7566938 | 7567675 | FAM90A8 | 141 |
| 6 | 149843561 | 149844150 | PPIL4 | 141 |
| 19 | 39183827 | 39186319 | ACTN4 | 141 |
| 6 | 91269804 | 91270302 | MAP3K7 | 141 |
| 2 | 61220713 | 61221370 | PUS10 | 141 |
| 2 | 61220713 | 61221370 | PEX13 | 141 |
| 5 | 135336867 | 135337858 | TGFBI | 141 |
| 8 | 70839058 | 70839637 | SLCO5A1 | 141 |
| 17 | 66015891 | 66017650 | C17orf58 | 141 |
| 21 | 36237643 | 36238447 | RUNX1 | 141 |
| 2 | 71684298 | 71684307 | DYSF | 141 |
| 2 | 122288707 | 122289588 | CLASP1 | 141 |
| 1 | 59387787 | 59388500 | JUN | 141 |
| 16 | 29638067 | 29639700 | SPN | 141 |
| 7 | 101499953 | 101500590 | CUX1 | 141 |
| 13 | 91909459 | 91909493 | GPC5 | 141 |
| 18 | 60476435 | 60477010 | PHLPP1 | 141 |
| 15 | 75992005 | 75993653 | CSPG4 | 141 |
| 14 | 70233321 | 70234775 | SRSF5 | 141 |
| 8 | 7400146 | 7401231 | FAM90A7 | 141 |
| 6 | 141460615 | 141461005 | NMBR | 141 |
| 14 | 67955983 | 67956907 | TMEM229B | 141 |
| 9 | 30090619 | 30091233 | LINGO2 | 141 |
| 7 | 17412764 | 17413599 | AHR | 141 |
| 2 | 98809812 | 98810457 | VWA3B | 140 |
| 10 | 24672059 | 24673408 | KIAA1217 | 140 |
| 13 | 82874196 | 82875178 | SLITRK1 | 140 |
| 6 | 25463858 | 25464621 | LRRC16A | 140 |
| 11 | 65409148 | 65409367 | SIPA1 | 140 |
| 9 | 120466508 | 120466945 | TLR4 | 140 |
| 11 | 104148405 | 104148859 | PDGFD | 140 |
| 17 | 73774478 | 73776051 | H3F3B | 140 |
| 6 | 137092915 | 137093467 | MAP3K5 | 140 |
| 21 | 36206981 | 36207798 | RUNX1 | 140 |
| 9 | 102130202 | 102131496 | SEC61B | 140 |
| 12 | 24754476 | 24754922 | SOX5 | 140 |
| 9 | 117134203 | 117135650 | AKNA | 140 |
| 19 | 48833063 | 48835341 | CCDC114 | 140 |
| 4 | 26232822 | 26233717 | RBPJ | 140 |
| 7 | 16890706 | 16891189 | AGR3 | 140 |
| 9 | 29668216 | 29668747 | LINGO2 | 140 |
| 5 | 16759927 | 16759940 | MYO10 | 140 |
| 8 | 19956482 | 19957155 | SLC18A1 | 140 |
| 22 | 45061463 | 45061740 | PRR5 | 140 |
| 19 | 2478313 | 2479310 | LMNB2 | 140 |
| 8 | 59153474 | 59153755 | UBXN2B | 140 |
| 12 | 121378004 | 121378647 | SPPL3 | 140 |
| 3 | 81464126 | 81464755 | GBE1 | 140 |
| 19 | 13274712 | 13275975 | STX10 | 140 |
| 13 | 89607847 | 89608476 | SLITRK5 | 140 |
| 15 | 52383872 | 52385407 | BCL2L10 | 140 |
| 3 | 183408935 | 183411386 | YEATS2 | 140 |
| 10 | 80896977 | 80898364 | ZMIZ1 | 140 |
| 17 | 66677051 | 66677348 | FAM20A | 140 |
| X | 19765480 | 19766208 | SH3KBP1 | 140 |
| 2 | 47297913 | 47300716 | TTC7A | 140 |
| 6 | 135007931 | 135008452 | ALDH8A1 | 140 |
| 5 | 156998805 | 156999088 | ADAM19 | 140 |
| 3 | 112471066 | 112471751 | CD200R1L | 140 |
| 1 | 39995292 | 39995470 | BMP8A | 140 |
| 17 | 74482446 | 74482453 | RHBDF2 | 139 |
| 8 | 38793856 | 38794996 | PLEKHA2 | 139 |
| 20 | 31268811 | 31270088 | C20orf203 | 139 |
| 9 | 124320118 | 124320913 | DAB2IP | 139 |
| 10 | 79802503 | 79803142 | RPS24 | 139 |
| 3 | 13151529 | 13153137 | IQSEC1 | 139 |
| 4 | 39946704 | 39947737 | PDS5A | 139 |
| 3 | 32556870 | 32558345 | CMTM6 | 139 |
| 11 | 69064441 | 69066717 | MYEOV | 139 |
| 2 | 66671448 | 66672176 | MEIS1 | 139 |
| 3 | 171701879 | 171702412 | FNDC3B | 139 |
| 8 | 9626830 | 9627436 | TNKS | 139 |
| 16 | 79127152 | 79128511 | WWOX | 139 |
| 12 | 48171326 | 48171343 | SLC48A1 | 139 |
| 16 | 89043224 | 89044246 | CBFA2T3 | 139 |
| 7 | 76522338 | 76522780 | CCDC146 | 139 |
| 2 | 24030244 | 24030733 | ATAD2B | 139 |
| 8 | 99048843 | 99049326 | C8orf47 | 139 |
| 10 | 112745967 | 112746451 | SHOC2 | 139 |
| 14 | 103781776 | 103783568 | EIF5 | 139 |
| 10 | 71240346 | 71241026 | TSPAN15 | 139 |
| 4 | 35697918 | 35698507 | ARAP2 | 139 |
| 10 | 71238764 | 71239756 | TSPAN15 | 139 |
| 6 | 156817300 | 156818029 | ARID1B | 139 |
| 2 | 32547152 | 32547741 | BIRC6 | 139 |
| 11 | 128593708 | 128594396 | FLI1 | 139 |
| 6 | 34785355 | 34786259 | UHRF1BP1 | 139 |
| 3 | 85442919 | 85443331 | CADM2 | 139 |
| 10 | 80503769 | 80505024 | ZMIZ1 | 139 |
| 13 | 110790359 | 110790837 | COL4A1 | 139 |
| 10 | 117189129 | 117189695 | ATRNL1 | 139 |
| 15 | 75338236 | 75340179 | PPCDC | 138 |
| 8 | 23103570 | 23104625 | CHMP7 | 138 |
| 13 | 114444789 | 114445714 | FAM70B | 138 |
| 5 | 70751239 | 70752090 | BDP1 | 138 |
| 5 | 149462450 | 149462872 | CSF1R | 138 |
| 13 | 21050469 | 21050640 | CRYL1 | 138 |
| 3 | 73144090 | 73144674 | EBLN2 | 138 |
| 16 | 17472942 | 17473515 | XYLT1 | 138 |
| 7 | 13458945 | 13459734 | ETV1 | 138 |
| 2 | 43447852 | 43449357 | ZFP36L2 | 138 |
| 3 | 48377234 | 48377532 | SPINK8 | 138 |
| 4 | 8199774 | 8204390 | SH3TC1 | 138 |
| 6 | 39303584 | 39304417 | KIF6 | 138 |
| 8 | 27262961 | 27263323 | PTK2B | 138 |
| 6 | 26158247 | 26159233 | HIST1H2BD | 138 |
| 12 | 12381693 | 12381730 | LRP6 | 138 |
| 12 | 40574454 | 40575440 | LRRK2 | 138 |
| 7 | 99624286 | 99625332 | ZKSCAN1 | 138 |
| 19 | 11244310 | 11246714 | LDLR | 138 |
| 13 | 21918542 | 21919309 | ZDHHC20 | 138 |
| 2 | 239432153 | 239432603 | ASB1 | 138 |
| 10 | 43578051 | 43579633 | RET | 138 |
| 7 | 29669076 | 29669533 | PRR15 | 138 |
| 14 | 74278302 | 74278845 | C14orf43 | 138 |
| 16 | 80810539 | 80811126 | CDYL2 | 138 |
| 9 | 5492647 | 5492983 | PDCD1LG2 | 138 |
| 3 | 150186492 | 150187139 | TSC22D2 | 138 |
| 2 | 25516555 | 25518462 | DNMT3A | 138 |
| 17 | 2717401 | 2718809 | RAP1GAP2 | 138 |
| 3 | 119812744 | 119814744 | GSK3B | 138 |
| 11 | 108873482 | 108873942 | DDX10 | 138 |
| 19 | 44249583 | 44250560 | SMG9 | 138 |
| 2 | 24088867 | 24089304 | ATAD2B | 138 |
| 2 | 162164522 | 162165413 | PSMD14 | 138 |
| 19 | 28283740 | 28285927 | UQCRFS1 | 138 |
| 12 | 47001388 | 47002273 | SLC38A4 | 138 |
| 17 | 75470327 | 75470764 | SEPT9 | 138 |
| 20 | 23206198 | 23206746 | NXT1 | 138 |
| 1 | 44470954 | 44471745 | SLC6A9 | 138 |
| 7 | 77535809 | 77536348 | PHTF2 | 138 |
| 3 | 196309809 | 196310721 | FBXO45 | 138 |
| X | 108280891 | 108281250 | IRS4 | 138 |
| 11 | 44644615 | 44645296 | CD82 | 138 |
| 6 | 153572884 | 153573425 | RGS17 | 138 |
| 8 | 128748175 | 128748909 | MYC | 138 |
| 3 | 188405224 | 188406003 | LPP | 138 |
| 10 | 101742145 | 101743509 | DNMBP | 138 |
| 7 | 105720544 | 105721096 | SYPL1 | 137 |
| 19 | 16482973 | 16483288 | EPS15L1 | 137 |
| 8 | 14047978 | 14048191 | SGCZ | 137 |
| 15 | 65278247 | 65279080 | SPG21 | 137 |
| 3 | 186193505 | 186194897 | CRYGS | 137 |
| 11 | 67821168 | 67821197 | CHKA | 137 |
| 6 | 159501899 | 159502548 | TAGAP | 137 |
| 16 | 77065828 | 77066256 | MON1B | 137 |
| 6 | 56595749 | 56596092 | DST | 137 |
| 18 | 46449140 | 46450302 | SMAD7 | 137 |
| 2 | 242316579 | 242317151 | FARP2 | 137 |
| 4 | 38218478 | 38218975 | PTTG2 | 137 |
| 2 | 43360388 | 43362494 | ZFP36L2 | 137 |
| 14 | 74276603 | 74277516 | C14orf43 | 137 |
| 6 | 155485317 | 155486229 | TIAM2 | 137 |
| 4 | 39639809 | 39641657 | C4orf34 | 137 |
| 3 | 190124311 | 190125038 | CLDN16 | 137 |
| 2 | 86117015 | 86117512 | ST3GAL5 | 137 |
| 3 | 191575287 | 191575864 | PYDC2 | 137 |
| 17 | 78431222 | 78431931 | NPTX1 | 137 |
| 16 | 85967868 | 85968844 | IRF8 | 137 |
| 9 | 116310786 | 116311521 | RGS3 | 137 |
| 14 | 90863000 | 90863904 | CALM1 | 137 |
| 2 | 75136444 | 75136798 | POLE4 | 137 |
| 7 | 2611575 | 2612202 | IQCE | 137 |
| 20 | 10454926 | 10455467 | C20orf94 | 137 |
| 8 | 23020236 | 23022110 | TNFRSF10D | 137 |
| 3 | 123126979 | 123127769 | ADCY5 | 137 |
| 2 | 200819849 | 200820641 | C2orf47 | 137 |
| 2 | 200819849 | 200820641 | TYW5 | 137 |
| 1 | 109461377 | 109461975 | GPSM2 | 137 |
| 10 | 114135763 | 114136435 | ACSL5 | 137 |
| 13 | 41635022 | 41636284 | WBP4 | 137 |
| 12 | 43637998 | 43638568 | ADAMTS20 | 137 |
| 1 | 65840748 | 65841547 | DNAJC6 | 137 |
| 3 | 119806301 | 119807184 | GSK3B | 137 |
| 7 | 23000163 | 23000746 | FAM126A | 137 |
| 7 | 11052705 | 11053226 | PHF14 | 137 |
| 6 | 23204471 | 23205011 | HDGFL1 | 137 |
| 3 | 18392507 | 18392984 | SATB1 | 137 |
| 2 | 152493806 | 152494215 | NEB | 137 |
| 10 | 121329822 | 121329836 | RGS10 | 137 |
| 13 | 86212119 | 86214062 | SLITRK6 | 137 |
| 5 | 104727501 | 104728368 | NUDT12 | 137 |
| 20 | 56007530 | 56008769 | RBM38 | 137 |
| 8 | 86300614 | 86301141 | CA1 | 137 |
| 18 | 60648777 | 60649583 | PHLPP1 | 137 |
| 7 | 13174358 | 13174908 | ARL4A | 137 |
| 14 | 94477537 | 94478055 | OTUB2 | 137 |
| 15 | 63795001 | 63797076 | USP3 | 137 |
| 3 | 64008551 | 64009563 | PSMD6 | 137 |
| 13 | 91935576 | 91936372 | GPC5 | 137 |
| X | 128920456 | 128921032 | SASH3 | 137 |
| 15 | 42329252 | 42330070 | PLA2G4E | 137 |
| 15 | 32933507 | 32934198 | SCG5 | 137 |
| 20 | 45178789 | 45179526 | C20orf123 | 137 |
| 11 | 75554620 | 75555063 | UVRAG | 137 |
| 21 | 25001102 | 25001696 | MRPL39 | 137 |
| 3 | 180905720 | 180906245 | DNAJC19 | 137 |
| 3 | 38095872 | 38096142 | DLEC1 | 137 |
| 14 | 45721838 | 45723229 | MIS18BP1 | 137 |
| 5 | 102627811 | 102628291 | C5orf30 | 136 |
| 4 | 2838886 | 2839935 | SH3BP2 | 136 |
| 12 | 38594833 | 38594959 | ALG10B | 136 |
| 6 | 47209988 | 47210528 | TNFRSF21 | 136 |
| 17 | 2693562 | 2694404 | RAP1GAP2 | 136 |
| 1 | 23042888 | 23044149 | EPHB2 | 136 |
| 15 | 75334796 | 75336783 | PPCDC | 136 |
| 10 | 65057589 | 65058296 | JMJD1C | 136 |
| 18 | 20883409 | 20884329 | C18orf45 | 136 |
| 19 | 8550546 | 8555251 | HNRNPM | 136 |
| 6 | 164146951 | 164147739 | QKI | 136 |
| 16 | 1294541 | 1295460 | TPSG1 | 136 |
| 13 | 75917294 | 75918208 | TBC1D4 | 136 |
| 16 | 4713490 | 4714659 | MGRN1 | 136 |
| 2 | 101915092 | 101915672 | RNF149 | 136 |
| 3 | 23639742 | 23640468 | UBE2E1 | 136 |
| 12 | 46357871 | 46358544 | SCAF11 | 136 |
| 12 | 39718847 | 39719677 | KIF21A | 136 |
| 1 | 36106189 | 36107955 | PSMB2 | 136 |
| 7 | 120066452 | 120067085 | KCND2 | 136 |
| 17 | 78414701 | 78415468 | ENDOV | 136 |
| 9 | 139513736 | 139514859 | EGFL7 | 136 |
| 19 | 33395291 | 33395864 | CEP89 | 136 |
| 2 | 8474967 | 8475626 | ID2 | 136 |
| 8 | 11862764 | 11863406 | DEFB134 | 136 |
| 14 | 51357893 | 51358389 | ABHD12B | 136 |
| 5 | 36389787 | 36390485 | RANBP3L | 136 |
| 3 | 155894091 | 155894793 | KCNAB1 | 136 |
| 13 | 90302070 | 90303023 | GPC5 | 136 |
| 3 | 46580060 | 46580563 | LRRC2 | 136 |
| 22 | 38028854 | 38030565 | PDXP | 136 |
| 16 | 3207344 | 3208450 | ZNF213 | 136 |
| 16 | 48399415 | 48400014 | SIAH1 | 136 |
| 3 | 30103144 | 30103786 | TGFBR2 | 135 |
| 2 | 17219308 | 17219819 | FAM49A | 135 |
| 18 | 74792087 | 74792707 | MBP | 135 |
| 6 | 6655417 | 6655846 | LY86 | 135 |
| 12 | 9803051 | 9803461 | CLEC2D | 135 |
| 15 | 94748992 | 94749833 | MCTP2 | 135 |
| 1 | 147874402 | 147874915 | PPIAL4A | 135 |
| 7 | 101515793 | 101516585 | CUX1 | 135 |
| 3 | 197147770 | 197149760 | DLG1 | 135 |
| 7 | 155153851 | 155154826 | INSIG1 | 135 |
| 6 | 44204656 | 44206103 | HSP90AB1 | 135 |
| 13 | 90884955 | 90886148 | GPC5 | 135 |
| 14 | 51382448 | 51382849 | PYGL | 135 |
| 1 | 109234567 | 109235504 | PRPF38B | 135 |
| 3 | 166859211 | 166859784 | ZBBX | 135 |
| 7 | 6266015 | 6266827 | CYTH3 | 135 |
| 17 | 66129229 | 66129925 | KPNA2 | 135 |
| 11 | 70269912 | 70270890 | CTTN | 135 |
| 1 | 105827603 | 105828070 | AMY1B | 135 |
| 12 | 39432046 | 39432840 | CPNE8 | 135 |
| 4 | 2430616 | 2431921 | LOC402160 | 135 |
| 1 | 154379972 | 154380412 | IL6R | 135 |
| 10 | 24973522 | 24974047 | ARHGAP21 | 135 |
| 16 | 70461596 | 70461635 | ST3GAL2 | 135 |
| 1 | 87467265 | 87468019 | HS2ST1 | 135 |
| 6 | 170403238 | 170405631 | DLL1 | 135 |
| 18 | 77551882 | 77553063 | KCNG2 | 135 |
| 4 | 78784424 | 78784548 | MRPL1 | 135 |
| 10 | 75489805 | 75490675 | SEC24C | 135 |
| 14 | 73707118 | 73708340 | PAPLN | 135 |
| 17 | 4458117 | 4459138 | MYBBP1A | 135 |
| 6 | 6885300 | 6886727 | RREB1 | 135 |
| 4 | 174255820 | 174256701 | HMGB2 | 135 |
| 10 | 129775932 | 129776375 | PTPRE | 135 |
| 7 | 13091768 | 13092368 | ARL4A | 135 |
| 5 | 148726042 | 148726711 | GRPEL2 | 135 |
| 2 | 29052274 | 29053505 | SPDYA | 135 |
| 14 | 70080590 | 70081365 | KIAA0247 | 135 |
| 5 | 82356144 | 82356734 | TMEM167A | 135 |
| 18 | 55549092 | 55549610 | ATP8B1 | 135 |
| 12 | 38948366 | 38949136 | ALG10B | 135 |
| 17 | 41819388 | 41819925 | SOST | 135 |
| 10 | 6513592 | 6514369 | PRKCQ | 135 |
| 20 | 47404164 | 47404165 | PREX1 | 135 |
| 17 | 39228109 | 39228674 | KRTAP2-1 | 135 |
| 13 | 114443708 | 114444473 | FAM70B | 135 |
| 12 | 9800302 | 9801353 | CLEC2D | 135 |
| 7 | 65165069 | 65165734 | VKORC1L1 | 135 |
| 5 | 74980082 | 74981346 | POC5 | 135 |
| 3 | 184406666 | 184410008 | MAGEF1 | 135 |
| 6 | 23920767 | 23921196 | NRSN1 | 135 |
| 8 | 29609422 | 29609659 | TMEM66 | 135 |
| 17 | 75883405 | 75884023 | TNRC6C | 135 |
| 7 | 87440393 | 87441041 | RUNDC3B | 135 |
| 2 | 37411573 | 37412110 | SULT6B1 | 135 |
| 7 | 32613 | 33278 | FAM20C | 135 |
| 11 | 10878867 | 10880610 | ZBED5 | 134 |
| 15 | 81157327 | 81157898 | KIAA1199 | 134 |
| 15 | 50715550 | 50716913 | USP8 | 134 |
| 3 | 195700324 | 195703200 | TNK2 | 134 |
| 3 | 184404266 | 184406535 | MAGEF1 | 134 |
| 13 | 114063653 | 114064891 | ADPRHL1 | 134 |
| 13 | 81133587 | 81134272 | SPRY2 | 134 |
| 3 | 196992338 | 196993582 | DLG1 | 134 |
| 13 | 91894091 | 91895118 | GPC5 | 134 |
| 9 | 130545019 | 130545894 | SH2D3C | 134 |
| 1 | 51434277 | 51435844 | CDKN2C | 134 |
| 6 | 38032649 | 38033226 | ZFAND3 | 134 |
| 8 | 129061084 | 129061970 | MYC | 134 |
| 3 | 516911 | 517461 | CHL1 | 134 |
| 16 | 83290119 | 83290573 | CDH13 | 134 |
| 6 | 136610035 | 136611397 | BCLAF1 | 134 |
| 15 | 67799183 | 67800694 | C15orf61 | 134 |
| 10 | 22014011 | 22015279 | MLLT10 | 134 |
| 12 | 64894432 | 64894939 | TBK1 | 134 |
| 10 | 75006546 | 75007562 | DNAJC9 | 134 |
| 6 | 37437661 | 37438379 | FTSJD2 | 134 |
| 4 | 39774727 | 39776502 | UBE2K | 134 |
| 8 | 42195309 | 42196485 | POLB | 134 |
| 16 | 89044292 | 89045820 | CBFA2T3 | 134 |
| 7 | 44678488 | 44679450 | OGDH | 134 |
| 11 | 76885750 | 76887482 | MYO7A | 134 |
| 15 | 28730883 | 28731836 | HERC2 | 134 |
| 7 | 56133940 | 56133967 | SUMF2 | 134 |
| 3 | 148750220 | 148750896 | HLTF | 134 |
| Y | 59032612 | 59033225 | SPRY3 | 134 |
| 3 | 155894996 | 155895575 | KCNAB1 | 134 |
| 13 | 89000648 | 89002339 | SLITRK5 | 134 |
| 2 | 120144674 | 120144855 | C2orf76 | 134 |
| 10 | 73556923 | 73558363 | CDH23 | 134 |
| 18 | 48679900 | 48680965 | MEX3C | 134 |
| 19 | 50018187 | 50018558 | FCGRT | 134 |
| 2 | 100851370 | 100852268 | LONRF2 | 134 |
| 4 | 48130227 | 48130962 | TXK | 134 |
| 4 | 10672401 | 10673163 | CLNK | 133 |
| 3 | 183087737 | 183088303 | MCF2L2 | 133 |
| 16 | 15686950 | 15687620 | KIAA0430 | 133 |
| 8 | 39044704 | 39045202 | ADAM32 | 133 |
| 18 | 74826327 | 74827300 | MBP | 133 |
| 1 | 27762448 | 27763012 | WASF2 | 133 |
| 11 | 62270514 | 62271478 | AHNAK | 133 |
| 12 | 10603767 | 10604420 | KLRC1 | 133 |
| 2 | 55646407 | 55647218 | CCDC88A | 133 |
| 15 | 93447184 | 93447891 | CHD2 | 133 |
| 2 | 74681810 | 74682595 | INO80B | 133 |
| 6 | 132680505 | 132681060 | MOXD1 | 133 |
| 11 | 63637448 | 63637990 | MARK2 | 133 |
| 22 | 31886070 | 31886396 | EIF4ENIF1 | 133 |
| 19 | 35536334 | 35537745 | HPN | 133 |
| 11 | 10765157 | 10765631 | CTR9 | 133 |
| 2 | 65144288 | 65144492 | SLC1A4 | 133 |
| 22 | 18596456 | 18597262 | TUBA8 | 133 |
| 7 | 133318108 | 133318640 | EXOC4 | 133 |
| 1 | 147790156 | 147790446 | PPIAL4A | 133 |
| 6 | 146011384 | 146011918 | EPM2A | 133 |
| 11 | 65525020 | 65525800 | OVOL1 | 133 |
| 8 | 26466943 | 26467366 | DPYSL2 | 133 |
| 1 | 51796712 | 51797162 | TTC39A | 133 |
| 15 | 45899429 | 45900035 | PLDN | 133 |
| 6 | 75032493 | 75033109 | CD109 | 133 |
| 6 | 42784108 | 42784791 | KIAA0240 | 133 |
| 16 | 68291554 | 68292358 | PLA2G15 | 133 |
| 19 | 13942982 | 13945822 | ZSWIM4 | 133 |
| 12 | 129273560 | 129273947 | SLC15A4 | 133 |
| 14 | 91750639 | 91751340 | CCDC88C | 133 |
| 22 | 18254402 | 18255046 | BID | 133 |
| 9 | 137213435 | 137213613 | RXRA | 133 |
| 14 | 101106214 | 101107556 | BEGAIN | 133 |
| 10 | 35740401 | 35740994 | CCNY | 133 |
| 9 | 100319590 | 100320235 | TMOD1 | 133 |
| 6 | 41168481 | 41169244 | TREML2 | 133 |
| 3 | 119597900 | 119598464 | GSK3B | 133 |
| 12 | 46204424 | 46205384 | ARID2 | 133 |
| 4 | 22496917 | 22497640 | GPR125 | 133 |
| 17 | 75436352 | 75437594 | SEPT9 | 133 |
| 3 | 152726279 | 152727122 | RAP2B | 133 |
| 11 | 102157499 | 102157892 | BIRC3 | 132 |
| 14 | 51717022 | 51717631 | TMX1 | 132 |
| 21 | 37553406 | 37554212 | DOPEY2 | 132 |
| 1 | 9031378 | 9031844 | CA6 | 132 |
| 12 | 37859929 | 37860649 | ALG10B | 132 |
| 8 | 81447035 | 81447685 | ZBTB10 | 132 |
| 6 | 112171674 | 112172247 | FYN | 132 |
| 11 | 8703623 | 8704586 | RPL27A | 132 |
| 7 | 150147487 | 150149186 | GIMAP8 | 132 |
| 20 | 7946162 | 7946672 | HAO1 | 132 |
| 2 | 228324745 | 228325194 | AGFG1 | 132 |
| 6 | 26122548 | 26122611 | HIST1H2AC | 132 |
| 7 | 23501769 | 23502558 | IGF2BP3 | 132 |
| 4 | 26413063 | 26414008 | RBPJ | 132 |
| 2 | 26516800 | 26517404 | HADHB | 132 |
| 3 | 189919085 | 189919817 | LEPREL1 | 132 |
| 6 | 103640409 | 103641038 | HACE1 | 132 |
| 2 | 44183619 | 44184137 | LRPPRC | 132 |
| 21 | 36226068 | 36226482 | RUNX1 | 132 |
| 10 | 126335671 | 126337053 | FAM53B | 132 |
| 22 | 33153119 | 33153641 | SYN3 | 132 |
| 1 | 35341768 | 35342539 | DLGAP3 | 132 |
| 17 | 80358398 | 80359418 | C17orf101 | 132 |
| 17 | 80358398 | 80359418 | HEXDC | 132 |
| 5 | 107403976 | 107404566 | FBXL17 | 132 |
| 7 | 123175391 | 123175890 | IQUB | 132 |
| 1 | 161696341 | 161696843 | FCRLB | 132 |
| 1 | 34859821 | 34860339 | C1orf94 | 132 |
| 8 | 56734559 | 56734849 | TGS1 | 132 |
| 15 | 75641570 | 75642902 | NEIL1 | 132 |
| 9 | 116766470 | 116767245 | ZNF618 | 132 |
| 2 | 234074538 | 234075333 | INPP5D | 131 |
| 3 | 196684834 | 196685914 | PIGZ | 131 |
| 2 | 238806673 | 238806736 | RAMP1 | 131 |
| 9 | 127084546 | 127085590 | NEK6 | 131 |
| 13 | 80015801 | 80016300 | RBM26 | 131 |
| 1 | 209823546 | 209823589 | LAMB3 | 131 |
| 17 | 61919948 | 61921028 | SMARCD2 | 131 |
| 10 | 72179608 | 72180228 | EIF4EBP2 | 131 |
| 15 | 64337065 | 64337277 | DAPK2 | 131 |
| 15 | 66909503 | 66910935 | LCTL | 131 |
| 16 | 74959680 | 74960323 | WDR59 | 131 |
| 5 | 75148276 | 75149532 | POC5 | 131 |
| 2 | 209130453 | 209131109 | PIKFYVE | 131 |
| 3 | 14607249 | 14607830 | GRIP2 | 131 |
| 13 | 47370524 | 47371593 | ESD | 131 |
| 17 | 41437977 | 41438763 | ARL4D | 131 |
| 6 | 94080381 | 94080998 | EPHA7 | 131 |
| 13 | 107188495 | 107188960 | EFNB2 | 131 |
| 13 | 91236627 | 91237370 | GPC5 | 131 |
| X | 134908520 | 134910401 | CT45A3 | 131 |
| 8 | 141114544 | 141115184 | TRAPPC9 | 131 |
| 10 | 61008786 | 61009255 | PHYHIPL | 131 |
| 10 | 61008786 | 61009255 | FAM13C | 131 |
| 19 | 6161919 | 6161957 | ACSBG2 | 131 |
| 7 | 123388639 | 123389737 | WASL | 131 |
| 17 | 54929418 | 54929848 | DGKE | 131 |
| 6 | 24359800 | 24360441 | DCDC2 | 131 |
| 6 | 24359800 | 24360441 | KAAG1 | 131 |
| 19 | 8854702 | 8856087 | OR2Z1 | 131 |
| 3 | 29664694 | 29665135 | RBMS3 | 131 |
| 4 | 46816608 | 46817300 | COX7B2 | 131 |
| 19 | 13945847 | 13951546 | NANOS3 | 131 |
| 4 | 40244502 | 40245700 | RHOH | 131 |
| 9 | 134247987 | 134249597 | PRRC2B | 131 |
| 6 | 27143209 | 27143868 | HIST1H2BK | 131 |
| 1 | 102945271 | 102945851 | OLFM3 | 131 |
| 22 | 29188321 | 29189229 | XBP1 | 131 |
| 17 | 48783824 | 48785101 | ANKRD40 | 131 |
| 11 | 16759812 | 16760568 | C11orf58 | 131 |
| 17 | 78710874 | 78711476 | RPTOR | 131 |
| 21 | 45625529 | 45628527 | ICOSLG | 131 |
| 19 | 45257100 | 45257804 | BCL3 | 131 |
| 19 | 19570768 | 19572008 | GATAD2A | 131 |
| 1 | 145712000 | 145712608 | CD160 | 131 |
| 15 | 102064608 | 102065248 | PCSK6 | 131 |
| 13 | 84248447 | 84249184 | SLITRK1 | 131 |
| 12 | 54808141 | 54809041 | ITGA5 | 131 |
| 11 | 58321270 | 58321818 | LPXN | 131 |
| 9 | 126137798 | 126138500 | CRB2 | 131 |
| 10 | 78881462 | 78882125 | KCNMA1 | 131 |
| 22 | 36868727 | 36869561 | TXN2 | 131 |
| 8 | 12866066 | 12866663 | KIAA1456 | 131 |
| 10 | 98500596 | 98501462 | PIK3AP1 | 131 |
| 1 | 35325859 | 35325902 | C1orf212 | 131 |
| 1 | 55181006 | 55181940 | TTC4 | 130 |
| 1 | 16304917 | 16305313 | C1orf64 | 130 |
| 20 | 39134999 | 39135579 | MAFB | 130 |
| 2 | 32876861 | 32877486 | TTC27 | 130 |
| 7 | 141437385 | 141438607 | SSBP1 | 130 |
| 15 | 75321193 | 75322806 | PPCDC | 130 |
| 6 | 93921721 | 93922301 | EPHA7 | 130 |
| 17 | 60132237 | 60132886 | MED13 | 130 |
| 16 | 81459603 | 81460151 | CMIP | 130 |
| 16 | 84640036 | 84641359 | COTL1 | 130 |
| 19 | 44281533 | 44282231 | KCNN4 | 130 |
| 20 | 42578718 | 42579341 | TOX2 | 130 |
| 20 | 22470574 | 22472769 | FOXA2 | 130 |
| 19 | 7609560 | 7610829 | PNPLA6 | 130 |
| 11 | 130010670 | 130011168 | APLP2 | 130 |
| 17 | 56406567 | 56407116 | BZRAP1 | 130 |
| 6 | 49693216 | 49693696 | CRISP2 | 130 |
| 14 | 58892613 | 58893473 | KIAA0586 | 130 |
| 4 | 40806081 | 40806511 | NSUN7 | 130 |
| 3 | 106151835 | 106152585 | CBLB | 130 |
| 3 | 181663679 | 181664226 | SOX2 | 130 |
| 8 | 124408318 | 124409213 | ATAD2 | 130 |
| 1 | 42317542 | 42317986 | HIVEP3 | 130 |
| 9 | 20598893 | 20599338 | MLLT3 | 130 |
| 18 | 13293999 | 13294846 | C18orf1 | 130 |
| 1 | 8544375 | 8544906 | RERE | 130 |
| 3 | 188426372 | 188426767 | LPP | 130 |
| 7 | 45091023 | 45091728 | CCM2 | 130 |
| 17 | 59298047 | 59298515 | BCAS3 | 130 |
| 9 | 21334916 | 21335468 | KLHL9 | 130 |
| 3 | 182770939 | 182772391 | MCCC1 | 130 |
| 10 | 121047730 | 121049188 | GRK5 | 130 |
| 7 | 535672 | 537033 | PDGFA | 130 |
| 5 | 139991763 | 139992602 | TMCO6 | 130 |
| 10 | 73488884 | 73490371 | C10orf105 | 130 |
| 10 | 73488884 | 73490371 | CDH23 | 130 |
| 10 | 72267891 | 72269511 | KIAA1274 | 129 |
| 6 | 27805645 | 27806219 | HIST1H2BN | 129 |
| 6 | 154797003 | 154797602 | CNKSR3 | 129 |
| 14 | 93593650 | 93593680 | ITPK1 | 129 |
| 15 | 50923999 | 50924612 | TRPM7 | 129 |
| 11 | 97645649 | 97646331 | CNTN5 | 129 |
| 7 | 155394658 | 155396529 | RBM33 | 129 |
| 6 | 141701875 | 141702509 | NMBR | 129 |
| 11 | 61770838 | 61772004 | FTH1 | 129 |
| 11 | 125481633 | 125482090 | STT3A | 129 |
| 5 | 103037658 | 103038355 | NUDT12 | 129 |
| 6 | 43891194 | 43892494 | C6orf223 | 129 |
| 2 | 160142259 | 160142861 | WDSUB1 | 129 |
| 4 | 3877468 | 3877788 | ADRA2C | 129 |
| 20 | 30134672 | 30135621 | HM13 | 129 |
| 13 | 71083286 | 71083935 | KLHL1 | 129 |
| 3 | 13454596 | 13455095 | NUP210 | 129 |
| 1 | 200838881 | 200839644 | C1orf106 | 129 |
| 12 | 133338703 | 133339420 | ANKLE2 | 129 |
| 2 | 54342335 | 54343208 | ACYP2 | 129 |
| 5 | 145807448 | 145808296 | TCERG1 | 129 |
| 14 | 75277422 | 75278060 | YLPM1 | 129 |
| 10 | 93301683 | 93301700 | PPP1R3C | 129 |
| 1 | 44383374 | 44384224 | ST3GAL3 | 129 |
| 10 | 1289826 | 1290755 | ADARB2 | 129 |
| 2 | 9679082 | 9679656 | ADAM17 | 129 |
| 5 | 17586658 | 17587293 | BASP1 | 129 |
| 13 | 67931708 | 67932236 | PCDH9 | 129 |
| 2 | 102234436 | 102234747 | MAP4K4 | 129 |
| 5 | 32211894 | 32212032 | GOLPH3 | 129 |
| 1 | 118075904 | 118076471 | FAM46C | 129 |
| 19 | 10379931 | 10382924 | ICAM1 | 129 |
| 3 | 191338326 | 191338991 | PYDC2 | 129 |
| 12 | 98897490 | 98898111 | TMPO | 129 |
| 3 | 158185514 | 158186154 | RSRC1 | 129 |
| 16 | 87064152 | 87064550 | C16orf95 | 129 |
| 12 | 120729840 | 120730544 | PXN | 129 |
| 10 | 43615742 | 43616680 | RET | 129 |
| 1 | 144029466 | 144030229 | FAM72D | 128 |
| 8 | 96227214 | 96227662 | C8orf37 | 128 |
| 10 | 3785698 | 3786361 | KLF6 | 128 |
| 1 | 99138140 | 99138606 | SNX7 | 128 |
| 17 | 38443500 | 38444575 | CDC6 | 128 |
| 12 | 8129525 | 8130098 | SLC2A3 | 128 |
| 19 | 50379324 | 50381408 | TBC1D17 | 128 |
| 12 | 133291549 | 133291563 | PGAM5 | 128 |
| 2 | 54785393 | 54786282 | SPTBN1 | 128 |
| 12 | 40643817 | 40644488 | LRRK2 | 128 |
| 3 | 110003726 | 110004369 | PVRL3 | 128 |
| 5 | 68474529 | 68475733 | CENPH | 128 |
| 15 | 76946353 | 76946853 | SCAPER | 128 |
| 7 | 9956493 | 9957203 | NDUFA4 | 128 |
| 15 | 45746522 | 45747613 | C15orf48 | 128 |
| 4 | 24975264 | 24975958 | CCDC149 | 128 |
| 4 | 139937460 | 139938213 | CCRN4L | 128 |
| 6 | 73397332 | 73397941 | KCNQ5 | 128 |
| 3 | 160154117 | 160155262 | TRIM59 | 128 |
| 4 | 6919044 | 6919853 | TBC1D14 | 128 |
| 6 | 35294062 | 35294761 | PPARD | 128 |
| 19 | 29208080 | 29208583 | UQCRFS1 | 128 |
| 7 | 87935748 | 87936391 | STEAP4 | 128 |
| 13 | 90756489 | 90757495 | GPC5 | 128 |
| 4 | 5872088 | 5873511 | CRMP1 | 128 |
| 2 | 62752029 | 62752713 | TMEM17 | 128 |
| 17 | 5000171 | 5000669 | ZFP3 | 128 |
| 8 | 101328614 | 101328979 | RNF19A | 128 |
| 6 | 152638240 | 152638602 | SYNE1 | 128 |
| 11 | 65419306 | 65420998 | RELA | 128 |
| 17 | 19119006 | 19120610 | EPN2 | 128 |
| 11 | 118305273 | 118307274 | MLL | 128 |
| 15 | 49099383 | 49099882 | CEP152 | 128 |
| 13 | 114461390 | 114461977 | FAM70B | 127 |
| 3 | 112325126 | 112325599 | CCDC80 | 127 |
| 17 | 29922930 | 29924379 | RAB11FIP4 | 127 |
| 19 | 1359762 | 1361812 | MUM1 | 127 |
| 18 | 60769489 | 60770125 | BCL2 | 127 |
| 4 | 45235581 | 45236069 | GNPDA2 | 127 |
| 10 | 97740724 | 97741250 | CC2D2B | 127 |
| 15 | 93460238 | 93461141 | CHD2 | 127 |
| 10 | 75488660 | 75489429 | SEC24C | 127 |
| 19 | 51098007 | 51098239 | LRRC4B | 127 |
| 2 | 98463160 | 98463687 | TMEM131 | 127 |
| 7 | 125201710 | 125202537 | POT1 | 127 |
| 17 | 75473503 | 75474117 | SEPT9 | 127 |
| 1 | 521382 | 521709 | OR4F3 | 127 |
| 4 | 81122834 | 81123790 | PRDM8 | 127 |
| 1 | 39997970 | 39999201 | BMP8A | 127 |
| 13 | 91909512 | 91910426 | GPC5 | 127 |
| 4 | 18000668 | 18001250 | LCORL | 127 |
| 7 | 26354818 | 26355916 | SNX10 | 127 |
| 6 | 165740224 | 165740652 | C6orf118 | 127 |
| 11 | 126526520 | 126526964 | KIRREL3 | 127 |
| 12 | 49628062 | 49629489 | TUBA1C | 127 |
| 12 | 46127569 | 46128277 | ARID2 | 127 |
| 4 | 141159491 | 141160161 | SCOC | 127 |
| 17 | 66014910 | 66015693 | C17orf58 | 127 |
| 3 | 41460989 | 41461540 | ULK4 | 127 |
| 13 | 50481714 | 50482250 | SPRYD7 | 127 |
| 3 | 170587376 | 170588600 | RPL22L1 | 127 |
| 4 | 37548134 | 37548694 | C4orf19 | 127 |
| 19 | 33751328 | 33753186 | SLC7A10 | 127 |
| 12 | 3238921 | 3240534 | TSPAN9 | 127 |
| 1 | 169074772 | 169075260 | ATP1B1 | 127 |
| 1 | 202512430 | 202512955 | PPP1R12B | 127 |
| 3 | 197875916 | 197876492 | FAM157A | 127 |
| 19 | 5122590 | 5122600 | KDM4B | 127 |
| 3 | 158344328 | 158344861 | GFM1 | 127 |
| 18 | 41581469 | 41581956 | SETBP1 | 127 |
| 3 | 149469773 | 149470709 | COMMD2 | 127 |
| 5 | 179927142 | 179927671 | CNOT6 | 127 |
| 8 | 42188122 | 42188982 | IKBKB | 127 |
| 15 | 71044040 | 71044675 | UACA | 127 |
| 11 | 126618235 | 126618670 | KIRREL3 | 127 |
| 2 | 174889994 | 174890629 | SP3 | 126 |
| 16 | 84799534 | 84800436 | USP10 | 126 |
| 17 | 42568553 | 42568972 | GPATCH8 | 126 |
| 13 | 31612979 | 31613516 | C13orf26 | 126 |
| 9 | 101337994 | 101338550 | GABBR2 | 126 |
| 1 | 244265175 | 244265979 | ZNF238 | 126 |
| 3 | 36820866 | 36821279 | DCLK3 | 126 |
| 13 | 92001192 | 92001611 | GPC5 | 126 |
| 13 | 89914055 | 89915552 | SLITRK5 | 126 |
| 15 | 30504196 | 30504632 | CHRFAM7A | 126 |
| 5 | 177665939 | 177667133 | COL23A1 | 126 |
| 12 | 57881124 | 57882103 | MARS | 126 |
| 2 | 112105237 | 112105898 | BCL2L11 | 126 |
| 13 | 92170470 | 92171112 | GPC5 | 126 |
| 5 | 111434523 | 111435083 | C5orf13 | 126 |
| 14 | 104179989 | 104181643 | ZFYVE21 | 126 |
| 2 | 240162207 | 240162736 | HDAC4 | 126 |
| 7 | 30068245 | 30068862 | PLEKHA8 | 126 |
| 13 | 50367645 | 50367809 | KPNA3 | 126 |
| 19 | 8631840 | 8633749 | MYO1F | 126 |
| 15 | 101557492 | 101557970 | LRRK1 | 126 |
| 9 | 126964123 | 126965004 | NEK6 | 126 |
| 3 | 38022471 | 38022810 | CTDSPL | 126 |
| 5 | 90282796 | 90283311 | GPR98 | 126 |
| 15 | 36066626 | 36067060 | ATPBD4 | 126 |
| 10 | 101941768 | 101941787 | ERLIN1 | 126 |
| 16 | 88766884 | 88768125 | RNF166 | 126 |
| 6 | 137571269 | 137571806 | IFNGR1 | 126 |
| 10 | 81422508 | 81423102 | SFTPA1 | 126 |
| 5 | 131949125 | 131949561 | RAD50 | 126 |
| 1 | 167619236 | 167620049 | RCSD1 | 125 |
| 5 | 1539754 | 1540514 | LPCAT1 | 125 |
| 5 | 69746468 | 69746975 | SMN1 | 125 |
| 20 | 7036321 | 7036910 | BMP2 | 125 |
| 18 | 76635708 | 76636683 | SALL3 | 125 |
| 2 | 70418691 | 70419268 | C2orf42 | 125 |
| 6 | 27642657 | 27643302 | HIST1H2BL | 125 |
| 5 | 147194275 | 147194771 | SPINK1 | 125 |
| 1 | 58989160 | 58989581 | OMA1 | 125 |
| 6 | 75993809 | 75995114 | TMEM30A | 125 |
| 17 | 42647300 | 42647401 | FZD2 | 125 |
| 15 | 75464025 | 75465045 | C15orf39 | 125 |
| 7 | 54955641 | 54956578 | SEC61G | 125 |
| 5 | 68571628 | 68572207 | CDK7 | 125 |
| 6 | 27791557 | 27792665 | HIST1H4J | 125 |
| 3 | 15688356 | 15688880 | BTD | 125 |
| 19 | 50057265 | 50059188 | PRRG2 | 125 |
| 5 | 150479090 | 150480044 | TNIP1 | 125 |
| 16 | 374092 | 375659 | AXIN1 | 125 |
| 10 | 62511858 | 62512650 | CDK1 | 125 |
| 8 | 126524939 | 126525840 | TRIB1 | 125 |
| 1 | 206289995 | 206290453 | C1orf186 | 125 |
| 2 | 106392241 | 106393439 | NCK2 | 125 |
| 6 | 43351651 | 43352346 | ZNF318 | 125 |
| 12 | 52418573 | 52419377 | NR4A1 | 125 |
| 15 | 30579016 | 30579652 | CHRFAM7A | 125 |
| 13 | 82670469 | 82671257 | SPRY2 | 125 |
| 10 | 82437528 | 82438010 | SH2D4B | 125 |
| 17 | 73333444 | 73334359 | GRB2 | 125 |
| 1 | 76210052 | 76210256 | ACADM | 125 |
| 1 | 11880774 | 11881501 | CLCN6 | 125 |
| 11 | 82264480 | 82265050 | FAM181B | 125 |
| X | 41093707 | 41094202 | USP9X | 125 |
| 13 | 110352185 | 110352903 | IRS2 | 125 |
| 22 | 24639516 | 24640126 | GGT5 | 125 |
| 10 | 50395961 | 50396648 | C10orf128 | 125 |
| 3 | 195194078 | 195195068 | ACAP2 | 125 |
| 16 | 75338569 | 75338868 | CFDP1 | 125 |
| 3 | 26875774 | 26876153 | LRRC3B | 125 |
| 7 | 21955096 | 21956219 | CDCA7L | 125 |
| 11 | 125759172 | 125759873 | HYLS1 | 125 |
| 11 | 125759172 | 125759873 | PUS3 | 125 |
| 20 | 35311018 | 35311748 | NDRG3 | 125 |
| 16 | 85960801 | 85961328 | IRF8 | 125 |
| 19 | 46458689 | 46459380 | NOVA2 | 125 |
| 10 | 22622721 | 22624799 | SPAG6 | 124 |
| 10 | 134401048 | 134401440 | INPP5A | 124 |
| 9 | 5629166 | 5629899 | KIAA1432 | 124 |
| 11 | 43380032 | 43381175 | TTC17 | 124 |
| 20 | 60876444 | 60877340 | ADRM1 | 124 |
| 13 | 49079982 | 49080512 | RCBTB2 | 124 |
| 15 | 43027655 | 43028560 | CDAN1 | 124 |
| 8 | 100873493 | 100874551 | VPS13B | 124 |
| 16 | 84602292 | 84603147 | COTL1 | 124 |
| 12 | 49365871 | 49366803 | WNT10B | 124 |
| 4 | 39706097 | 39708318 | UBE2K | 124 |
| 6 | 108395200 | 108395958 | OSTM1 | 124 |
| 15 | 57511730 | 57512316 | TCF12 | 124 |
| 7 | 72630215 | 72631094 | NSUN5 | 124 |
| 10 | 38264906 | 38265906 | ZNF25 | 124 |
| 1 | 86821911 | 86822348 | ODF2L | 124 |
| 8 | 9444696 | 9445312 | TNKS | 124 |
| 15 | 65970160 | 65970615 | DENND4A | 124 |
| 15 | 44768128 | 44768690 | CTDSPL2 | 124 |
| 1 | 43549714 | 43550319 | FAM183A | 124 |
| 9 | 102581522 | 102581927 | NR4A3 | 124 |
| 7 | 38398210 | 38399175 | TARP | 124 |
| 8 | 61822396 | 61823395 | CHD7 | 124 |
| 19 | 16704682 | 16705482 | MED26 | 124 |
| 5 | 53306318 | 53306949 | ARL15 | 124 |
| 4 | 4443841 | 4444416 | STX18 | 124 |
| 7 | 22143523 | 22144301 | CDCA7L | 124 |
| 4 | 6917987 | 6918757 | TBC1D14 | 124 |
| 12 | 10348877 | 10349417 | OLR1 | 124 |
| 9 | 27955706 | 27956140 | LINGO2 | 124 |
| 15 | 31320376 | 31321026 | TRPM1 | 124 |
| 15 | 73988542 | 73989133 | CD276 | 124 |
| X | 152991995 | 152992976 | ABCD1 | 124 |
| 4 | 99849257 | 99849907 | EIF4E | 124 |
| 10 | 27529821 | 27531405 | ACBD5 | 124 |
| 14 | 65141578 | 65142458 | PLEKHG3 | 123 |
| 7 | 12551162 | 12551792 | SCIN | 123 |
| 5 | 69206607 | 69209191 | SERF1A | 123 |
| 10 | 134401789 | 134404959 | INPP5A | 123 |
| 22 | 38465085 | 38465150 | PICK1 | 123 |
| 18 | 74191278 | 74192212 | ZNF516 | 123 |
| 22 | 22046089 | 22046661 | PPIL2 | 123 |
| 15 | 36940410 | 36940906 | C15orf41 | 123 |
| 22 | 38031936 | 38032585 | PDXP | 123 |
| 16 | 66550205 | 66551533 | TK2 | 123 |
| 6 | 149594295 | 149594922 | TAB2 | 123 |
| 6 | 108396018 | 108397019 | OSTM1 | 123 |
| 3 | 43541132 | 43541882 | ANO10 | 123 |
| 1 | 65192108 | 65192656 | RAVER2 | 123 |
| 6 | 83797533 | 83798161 | DOPEY1 | 123 |
| 15 | 67293083 | 67293656 | SMAD3 | 123 |
| 10 | 45920964 | 45922028 | ALOX5 | 123 |
| 11 | 129993071 | 129994127 | APLP2 | 123 |
| 3 | 52531811 | 52534153 | STAB1 | 123 |
| 8 | 134308681 | 134309542 | NDRG1 | 123 |
| 7 | 47534412 | 47535176 | TNS3 | 123 |
| 8 | 6185451 | 6186667 | MCPH1 | 123 |
| 3 | 90099871 | 90100495 | EPHA3 | 123 |
| 3 | 69421397 | 69421814 | FRMD4B | 123 |
| 10 | 129863018 | 129863779 | PTPRE | 123 |
| 2 | 232333674 | 232334316 | NCL | 123 |
| 6 | 106352269 | 106353013 | PRDM1 | 123 |
| 7 | 151535910 | 151536876 | PRKAG2 | 123 |
| 8 | 81805737 | 81806384 | ZNF704 | 123 |
| 3 | 196057460 | 196059305 | TM4SF19 | 123 |
| 8 | 22830899 | 22832078 | RHOBTB2 | 123 |
| 3 | 159560915 | 159561727 | SCHIP1 | 123 |
| 3 | 159560915 | 159561727 | IQCJ-SCHIP1 | 123 |
| 17 | 4650792 | 4651197 | TM4SF5 | 123 |
| 9 | 129233324 | 129233829 | FAM125B | 123 |
| 3 | 185654646 | 185657539 | TRA2B | 122 |
| 20 | 40037651 | 40038074 | CHD6 | 122 |
| 10 | 112366662 | 112367428 | SMC3 | 122 |
| 6 | 135542673 | 135543430 | MYB | 122 |
| 14 | 50365570 | 50366198 | ARF6 | 122 |
| 3 | 159032822 | 159033315 | SCHIP1 | 122 |
| 3 | 135914730 | 135916180 | MSL2 | 122 |
| 3 | 159032822 | 159033315 | IQCJ-SCHIP1 | 122 |
| 15 | 31679727 | 31680209 | KLF13 | 122 |
| 5 | 129834472 | 129835058 | CHSY3 | 122 |
| 17 | 5137680 | 5138625 | C17orf87 | 122 |
| 18 | 4510615 | 4511307 | DLGAP1 | 122 |
| 1 | 66220447 | 66221108 | PDE4B | 122 |
| 21 | 45253856 | 45255944 | AGPAT3 | 122 |
| 3 | 117331152 | 117331895 | LSAMP | 122 |
| 18 | 29700955 | 29701545 | RNF138 | 122 |
| 14 | 67954658 | 67955697 | TMEM229B | 122 |
| 13 | 77632592 | 77633314 | MYCBP2 | 122 |
| 11 | 56728106 | 56728703 | OR5AK2 | 122 |
| 15 | 76599569 | 76600289 | ETFA | 122 |
| 18 | 13069630 | 13070367 | CEP192 | 122 |
| 19 | 12791661 | 12792042 | DHPS | 122 |
| 19 | 55105764 | 55106161 | LILRA1 | 122 |
| 4 | 9341946 | 9343999 | LOC728405 | 122 |
| 2 | 191884734 | 191885755 | STAT1 | 122 |
| 9 | 19255944 | 19257235 | DENND4C | 122 |
| 12 | 25806569 | 25807094 | IFLTD1 | 122 |
| 19 | 33771529 | 33772357 | CEBPA | 122 |
| 7 | 17285671 | 17286281 | AHR | 122 |
| 8 | 41671325 | 41671911 | ANK1 | 122 |
| 13 | 90026298 | 90027144 | SLITRK5 | 122 |
| 2 | 64419965 | 64420311 | PELI1 | 122 |
| 10 | 76025852 | 76026367 | ADK | 122 |
| 3 | 97473305 | 97474082 | ARL6 | 122 |
| 12 | 6569776 | 6570691 | TAPBPL | 122 |
| 12 | 6569776 | 6570691 | VAMP1 | 122 |
| 14 | 97706266 | 97706819 | VRK1 | 122 |
| 2 | 232367858 | 232368272 | NMUR1 | 122 |
| X | 70752451 | 70753315 | OGT | 122 |
| 17 | 66856537 | 66857026 | ABCA8 | 122 |
| 3 | 142837443 | 142838490 | CHST2 | 122 |
| 9 | 133801210 | 133801885 | FIBCD1 | 121 |
| 3 | 57904593 | 57905312 | SLMAP | 121 |
| 8 | 97518689 | 97519297 | SDC2 | 121 |
| 11 | 88070302 | 88071197 | CTSC | 121 |
| 17 | 73091510 | 73093031 | SLC16A5 | 121 |
| 4 | 4655865 | 4656847 | STX18 | 121 |
| 5 | 39209239 | 39210395 | FYB | 121 |
| 3 | 32665669 | 32666203 | DYNC1LI1 | 121 |
| 22 | 24836423 | 24837070 | ADORA2A | 121 |
| 3 | 196794301 | 196794898 | DLG1 | 121 |
| 4 | 15095019 | 15095790 | CPEB2 | 121 |
| 3 | 196344417 | 196345838 | LRRC33 | 121 |
| 15 | 63356657 | 63357244 | TPM1 | 121 |
| 10 | 94459002 | 94459677 | HHEX | 121 |
| 11 | 69330031 | 69330550 | CCND1 | 121 |
| 12 | 49411761 | 49412885 | PRKAG1 | 121 |
| X | 154254995 | 154255472 | FUNDC2 | 121 |
| 19 | 16683239 | 16684254 | SLC35E1 | 121 |
| 8 | 19088128 | 19088649 | SH2D4A | 121 |
| 7 | 81632874 | 81633475 | CACNA2D1 | 121 |
| X | 108779917 | 108780540 | NXT2 | 121 |
| 13 | 78425983 | 78426804 | EDNRB | 121 |
| 3 | 155987516 | 155988123 | KCNAB1 | 121 |
| 7 | 41805580 | 41805659 | INHBA | 121 |
| 11 | 35873548 | 35874063 | LDLRAD3 | 121 |
| 18 | 20877287 | 20878114 | C18orf45 | 121 |
| 11 | 69526902 | 69527729 | FGF19 | 121 |
| 16 | 86413661 | 86414144 | FOXF1 | 121 |
| 5 | 77793350 | 77793995 | LHFPL2 | 121 |
| 15 | 38368855 | 38369366 | TMCO5A | 120 |
| 14 | 59509137 | 59509704 | DAAM1 | 120 |
| 8 | 107731701 | 107732436 | OXR1 | 120 |
| 21 | 21215597 | 21216087 | NCAM2 | 120 |
| 7 | 72930504 | 72930598 | BAZ1B | 120 |
| 12 | 39375226 | 39375970 | CPNE8 | 120 |
| 4 | 2591478 | 2593425 | FAM193A | 120 |
| 11 | 130024872 | 130025613 | ST14 | 120 |
| 6 | 111194962 | 111196605 | AMD1 | 120 |
| 17 | 2694502 | 2695550 | RAP1GAP2 | 120 |
| 10 | 73511290 | 73513066 | C10orf54 | 120 |
| 10 | 73534708 | 73535622 | C10orf54 | 120 |
| 10 | 73511290 | 73513066 | CDH23 | 120 |
| 10 | 73534708 | 73535622 | CDH23 | 120 |
| 1 | 76620803 | 76621454 | ST6GALNAC3 | 120 |
| 20 | 3410868 | 3411298 | C20orf194 | 120 |
| 4 | 29671365 | 29671953 | PCDH7 | 120 |
| 13 | 77645956 | 77646553 | MYCBP2 | 120 |
| 2 | 88330687 | 88331215 | KRCC1 | 120 |
| 5 | 57375468 | 57375987 | PLK2 | 120 |
| 16 | 89252492 | 89253597 | CDH15 | 120 |
| 12 | 52419589 | 52419954 | NR4A1 | 120 |
| 3 | 661845 | 662029 | CHL1 | 120 |
| 11 | 268399 | 269131 | ATHL1 | 120 |
| 5 | 14681333 | 14681811 | FAM105B | 120 |
| 8 | 146227698 | 146228678 | C8orf33 | 120 |
| 12 | 131713991 | 131714595 | GPR133 | 120 |
| 14 | 47488621 | 47489162 | MDGA2 | 120 |
| 12 | 44673145 | 44673864 | TMEM117 | 120 |
| 17 | 75877306 | 75878305 | TNRC6C | 120 |
| 15 | 96828618 | 96829464 | NR2F2 | 120 |
| 19 | 17219710 | 17221738 | MYO9B | 120 |
| 12 | 53397184 | 53397890 | EIF4B | 120 |
| 6 | 28197464 | 28198070 | ZNF193 | 120 |
| 17 | 25857073 | 25857674 | KSR1 | 120 |
| 8 | 96099956 | 96100783 | PLEKHF2 | 120 |
| 3 | 5057240 | 5058248 | BHLHE40 | 120 |
| 7 | 158839431 | 158839956 | VIPR2 | 120 |
| 9 | 68362722 | 68363308 | ANKRD20A3 | 120 |
| 17 | 55038120 | 55038634 | COIL | 120 |
| 22 | 38465889 | 38466414 | PICK1 | 120 |
| 4 | 3877860 | 3878478 | ADRA2C | 120 |
| 10 | 101497270 | 101497974 | COX15 | 120 |
| 10 | 101497270 | 101497974 | CUTC | 120 |
| 17 | 10018035 | 10018931 | GAS7 | 120 |
| 6 | 105565512 | 105565992 | BVES | 120 |
| 10 | 33950512 | 33951147 | NRP1 | 120 |
| 7 | 144189222 | 144189757 | TPK1 | 120 |
| 12 | 42759692 | 42760340 | PPHLN1 | 120 |
| 6 | 111626058 | 111626558 | REV3L | 120 |
| 9 | 68411867 | 68415495 | ANKRD20A3 | 120 |
| 13 | 87369138 | 87369854 | SLITRK5 | 119 |
| 22 | 36890512 | 36890570 | FOXRED2 | 119 |
| 7 | 3859596 | 3860014 | SDK1 | 119 |
| 14 | 101586449 | 101587821 | RTL1 | 119 |
| 1 | 111501912 | 111502476 | LRIF1 | 119 |
| 2 | 74698785 | 74701207 | MRPL53 | 119 |
| 13 | 77811888 | 77812763 | MYCBP2 | 119 |
| 21 | 20586138 | 20586636 | TMPRSS15 | 119 |
| 19 | 17828422 | 17829294 | MAP1S | 119 |
| 7 | 128579171 | 128581877 | IRF5 | 119 |
| 2 | 31541416 | 31542237 | EHD3 | 119 |
| 16 | 75133166 | 75134301 | ZNRF1 | 119 |
| X | 48979837 | 48980701 | GPKOW | 119 |
| 11 | 128778414 | 128779141 | C11orf45 | 119 |
| 11 | 128778414 | 128779141 | KCNJ5 | 119 |
| 1 | 208909933 | 208910419 | PLXNA2 | 119 |
| 7 | 32027503 | 32027925 | PDE1C | 119 |
| 8 | 28902729 | 28903316 | HMBOX1 | 119 |
| 17 | 72736820 | 72738956 | RAB37 | 119 |
| 2 | 84961157 | 84961524 | DNAH6 | 119 |
| 17 | 65952369 | 65953449 | BPTF | 119 |
| 2 | 217339734 | 217340222 | SMARCAL1 | 119 |
| 18 | 42078846 | 42079356 | SETBP1 | 119 |
| 14 | 81340684 | 81341358 | CEP128 | 119 |
| 20 | 5943945 | 5944538 | MCM8 | 119 |
| 9 | 4709921 | 4710917 | AK3 | 119 |
| 3 | 45208765 | 45209456 | CDCP1 | 119 |
| 6 | 150173415 | 150174045 | LRP11 | 119 |
| 10 | 105517100 | 105518036 | SH3PXD2A | 119 |
| 1 | 36626172 | 36627063 | MAP7D1 | 119 |
| 22 | 39040723 | 39040883 | LOC646851 | 119 |
| 3 | 193854359 | 193855654 | HES1 | 119 |
| 6 | 108038658 | 108038683 | SCML4 | 119 |
| 2 | 37875290 | 37876327 | CDC42EP3 | 119 |
| 14 | 90722514 | 90723431 | PSMC1 | 119 |
| 20 | 3326255 | 3326790 | C20orf194 | 119 |
| 8 | 103688393 | 103689425 | KLF10 | 119 |
| 19 | 13291930 | 13293262 | IER2 | 119 |
| 6 | 25609306 | 25609850 | LRRC16A | 119 |
| 18 | 24376928 | 24377594 | AQP4 | 119 |
| 5 | 148191764 | 148192541 | ADRB2 | 118 |
| 9 | 137212555 | 137212993 | RXRA | 118 |
| 16 | 85962743 | 85967160 | IRF8 | 118 |
| 8 | 71129275 | 71129810 | NCOA2 | 118 |
| 1 | 114369137 | 114369719 | PTPN22 | 118 |
| 2 | 10522616 | 10523730 | HPCAL1 | 118 |
| 10 | 29594979 | 29595577 | LYZL1 | 118 |
| 5 | 43064547 | 43065336 | C5orf39 | 118 |
| 8 | 145002021 | 145003123 | PLEC | 118 |
| 1 | 36707767 | 36708349 | THRAP3 | 118 |
| 17 | 7482731 | 7483490 | CD68 | 118 |
| 19 | 51634809 | 51635232 | SIGLEC9 | 118 |
| 15 | 80319733 | 80319749 | ZFAND6 | 118 |
| 3 | 141594099 | 141594315 | ATP1B3 | 118 |
| 17 | 44793457 | 44794092 | NSF | 118 |
| 17 | 80551139 | 80553187 | FOXK2 | 118 |
| 11 | 69390389 | 69391356 | CCND1 | 118 |
| 18 | 375120 | 375237 | COLEC12 | 118 |
| 5 | 16758991 | 16759430 | MYO10 | 118 |
| 1 | 54950323 | 54951051 | ACOT11 | 118 |
| 19 | 4790582 | 4792328 | FEM1A | 118 |
| 20 | 48781855 | 48782290 | CEBPB | 118 |
| 5 | 140892219 | 140892887 | PCDHGB5 | 118 |
| 5 | 140892219 | 140892887 | PCDHGB1 | 118 |
| 5 | 140892219 | 140892887 | PCDHGA8 | 118 |
| 5 | 140892219 | 140892887 | PCDHGA12 | 118 |
| 5 | 140892219 | 140892887 | PCDHGA11 | 118 |
| 5 | 140892219 | 140892887 | PCDHGA10 | 118 |
| 5 | 140892219 | 140892887 | PCDHGC3 | 118 |
| 5 | 140892219 | 140892887 | PCDHGA6 | 118 |
| 5 | 140892219 | 140892887 | PCDHGA4 | 118 |
| 5 | 140892219 | 140892887 | PCDHGB6 | 118 |
| 5 | 140892219 | 140892887 | PCDHGB2 | 118 |
| 5 | 140892219 | 140892887 | PCDHGA1 | 118 |
| 5 | 140892219 | 140892887 | PCDHGC5 | 118 |
| 5 | 140892219 | 140892887 | PCDHGA7 | 118 |
| 5 | 140892219 | 140892887 | PCDHGB7 | 118 |
| 5 | 140892219 | 140892887 | PCDHGA9 | 118 |
| 5 | 140892219 | 140892887 | PCDHGA3 | 118 |
| 5 | 140892219 | 140892887 | PCDHGB3 | 118 |
| 5 | 140892219 | 140892887 | PCDHGA2 | 118 |
| 5 | 140892219 | 140892887 | PCDHGA5 | 118 |
| 5 | 140892219 | 140892887 | PCDHGB4 | 118 |
| 5 | 140892219 | 140892887 | PCDHGC4 | 118 |
| 13 | 89871101 | 89871961 | SLITRK5 | 118 |
| 1 | 88292039 | 88292303 | LMO4 | 118 |
| 6 | 167070568 | 167070918 | RPS6KA2 | 118 |
| 6 | 113107141 | 113107696 | RFPL4B | 118 |
| 15 | 67751718 | 67752193 | IQCH | 118 |
| 5 | 123987224 | 123987840 | ZNF608 | 118 |
| 11 | 126153796 | 126154534 | TIRAP | 117 |
| 2 | 91714020 | 91715328 | RPIA | 117 |
| 3 | 37387069 | 37387527 | GOLGA4 | 117 |
| 11 | 65185734 | 65185861 | FRMD8 | 117 |
| 4 | 34457486 | 34458016 | ARAP2 | 117 |
| 3 | 163079105 | 163079803 | SI | 117 |
| 9 | 7148892 | 7149718 | KDM4C | 117 |
| 1 | 203575984 | 203576253 | ATP2B4 | 117 |
| 16 | 77756343 | 77757204 | NUDT7 | 117 |
| 18 | 18679478 | 18680069 | ROCK1 | 117 |
| 3 | 46967847 | 46969302 | CCDC12 | 117 |
| 3 | 194459122 | 194459579 | FAM43A | 117 |
| 4 | 40126854 | 40127622 | N4BP2 | 117 |
| 12 | 65612294 | 65612941 | LEMD3 | 117 |
| 6 | 58287250 | 58288424 | PRIM2 | 117 |
| 12 | 10826352 | 10827422 | STYK1 | 117 |
| 6 | 110726061 | 110726472 | DDO | 117 |
| 14 | 97742904 | 97743322 | VRK1 | 117 |
| 1 | 117909720 | 117910278 | MAN1A2 | 117 |
| 13 | 41634362 | 41634982 | WBP4 | 117 |
| 7 | 99966598 | 99968018 | PILRA | 117 |
| 10 | 119221090 | 119221745 | EMX2 | 117 |
| 9 | 6512655 | 6513344 | UHRF2 | 117 |
| 3 | 15689194 | 15689907 | BTD | 117 |
| 11 | 57251543 | 57252697 | SLC43A1 | 117 |
| 6 | 139210227 | 139210683 | ECT2L | 117 |
| 11 | 126153143 | 126153708 | TIRAP | 117 |
| 3 | 17487842 | 17488579 | TBC1D5 | 117 |
| 13 | 82779921 | 82779936 | SLITRK1 | 117 |
| 19 | 47731334 | 47732518 | BBC3 | 117 |
| 17 | 38478458 | 38479505 | RARA | 117 |
| 11 | 100865854 | 100866280 | TMEM133 | 117 |
| 5 | 131916661 | 131917383 | RAD50 | 116 |
| 19 | 58975809 | 58976904 | ZNF132 | 116 |
| 6 | 163834263 | 163834817 | QKI | 116 |
| 9 | 3919861 | 3920332 | GLIS3 | 116 |
| 1 | 12718708 | 12718929 | AADACL4 | 116 |
| 5 | 140937143 | 140937516 | DIAPH1 | 116 |
| 21 | 40734701 | 40734839 | WRB | 116 |
| 10 | 12647358 | 12648034 | CAMK1D | 116 |
| 10 | 28809959 | 28810497 | WAC | 116 |
| 7 | 92462081 | 92463620 | CDK6 | 116 |
| 6 | 148688028 | 148689297 | SASH1 | 116 |
| 10 | 73514590 | 73515396 | C10orf54 | 116 |
| 11 | 85767988 | 85768693 | PICALM | 116 |
| 10 | 73514590 | 73515396 | CDH23 | 116 |
| 16 | 66994848 | 66995703 | CES3 | 116 |
| 6 | 142707678 | 142708294 | GPR126 | 116 |
| 6 | 54074668 | 54075208 | MLIP | 116 |
| 21 | 44510895 | 44511615 | CBS | 116 |
| 19 | 1252000 | 1252830 | MIDN | 116 |
| 3 | 57967140 | 57968290 | FLNB | 116 |
| 11 | 113746334 | 113747358 | USP28 | 116 |
| 19 | 5138337 | 5140232 | KDM4B | 116 |
| 11 | 94951600 | 94952252 | SESN3 | 116 |
| 3 | 188054584 | 188055313 | LPP | 116 |
| 15 | 64028586 | 64029261 | HERC1 | 116 |
| 3 | 197632148 | 197633251 | IQCG | 116 |
| 1 | 80138079 | 80138534 | ELTD1 | 116 |
| 9 | 16659937 | 16660397 | BNC2 | 116 |
| 5 | 17443268 | 17444175 | BASP1 | 116 |
| 15 | 57058177 | 57058816 | ZNF280D | 116 |
| 16 | 31212900 | 31214479 | PYCARD | 116 |
| 6 | 44528372 | 44529227 | CDC5L | 116 |
| 1 | 65776838 | 65777295 | DNAJC6 | 116 |
| 13 | 83140866 | 83141326 | SLITRK1 | 115 |
| 12 | 41863141 | 41864031 | PDZRN4 | 115 |
| 3 | 182928457 | 182929638 | MCF2L2 | 115 |
| 2 | 44576570 | 44577018 | PREPL | 115 |
| 2 | 55194721 | 55195303 | EML6 | 115 |
| 10 | 98739412 | 98740002 | C10orf12 | 115 |
| 19 | 8564698 | 8566324 | PRAM1 | 115 |
| 17 | 38514542 | 38515685 | GJD3 | 115 |
| 7 | 104600396 | 104601248 | MLL5 | 115 |
| 7 | 50578938 | 50579491 | DDC | 115 |
| 4 | 56262339 | 56262392 | TMEM165 | 115 |
| 7 | 98706595 | 98707321 | SMURF1 | 115 |
| 6 | 77629062 | 77629608 | HTR1B | 115 |
| 9 | 139997152 | 139997816 | MAN1B1 | 115 |
| 16 | 66440436 | 66441125 | BEAN1 | 115 |
| 12 | 40191850 | 40192908 | SLC2A13 | 115 |
| 7 | 41499243 | 41499776 | INHBA | 115 |
| 7 | 17247947 | 17248473 | AHR | 115 |
| 7 | 148842919 | 148844124 | ZNF398 | 115 |
| 19 | 58006635 | 58007190 | ZNF772 | 115 |
| 14 | 60771350 | 60771911 | PPM1A | 115 |
| 14 | 91828078 | 91828664 | CCDC88C | 115 |
| 8 | 107314336 | 107315443 | OXR1 | 115 |
| 1 | 161015096 | 161015911 | USF1 | 115 |
| X | 54665653 | 54666601 | GNL3L | 115 |
| 11 | 130023734 | 130024477 | ST14 | 115 |
| 16 | 67423744 | 67424579 | TPPP3 | 115 |
| 16 | 21963540 | 21965165 | UQCRC2 | 115 |
| 3 | 128646120 | 128647172 | ACAD9 | 115 |
| 21 | 44123626 | 44124752 | PDE9A | 115 |
| 13 | 80913816 | 80915015 | SPRY2 | 115 |
| 21 | 47933890 | 47934481 | DIP2A | 115 |
| 18 | 60766586 | 60767731 | BCL2 | 114 |
| 5 | 171537856 | 171538594 | STK10 | 114 |
| 19 | 44446840 | 44447542 | ZNF221 | 114 |
| 9 | 7085569 | 7086290 | KDM4C | 114 |
| 13 | 114827776 | 114828905 | RASA3 | 114 |
| 1 | 4067283 | 4068026 | C1orf174 | 114 |
| 12 | 46383212 | 46386209 | SCAF11 | 114 |
| 5 | 118581019 | 118581652 | DMXL1 | 114 |
| 1 | 120648698 | 120649013 | NOTCH2 | 114 |
| 19 | 1075035 | 1075483 | HMHA1 | 114 |
| 1 | 99337665 | 99338203 | LPPR5 | 114 |
| 20 | 22225015 | 22225453 | FOXA2 | 114 |
| 22 | 30819334 | 30820539 | MTFP1 | 114 |
| 17 | 28727604 | 28728123 | CPD | 114 |
| 3 | 142079193 | 142079778 | XRN1 | 114 |
| 4 | 2432466 | 2433220 | LOC402160 | 114 |
| 2 | 65057890 | 65058929 | SLC1A4 | 114 |
| 14 | 76008454 | 76009478 | BATF | 114 |
| 20 | 47388222 | 47389589 | PREX1 | 114 |
| 5 | 37043011 | 37043677 | NIPBL | 114 |
| 19 | 14624839 | 14625347 | GIPC1 | 114 |
| 17 | 80254949 | 80256822 | CSNK1D | 114 |
| 19 | 55538199 | 55539531 | GP6 | 114 |
| 6 | 24786913 | 24787640 | GMNN | 114 |
| 8 | 17124096 | 17124677 | VPS37A | 114 |
| 17 | 59321720 | 59322248 | BCAS3 | 114 |
| 22 | 38571884 | 38572833 | PLA2G6 | 114 |
| 7 | 16127867 | 16128491 | ISPD | 114 |
| 9 | 107890557 | 107890838 | SLC44A1 | 114 |
| 2 | 12197396 | 12198099 | LPIN1 | 114 |
| 5 | 32027119 | 32027519 | PDZD2 | 113 |
| 5 | 89661507 | 89662249 | CETN3 | 113 |
| 12 | 121018585 | 121019543 | POP5 | 113 |
| 1 | 45024829 | 45028474 | RNF220 | 113 |
| 1 | 57170976 | 57171502 | PRKAA2 | 113 |
| 13 | 83756794 | 83758826 | SLITRK1 | 113 |
| 4 | 140004540 | 140005569 | ELF2 | 113 |
| 4 | 3546061 | 3546452 | LRPAP1 | 113 |
| 21 | 35152030 | 35152587 | ITSN1 | 113 |
| 8 | 17779791 | 17781771 | PCM1 | 113 |
| 7 | 151534523 | 151535577 | PRKAG2 | 113 |
| 3 | 37059325 | 37059851 | MLH1 | 113 |
| 11 | 89609344 | 89610011 | TRIM64B | 113 |
| 12 | 41865525 | 41866221 | PDZRN4 | 113 |
| 10 | 64441111 | 64441547 | ADO | 113 |
| 10 | 72804829 | 72805999 | PCBD1 | 113 |
| 17 | 65853019 | 65854293 | BPTF | 113 |
| 16 | 57180927 | 57181887 | CPNE2 | 113 |
| 8 | 23145209 | 23146134 | R3HCC1 | 113 |
| 4 | 49182562 | 49183370 | CWH43 | 113 |
| 1 | 87248891 | 87249275 | SH3GLB1 | 113 |
| 2 | 175918827 | 175919247 | CHN1 | 113 |
| 14 | 70232541 | 70233255 | SRSF5 | 113 |
| 4 | 2787655 | 2789080 | SH3BP2 | 113 |
| 14 | 105526614 | 105528757 | GPR132 | 113 |
| 20 | 37062900 | 37064195 | RALGAPB | 113 |
| 17 | 73335026 | 73335794 | GRB2 | 112 |
| 7 | 74178485 | 74182439 | NCF1 | 112 |
| 12 | 124864519 | 124865489 | NCOR2 | 112 |
| 1 | 52749847 | 52750475 | ZFYVE9 | 112 |
| 7 | 16189222 | 16189701 | ISPD | 112 |
| 7 | 38234538 | 38234919 | STARD3NL | 112 |
| 3 | 119107575 | 119108270 | ARHGAP31 | 112 |
| 7 | 107543231 | 107543879 | DLD | 112 |
| 6 | 148573410 | 148574158 | SASH1 | 112 |
| 14 | 103415199 | 103417449 | CDC42BPB | 112 |
| 19 | 40925911 | 40928108 | PRX | 112 |
| 15 | 85934589 | 85935453 | AKAP13 | 112 |
| 2 | 11228892 | 11229501 | C2orf50 | 112 |
| 5 | 42991992 | 42993404 | C5orf39 | 112 |
| 12 | 108707555 | 108707878 | CMKLR1 | 112 |
| 6 | 166849797 | 166849903 | RPS6KA2 | 112 |
| 16 | 89682737 | 89683904 | DPEP1 | 112 |
| 3 | 129749677 | 129750235 | ALG1L2 | 112 |
| 4 | 156540160 | 156540711 | GUCY1A3 | 112 |
| 3 | 183621821 | 183623663 | PARL | 112 |
| 10 | 35933158 | 35933713 | FZD8 | 112 |
| 4 | 103352002 | 103352608 | NFKB1 | 112 |
| 10 | 75123384 | 75124021 | TTC18 | 112 |
| 10 | 104848102 | 104848609 | NT5C2 | 112 |
| 3 | 195180075 | 195180749 | ACAP2 | 112 |
| 12 | 6475485 | 6476167 | SCNN1A | 112 |
| 1 | 243511725 | 243511815 | SDCCAG8 | 112 |
| 6 | 36739290 | 36739404 | CPNE5 | 112 |
| 2 | 37233245 | 37233917 | HEATR5B | 112 |
| 13 | 85682255 | 85683347 | SLITRK6 | 112 |
| 19 | 28894491 | 28895102 | UQCRFS1 | 112 |
| 8 | 134309646 | 134310243 | NDRG1 | 112 |
| 15 | 101709508 | 101710432 | CHSY1 | 112 |
| 18 | 74844911 | 74846649 | MBP | 112 |
| 8 | 19362448 | 19363631 | CSGALNACT1 | 111 |
| 14 | 50469618 | 50470835 | C14orf182 | 111 |
| 19 | 54751090 | 54751846 | LILRB3 | 111 |
| 5 | 148669158 | 148669670 | AFAP1L1 | 111 |
| 9 | 42455184 | 42456060 | ANKRD20A2 | 111 |
| 3 | 101396408 | 101396649 | ZBTB11 | 111 |
| 6 | 116730226 | 116730947 | DSE | 111 |
| 12 | 41845784 | 41846383 | PDZRN4 | 111 |
| 3 | 24780362 | 24780872 | THRB | 111 |
| X | 39719226 | 39720515 | BCOR | 111 |
| 10 | 124653647 | 124654121 | FAM24A | 111 |
| 22 | 36863754 | 36864720 | TXN2 | 111 |
| 17 | 60462962 | 60463486 | EFCAB3 | 111 |
| 13 | 87027190 | 87027791 | SLITRK6 | 111 |
| 12 | 66569392 | 66569834 | IRAK3 | 111 |
| 8 | 67025000 | 67026397 | TRIM55 | 111 |
| 11 | 61209089 | 61209093 | SDHAF2 | 111 |
| 3 | 164354230 | 164354787 | SI | 111 |
| 14 | 52938766 | 52939328 | TXNDC16 | 111 |
| 7 | 5259583 | 5261029 | WIPI2 | 111 |
| 4 | 45277777 | 45278364 | GNPDA2 | 111 |
| 11 | 102401128 | 102401559 | MMP7 | 111 |
| 15 | 72209415 | 72209986 | MYO9A | 111 |
| 10 | 65428037 | 65428512 | REEP3 | 111 |
| 9 | 96267145 | 96267737 | FAM120A | 111 |
| 1 | 22086758 | 22087680 | USP48 | 111 |
| 2 | 9276050 | 9276673 | ASAP2 | 111 |
| 17 | 46560213 | 46560737 | HOXB1 | 111 |
| 18 | 43266736 | 43267671 | SLC14A2 | 111 |
| 12 | 124950267 | 124951370 | NCOR2 | 111 |
| 7 | 5897476 | 5897486 | OCM | 110 |
| 2 | 33097967 | 33098675 | LTBP1 | 110 |
| 1 | 8167170 | 8167542 | ERRFI1 | 110 |
| 2 | 3200203 | 3200300 | TSSC1 | 110 |
| 12 | 104680544 | 104681259 | TXNRD1 | 110 |
| 12 | 104680544 | 104681259 | EID3 | 110 |
| 5 | 54469571 | 54469937 | CDC20B | 110 |
| 3 | 50279179 | 50280057 | GNAI2 | 110 |
| 1 | 51217441 | 51217448 | FAF1 | 110 |
| 3 | 56788615 | 56789293 | ARHGEF3 | 110 |
| 1 | 62510370 | 62510962 | INADL | 110 |
| 14 | 65174943 | 65175759 | PLEKHG3 | 110 |
| 3 | 195780342 | 195781471 | TFRC | 110 |
| 19 | 45958765 | 45959872 | FOSB | 110 |
| 1 | 53400137 | 53400749 | SCP2 | 110 |
| 17 | 73771824 | 73772571 | GALK1 | 110 |
| 8 | 18643178 | 18643812 | PSD3 | 110 |
| 17 | 79258713 | 79263496 | SLC38A10 | 110 |
| 19 | 14628626 | 14630153 | DNAJB1 | 110 |
| 10 | 30627384 | 30627915 | MTPAP | 110 |
| 8 | 142159736 | 142160440 | DENND3 | 110 |
| 2 | 65527174 | 65528616 | SPRED2 | 110 |
| 1 | 152008774 | 152009585 | S100A11 | 110 |
| 16 | 78165200 | 78166494 | WWOX | 110 |
| 11 | 109974106 | 109974934 | ZC3H12C | 110 |
| 14 | 74221820 | 74222489 | C14orf43 | 110 |
| 15 | 44281946 | 44282486 | FRMD5 | 110 |
| 6 | 41684063 | 41685040 | TFEB | 109 |
| 17 | 37249227 | 37249246 | PLXDC1 | 109 |
| 5 | 86767273 | 86767839 | CCNH | 109 |
| X | 9309412 | 9309839 | TBL1X | 109 |
| 10 | 102295384 | 102296000 | HIF1AN | 109 |
| 1 | 703081 | 703707 | OR4F3 | 109 |
| 12 | 39679744 | 39680835 | KIF21A | 109 |
| 13 | 88670880 | 88671933 | SLITRK5 | 109 |
| 15 | 40384502 | 40385975 | BMF | 109 |
| 12 | 131762635 | 131763323 | GPR133 | 109 |
| 16 | 73187542 | 73187916 | ZFHX3 | 109 |
| 7 | 104584901 | 104585876 | MLL5 | 109 |
| 11 | 72542239 | 72543379 | ATG16L2 | 109 |
| X | 24037320 | 24037871 | KLHL15 | 109 |
| 10 | 30337866 | 30338729 | KIAA1462 | 109 |
| 16 | 75366622 | 75367461 | CFDP1 | 109 |
| 9 | 130186154 | 130186902 | ZNF79 | 109 |
| 12 | 105629741 | 105630332 | APPL2 | 109 |
| 2 | 10465076 | 10466115 | HPCAL1 | 109 |
| 7 | 50772329 | 50772966 | GRB10 | 108 |
| 19 | 39897437 | 39899349 | ZFP36 | 108 |
| 2 | 173117889 | 173118575 | DLX2 | 108 |
| 3 | 45672551 | 45672620 | LIMD1 | 108 |
| 14 | 74222535 | 74222565 | C14orf43 | 108 |
| 6 | 42896609 | 42897778 | CNPY3 | 108 |
| 14 | 64107266 | 64107911 | WDR89 | 108 |
| 5 | 169854332 | 169854963 | KCNIP1 | 108 |
| 19 | 16368239 | 16370423 | AP1M1 | 108 |
| 22 | 44578130 | 44578762 | PARVG | 108 |
| 15 | 78781116 | 78782091 | IREB2 | 108 |
| 18 | 54886379 | 54886889 | ST8SIA3 | 108 |
| 2 | 32235785 | 32236486 | MEMO1 | 108 |
| 2 | 191745378 | 191745859 | GLS | 108 |
| 1 | 205819991 | 205820583 | PM20D1 | 108 |
| 3 | 135856295 | 135856762 | PPP2R3A | 108 |
| 11 | 72150737 | 72152093 | CLPB | 108 |
| 13 | 86122122 | 86122775 | SLITRK6 | 108 |
| X | 52963650 | 52964880 | FAM156B | 108 |
| 8 | 95732991 | 95733597 | DPY19L4 | 107 |
| 15 | 40886168 | 40887132 | CASC5 | 107 |
| 1 | 17059759 | 17060713 | NBPF1 | 107 |
| 6 | 27806258 | 27807216 | HIST1H2AK | 107 |
| 15 | 69959801 | 69960772 | RPLP1 | 107 |
| 12 | 2725216 | 2726896 | CACNA1C | 107 |
| 2 | 54786464 | 54787750 | SPTBN1 | 107 |
| 3 | 196221375 | 196221970 | RNF168 | 107 |
| 10 | 104404006 | 104406495 | TRIM8 | 107 |
| 12 | 12860442 | 12861219 | APOLD1 | 107 |
| 15 | 102067463 | 102068713 | PCSK6 | 107 |
| 17 | 75471719 | 75471916 | SEPT9 | 107 |
| 10 | 98724583 | 98725515 | C10orf12 | 107 |
| X | 46763092 | 46763717 | PHF16 | 107 |
| 1 | 12843221 | 12843681 | PRAMEF1 | 106 |
| 4 | 153535296 | 153535819 | TMEM154 | 106 |
| 6 | 109950529 | 109951199 | AKD1 | 106 |
| 10 | 17031621 | 17032251 | CUBN | 106 |
| 11 | 122931931 | 122933964 | HSPA8 | 106 |
| 2 | 58395180 | 58395836 | FANCL | 106 |
| 12 | 31881858 | 31882580 | AMN1 | 106 |
| 8 | 142218545 | 142219489 | SLC45A4 | 106 |
| 11 | 17099677 | 17099977 | RPS13 | 106 |
| 18 | 74332799 | 74333017 | ZNF516 | 106 |
| 12 | 34175045 | 34175897 | ALG10 | 106 |
| 13 | 86540563 | 86541444 | SLITRK6 | 106 |
| 13 | 26760257 | 26761482 | RNF6 | 106 |
| 6 | 42895168 | 42896258 | CNPY3 | 106 |
| 20 | 61370323 | 61372470 | NTSR1 | 106 |
| 5 | 138655370 | 138656061 | MATR3 | 106 |
| 17 | 16913547 | 16915483 | MPRIP | 106 |
| 1 | 34058065 | 34058628 | CSMD2 | 106 |
| 18 | 46359188 | 46359912 | CTIF | 106 |
| 4 | 15854238 | 15854958 | CD38 | 106 |
| 1 | 27128022 | 27128682 | ZDHHC18 | 105 |
| 1 | 150374891 | 150375504 | RPRD2 | 105 |
| 7 | 156477888 | 156478499 | LMBR1 | 105 |
| 17 | 30615424 | 30616195 | RHBDL3 | 105 |
| 8 | 142219499 | 142219645 | SLC45A4 | 105 |
| 13 | 114828914 | 114828996 | RASA3 | 105 |
| 6 | 105272465 | 105273018 | HACE1 | 105 |
| 3 | 157837522 | 157838134 | RSRC1 | 104 |
| 15 | 102160839 | 102161505 | TM2D3 | 104 |
| 8 | 100862691 | 100863265 | VPS13B | 104 |
|  |  |  |  |  |
