# Supplementary material for: The DEK oncoprotein binds to highly and ubiquitously expressed genes with a dual role in their transcriptional regulation
Source: Mol Cancer. 2014 Sep 12;13:215. doi: 10.1186/1476-4598-13-215 (PMC4175287; doi:10.1186/1476-4598-13-215)
Supplement: Supplementary file 2 — Additional file 2: Table S2: Correlation between the binding pattern of DEK and those of other factors. Complete list of all 2642 ChIP-seq experiments in the Encode database, ordered from that with the most to that with the least similar binding pattern to that of DEK. (DOCX 152 KB) [file 12943_2014_1416_MOESM2_ESM.docx]

**Additional file 2: Table S2.** **Correlation between the binding pattern of DEK and those of other factors.** Complete list of all 2642 ChIP-seq experiments in the Encode database, ordered from that with the most to that with the least similar binding pattern to that of DEK.

| TRACK | SIMILARITY SCORE |
| --- | --- |
|  |  |
|  |  |
| EncodeHaibTfbsHl60Pol24h8V0422111PkRep2 | 0.089874 |
| EncodeHaibTfbsGm12878Runx3sc101553V0422111PkRep2 | 0.088592 |
| EncodeOpenChromChipGm12878Pol2Pk | 0.080866 |
| EncodeHaibTfbsGm12878Mta3sc81325V0422111PkRep2 | 0.077809 |
| EncodeOpenChromChipMcf7Pol2SerumstvdPkRep1 | 0.076889 |
| EncodeHaibTfbsGm12878Runx3sc101553V0422111PkRep1 | 0.074765 |
| EncodeHaibTfbsGm12878Pmlsc71910V0422111PkRep2 | 0.074663 |
| EncodeHaibTfbsGm12878Elf1sc631V0416101PkRep2 | 0.074556 |
| EncodeOpenChromChipHelas3Pol2Pk | 0.073332 |
| EncodeOpenChromChipMcf7Pol2SerumstimPkRep1 | 0.073268 |
| EncodeOpenChromChipMcf7Pol2PkRep1 | 0.073212 |
| EncodeHaibTfbsHl60Pol24h8V0422111PkRep1 | 0.071680 |
| EncodeHaibTfbsGm12878Pol2Pcr2xPkRep1 | 0.070023 |
| EncodeHaibTfbsGm12878Foxm1sc502V0422111PkRep2 | 0.068460 |
| EncodeHaibTfbsGm12892Pol2V0416102PkRep1 | 0.067508 |
| EncodeOpenChromDnaseNaivebcellPk | 0.065978 |
| EncodeHaibTfbsGm12878Pol2Pcr2xPkRep2 | 0.065608 |
| EncodeHaibTfbsGm12878Bclaf101388V0416101PkRep1 | 0.065088 |
| EncodeHaibTfbsHelas3Pol2Pcr1xPkRep2 | 0.064778 |
| EncodeHaibTfbsGm12878Pol24h8Pcr1xPkRep2 | 0.064508 |
| EncodeOpenChromChipA549Pol2PkRep1 | 0.064493 |
| EncodeHaibTfbsK562MaxV0416102PkRep2 | 0.064209 |
| EncodeOpenChromChipProgfibPol2PkRep1 | 0.063698 |
| EncodeHaibTfbsGm12878Stat5asc74442V0422111PkRep2 | 0.063695 |
| EncodeOpenChromChipH1hescPol2Pk | 0.063588 |
| EncodeBroadHistoneMonocd14ro1746H3k09acPk | 0.063303 |
| EncodeBroadHistoneMonocd14ro1746H3k04me2Pk | 0.063018 |
| EncodeHaibTfbsGm12891Pou2f2Pcr1xPkRep1 | 0.062555 |
| EncodeOpenChromChipK562Pol2Pk | 0.062146 |
| EncodeOpenChromChipGlioblaPol2PkRep1 | 0.061357 |
| EncodeHaibTfbsGm12878Foxm1sc502V0422111PkRep1 | 0.060881 |
| EncodeHaibTfbsSknshPol24h8V0416101PkRep2 | 0.060824 |
| EncodeHaibTfbsGm12878Yy1sc281Pcr1xPkRep1 | 0.060568 |
| EncodeHaibTfbsGm12892Pol24h8V0416102PkRep2 | 0.060028 |
| EncodeHaibTfbsGm12891Pol2Pcr1xPkRep2 | 0.060024 |
| EncodeOpenChromDnaseGm10248Pk | 0.059794 |
| EncodeOpenChromDnaseGm13976Pk | 0.059498 |
| EncodeOpenChromDnaseGcbcellPk | 0.059475 |
| EncodeBroadHistoneCd20H3k04me2Pk | 0.059048 |
| EncodeHaibTfbsGm12892Pol24h8V0416102PkRep1 | 0.058817 |
| EncodeHaibTfbsSknshSin3ak20V0416101PkRep2 | 0.058557 |
| EncodeHaibTfbsGm12892Pol2V0416102PkRep2 | 0.058398 |
| EncodeHaibTfbsSknmcPol24h8V0416101PkRep1 | 0.057885 |
| EncodeHaibTfbsHelas3Pol2Pcr1xPkRep1 | 0.057792 |
| EncodeHaibTfbsGm12891Pol24h8Pcr1xPkRep1 | 0.057712 |
| EncodeHaibTfbsGm12891Pol2Pcr1xPkRep1 | 0.057506 |
| EncodeHaibTfbsH1hescTaf1V0416102PkRep1 | 0.057496 |
| EncodeOpenChromDnaseGm18507Pk | 0.057303 |
| EncodeHaibTfbsK562Hey1Pcr1xPkRep1 | 0.056659 |
| EncodeHaibTfbsK562Pol2V0416101PkRep2 | 0.056443 |
| EncodeHaibTfbsGm12878Pol24h8Pcr1xPkRep1 | 0.056183 |
| EncodeOpenChromDnaseCllPk | 0.056137 |
| EncodeOpenChromChipHuvecPol2Pk | 0.056108 |
| EncodeHaibTfbsGm12891Pol24h8Pcr1xPkRep2 | 0.056051 |
| EncodeOpenChromDnaseGm19238Pk | 0.055856 |
| EncodeSydhHistonePbmcH3k4me1UcdPk | 0.055735 |
| EncodeHaibTfbsH1hescPol2V0416102PkRep2 | 0.055192 |
| EncodeHaibTfbsGm12878Ebfsc137065Pcr1xPkRep2 | 0.055113 |
| EncodeOpenChromDnaseGm12878Pk | 0.055037 |
| EncodeHaibTfbsHct116Pol24h8V0416101PkRep2 | 0.054272 |
| EncodeHaibTfbsH1hescPol24h8V0416102PkRep1 | 0.054259 |
| EncodeHaibTfbsHepg2Mybl2sc81192V0422111PkRep1 | 0.054246 |
| EncodeHaibTfbsGm12878Tcf12Pcr1xPkRep1 | 0.054197 |
| EncodeHaibTfbsGm12878Atf2sc81188V0422111PkRep1 | 0.054184 |
| EncodeHaibTfbsHl60Pu1V0422111PkRep1 | 0.054145 |
| EncodeHaibTfbsA549MaxV0422111PkRep1 | 0.054028 |
| EncodeHaibTfbsGm12878Taf1Pcr1xPkRep2 | 0.053908 |
| EncodeHaibTfbsA549Pol2Pcr2xDex100nmPkRep1 | 0.053882 |
| EncodeOpenChromDnaseHsmmembPk | 0.053774 |
| EncodeOpenChromSynthGm12878Pk | 0.053535 |
| EncodeOpenChromFaireGm12891Pk | 0.053336 |
| EncodeHaibTfbsGm12878Elf1sc631V0416101PkRep1 | 0.053294 |
| EncodeHaibTfbsPanc1Pol24h8V0416101PkRep2 | 0.053215 |
| EncodeHaibTfbsA549JundV0416102Etoh02PkRep2 | 0.053090 |
| EncodeHaibTfbsK562Pol2V0416101PkRep1 | 0.053004 |
| EncodeHaibTfbsGm12878Pou2f2Pcr1xPkRep2 | 0.052938 |
| EncodeOpenChromFaireGm18507Pk | 0.052821 |
| EncodeHaibTfbsU87Pol24h8V0416101PkRep1V2 | 0.052682 |
| EncodeOpenChromDnaseUrotsaPk | 0.052554 |
| EncodeHaibTfbsK562Pol24h8V0416101PkRep2 | 0.052455 |
| EncodeBroadHistoneMonocd14ro1746H3k27acPk | 0.052405 |
| EncodeOpenChromDnaseStellatePk | 0.052390 |
| EncodeOpenChromDnaseGm12892Pk | 0.052196 |
| EncodeOpenChromSynthGm18507Pk | 0.052184 |
| EncodeHaibTfbsH1hescTaf1V0416102PkRep2 | 0.052063 |
| EncodeHaibTfbsHct116Pol24h8V0416101PkRep1 | 0.052047 |
| EncodeOpenChromDnaseMonocd14Pk | 0.051861 |
| EncodeHaibTfbsK562Cbx3sc101004V0422111PkRep1 | 0.051743 |
| EncodeBroadHistoneGm12878H3k9acStdPk | 0.051080 |
| EncodeBroadHistoneCd20H2azPk | 0.050945 |
| EncodeHaibTfbsGm12878Nficsc81335V0422111PkRep1 | 0.050844 |
| EncodeOpenChromDnaseGm19240Pk | 0.050809 |
| EncodeHaibTfbsK562Hey1Pcr1xPkRep2 | 0.050767 |
| EncodeBroadHistoneMonocd14ro1746H3k04me3Pk | 0.050598 |
| EncodeHaibTfbsA549MaxV0422111PkRep2 | 0.050527 |
| EncodeHaibTfbsSknshPol24h8V0416101PkRep1 | 0.050399 |
| EncodeHaibTfbsHuvecPol24h8V0416101PkRep2 | 0.050384 |
| EncodeOpenChromDnaseAdultcd4th1Pk | 0.050304 |
| EncodeOpenChromSynthGm12891Pk | 0.050172 |
| EncodeOpenChromSynthGm12892Pk | 0.050022 |
| EncodeBroadHistoneGm12878H2azStdPk | 0.049986 |
| EncodeOpenChromChipHepg2Pol2Pk | 0.049967 |
| EncodeHaibTfbsK562Elf1sc631V0416102PkRep2 | 0.049904 |
| EncodeHaibTfbsGm12878Pmlsc71910V0422111PkRep1 | 0.049800 |
| EncodeBroadHistoneK562Phf8a301772aStdPk | 0.049785 |
| EncodeHaibTfbsSknshTaf1V0416101PkRep2 | 0.049749 |
| EncodeOpenChromDnaseAdultcd4th0Pk | 0.049637 |
| EncodeHaibTfbsU87Pol24h8V0416101PkRep2V2 | 0.049611 |
| EncodeHaibTfbsHepg2Hey1V0416101PkRep1 | 0.049610 |
| EncodeHaibTfbsEcc1Pol2V0416102Dm002p1hPkRep2 | 0.049540 |
| EncodeHaibTfbsSknshMaxV0422111PkRep1 | 0.049535 |
| EncodeBroadHistoneMonocd14ro1746H3k04me1Pk | 0.049384 |
| EncodeHaibTfbsGm12892Yy1V0416101PkRep1 | 0.049380 |
| EncodeHaibTfbsGm12878Tcf3Pcr1xPkRep1 | 0.049242 |
| EncodeOpenChromDnaseFibropag20443Pk | 0.049075 |
| EncodeOpenChromDnaseMcf7RandshrnaPk | 0.049020 |
| EncodeOpenChromDnaseHelas3Pk | 0.049006 |
| EncodeOpenChromDnaseFibroblgm03348LentimyodPk | 0.048988 |
| EncodeOpenChromDnaseFibroblgm03348LenticonPk | 0.048951 |
| EncodeHaibTfbsHuvecPol2Pcr1xPkRep2 | 0.048944 |
| EncodeHaibTfbsHepg2Pol2Pcr2xPkRep2 | 0.048797 |
| EncodeHaibTfbsK562Pmlsc71910V0422111PkRep2 | 0.048707 |
| EncodeOpenChromDnaseMcf7CtcfshrnaPk | 0.048676 |
| EncodeBroadHistoneGm12878H3k4me2StdPk | 0.048513 |
| EncodeSydhHistonePbmcH3k04me3bUcdPk | 0.048330 |
| EncodeHaibTfbsA549Pol2Pcr2xDex100nmPkRep2 | 0.048287 |
| EncodeOpenChromDnaseGlioblaPk | 0.048255 |
| EncodeSydhTfbsGm12891Pol2IggmusPk | 0.048130 |
| EncodeSydhTfbsRajiPol2UcdPk | 0.047891 |
| EncodeOpenChromDnaseGm20000Pk | 0.047781 |
| EncodeOpenChromDnaseFibroblgm03348Pk | 0.047743 |
| EncodeHaibTfbsGm12878Yy1sc281Pcr1xPkRep2 | 0.047735 |
| EncodeOpenChromDnaseK562PkV2 | 0.047642 |
| EncodeOpenChromDnaseMcf7HypoxlacconPk | 0.047573 |
| EncodeHaibTfbsHuvecPol24h8V0416101PkRep1 | 0.047399 |
| EncodeOpenChromDnaseGm12891Pk | 0.047366 |
| EncodeHaibTfbsGm12878Pax5c20Pcr1xPkRep1 | 0.047316 |
| EncodeOpenChromDnaseGm19239Pk | 0.047206 |
| EncodeOpenChromDnaseGm13977Pk | 0.047114 |
| EncodeSydhTfbsGm19193Pol2IggmusPk | 0.047106 |
| EncodeHaibTfbsA549Sp1V0422111Etoh02PkRep1 | 0.047076 |
| EncodeHaibTfbsA549Sin3ak20V0422111Etoh02PkRep1 | 0.047030 |
| EncodeOpenChromDnaseFibropag08395Pk | 0.046950 |
| EncodeOpenChromFaireGm12892Pk | 0.046916 |
| EncodeOpenChromSynthGm19239Pk | 0.046898 |
| EncodeHaibTfbsGm12878Cebpbsc150V0422111PkRep1 | 0.046866 |
| EncodeHaibTfbsK562Taf1V0416101PkRep1 | 0.046753 |
| EncodeHaibTfbsGm12878Mta3sc81325V0422111PkRep1 | 0.046736 |
| EncodeHaibTfbsA549Pol2Pcr2xEtoh02PkRep1 | 0.046646 |
| EncodeOpenChromDnaseUrothelPkV2 | 0.046552 |
| EncodeOpenChromDnaseCd20ro01794Pk | 0.046516 |
| EncodeHaibTfbsU87Pol24h8V0416101PkRep2 | 0.046427 |
| EncodeBroadHistoneCd20ro01794H3k27acPk | 0.046401 |
| EncodeHaibTfbsGm12891Pou2f2Pcr1xPkRep2 | 0.046381 |
| EncodeHaibTfbsSknmcPol24h8V0416101PkRep2 | 0.046366 |
| EncodeHaibTfbsSknshMaxV0422111PkRep2 | 0.046354 |
| EncodeOpenChromDnaseEcc1Est10nm30mPk | 0.046172 |
| EncodeHaibTfbsHepg2Hey1V0416101PkRep2 | 0.045923 |
| EncodeHaibTfbsMcf7MaxV0422111PkRep1 | 0.045905 |
| EncodeOpenChromDnaseHsmmfshdPk | 0.045788 |
| EncodeHaibTfbsK562Yy1V0416102PkRep2 | 0.045770 |
| EncodeHaibTfbsPfsk1Pol24h8V0416101PkRep1 | 0.045746 |
| EncodeOpenChromDnaseK562SahactrlPk | 0.045737 |
| EncodeHaibTfbsK562MaxV0416102PkRep1 | 0.045685 |
| EncodeOpenChromDnaseA549Pk | 0.045675 |
| EncodeHaibTfbsHelas3Taf1Pcr1xPkRep2 | 0.045663 |
| EncodeOpenChromDnaseHtr8Pk | 0.045587 |
| EncodeHaibTfbsGm12878Pax5n19Pcr1xPkRep2 | 0.045558 |
| EncodeOpenChromDnaseHpde6e6e7Pk | 0.045516 |
| EncodeOpenChromDnaseImr90Pk | 0.045430 |
| EncodeOpenChromDnaseRwpe1Pk | 0.045302 |
| EncodeHaibTfbsH1hescPol2V0416102PkRep1 | 0.045279 |
| EncodeHaibTfbsA549Yy1cV0422111Etoh02PkRep1 | 0.045249 |
| EncodeHaibTfbsGm12878Bclaf101388V0416101PkRep2 | 0.045192 |
| EncodeOpenChromDnaseT47dEst10nm30mPk | 0.045109 |
| EncodeOpenChromDnaseHepg2Pk | 0.045001 |
| EncodeOpenChromDnaseOlfneurospherePk | 0.044969 |
| EncodeSydhTfbsMcf10aesCmycEtoh01HvdPk | 0.044869 |
| EncodeHaibTfbsPanc1Pol24h8V0416101PkRep1 | 0.044865 |
| EncodeOpenChromDnaseFibroblPk | 0.044853 |
| EncodeOpenChromSynthHuvecPk | 0.044839 |
| EncodeOpenChromDnaseGm10266Pk | 0.044748 |
| EncodeOpenChromDnaseUrotsaUt189Pk | 0.044720 |
| EncodeOpenChromDnaseFibropag08396Pk | 0.044701 |
| EncodeBroadHistoneHuvecH3k9acStdPk | 0.044677 |
| EncodeBroadHistoneNhekH3k9acStdPk | 0.044620 |
| EncodeBroadHistoneHelas3H3k27acStdPk | 0.044560 |
| EncodeOpenChromDnaseCerebellumocPk | 0.044518 |
| EncodeSydhTfbsGm12878Pol2IggmusPk | 0.044432 |
| EncodeOpenChromDnaseUrothelUt189PkV2 | 0.044429 |
| EncodeOpenChromDnaseIshikawaTam10030Pk | 0.044407 |
| EncodeHaibTfbsEcc1MaxV0422111PkRep2 | 0.044373 |
| EncodeSydhTfbsGm19099Pol2IggmusPk | 0.044331 |
| EncodeBroadHistoneK562Plu1StdPk | 0.044303 |
| EncodeOpenChromDnasePanisdPk | 0.044206 |
| EncodeHaibTfbsHuvecPol2Pcr1xPkRep1 | 0.044043 |
| EncodeBroadHistoneK562H3k4me2StdPk | 0.043988 |
| EncodeHaibTfbsGm12878Sp1Pcr1xPkRep2 | 0.043982 |
| EncodeSydhTfbsH1hescTbpIggrabPk | 0.043918 |
| EncodeHaibTfbsEcc1MaxV0422111PkRep1 | 0.043838 |
| EncodeHaibTfbsPfsk1Taf1V0416101PkRep2 | 0.043785 |
| EncodeHaibTfbsA549Creb1sc240V0416102Dex100nmPkRep1 | 0.043656 |
| EncodeHaibTfbsK562Tead4sc101184V0422111PkRep2 | 0.043648 |
| EncodeOpenChromDnaseEcc1Dm002p1hPk | 0.043596 |
| EncodeHaibTfbsGm12878Nfatc1sc17834V0422111PkRep2 | 0.043547 |
| EncodeHaibTfbsEcc1Pol2V0416102Dm002p1hPkRep1 | 0.043544 |
| EncodeOpenChromDnaseHuvecPk | 0.043529 |
| EncodeSydhTfbsHepg2TbpIggrabPk | 0.043501 |
| EncodeSydhTfbsGm19099NfkbTnfaIggrabPk | 0.043366 |
| EncodeOpenChromFaireHelas3Ifng4hPk | 0.043315 |
| EncodeOpenChromFaireGm19239Pk | 0.043273 |
| EncodeHaibTfbsGm12878Stat5asc74442V0422111PkRep1 | 0.043254 |
| EncodeOpenChromDnaseProgfibPk | 0.043186 |
| EncodeHaibTfbsH1hescPol24h8V0416102PkRep2 | 0.043166 |
| EncodeOpenChromSynthHelas3Pk | 0.043107 |
| EncodeOpenChromDnaseNhekPk | 0.043086 |
| EncodeHaibTfbsHepg2Mybl2sc81192V0422111PkRep2 | 0.043033 |
| EncodeHaibTfbsA549Tcf12V0422111Etoh02PkRep2 | 0.042911 |
| EncodeBroadHistoneHelas3H3k9acStdPk | 0.042876 |
| EncodeOpenChromDnaseCerebrumfrontalocPk | 0.042837 |
| EncodeOpenChromSynthHepg2Pk | 0.042792 |
| EncodeOpenChromDnaseIshikawaEst10nm30mPk | 0.042733 |
| EncodeOpenChromDnaseHek293tPk | 0.042721 |
| EncodeBroadHistoneNhdfadH3k4me2StdPk | 0.042699 |
| EncodeBroadHistoneA549H3k09acEtoh02Pk | 0.042661 |
| EncodeOpenChromDnaseK562Saha1u72hrPk | 0.042659 |
| EncodeOpenChromDnaseK562G2mphasePk | 0.042592 |
| EncodeOpenChromDnaseMcf7HypoxlacPk | 0.042518 |
| EncodeHaibTfbsK562Yy1sc281V0416101PkRep1 | 0.042456 |
| EncodeHaibTfbsK562Yy1V0416101PkRep1 | 0.042456 |
| EncodeBroadHistoneDnd41H3k09acPk | 0.042388 |
| EncodeHaibTfbsU87Pol24h8V0416101PkRep1 | 0.042358 |
| EncodeHaibTfbsK562Nr2f2sc271940V0422111PkRep2 | 0.042307 |
| EncodeHaibTfbsA549Taf1V0422111Etoh02PkRep1 | 0.042217 |
| EncodeBroadHistoneHuvecH3k4me2StdPk | 0.042080 |
| EncodeOpenChromDnaseMel2183Pk | 0.042017 |
| EncodeSydhTfbsK562Ccnt2StdPk | 0.041932 |
| EncodeHaibTfbsHepg2Pol2Pcr2xPkRep1 | 0.041927 |
| EncodeBroadHistoneNhekH3k4me3StdPk | 0.041896 |
| EncodeOpenChromDnaseFibropPk | 0.041811 |
| EncodeHaibTfbsH1neuronsPol24h8V0422111PkRep1 | 0.041735 |
| EncodeHaibTfbsGm12878Tcf3Pcr1xPkRep2 | 0.041719 |
| EncodeOpenChromDnaseMelanoPk | 0.041708 |
| EncodeBroadHistoneNhdfadH3k9acStdPk | 0.041667 |
| EncodeBroadHistoneNhaH3k04me2Pk | 0.041657 |
| EncodeSydhTfbsGm18505Pol2IggmusPk | 0.041650 |
| EncodeBroadHistoneH1hescPhf8a301772aPk | 0.041582 |
| EncodeBroadHistoneGm12878H3k04me3StdPkV2 | 0.041572 |
| EncodeBroadHistoneK562H3k27acStdPk | 0.041565 |
| EncodeSydhTfbsHepg2Pol2IggrabPk | 0.041564 |
| EncodeSydhTfbsNb4Pol2StdPk | 0.041524 |
| EncodeHaibTfbsGm12891Taf1Pcr1xPkRep1 | 0.041521 |
| EncodeOpenChromDnaseK562NabutPk | 0.041514 |
| EncodeBroadHistoneHuvecH3k4me3StdPk | 0.041489 |
| EncodeOpenChromDnaseHeartocPk | 0.041444 |
| EncodeSydhTfbsHepg2Mxi1StdPk | 0.041417 |
| EncodeHaibTfbsK562Pol24h8V0416101PkRep1 | 0.041329 |
| EncodeHaibTfbsGm12891Yy1sc281V0416101PkRep2 | 0.041309 |
| EncodeBroadHistoneMonocd14ro1746H2azPk | 0.041199 |
| EncodeBroadHistoneK562H3k4me3StdPk | 0.041178 |
| EncodeBroadHistoneNhlfH3k27acStdPk | 0.041143 |
| EncodeBroadHistoneHepg2H3k4me3StdPk | 0.041116 |
| EncodeBroadHistoneDnd41H3k04me2Pk | 0.041092 |
| EncodeSydhTfbsGm12892Pol2IggmusPk | 0.041075 |
| EncodeSydhTfbsH1hescSin3anb6001263IggrabPk | 0.041062 |
| EncodeBroadHistoneHuvecH3k27acStdPk | 0.040976 |
| EncodeSydhTfbsK562Hmgn3StdPk | 0.040950 |
| EncodeHaibTfbsH1neuronsPol24h8V0422111PkRep2 | 0.040795 |
| EncodeBroadHistoneOsteoH3k04me3Pk | 0.040741 |
| EncodeBroadHistoneHsmmtH3k4me2StdPk | 0.040736 |
| EncodeBroadHistoneA549H3k04me3Dex100nmPk | 0.040697 |
| EncodeHaibTfbsSknshraYy1sc281V0416102PkRep2 | 0.040579 |
| EncodeBroadHistoneHsmmtH3k4me3StdPk | 0.040534 |
| EncodeOpenChromDnaseK562G1phasePk | 0.040498 |
| EncodeBroadHistoneNhekH3k4me2StdPk | 0.040491 |
| EncodeHaibTfbsMcf7Sin3ak20V0422111PkRep1 | 0.040407 |
| EncodeOpenChromFaireHuvecPk | 0.040366 |
| EncodeOpenChromDnasePhtePk | 0.040313 |
| EncodeHaibTfbsHepg2Yy1sc281V0416101PkRep1 | 0.040307 |
| EncodeBroadHistoneA549H3k27acDex100nmPk | 0.040287 |
| EncodeBroadHistoneNhaH3k4me3StdPk | 0.040287 |
| EncodeBroadHistoneHepg2H3k4me2StdPk | 0.040071 |
| EncodeBroadHistoneHsmmH3k4me3StdPk | 0.040053 |
| EncodeOpenChromDnaseAosmcSerumfreePk | 0.039964 |
| EncodeHaibTfbsK562GabpV0416101PkRep2 | 0.039954 |
| EncodeBroadHistoneHelas3H3k4me3StdPk | 0.039779 |
| EncodeBroadHistoneNhlfH3k9acStdPk | 0.039744 |
| EncodeOpenChromDnaseSknshPk | 0.039729 |
| EncodeBroadHistoneNhaH3k09acPk | 0.039661 |
| EncodeSydhHistonePanc1H3k04me3bUcdPk | 0.039589 |
| EncodeHaibTfbsA549Pol2Pcr2xEtoh02PkRep2 | 0.039574 |
| EncodeHaibTfbsGm12878Pou2f2Pcr1xPkRep1 | 0.039526 |
| EncodeBroadHistoneNhdfadH3k4me3StdPk | 0.039519 |
| EncodeBroadHistoneGm12878H3k4me3StdPk | 0.039412 |
| EncodeOpenChromDnaseMcf7Pk | 0.039333 |
| EncodeSydhHistonePanc1H3k27acUcdPk | 0.039211 |
| EncodeHaibTfbsHepg2Pol24h8V0416102PkRep1 | 0.039166 |
| EncodeBroadHistoneA549H3k04me2Etoh02Pk | 0.039122 |
| EncodeSydhTfbsPbdePol2UcdPk | 0.039040 |
| EncodeBroadHistoneHsmmH3k4me2StdPk | 0.039033 |
| EncodeHaibTfbsA549GabpV0422111Etoh02PkRep1 | 0.039020 |
| EncodeOpenChromSynthK562Pk | 0.038959 |
| EncodeSydhTfbsGm18951Pol2IggmusPk | 0.038948 |
| EncodeOpenChromFaireHelas3Ifna4hPk | 0.038935 |
| EncodeBroadHistoneK562H3k9acStdPk | 0.038821 |
| EncodeBroadHistoneHsmmtH3k9acStdPk | 0.038778 |
| EncodeHaibTfbsK562Taf1V0416101PkRep2 | 0.038745 |
| EncodeOpenChromDnaseIpsnihi11Pk | 0.038501 |
| EncodeBroadHistoneA549H3k04me3Etoh02Pk | 0.038480 |
| EncodeSydhTfbsMcf10aesE2f4TamHvdPk | 0.038343 |
| EncodeHaibTfbsHct116Yy1sc281V0416101PkRep2 | 0.038295 |
| EncodeHaibTfbsA549Sp1V0422111Etoh02PkRep2 | 0.038241 |
| EncodeSydhTfbsHelas3Pol2StdPk | 0.038226 |
| EncodeOpenChromDnaseMedullod341Pk | 0.038217 |
| EncodeSydhTfbsGm18526Pol2IggmusPk | 0.038108 |
| EncodeSydhTfbsK562CmycIfng30StdPk | 0.038055 |
| EncodeBroadHistoneDnd41H3k04me3Pk | 0.038022 |
| EncodeOpenChromDnaseIpsnihi7Pk | 0.038002 |
| EncodeBroadHistoneHsmmH3k9acStdPk | 0.037998 |
| EncodeSydhTfbsGm15510NfkbTnfaIggrabPk | 0.037978 |
| EncodeOpenChromSynthH1hescPk | 0.037820 |
| EncodeOpenChromDnaseColo829Pk | 0.037603 |
| EncodeSydhTfbsK562Pol2StdPk | 0.037595 |
| EncodeHaibTfbsPfsk1Foxp2Pcr2xPkRep1 | 0.037594 |
| EncodeSydhTfbsGm18951NfkbTnfaIggrabPk | 0.037516 |
| EncodeOpenChromDnaseLncapAndroPk | 0.037432 |
| EncodeBroadHistoneHepg2H3k9acStdPk | 0.037391 |
| EncodeOpenChromDnaseMyometrPk | 0.037295 |
| EncodeBroadHistoneHsmmtH2azStdPk | 0.037279 |
| EncodeHaibTfbsPanc1Sin3ak20V0416101PkRep1 | 0.037185 |
| EncodeOpenChromDnaseHelas3Ifna4hPk | 0.037152 |
| EncodeSydhHistoneNt2d1H3k9acbUcdPk | 0.037133 |
| EncodeBroadHistoneNhdfadH3k27acStdPk | 0.037065 |
| EncodeSydhTfbsGm15510Pol2IggmusPk | 0.036991 |
| EncodeOpenChromChipMcf7CmycVehPkRep1 | 0.036953 |
| EncodeOpenChromSynthGlioblaPk | 0.036853 |
| EncodeSydhHistoneNt2d1H3k4me3bUcdPk | 0.036845 |
| EncodeBroadHistoneGm12878H3k04me1StdPkV2 | 0.036836 |
| EncodeUchicagoTfbsK562EjundControlPk | 0.036757 |
| EncodeBroadHistoneNhlfH3k4me2StdPk | 0.036731 |
| EncodeBroadHistoneNhekH3k27acStdPk | 0.036595 |
| EncodeBroadHistoneA549H3k04me2Dex100nmPk | 0.036586 |
| EncodeOpenChromDnaseIpscwru1Pk | 0.036513 |
| EncodeHaibTfbsA549Elf1V0422111Etoh02PkRep2 | 0.036326 |
| EncodeOpenChromDnaseHuh7Pk | 0.036305 |
| EncodeHaibTfbsHepg2Pol24h8V0416102PkRep2 | 0.036298 |
| EncodeBroadHistoneK562H2azStdPk | 0.036229 |
| EncodeSydhTfbsMcf10aesCmycTam14hHvdPk | 0.036172 |
| EncodeBroadHistoneHuvecH2azPk | 0.036168 |
| EncodeHaibTfbsGm12878Nficsc81335V0422111PkRep2 | 0.035935 |
| EncodeSydhHistoneK562bH3k9acbUcdPk | 0.035921 |
| EncodeSydhHistoneK562H3k9acbUcdPk | 0.035921 |
| EncodeSydhTfbsGm12878Mazab85725IggmusPk | 0.035830 |
| EncodeBroadHistoneNhlfH3k4me3StdPk | 0.035811 |
| EncodeBroadHistoneHsmmH2azStdPk | 0.035735 |
| EncodeBroadHistoneHmecH3k4me3StdPk | 0.035650 |
| EncodeBroadHistoneH1hescHdac2a300705aPk | 0.035569 |
| EncodeOpenChromSynthHelas3Ifna4hPk | 0.035382 |
| EncodeHaibTfbsK562Elf1sc631V0416102PkRep1 | 0.035325 |
| EncodeOpenChromDnaseHsmmPk | 0.035318 |
| EncodeOpenChromDnaseK562Pk | 0.035229 |
| EncodeBroadHistoneH1hescH3k4me3StdPk | 0.035218 |
| EncodeOpenChromDnasePsoasmuscleocPk | 0.035112 |
| EncodeOpenChromChipMcf7CmycSerumstimPkRep1 | 0.035048 |
| EncodeBroadHistoneGm12878H3k4me1StdPk | 0.035032 |
| EncodeHaibTfbsA549Sin3ak20V0422111Etoh02PkRep2 | 0.035010 |
| EncodeHaibTfbsH1hescYy1sc281V0416102PkRep2 | 0.034948 |
| EncodeBroadHistoneK562Rbbp5a300109aStdPk | 0.034921 |
| EncodeSydhTfbsNb4MaxStdPk | 0.034893 |
| EncodeHaibTfbsMcf7Elf1V0422111PkRep1 | 0.034862 |
| EncodeBroadHistoneH1hescH3k4me2StdPk | 0.034855 |
| EncodeSydhTfbsGm12878Znf143166181apStdPk | 0.034791 |
| EncodeHaibTfbsGm12878Atf2sc81188V0422111PkRep2 | 0.034748 |
| EncodeOpenChromDnaseHuh75Pk | 0.034694 |
| EncodeSydhTfbsNb4CmycStdPk | 0.034668 |
| EncodeBroadHistoneH1hescSap3039731Pk | 0.034628 |
| EncodeOpenChromDnaseH7esPk | 0.034616 |
| EncodeBroadHistoneNhaH3k27acStdPk | 0.034604 |
| EncodeSydhTfbsGm12878Ebf1sc137065StdPk | 0.034591 |
| EncodeOpenChromSynthHtr8Pk | 0.034543 |
| EncodeHaibTfbsSknshraYy1sc281V0416102PkRep1 | 0.034513 |
| EncodeBroadHistoneHsmmtH3k27acStdPk | 0.034465 |
| EncodeBroadHistoneDnd41H3k04me1Pk | 0.034463 |
| EncodeBroadHistoneHelas3H3k4me2StdPk | 0.034419 |
| EncodeBroadHistoneA549H3k27acEtoh02Pk | 0.034350 |
| EncodeOpenChromSynthNhekPk | 0.034166 |
| EncodeBroadHistoneDnd41H3k27acPk | 0.033970 |
| EncodeSydhTfbsImr90Pol2IggrabPk | 0.033930 |
| EncodeBroadHistoneA549H2azDex100nmPk | 0.033773 |
| EncodeHaibTfbsK562Trim28sc81411V0422111PkRep1 | 0.033748 |
| EncodeBroadHistoneNhekH2azPk | 0.033687 |
| EncodeSydhTfbsImr90Mazab85725IggrabPk | 0.033631 |
| EncodeSydhTfbsMcf7Hae2f1UcdPk | 0.033600 |
| EncodeHaibTfbsA549Ets1V0422111Etoh02PkRep1 | 0.033591 |
| EncodeBroadHistoneHsmmH3k27acStdPk | 0.033525 |
| EncodeSydhTfbsGm12878Chd2ab68301IggmusPk | 0.033511 |
| EncodeUchicagoTfbsK562EjunbControlPk | 0.033432 |
| EncodeOpenChromFaireK562Pk | 0.033380 |
| EncodeHaibTfbsHepg2Elf1sc631V0416101PkRep2 | 0.033318 |
| EncodeHaibTfbsPfsk1Pol24h8V0416101PkRep2 | 0.033274 |
| EncodeHaibTfbsH1hescYy1sc281V0416102PkRep1 | 0.033119 |
| EncodeHaibTfbsGm12878Creb1sc240V0422111PkRep1 | 0.033114 |
| EncodeSydhTfbsGm12878Bhlhe40cIggmusPk | 0.033111 |
| EncodeBroadHistoneHuvecH3k4me1StdPk | 0.033050 |
| EncodeOpenChromDnasePanisletsPk | 0.033025 |
| EncodeHaibTfbsHepg2Nficsc81335V0422111PkRep1 | 0.032842 |
| EncodeSydhTfbsK562Mazab85725IggrabPk | 0.032525 |
| EncodeOpenChromDnaseFrontalcortexocPk | 0.032474 |
| EncodeSydhTfbsGm12878Stat1StdPk | 0.032453 |
| EncodeSydhHistoneHct116H3k27acUcdPk | 0.032407 |
| EncodeHaibTfbsEcc1Taf1V0422111PkRep1 | 0.032350 |
| EncodeSydhTfbsK562CmycIggrabPk | 0.032339 |
| EncodeOpenChromDnaseT47dPk | 0.032327 |
| EncodeSydhTfbsGm12878Mxi1IggmusPk | 0.032326 |
| EncodeOpenChromDnaseHmecPk | 0.032047 |
| EncodeOpenChromDnaseOsteoblPk | 0.032004 |
| EncodeHaibTfbsA549Bcl3V0422111Etoh02PkRep2 | 0.031896 |
| EncodeUwDnaseNb4PkRep1 | 0.031839 |
| EncodeHaibTfbsGm12878Pax5c20Pcr1xPkRep2 | 0.031809 |
| EncodeBroadHistoneH1hescJmjd2aa300861a1Pk | 0.031792 |
| EncodeADnaseUwNb4UniPk | 0.031787 |
| EncodeBroadHistoneK562Hdac1sc6298StdPk | 0.031732 |
| EncodeHaibTfbsH1hescTaf7sc101167V0416102PkRep2 | 0.031703 |
| EncodeSydhTfbsGm12878MaxIggmusPk | 0.031687 |
| EncodeHaibTfbsHepg2MaxV0422111PkRep2 | 0.031682 |
| EncodeOpenChromDnaseHsmmtPk | 0.031627 |
| EncodeBroadHistoneOsteoblH3k4me2StdPk | 0.031512 |
| EncodeHaibTfbsH1hescMaxV0422111PkRep2 | 0.031432 |
| EncodeSydhTfbsK562Bhlhe40nb100IggrabPk | 0.031386 |
| EncodeHaibTfbsEcc1Yy1sc281V0422111PkRep2 | 0.031384 |
| EncodeBroadHistoneH1hescRbbp5a300109aStdPk | 0.031277 |
| EncodeSydhTfbsA549MaxIggrabPk | 0.031226 |
| EncodeHaibTfbsHepg2Elf1sc631V0416101PkRep1 | 0.031223 |
| EncodeSydhTfbsK562Pol2Ifng30StdPk | 0.031211 |
| EncodeBroadHistoneNhekPol2bStdPk | 0.031153 |
| EncodeHaibTfbsMcf7MaxV0422111PkRep2 | 0.031149 |
| EncodeBroadHistoneHepg2H3k27acStdPk | 0.031041 |
| EncodeHaibTfbsA549NrsfV0422111Etoh02PkRep2 | 0.030652 |
| EncodeHaibTfbsH1hescSin3ak20Pcr1xPkRep1 | 0.030648 |
| EncodeOpenChromChipHuvecCmycPk | 0.030633 |
| EncodeHaibTfbsK562E2f6sc22823V0416102PkRep2 | 0.030508 |
| EncodeHaibTfbsK562E2f6V0416102PkRep2 | 0.030508 |
| EncodeHaibTfbsMcf7Pmlsc71910V0422111PkRep2 | 0.030435 |
| EncodeHaibTfbsGm12892Taf1V0416102PkRep1 | 0.030429 |
| EncodeBroadHistoneDnd41H2azPk | 0.030417 |
| EncodeADnaseUwCd34mobilizedUniPk | 0.030366 |
| EncodeHaibTfbsGm12878Pu1Pcr1xPkRep2 | 0.030359 |
| EncodeBroadHistoneH1hescH3k9acStdPk | 0.030354 |
| EncodeUwDnaseCd34mobilizedPkRep1 | 0.030350 |
| EncodeUwDnaseMonocd14ro1746PkRep2 | 0.030281 |
| EncodeUwDnaseNb4PkRep2 | 0.030205 |
| EncodeSydhTfbsGm12878Pol2s2IggmusPk | 0.030198 |
| EncodeBroadHistoneNhdfadH2azPk | 0.030193 |
| EncodeSydhTfbsK562Pol2Ifng6hStdPk | 0.030152 |
| EncodeADnaseUwMonocytescd14ro01746UniPk | 0.030138 |
| EncodeOpenChromSynthPanisletsPk | 0.030136 |
| EncodeOpenChromDnaseH9esPk | 0.030118 |
| EncodeHaibTfbsH1hescJundV0416102PkRep2 | 0.030058 |
| EncodeBroadHistoneK562Pol2bStdPk | 0.029952 |
| EncodeHaibTfbsGm12878Ebfsc137065Pcr1xPkRep1 | 0.029942 |
| EncodeSydhTfbsH1hescChd2IggrabPk | 0.029896 |
| EncodeSydhTfbsImr90Mxi1IggrabPk | 0.029728 |
| EncodeHaibTfbsPfsk1Sin3ak20V0416101PkRep2 | 0.029526 |
| EncodeBroadHistoneHuvecPol2bStdPk | 0.029362 |
| EncodeSydhTfbsGm12878Sin3anb6001263IggmusPk | 0.029360 |
| EncodeSydhTfbsHelas3Tcf7l2c9b92565UcdPk | 0.029291 |
| EncodeHaibTfbsGm12878Pou2f2Pcr1xPkRep3 | 0.029290 |
| EncodeHaibTfbsMcf7Elf1V0422111PkRep2 | 0.029231 |
| EncodeBroadHistoneA549H2azEtoh02Pk | 0.029225 |
| EncodeSydhTfbsHepg2Pol2ForsklnStdPk | 0.029187 |
| EncodeOpenChromDnaseIpsPk | 0.029180 |
| EncodeSydhTfbsHelas3Chd2IggrabPk | 0.029024 |
| EncodeSydhTfbsK562JundIggrabPk | 0.028958 |
| EncodeHaibTfbsMcf7GabpV0422111PkRep1 | 0.028880 |
| EncodeHaibTfbsH1neuronsNrsfV0422111PkRep1 | 0.028809 |
| EncodeHaibTfbsK562Egr1V0416101PkRep1 | 0.028799 |
| EncodeADnaseUwHl60UniPk | 0.028794 |
| EncodeSydhTfbsGm12878Tblr1ab24550IggmusPk | 0.028790 |
| EncodeOpenChromDnase8988tPk | 0.028755 |
| EncodeHaibTfbsH1hescSp4v20V0422111PkRep2 | 0.028679 |
| EncodeUwDnaseHl60PkRep1 | 0.028668 |
| EncodeUwDnaseMonocd14ro1746PkRep1V2 | 0.028627 |
| EncodeOpenChromDnaseMedulloPk | 0.028606 |
| EncodeUwDnaseMonocd14PkRep1 | 0.028583 |
| EncodeUwDnaseMonocd14ro1746PkRep1 | 0.028583 |
| EncodeOpenChromDnaseLncapPk | 0.028544 |
| EncodeHaibTfbsHl60Pu1V0422111PkRep2 | 0.028519 |
| EncodeHaibTfbsGm12878Nfatc1sc17834V0422111PkRep1 | 0.028460 |
| EncodeBroadHistoneHmecH3k4me2StdPk | 0.028218 |
| EncodeBroadHistoneGm12878H3k27acStdPk | 0.028215 |
| EncodeSydhTfbsU2osKap1UcdPk | 0.028153 |
| EncodeHaibTfbsHepg2Cebpdsc636V0416101PkRep1 | 0.028151 |
| EncodeBroadHistoneK562Sap3039731StdPk | 0.028142 |
| EncodeBroadHistoneNhlfH2azPk | 0.028108 |
| EncodeHaibTfbsEcc1Tcf12V0422111PkRep1 | 0.028101 |
| EncodeSydhTfbsK562Hcfc1nb10068209IggrabPk | 0.028050 |
| EncodeOpenChromDnaseH1hescPk | 0.028023 |
| EncodeHaibTfbsHepg2Sin3ak20Pcr1xPkRep2 | 0.028004 |
| EncodeHaibTfbsGm12892Pax5c20V0416101PkRep2 | 0.027997 |
| EncodeSydhTfbsGm12878P300bStdPk | 0.027952 |
| EncodeSydhTfbsHek293Pol2StdPk | 0.027929 |
| EncodeHaibTfbsHepg2Taf1Pcr2xPkRep2 | 0.027923 |
| EncodeBroadHistoneNhaH2azPk | 0.027907 |
| EncodeSydhTfbsHepg2MaxIggrabPk | 0.027863 |
| EncodeSydhTfbsHelas3Mxi1af4185IggrabPk | 0.027847 |
| EncodeSydhTfbsGm12878TbpIggmusPk | 0.027824 |
| EncodeSydhTfbsSknshMxi1IggrabPk | 0.027822 |
| EncodeSydhTfbsK562Gtf2bStdPk | 0.027745 |
| EncodeSydhTfbsH1hescMxi1IggrabPk | 0.027654 |
| EncodeHaibTfbsK562Pmlsc71910V0422111PkRep1 | 0.027639 |
| EncodeSydhTfbsK562MaxIggrabPk | 0.027638 |
| EncodeBroadHistoneK562Setdb1Pk | 0.027579 |
| EncodeBroadHistoneHmecH3k9acStdPk | 0.027333 |
| EncodeOpenChromDnaseChorionPk | 0.027308 |
| EncodeHaibTfbsK562Sin3ak20V0416101PkRep2 | 0.027306 |
| EncodeSydhTfbsHelas3Gtf2f1ab28179IggrabPk | 0.027246 |
| EncodeSydhHistoneK562bH3k4me3bUcdPk | 0.027205 |
| EncodeSydhHistoneK562H3k4me3bUcdPk | 0.027205 |
| EncodeHaibTfbsK562Ets1V0416101PkRep2 | 0.027195 |
| EncodeSydhTfbsK562TbpIggmusPk | 0.027175 |
| EncodeHaibTfbsH1hescAtf2sc81188V0422111PkRep2 | 0.027106 |
| EncodeOpenChromFaireGm12878Pk | 0.027105 |
| EncodeSydhTfbsHelas3Elk4UcdPk | 0.027088 |
| EncodeSydhTfbsHelas3MaxIggrabPk | 0.027026 |
| EncodeHaibTfbsGm12891Pu1Pcr1xPkRep1 | 0.026970 |
| EncodeHaibTfbsEcc1Yy1sc281V0422111PkRep1 | 0.026834 |
| EncodeSydhTfbsHelas3Pol2s2IggrabPk | 0.026809 |
| EncodeSydhTfbsHek293Kap1UcdPk | 0.026778 |
| EncodeBroadHistoneK562Hdac2a300705aStdPk | 0.026745 |
| EncodeBroadHistoneK562H3k4me1StdPk | 0.026413 |
| EncodeHaibTfbsK562GabpV0416101PkRep1 | 0.026410 |
| EncodeHaibTfbsGm12891Taf1Pcr1xPkRep2 | 0.026384 |
| EncodeSydhTfbsHepg2Chd2ab68301IggrabPk | 0.026323 |
| EncodeHaibTfbsH1hescMaxV0422111PkRep1 | 0.026204 |
| EncodeSydhTfbsHepg2Corestsc30189IggrabPk | 0.026170 |
| EncodeBroadHistoneGm12878H3k79me2StdPk | 0.026047 |
| EncodeHaibTfbsGm12878Taf1Pcr1xPkRep1 | 0.026014 |
| EncodeSydhTfbsH1hescJundIggrabPk | 0.025910 |
| EncodeHaibTfbsGm12878Zeb1sc25388V0416102PkRep2 | 0.025907 |
| EncodeBroadHistoneK562Sirt6Pk | 0.025742 |
| EncodeSydhTfbsK562CjunIggrabPk | 0.025590 |
| EncodeHaibTfbsGm12878Tcf12Pcr1xPkRep2 | 0.025569 |
| EncodeHaibTfbsHepg2Hdac2sc6296V0416101PkRep1 | 0.025560 |
| EncodeUwDnaseHl60PkRep2 | 0.025504 |
| EncodeOpenChromChipK562CmycPk | 0.025470 |
| EncodeHaibTfbsK562Egr1V0416101PkRep2 | 0.025458 |
| EncodeHaibTfbsK562Tead4sc101184V0422111PkRep1 | 0.025337 |
| EncodeBroadHistoneK562Chd7a301223a1Pk | 0.025285 |
| EncodeBroadHistoneK562Lsd1Pk | 0.025263 |
| EncodeOpenChromChipHepg2CmycPk | 0.025247 |
| EncodeBroadHistoneHsmmH3k4me1StdPk | 0.025216 |
| EncodeSydhTfbsK562Corestsc30189IggrabPk | 0.025178 |
| EncodeOpenChromSynthMedulloPk | 0.025164 |
| EncodeSydhTfbsHepg2Mazab85725IggrabPk | 0.025118 |
| EncodeBroadHistoneNhdfadH3k04me1Pk | 0.024946 |
| EncodeBroadHistoneMonocd14ro1746CtcfPk | 0.024811 |
| EncodeHaibTfbsA549NrsfV0422111Etoh02PkRep1 | 0.024527 |
| EncodeOpenChromFaireHelas3Pk | 0.024500 |
| EncodeHaibTfbsH1hescAtf2sc81188V0422111PkRep1 | 0.024415 |
| EncodeBroadHistoneNhekH3k4me1StdPk | 0.024369 |
| EncodeHaibTfbsK562Cebpbsc150V0422111PkRep1 | 0.024352 |
| EncodeHaibTfbsA549E2f6V0422111PkRep1 | 0.024330 |
| EncodeHaibTfbsSknshYy1sc281V0422111PkRep2 | 0.024284 |
| EncodeHaibTfbsA549GabpV0422111Etoh02PkRep2 | 0.024255 |
| EncodeHaibTfbsA549Usf1V0422111Etoh02PkRep2 | 0.024221 |
| EncodeSydhTfbsHepg2P300sc582IggrabPk | 0.024219 |
| EncodeHaibTfbsHepg2Creb1sc240V0422111PkRep1 | 0.024083 |
| EncodeSydhTfbsHelas3Mazab85725IggrabPk | 0.024052 |
| EncodeHaibTfbsGm12878Sp1Pcr1xPkRep1 | 0.024046 |
| EncodeSydhTfbsH1hescZnf143IggrabPk | 0.024046 |
| EncodeSydhTfbsHelas3Corestsc30189IggrabPk | 0.023924 |
| EncodeHaibTfbsGm12878Bcl11aPcr1xPkRep2 | 0.023922 |
| EncodeHaibTfbsHepg2Taf1Pcr2xPkRep1 | 0.023864 |
| EncodeSydhTfbsGm12878Chd1a301218aIggmusPk | 0.023844 |
| EncodeSydhTfbsK562Znfmizdcp1ab65767IggrabPk | 0.023731 |
| EncodeBroadHistoneHmecH3k27acStdPk | 0.023690 |
| EncodeBroadHistoneCd20CtcfPk | 0.023593 |
| EncodeHaibTfbsHepg2MaxV0422111PkRep1 | 0.023542 |
| EncodeBroadHistoneHsmmtH3k4me1StdPk | 0.023468 |
| EncodeHaibTfbsH1hescCreb1sc240V0422111PkRep2 | 0.023309 |
| EncodeHaibTfbsH1hescCreb1sc240V0422111PkRep1 | 0.023226 |
| EncodeHaibTfbsSknshTaf1V0416101PkRep1 | 0.023190 |
| EncodeHaibTfbsK562Zbtb7asc34508V0416101PkRep2 | 0.023164 |
| EncodeHaibTfbsH1hescSp1Pcr1xPkRep1 | 0.023150 |
| EncodeHaibTfbsK562Pu1Pcr1xPkRep1 | 0.023066 |
| EncodeHaibTfbsK562Hdac2sc6296V0416102PkRep1 | 0.023052 |
| EncodeOpenChromChipMcf7CmycEstroPkRep1 | 0.023046 |
| EncodeBroadHistoneA549H3k04me1Etoh02Pk | 0.023030 |
| EncodeBroadHistoneHmecH2azPk | 0.023010 |
| EncodeHaibTfbsGm12878Ets1Pcr1xPkRep1 | 0.023000 |
| EncodeHaibTfbsGm12878Ets1Pcr1xPkRep2 | 0.023000 |
| EncodeHaibTfbsH1hescGabpPcr1xPkRep1 | 0.022973 |
| EncodeSydhHistoneK562bH3k4me1UcdPk | 0.022872 |
| EncodeSydhHistoneK562H3k4me1UcdPk | 0.022872 |
| EncodeBroadHistoneH1hescH3k27acStdPk | 0.022796 |
| EncodeBroadHistoneMonocd14ro1746H3k79me2Pk | 0.022752 |
| EncodeBroadHistoneK562CtcfStdPk | 0.022728 |
| EncodeSydhTfbsK562Gtf2f1ab28179IggrabPk | 0.022709 |
| EncodeOpenChromDnaseHepatocytesPk | 0.022581 |
| EncodeUwDnaseGm06990PkRep1 | 0.022571 |
| EncodeHaibTfbsMcf7Hdac2sc6296V0422111PkRep1 | 0.022554 |
| EncodeBroadHistoneH1hescChd7a301223a1Pk | 0.022546 |
| EncodeBroadHistoneHepg2H3k04me1StdPk | 0.022527 |
| EncodeSydhHistoneMcf7H3k27acUcdPk | 0.022434 |
| EncodeRegDnaseClusteredV2 | 0.022393 |
| EncodeSydhTfbsMcf10aesPol2TamStdPk | 0.022392 |
| EncodeBroadHistoneK562Nsd2ab75359Pk | 0.022378 |
| EncodeADnaseUm06990UniPk | 0.022376 |
| EncodeSydhTfbsK562Pol2Ifna6hStdPk | 0.022362 |
| EncodeSydhTfbsHct116Tcf7l2UcdPk | 0.022343 |
| EncodeUwDnaseJurkatPkRep2 | 0.022262 |
| EncodeHaibTfbsA549Six5V0422111Etoh02PkRep2 | 0.022216 |
| EncodeHaibTfbsMcf7Sin3ak20V0422111PkRep2 | 0.022169 |
| EncodeHaibTfbsK562Ets1V0416101PkRep1 | 0.022107 |
| EncodeBroadHistoneK562H3k79me2StdPk | 0.022079 |
| EncodeSydhTfbsGm10847Pol2IggmusPk | 0.021962 |
| EncodeSydhTfbsH1hescChd1a301218aIggrabPk | 0.021960 |
| EncodeHaibTfbsGm12878Egr1V0416101PkRep2 | 0.021959 |
| EncodeHaibTfbsK562Nr2f2sc271940V0422111PkRep1 | 0.021931 |
| EncodeSydhTfbsHelas3TbpIggrabPk | 0.021820 |
| EncodeHaibTfbsHct116Sin3ak20V0422111PkRep1 | 0.021791 |
| EncodeUwDnaseJurkatPkRep1 | 0.021735 |
| EncodeUwDnaseCd20ro01778PkRep1 | 0.021665 |
| EncodeADnaseUwJurkatUniPk | 0.021659 |
| EncodeSydhTfbsHepg2Bhlhe40cIggrabPk | 0.021635 |
| EncodeBroadHistoneDnd41CtcfPk | 0.021625 |
| EncodeRegDnaseClustered | 0.021610 |
| EncodeHaibTfbsHepg2NrsfV0416101PkRep2 | 0.021522 |
| EncodeBroadHistoneA549H3k04me1Dex100nmPk | 0.021517 |
| EncodeBroadHistoneNhaH3k4me1StdPk | 0.021503 |
| EncodeHaibTfbsGm12878Irf4sc6059Pcr1xPkRep1 | 0.021482 |
| EncodeSydhTfbsGm12878Corestsc30189IggmusPk | 0.021461 |
| EncodeSydhTfbsK562Tblr1ab24550IggrabPk | 0.021403 |
| EncodeUwDnaseTh1PkRep2 | 0.021364 |
| EncodeHaibTfbsPfsk1Taf1V0416101PkRep1 | 0.021333 |
| EncodeSydhTfbsK562Chd2ab68301IggrabPk | 0.021274 |
| EncodeHaibTfbsA549Fosl2V0422111Etoh02PkRep2 | 0.021201 |
| EncodeUchicagoTfbsK562Egata2ControlPk | 0.021199 |
| EncodeSydhTfbsHelas3Hcfc1nb10068209IggrabPk | 0.021189 |
| EncodeUwHistoneCd20ro01794H3k04me3StdPkRep3 | 0.021057 |
| EncodeHaibTfbsMcf7Taf1V0422111PkRep2 | 0.021025 |
| EncodeUwHistoneGm12865H3k04me3StdPkRep1 | 0.020996 |
| EncodeBroadHistoneH1hescP300kat3bPk | 0.020971 |
| EncodeHaibTfbsA549Foxa2V0416102Etoh02PkRep1 | 0.020971 |
| EncodeSydhTfbsK562P300IggrabPk | 0.020958 |
| EncodeSydhTfbsK562Pol2s2IggrabPk | 0.020950 |
| EncodeHaibTfbsA549Creb1sc240V0416102Dex100nmPkRep2 | 0.020922 |
| EncodeSydhTfbsK562Znf143IggrabPk | 0.020891 |
| EncodeUwHistoneCd20ro01778H3k04me3StdPkRep1 | 0.020871 |
| EncodeADnaseDukeGm18507UniPk | 0.020861 |
| EncodeUwHistoneMonocd14ro1746H3k04me3StdPkRep1 | 0.020830 |
| EncodeSydhTfbsHek293tZnf263UcdPk | 0.020815 |
| EncodeBroadHistoneNhlfH3k4me1StdPk | 0.020802 |
| EncodeHaibTfbsH1neuronsTaf1V0422111PkRep2 | 0.020764 |
| EncodeUwHistoneCd20ro01778H3k04me3StdPkRep2 | 0.020739 |
| EncodeSydhTfbsK562Pol2Ifna30StdPk | 0.020663 |
| EncodeBroadHistoneHelas3H2azPk | 0.020626 |
| EncodeSydhTfbsK562Mxi1af4185IggrabPk | 0.020576 |
| EncodeSydhTfbsPanc1Tcf7l2UcdPk | 0.020575 |
| EncodeHaibTfbsHct116MaxV0422111PkRep2 | 0.020554 |
| EncodeADnaseDukeCllUniPk | 0.020510 |
| EncodeBroadHistoneOsteoblH3k27acStdPk | 0.020507 |
| EncodeUwHistoneGm12865H3k04me3StdPkRep2 | 0.020490 |
| EncodeSydhTfbsGm12878P300IggmusPk | 0.020475 |
| EncodeBroadHistoneHelas3H3k04me1StdPk | 0.020437 |
| EncodeHaibTfbsH1hescSp1Pcr1xPkRep2 | 0.020413 |
| EncodeHaibTfbsHepg2Fosl2V0416101PkRep1 | 0.020410 |
| EncodeHaibTfbsGm12878Pu1Pcr1xPkRep3 | 0.020324 |
| EncodeHaibTfbsHepg2GabpPcr2xPkRep1 | 0.020257 |
| EncodeSydhTfbsHelas3Zkscan1hpa006672IggrabPk | 0.020248 |
| EncodeSydhTfbsK562Ubtfsab1404509IggmusPk | 0.020246 |
| EncodeADnaseDukeUrotheliaUniPk | 0.020229 |
| EncodeUwDnaseTh2wb33676984PkRep1 | 0.020176 |
| EncodeSydhTfbsGm12878Ctcfsc15914c20StdPk | 0.020166 |
| EncodeUwDnaseCd20ro01778PkRep2 | 0.020149 |
| EncodeSydhTfbsGm12891NfkbTnfaIggrabPk | 0.020140 |
| EncodeBroadHistoneGm12878CtcfStdPk | 0.020115 |
| EncodeSydhTfbsHepg2Arid3anb100279IggrabPk | 0.020070 |
| EncodeBroadHistoneHuvecCtcfStdPk | 0.020068 |
| EncodeOpenChromChipMcf7CmycSerumstvdPkRep1 | 0.020052 |
| EncodeUwDnaseHmvecdbladPkRep2 | 0.020048 |
| EncodeADnaseUwCd20UniPk | 0.019998 |
| EncodeOpenChromChipGm12878CmycPk | 0.019997 |
| EncodeSydhTfbsMcf10aesStat3Etoh01StdPk | 0.019996 |
| EncodeBroadHistoneHepg2H2azStdPk | 0.019995 |
| EncodeHaibTfbsA549Usf1Pcr1xDex100nmPkRep1 | 0.019951 |
| EncodeUwDnaseTregwb78495824PkRep1 | 0.019923 |
| EncodeSydhTfbsHelas3Brca1a300IggrabPk | 0.019904 |
| EncodeHaibTfbsEcc1Nficsc81335V0422111PkRep2 | 0.019883 |
| EncodeBroadHistoneNhlfCtcfStdPk | 0.019874 |
| EncodeUwDnaseGm12878PkRep2 | 0.019866 |
| EncodeSydhTfbsK562E2f6UcdPk | 0.019845 |
| EncodeHaibTfbsA549Bcl3V0422111Etoh02PkRep1 | 0.019818 |
| EncodeBroadHistoneHelas3H3k79me2StdPk | 0.019803 |
| EncodeHaibTfbsK562CtcfcPcr1xPkRep1V2 | 0.019752 |
| EncodeSydhTfbsHelas3Rfx5200401194IggrabPk | 0.019740 |
| EncodeUwDnaseHmveclblPkRep2 | 0.019697 |
| EncodeHaibTfbsHepg2Mbd4sc271530V0422111PkRep2 | 0.019674 |
| EncodeSydhTfbsMcf10aesStat3Tam112hHvdPk | 0.019597 |
| EncodeUwDnaseRpmi7951PkRep1 | 0.019472 |
| EncodeUwHistoneGm12878H3k4me3StdPkRep2 | 0.019468 |
| EncodeSydhTfbsHelas3Gcn5StdPk | 0.019420 |
| EncodeUwDnaseRpmi7951PkRep2 | 0.019372 |
| EncodeSydhTfbsMcf10aesStat3TamStdPk | 0.019320 |
| EncodeHaibTfbsHepg2Tead4sc101184V0422111PkRep1 | 0.019254 |
| EncodeUwHistoneGm06990H3k4me3StdPkRep1 | 0.019251 |
| EncodeUwHistoneGm12864H3k04me3StdPkRep1 | 0.019238 |
| EncodeHaibTfbsHepg2JundPcr1xPkRep2 | 0.019231 |
| EncodeADnaseDukeHsmmembUniPk | 0.019207 |
| EncodeSydhTfbsK562Corestab24166IggrabPk | 0.019198 |
| EncodeUwHistoneGm12878H3k4me3StdPkRep1 | 0.019102 |
| EncodeUwHistoneGm12866H3k04me3StdPkRep1 | 0.019084 |
| EncodeBroadHistoneNhdfadH3k79me2Pk | 0.019063 |
| EncodeUwDnaseTh17PkRep1 | 0.019044 |
| EncodeUwHistoneGm06990H3k4me3StdPkRep2 | 0.019033 |
| EncodeUchicagoTfbsK562Ehdac8ControlPk | 0.019027 |
| EncodeHaibTfbsGm12878BatfPcr1xPkRep2 | 0.019026 |
| EncodeBroadHistoneH1hescH3k4me1StdPk | 0.019010 |
| EncodeHaibTfbsGm12878Pax5n19Pcr1xPkRep1 | 0.018986 |
| EncodeUwDnaseTh1PkRep1 | 0.018906 |
| EncodeSydhTfbsHepg2Pol2s2IggrabPk | 0.018896 |
| EncodeSydhTfbsGm12878Elk112771IggmusPk | 0.018848 |
| EncodeUwDnaseHmvecdbladPkRep1 | 0.018794 |
| EncodeADnaseUwHmvecdbladUniPk | 0.018791 |
| EncodeUwHistoneGm12864H3k04me3StdPkRep2 | 0.018773 |
| EncodeHaibTfbsSknshNrsfV0416101PkRep2 | 0.018682 |
| EncodeUwHistoneGm12875H3k04me3StdPkRep1 | 0.018546 |
| EncodeADnaseUwHpfUniPk | 0.018544 |
| EncodeUwDnaseHpfPkRep1 | 0.018517 |
| EncodeHaibTfbsA549P300V0422111Etoh02PkRep2 | 0.018495 |
| EncodeUwDnaseHpafPkRep1 | 0.018491 |
| EncodeUwHistoneHcpeH3k4me3StdPkRep2 | 0.018410 |
| EncodeADnaseUwHpafUniPk | 0.018388 |
| EncodeBroadHistoneA549CtcfEtoh02Pk | 0.018388 |
| EncodeUwHistoneHvmfH3k4me3StdPkRep1 | 0.018386 |
| EncodeOpenChromFaireHepg2Pk | 0.018381 |
| EncodeUwDnaseMscPkRep1 | 0.018380 |
| EncodeHaibTfbsGm12878Bcl3V0416101PkRep1 | 0.018357 |
| EncodeHaibTfbsHelas3GabpPcr1xPkRep2 | 0.018329 |
| EncodeHaibTfbsGm12878Irf4sc6059Pcr1xPkRep2 | 0.018312 |
| EncodeSydhHistonePanc1H3k04me1bUcdPk | 0.018302 |
| EncodeBroadHistoneNhekH3k79me2Pk | 0.018292 |
| EncodeADnaseDukeHtr8svnUniPk | 0.018275 |
| EncodeUwDnaseSknshraPkRep2 | 0.018241 |
| EncodeHaibTfbsEcc1Taf1V0422111PkRep2 | 0.018212 |
| EncodeUwHistoneJurkatH3k4me3StdPkRep2 | 0.018165 |
| EncodeUwDnaseHpafPkRep2 | 0.018139 |
| EncodeSydhTfbsHelas3Elk112771IggrabPk | 0.018122 |
| EncodeUwHistoneJurkatH3k4me3StdPkRep1 | 0.018083 |
| EncodeHaibTfbsK562Stat5asc74442V0422111PkRep1 | 0.018074 |
| EncodeSydhTfbsMcf10aesCfosTamHvdPk | 0.018068 |
| EncodeADnaseUwHmvecdlyneoUniPk | 0.018052 |
| EncodeUwDnaseHmvecdlyneoPkRep1 | 0.018041 |
| EncodeBroadHistoneHmecH3k4me1StdPk | 0.018036 |
| EncodeHaibTfbsSknshGabpV0422111PkRep2 | 0.018033 |
| EncodeUwHistoneHmfH3k4me3StdPkRep2 | 0.018026 |
| EncodeUwDnaseTh1wb54553204PkRep2 | 0.018006 |
| EncodeBroadHistoneHelas3Pol2bStdPk | 0.017998 |
| EncodeUwHistoneNhdfneoH3k4me3StdPkRep2 | 0.017982 |
| EncodeUwHistoneSaecH3k4me3StdPkRep1 | 0.017959 |
| EncodeUwHistoneSaecH3k4me3StdPkRep2 | 0.017950 |
| EncodeHaibTfbsEcc1Tcf12V0422111PkRep2 | 0.017949 |
| EncodeHaibTfbsHl60NrsfV0422111PkRep1 | 0.017946 |
| EncodeADnaseDukeGlioblaUniPk | 0.017931 |
| EncodeHaibTfbsGm12892Yy1V0416101PkRep2 | 0.017926 |
| EncodeUwHistoneHpafH3k4me3StdPkRep1 | 0.017897 |
| EncodeSydhTfbsGm19193NfkbTnfaIggrabPk | 0.017891 |
| EncodeBroadHistoneNhlfH3k79me2Pk | 0.017878 |
| EncodeSydhTfbsGm12878P300sc584IggmusPk | 0.017842 |
| EncodeUwHistoneHeeH3k4me3StdPkRep1 | 0.017824 |
| EncodeUwDnaseM059jPkRep2 | 0.017781 |
| EncodeUwHistoneH7esH3k4me3StdPkRep2 | 0.017772 |
| EncodeUwHistoneHpafH3k4me3StdPkRep2 | 0.017768 |
| EncodeUwHistoneAg10803H3k4me3StdPkRep2 | 0.017750 |
| EncodeUwHistoneHeeH3k4me3StdPkRep2 | 0.017744 |
| EncodeBroadHistoneDnd41H3k79me2Pk | 0.017729 |
| EncodeUwHistoneAg10803H3k4me3StdPkRep1 | 0.017714 |
| EncodeUwDnaseTh2wb54553204PkRep1 | 0.017703 |
| EncodeSydhTfbsHelas3Ini1IggmusPk | 0.017673 |
| EncodeUwHistoneHmecH3k4me3StdPkRep2 | 0.017600 |
| EncodeUwHistoneAg09309H3k4me3StdPkRep1 | 0.017581 |
| EncodeSydhTfbsH1hescBach1sc14700IggrabPk | 0.017577 |
| EncodeUwHistoneHvmfH3k4me3StdPkRep2 | 0.017564 |
| EncodeSydhTfbsH1hescCmycIggrabPk | 0.017553 |
| EncodeUwHistoneHcpeH3k4me3StdPkRep1 | 0.017535 |
| EncodeUwDnaseTh1wb33676984PkRep1 | 0.017530 |
| EncodeHaibTfbsGm12878Mef2aPcr1xPkRep1 | 0.017520 |
| EncodeUwHistoneNhlfH3k04me3StdPkRep1 | 0.017457 |
| EncodeUwHistoneHcmH3k4me3StdPkRep2 | 0.017450 |
| EncodeUwDnaseHcpePkRep1 | 0.017449 |
| EncodeUwHistoneHaspH3k4me3StdPkRep1 | 0.017438 |
| EncodeUwHistoneHuvecH3k4me3StdPkRep1 | 0.017429 |
| EncodeSydhTfbsMcf10aesCfosEtoh01HvdPk | 0.017428 |
| EncodeSydhTfbsHelas3CebpbIggrabPk | 0.017425 |
| EncodeSydhTfbsK562Znf384hpa004051IggrabPk | 0.017416 |
| EncodeUwDnaseHaePkRep1 | 0.017413 |
| EncodeBroadHistoneA549H3k79me2Etoh02Pk | 0.017411 |
| EncodeADnaseUwHcpepicUniPk | 0.017406 |
| EncodeBroadHistoneHuvecH3k79me2Pk | 0.017398 |
| EncodeUwHistoneAg09319H3k4me3StdPkRep1 | 0.017394 |
| EncodeBroadHistoneK562Cbx3sc101004Pk | 0.017390 |
| EncodeUwHistoneHepg2H3k4me3StdPkRep1 | 0.017388 |
| EncodeUwDnaseSkmcPkRep2 | 0.017370 |
| EncodeADnaseUwHaepicUniPk | 0.017363 |
| EncodeUwHistoneHrpeH3k4me3StdPkRep2 | 0.017347 |
| EncodeHaibTfbsHepg2Mbd4sc271530V0422111PkRep1 | 0.017316 |
| EncodeSydhTfbsPbdeGata1UcdPk | 0.017303 |
| EncodeBroadHistoneHsmmH3k79me2StdPk | 0.017298 |
| EncodeUwHistoneHrpeH3k4me3StdPkRep1 | 0.017294 |
| EncodeUwHistoneRptecH3k04me3StdPkRep1 | 0.017285 |
| EncodeUwHistoneHmfH3k4me3StdPkRep1 | 0.017277 |
| EncodeSydhTfbsHelas3E2f4StdPk | 0.017259 |
| EncodeUwHistoneHffH3k04me3StdPkRep1 | 0.017257 |
| EncodeUwHistoneHaspH3k4me3StdPkRep2 | 0.017236 |
| EncodeUwHistoneAg09319H3k4me3StdPkRep2 | 0.017219 |
| EncodeHaibTfbsGm12878Pbx3Pcr1xPkRep2 | 0.017218 |
| EncodeUwHistoneBjH3k4me3StdPkRep2 | 0.017217 |
| EncodeSydhTfbsHelas3Znf143IggrabPk | 0.017201 |
| EncodeUwHistoneHreH3k4me3StdPkRep2 | 0.017197 |
| EncodeBroadHistoneA549H4k20me1Etoh02Pk | 0.017188 |
| EncodeBroadHistoneA549H3k36me3Etoh02Pk | 0.017171 |
| EncodeHaibTfbsHepg2Yy1sc281V0416101PkRep2 | 0.017149 |
| EncodeADnaseDukeStellateUniPk | 0.017144 |
| EncodeSydhTfbsHct116Pol2UcdPk | 0.017138 |
| EncodeADnaseDukeAosmcUniPk | 0.017102 |
| EncodeUwHistoneNhdfneoH3k4me3StdPkRep1 | 0.017065 |
| EncodeUwDnaseHvmfPkRep2 | 0.017064 |
| EncodeBroadHistoneNhaH3k79me2Pk | 0.017057 |
| EncodeADnaseDukeGm19238UniPk | 0.017055 |
| EncodeUwDnaseGm12865PkRep2 | 0.017051 |
| EncodeUwHistoneRptecH3k04me3StdPkRep2 | 0.017048 |
| EncodeUwHistoneHuvecH3k4me3StdPkRep2 | 0.017039 |
| EncodeBroadHistoneHepg2H3k79me2StdPk | 0.017038 |
| EncodeUwHistoneHmecH3k4me3StdPkRep1 | 0.017029 |
| EncodeUwDnaseNhdfadPkRep2 | 0.017008 |
| EncodeUwHistoneHcfH3k4me3StdPkRep2 | 0.017004 |
| EncodeBroadHistoneCd20ro01794H4k20me1Pk | 0.016984 |
| EncodeUwHistoneSknshraH3k4me3StdPkRep1 | 0.016974 |
| EncodeADnaseDukeHpde6e6e7UniPk | 0.016970 |
| EncodeUwDnaseTh2PkRep1 | 0.016969 |
| EncodeHaibTfbsSknshraRad21V0416102PkRep1 | 0.016957 |
| EncodeBroadHistoneNhekCtcfStdPk | 0.016952 |
| EncodeBroadHistoneMonocd14ro1746H4k20me1Pk | 0.016919 |
| EncodeADnaseDukeUrotheliaut189UniPk | 0.016916 |
| EncodeHaibTfbsA549Tcf12V0422111Etoh02PkRep1 | 0.016897 |
| EncodeHaibTfbsK562NrsfV0416102PkRep1 | 0.016883 |
| EncodeUwHistoneHbmecH3k4me3StdPkRep2 | 0.016882 |
| EncodeADnaseUm12865UniPk | 0.016881 |
| EncodeUwHistoneAg04449H3k4me3StdPkRep1 | 0.016880 |
| EncodeUwDnaseGm12865PkRep1 | 0.016878 |
| EncodeADnaseUwHmveclblUniPk | 0.016877 |
| EncodeUwDnaseHmveclblPkRep1 | 0.016876 |
| EncodeUwHistoneSknshraH3k4me3StdPkRep2 | 0.016860 |
| EncodeUwHistoneHepg2H3k4me3StdPkRep2 | 0.016853 |
| EncodeUwHistoneCaco2H3k4me3StdPkRep2 | 0.016829 |
| EncodeUwDnaseGm06990PkRep2 | 0.016825 |
| EncodeUwHistoneHbmecH3k4me3StdPkRep1 | 0.016825 |
| EncodeUwHistoneNhekH3k4me3StdPkRep1 | 0.016821 |
| EncodeBroadHistoneHelas3CtcfStdPk | 0.016782 |
| EncodeHaibTfbsK562CtcfcPcr1xPkRep1 | 0.016777 |
| EncodeSydhTfbsK562Irf1Ifng6hStdPk | 0.016763 |
| EncodeHaibTfbsSknmcFoxp2Pcr2xPkRep2 | 0.016757 |
| EncodeBroadHistoneHsmmtH3k79me2StdPk | 0.016751 |
| EncodeBroadHistoneA549H3k79me2Dex100nmPk | 0.016742 |
| EncodeHaibTfbsHct116Sin3ak20V0422111PkRep2 | 0.016734 |
| EncodeADnaseDukeGm12892UniPk | 0.016728 |
| EncodeUwHistoneK562H3k04me3StdZnf2c10c5PkRep1 | 0.016683 |
| EncodeUwDnaseHepg2PkRep2 | 0.016679 |
| EncodeUwHistoneAg04450H3k4me3StdPkRep2 | 0.016656 |
| EncodeUwHistoneNhekH3k4me3StdPkRep2 | 0.016652 |
| EncodeUwHistoneHreH3k4me3StdPkRep1 | 0.016627 |
| EncodeBroadHistoneOsteoblH2azStdPk | 0.016616 |
| EncodeHaibTfbsK562Cebpdsc636V0422111PkRep1 | 0.016609 |
| EncodeUwHistoneBjH3k4me3StdPkRep1 | 0.016609 |
| EncodeUwHistoneH7esH3k04me3StdDiffa2dPkRep1 | 0.016604 |
| EncodeUchicagoTfbsK562EfosControlPk | 0.016597 |
| EncodeUwHistoneHelas3H3k4me3StdPkRep2 | 0.016591 |
| EncodeSydhTfbsMcf10aesStat3Etoh01bStdPk | 0.016581 |
| EncodeUwDnaseM059jPkRep1 | 0.016535 |
| EncodeUwDnaseHcmPkRep2 | 0.016526 |
| EncodeUwHistoneHcfaaH3k4me3StdPkRep1 | 0.016508 |
| EncodeSydhTfbsHepg2Rfx5200401194IggrabPk | 0.016501 |
| EncodeUwHistoneK562H3k04me3StdZnfa41c6PkRep1 | 0.016479 |
| EncodeUwHistoneHl60H3k4me3StdPkRep1 | 0.016455 |
| EncodeHaibTfbsH1hescE2f6V0422111PkRep2 | 0.016454 |
| EncodeUwDnaseAoafPkRep2 | 0.016441 |
| EncodeUwHistoneSkmcH3k04me3StdPkRep1 | 0.016441 |
| EncodeUwHistoneHacH3k04me3StdPkRep2 | 0.016437 |
| EncodeUwDnaseH7esDiffa2dPkRep1 | 0.016405 |
| EncodeADnaseDukeRwpe1UniPk | 0.016397 |
| EncodeUwHistoneAg04449H3k4me3StdPkRep2 | 0.016395 |
| EncodeSydhTfbsImr90Rad21IggrabPk | 0.016391 |
| EncodeSydhTfbsK562Tblr1nb600270IggrabPk | 0.016377 |
| EncodeUwHistoneK562H3k04me3StdZnfp5PkRep2 | 0.016376 |
| EncodeUwHistoneAoafH3k4me3StdPkRep1 | 0.016375 |
| EncodeUwHistoneAg09309H3k4me3StdPkRep2 | 0.016367 |
| EncodeBroadHistoneHsmmtCtcfStdPk | 0.016366 |
| EncodeUwHistoneCaco2H3k4me3StdPkRep1 | 0.016352 |
| EncodeUwHistoneK562H3k04me3StdZnfp5PkRep1 | 0.016296 |
| EncodeUwHistoneHek293H3k4me3StdPkRep2 | 0.016290 |
| EncodeUwHistoneHl60H3k4me3StdPkRep2 | 0.016282 |
| EncodeUwHistoneA549H3k04me3StdPkRep2 | 0.016274 |
| EncodeUwHistoneWerirb1H3k04me3StdPkRep2 | 0.016254 |
| EncodeHaibTfbsHct116MaxV0422111PkRep1 | 0.016251 |
| EncodeUwDnaseCaco2PkRep1 | 0.016230 |
| EncodeUwHistoneNhlfH3k04me3StdPkRep2 | 0.016226 |
| EncodeUwHistoneK562H3k04me3StdZnf2c10c5PkRep2 | 0.016207 |
| EncodeHaibTfbsGm12878P300Pcr1xPkRep1 | 0.016196 |
| EncodeUwDnaseMscPkRep2 | 0.016195 |
| EncodeUwHistoneK562H3k04me3StdZnff41b2PkRep1 | 0.016195 |
| EncodeUwHistoneSkmcH3k04me3StdPkRep2 | 0.016176 |
| EncodeADnaseUwTh2UniPk | 0.016170 |
| EncodeADnaseDukeFibroblUniPk | 0.016139 |
| EncodeUwDnaseHrePkRep2 | 0.016136 |
| EncodeUwHistoneHct116H3k4me3StdPkRep1 | 0.016134 |
| EncodeUwDnaseHbmecPkRep2 | 0.016115 |
| EncodeUwHistoneHcmH3k4me3StdPkRep1 | 0.016113 |
| EncodeUwHistoneK562H3k04me3StdZnf4c50c4PkRep1 | 0.016112 |
| EncodeUwDnaseHpaecPkRep1 | 0.016094 |
| EncodeHaibTfbsGm12878Bcl11aPcr1xPkRep1 | 0.016085 |
| EncodeUwHistoneK562H3k4me3StdPkRep2 | 0.016061 |
| EncodeUwHistoneAg04450H3k4me3StdPkRep1 | 0.016060 |
| EncodeUwDnaseSknshraPkRep1 | 0.016044 |
| EncodeUwHistoneWerirb1H3k04me3StdPkRep1 | 0.016044 |
| EncodeADnaseUwHpaecUniPk | 0.016038 |
| EncodeUwHistoneWi38H3k04me3StdPkRep2 | 0.016035 |
| EncodeBroadHistoneH1hescSirt6Pk | 0.016023 |
| EncodeUwHistoneHcfH3k4me3StdPkRep1 | 0.015989 |
| EncodeHaibTfbsA549JundV0416102Etoh02PkRep1 | 0.015978 |
| EncodeUwHistoneHacH3k04me3StdPkRep1 | 0.015963 |
| EncodeADnaseDukeFibropUniPk | 0.015950 |
| EncodeUwHistoneAg04450H3k27acStdPkRep1 | 0.015939 |
| EncodeADnaseUwSknshraUniPk | 0.015936 |
| EncodeHaibTfbsHepg2GabpPcr2xPkRep2 | 0.015901 |
| EncodeUwHistoneWi38H3k04me3OhtamStdPkRep1 | 0.015900 |
| EncodeUwHistoneA549H3k04me3StdPkRep1 | 0.015885 |
| EncodeUwHistoneHek293H3k4me3StdPkRep1 | 0.015878 |
| EncodeUwDnaseHs27aPkRep1 | 0.015854 |
| EncodeUwHistoneK562H3k4me3StdPkRep1 | 0.015850 |
| EncodeSydhTfbsMcf10aesCfosTam112hHvdPk | 0.015843 |
| EncodeUwHistoneAoafH3k4me3StdPkRep2 | 0.015839 |
| EncodeSydhTfbsGm12878WhipIggmusPk | 0.015834 |
| EncodeSydhTfbsGm10847NfkbTnfaIggrabPk | 0.015807 |
| EncodeSydhTfbsH1hescMaxUcdPk | 0.015806 |
| EncodeUwDnaseHpfPkRep2 | 0.015782 |
| EncodeADnaseDukeGm12891UniPk | 0.015762 |
| EncodeUwHistoneH7esH3k04me3StdDiffa2dPkRep2 | 0.015751 |
| EncodeUwHistoneHct116H3k4me3StdPkRep2 | 0.015737 |
| EncodeUwDnaseTregwb83319432PkRep1 | 0.015731 |
| EncodeUwHistoneH7esH3k04me3StdDiffa5dPkRep2 | 0.015731 |
| EncodeADnaseDukeTh0UniPk | 0.015703 |
| EncodeUwDnaseCd4naivewb78495824PkRep1 | 0.015692 |
| EncodeBroadHistoneK562Hdac6a301341aPk | 0.015690 |
| EncodeSydhTfbsHuvecCfosUcdPk | 0.015681 |
| EncodeUwDnaseTh1wb54553204PkRep1 | 0.015673 |
| EncodeBroadHistoneA549CtcfDex100nmPk | 0.015636 |
| EncodeHaibTfbsSknshraUsf1sc8983V0416102PkRep2 | 0.015599 |
| EncodeUwHistoneWi38H3k04me3StdPkRep1 | 0.015598 |
| EncodeBroadHistoneA549H3k36me3Dex100nmPk | 0.015585 |
| EncodeSydhTfbsHelas3Smc3ab9263IggrabPk | 0.015573 |
| EncodeUwHistoneWi38H3k04me3OhtamStdPkRep2 | 0.015568 |
| EncodeHaibTfbsMcf7SrfV0422111PkRep1 | 0.015467 |
| EncodeSydhTfbsH1hescUsf2IggrabPk | 0.015467 |
| EncodeUwDnaseHcfPkRep1 | 0.015429 |
| EncodeADnaseUwHcfUniPk | 0.015419 |
| EncodeUwDnaseHmvecdneoPkRep2 | 0.015407 |
| EncodeBroadHistoneK562H3k36me3StdPk | 0.015392 |
| EncodeUwDnaseWi38PkRep1 | 0.015372 |
| EncodeHaibTfbsSknshraUsf1sc8983V0416102PkRep1 | 0.015360 |
| EncodeADnaseUwCaco2UniPk | 0.015350 |
| EncodeUwDnaseTh2PkRep2 | 0.015327 |
| EncodeADnaseUwHmvecdblneoUniPk | 0.015323 |
| EncodeUwHistoneK562H3k04me3StdZnf4c50c4PkRep2 | 0.015322 |
| EncodeUwDnaseHmvecdblneoPkRep1 | 0.015314 |
| EncodeUwDnaseSkmcPkRep1 | 0.015314 |
| EncodeUwHistoneHffmycH3k04me3StdPkRep1 | 0.015310 |
| EncodeBroadHistoneK562H4k20me1StdPk | 0.015304 |
| EncodeADnaseDukePanisletdUniPk | 0.015301 |
| EncodeADnaseUwWi38UniPk | 0.015298 |
| EncodeBroadHistoneNhdfadCtcfStdPk | 0.015297 |
| EncodeBroadHistoneNhekH3k9me1StdPk | 0.015284 |
| EncodeSydhTfbsGm12878Usf2IggmusPk | 0.015276 |
| EncodeHaibTfbsMcf7GabpV0422111PkRep2 | 0.015268 |
| EncodeHaibTfbsGm12878Egr1V0416101PkRep1 | 0.015258 |
| EncodeADnaseUwSkmcUniPk | 0.015243 |
| EncodeUwDnaseHbvsmcPkRep2 | 0.015210 |
| EncodeSydhTfbsK562Zc3h11anb10074650IggrabPk | 0.015195 |
| EncodeUwDnaseHrcePkRep1 | 0.015164 |
| EncodeADnaseUwHrcepicUniPk | 0.015160 |
| EncodeHaibTfbsH1hescTead4sc101184V0422111PkRep2 | 0.015148 |
| EncodeHaibTfbsA549Atf3V0422111Etoh02PkRep2 | 0.015124 |
| EncodeBroadHistoneHsmmCtcfStdPk | 0.015121 |
| EncodeADnaseUwdukeTh1UniPk | 0.015117 |
| EncodeHaibTfbsHepg2Nficsc81335V0422111PkRep2 | 0.015116 |
| EncodeADnaseDukeHuh7UniPk | 0.015111 |
| EncodeUwHistoneHffmycH3k04me3StdPkRep2 | 0.015110 |
| EncodeUwHistoneSknmcH3k04me3StdPkRep2 | 0.015105 |
| EncodeBroadHistoneOsteoblCtcfStdPk | 0.015102 |
| EncodeUwHistoneNb4H3k4me3StdPkRep1 | 0.015082 |
| EncodeHaibTfbsHct116Sp1V0422111PkRep1 | 0.015079 |
| EncodeBroadHistoneNhekH3k36me3StdPk | 0.015058 |
| EncodeBroadHistoneDnd41H4k20me1Pk | 0.015015 |
| EncodeHaibTfbsMcf7Nr2f2sc271940V0422111PkRep1 | 0.015008 |
| EncodeHaibTfbsA549Gata3V0422111PkRep2 | 0.014997 |
| EncodeBroadHistoneOsteoblH3k4me1StdPk | 0.014988 |
| EncodeUwDnaseGm04504PkRep1 | 0.014985 |
| EncodeADnaseUwWerirb1UniPk | 0.014935 |
| EncodeUwDnaseWerirb1PkRep1 | 0.014932 |
| EncodeUwHistonePanc1H3k04me3StdPkRep1 | 0.014926 |
| EncodeUwHistoneK562H3k04me3StdZnff41b2PkRep2 | 0.014920 |
| EncodeHaibTfbsA549Cebpbsc150V0422111PkRep1 | 0.014911 |
| EncodeUwHistoneSknmcH3k04me3StdPkRep1 | 0.014908 |
| EncodeADnaseUwNhdfadUniPk | 0.014894 |
| EncodeUwDnaseNhdfadPkRep1 | 0.014891 |
| EncodeHaibTfbsA549Six5V0422111Etoh02PkRep1 | 0.014865 |
| EncodeBroadHistoneK562PcafPk | 0.014864 |
| EncodeUwDnaseHmvecdlyneoPkRep2 | 0.014856 |
| EncodeUwDnaseHrePkRep2V2 | 0.014854 |
| EncodeUwDnaseHrePkRep1 | 0.014828 |
| EncodeBroadHistoneDnd41H3k36me3Pk | 0.014802 |
| EncodeBroadHistoneHsmmH3k36me3StdPk | 0.014789 |
| EncodeHaibTfbsHepg2Usf1Pcr1xPkRep2 | 0.014774 |
| EncodeADnaseDukeIshikawatamoxifenUniPk | 0.014767 |
| EncodeUwHistoneHelas3H3k4me3StdPkRep1 | 0.014765 |
| EncodeUwHistoneH7esH3k4me3StdPkRep1 | 0.014728 |
| EncodeHaibTfbsEcc1Creb1sc240V0422111PkRep2 | 0.014702 |
| EncodeBroadHistoneHepg2CtcfStdPk | 0.014693 |
| EncodeHaibTfbsK562Cebpdsc636V0422111PkRep2 | 0.014691 |
| EncodeADnaseDukeIshikawaestradiolUniPk | 0.014664 |
| EncodeUwDnaseHcfPkRep2 | 0.014663 |
| EncodeHaibTfbsK562Usf1V0416101PkRep1 | 0.014658 |
| EncodeADnaseDukeHelas3ifna4hUniPk | 0.014656 |
| EncodeUwDnaseHconfPkRep1 | 0.014626 |
| EncodeUwHistoneLncapH3k04me3StdPkRep2 | 0.014625 |
| EncodeADnaseUwHconfUniPk | 0.014617 |
| EncodeBroadHistoneH1hescH3k79me2StdPk | 0.014592 |
| EncodeOpenChromFaireHtr8Pk | 0.014592 |
| EncodeUwDnaseHuvecPkRep1 | 0.014580 |
| EncodeHaibTfbsK562Atf3V0416101PkRep2 | 0.014565 |
| EncodeUwDnaseHaePkRep2 | 0.014546 |
| EncodeUwHistoneHpfH3k4me3StdPkRep1 | 0.014524 |
| EncodeUwDnaseCd4naivewb11970640PkRep1 | 0.014518 |
| EncodeSydhTfbsMcf10aesStat3Etoh01cStdPk | 0.014508 |
| EncodeHaibTfbsSknshNrsfV0416101PkRep1 | 0.014496 |
| EncodeUwDnaseH7esPkRep2 | 0.014478 |
| EncodeSydhTfbsK562CmycIfna30StdPk | 0.014452 |
| EncodeHaibTfbsHepg2Cebpdsc636V0416101PkRep2 | 0.014437 |
| EncodeUwDnaseCaco2PkRep2 | 0.014429 |
| EncodeSydhTfbsA549CtcfbIggrabPk | 0.014417 |
| EncodeUwDnaseGm12878PkRep1 | 0.014404 |
| EncodeHaibTfbsMcf7Taf1V0422111PkRep1 | 0.014403 |
| EncodeUwHistoneLncapH3k04me3StdPkRep1 | 0.014403 |
| EncodeHaibTfbsMcf7Hdac2sc6296V0422111PkRep2 | 0.014356 |
| EncodeADnaseUwHmfUniPk | 0.014352 |
| EncodeUwDnaseHmfPkRep1 | 0.014321 |
| EncodeSydhTfbsImr90CebpbIggrabPk | 0.014262 |
| EncodeUwDnaseWi38OhtamPkRep1 | 0.014255 |
| EncodeADnaseUwWi38tamoxifentamoxifenUniPk | 0.014210 |
| EncodeHaibTfbsSknshNrsfPcr2xPkRep2 | 0.014204 |
| EncodeUwDnaseHipePkRep2 | 0.014186 |
| EncodeADnaseDukeMelanoUniPk | 0.014172 |
| EncodeHaibTfbsK562Rad21V0416102PkRep2 | 0.014162 |
| EncodeSydhTfbsK562Elk112771IggrabPk | 0.014150 |
| EncodeUwDnaseH7esPkRep1 | 0.014150 |
| EncodeADnaseDukeGm19240UniPk | 0.014093 |
| EncodeUwDnaseLhcnm2PkRep1 | 0.014089 |
| EncodeSydhTfbsHelas3MafkIggrabPk | 0.014073 |
| EncodeUwDnaseHpdlfPkRep1 | 0.014057 |
| EncodeUwDnaseH7esDiffa9dPkRep1 | 0.014056 |
| EncodeADnaseUwHpdlfUniPk | 0.014049 |
| EncodeSydhTfbsHepg2CebpbIggrabPk | 0.014041 |
| EncodeBroadHistoneH1hescCtcfStdPk | 0.014036 |
| EncodeUwDnaseHrePkRep1V2 | 0.014008 |
| EncodeADnaseUwHreUniPk | 0.014006 |
| EncodeUwDnaseAoafPkRep1 | 0.013993 |
| EncodeUwDnaseHmfPkRep2 | 0.013982 |
| EncodeBroadHistoneNhekH4k20me1StdPk | 0.013981 |
| EncodeUwHistonePanc1H3k04me3StdPkRep2 | 0.013972 |
| EncodeHaibTfbsK562Atf3V0416101PkRep1 | 0.013967 |
| EncodeADnaseDukePanisletsUniPk | 0.013947 |
| EncodeADnaseUwSaecUniPk | 0.013946 |
| EncodeUwDnaseSaecPkRep1 | 0.013946 |
| EncodeUwDnaseHcmPkRep1 | 0.013911 |
| EncodeHaibTfbsA549Fosl2V0422111Etoh02PkRep1 | 0.013907 |
| EncodeBroadHistoneK562H3k9me1StdPk | 0.013905 |
| EncodeADnaseUwHcmUniPk | 0.013897 |
| EncodeUwHistoneK562H3k04me3StdZnfa41c6PkRep2 | 0.013887 |
| EncodeADnaseUwAoafUniPk | 0.013870 |
| EncodeHaibTfbsEcc1P300V0422111PkRep1 | 0.013849 |
| EncodeUwDnaseHrcePkRep2 | 0.013843 |
| EncodeSydhHistoneHct116H3k04me1UcdPk | 0.013817 |
| EncodeHaibTfbsH1neuronsTaf1V0422111PkRep1 | 0.013802 |
| EncodeADnaseUwHvmfUniPk | 0.013792 |
| EncodeUwDnaseHvmfPkRep1 | 0.013769 |
| EncodeUwDnaseHgfPkRep1 | 0.013766 |
| EncodeHaibTfbsT47dFoxa1sc6553V0416102Dm002p1hPkRep2 | 0.013751 |
| EncodeADnaseUwHgfUniPk | 0.013746 |
| EncodeSydhTfbsK562CebpbIggrabPk | 0.013739 |
| EncodeHaibTfbsGm12878Ets1Pcr1xPkRep2V2 | 0.013734 |
| EncodeHaibTfbsEcc1Tead4sc101184V0422111PkRep2 | 0.013722 |
| EncodeSydhTfbsK562CmycIfng6hStdPk | 0.013721 |
| EncodeUwDnaseHpdlfPkRep2 | 0.013701 |
| EncodeHaibTfbsPfsk1Sin3ak20V0416101PkRep1 | 0.013685 |
| EncodeHaibTfbsSknshSin3ak20V0416101PkRep1 | 0.013660 |
| EncodeSydhTfbsA549Pol2s2IggrabPk | 0.013648 |
| EncodeUwDnaseHepg2PkRep1 | 0.013640 |
| EncodeUwDnaseCmkPkRep1 | 0.013627 |
| EncodeHaibTfbsH1hescRad21V0416102PkRep2 | 0.013620 |
| EncodeUwDnaseHuvecPkRep1V2 | 0.013592 |
| EncodeADnaseUwCmkUniPk | 0.013590 |
| EncodeHaibTfbsHepg2JundPcr1xPkRep1 | 0.013553 |
| EncodeHaibTfbsA549Zbtb33V0422111Etoh02PkRep1 | 0.013546 |
| EncodeBroadHistoneHepg2H3k27me3StdPk | 0.013535 |
| EncodeADnaseUwdukeHepg2UniPk | 0.013496 |
| EncodeBroadHistoneHsmmtH4k20me1StdPk | 0.013354 |
| EncodeUwDnaseGm04504PkRep2 | 0.013353 |
| EncodeBroadHistoneH1hescSuz12051317Pk | 0.013351 |
| EncodeSydhTfbsK562NelfeStdPk | 0.013330 |
| EncodeUwHistoneH7esH3k04me3StdDiffa9dPkRep2 | 0.013307 |
| EncodeOpenChromChipMcf7CtcfSerumstimPkRep1 | 0.013303 |
| EncodeBroadHistoneNhlfH3k36me3StdPk | 0.013298 |
| EncodeBroadHistoneMonocd14ro1746H3k36me3Pk | 0.013292 |
| EncodeHaibTfbsHepg2Foxa1sc101058V0416101PkRep2 | 0.013292 |
| EncodeSydhTfbsK562Ubfsc13125IggmusPk | 0.013274 |
| EncodeUwDnaseHcpePkRep2 | 0.013272 |
| EncodeSydhTfbsImr90CtcfbIggrabPk | 0.013269 |
| EncodeUwDnaseBjPkRep2 | 0.013247 |
| EncodeUwHistoneMcf7H3k04me3StdPkRep2 | 0.013247 |
| EncodeUwHistoneH7esH3k04me3StdDiffa9dPkRep1 | 0.013217 |
| EncodeHaibTfbsHepg2Sp1Pcr1xPkRep1 | 0.013213 |
| EncodeHaibTfbsEcc1SrfV0422111PkRep1 | 0.013151 |
| EncodeHaibTfbsGm12878Pu1Pcr1xPkRep1 | 0.013148 |
| EncodeSydhTfbsMcf10aesCfosTam14hHvdPk | 0.013144 |
| EncodeHaibTfbsEcc1Tead4sc101184V0422111PkRep1 | 0.013128 |
| EncodeHaibTfbsK562Creb1sc240V0422111PkRep1 | 0.013075 |
| EncodeUwHistoneH7esH3k04me3StdDiffa14dPkRep2 | 0.013051 |
| EncodeUwHistoneH7esH3k04me3StdPkRep2 | 0.013051 |
| EncodeBroadHistoneK562Chd4mi2Pk | 0.013037 |
| EncodeUwDnaseHacPkRep2 | 0.013032 |
| EncodeADnaseDukePhteUniPk | 0.013026 |
| EncodeSydhTfbsHepg2Brca1a300IggrabPk | 0.013023 |
| EncodeUwDnaseHs5PkRep1 | 0.013021 |
| EncodeUwDnaseHmvecdblneoPkRep2 | 0.012986 |
| EncodeOpenChromChipMcf7CtcfSerumstvdPkRep1 | 0.012975 |
| EncodeSydhTfbsK562CtcfbIggrabPk | 0.012968 |
| EncodeADnaseDukeMedulloUniPk | 0.012959 |
| EncodeHaibTfbsK562Pu1Pcr1xPkRep2 | 0.012953 |
| EncodeHaibTfbsSknshPbx3V0422111PkRep2 | 0.012938 |
| EncodeADnaseDukeT47dUniPk | 0.012932 |
| EncodeBroadHistoneK562Chd1a301218aStdPk | 0.012930 |
| EncodeUwDnaseHconfPkRep2 | 0.012929 |
| EncodeHaibTfbsK562Yy1sc281V0416101PkRep2 | 0.012909 |
| EncodeHaibTfbsK562Yy1V0416101PkRep2 | 0.012909 |
| EncodeSydhTfbsHepg2Hsf1ForsklnStdPk | 0.012879 |
| EncodeUwHistoneHpfH3k4me3StdPkRep2 | 0.012868 |
| EncodeBroadHistoneGm12878H3k36me3StdPk | 0.012845 |
| EncodeUwHistoneMcf7H3k4me3StdPkRep1 | 0.012825 |
| EncodeSydhTfbsGm12878Smc3ab9263IggmusPk | 0.012820 |
| EncodeSydhTfbsH1hescRad21IggrabPk | 0.012792 |
| EncodeBroadHistoneNhaH4k20me1Pk | 0.012788 |
| EncodeSydhTfbsGm12878Nrf1IggmusPk | 0.012786 |
| EncodeBroadHistoneHsmmtH3k36me3StdPk | 0.012780 |
| EncodeHaibTfbsEcc1Usf1V0422111PkRep2 | 0.012776 |
| EncodeHaibTfbsEcc1Usf1V0422111PkRep1 | 0.012767 |
| EncodeADnaseDukeHuh75UniPk | 0.012751 |
| EncodeBroadHistoneH1hescHdac6a301341aPk | 0.012727 |
| EncodeBroadHistoneHmecH3k79me2Pk | 0.012725 |
| EncodeHaibTfbsT47dGata3sc268V0416102Dm002p1hPkRep2 | 0.012712 |
| EncodeSydhTfbsH1hescCebpbIggrabPk | 0.012644 |
| EncodeHaibTfbsMcf7Cebpbsc150V0422111PkRep1 | 0.012579 |
| EncodeUwDnaseSaecPkRep2 | 0.012574 |
| EncodeADnaseUm12864UniPk | 0.012560 |
| EncodeUwDnaseGm12864PkRep1 | 0.012551 |
| EncodeHaibTfbsK562CtcfcPcr1xPkRep2 | 0.012480 |
| EncodeBroadHistoneNhdfadH3k36me3StdPk | 0.012424 |
| EncodeADnaseUwdukeGm12878UniPk | 0.012415 |
| EncodeHaibTfbsHelas3Taf1Pcr1xPkRep1 | 0.012410 |
| EncodeADnaseDukeProgfibUniPk | 0.012383 |
| EncodeUwDnasePrecPkRep2 | 0.012381 |
| EncodeUwHistoneH7esH3k04me3StdDiffa14dPkRep1 | 0.012365 |
| EncodeADnaseDukeGm19239UniPk | 0.012299 |
| EncodeOpenChromChipHelas3CmycPk | 0.012274 |
| EncodeHaibTfbsPanc1Sin3ak20V0416101PkRep2 | 0.012260 |
| EncodeBroadHistoneHuvecH3k36me3StdPk | 0.012258 |
| EncodeUwDnaseNhaPkRep2 | 0.012238 |
| EncodeADnaseUwHacUniPk | 0.012208 |
| EncodeHaibTfbsT47dP300V0416102Dm002p1hPkRep2 | 0.012203 |
| EncodeHaibTfbsH1hescCtcfsc5916V0416102PkRep1 | 0.012197 |
| EncodeHaibTfbsEcc1Nficsc81335V0422111PkRep1 | 0.012157 |
| EncodeBroadHistoneGm12878Ezh239875Pk | 0.012123 |
| EncodeOpenChromChipMcf7CtcfPk | 0.012119 |
| EncodeUwDnaseHbvsmcPkRep1 | 0.012116 |
| EncodeHaibTfbsHepg2Ctcfsc5916V0416101PkRep1 | 0.012103 |
| EncodeSydhTfbsH1hescCtbp2UcdPk | 0.012094 |
| EncodeBroadHistoneDnd41Ezh239875Pk | 0.012093 |
| EncodeOpenChromFaireUrotsaUt189Pk | 0.012089 |
| EncodeHaibTfbsSknshUsf1V0422111PkRep2 | 0.012087 |
| EncodeHaibTfbsH1hescCtcfsc5916V0416102PkRep2 | 0.012034 |
| EncodeSydhTfbsSknshCtcfbIggrabPk | 0.012027 |
| EncodeOpenChromFaireUrotsaPk | 0.012019 |
| EncodeUwDnaseLhcnm2Diff4dPkRep2 | 0.012005 |
| EncodeUwDnaseWi38OhtamPkRep2 | 0.011962 |
| EncodeUwDnaseHacPkRep1 | 0.011942 |
| EncodeUwDnaseHrgecPkRep1 | 0.011919 |
| EncodeUwDnaseWi38PkRep2 | 0.011902 |
| EncodeBroadHistoneH1hescH2azStdPk | 0.011899 |
| EncodeADnaseUwdukeHuvecUniPk | 0.011893 |
| EncodeHaibTfbsHepg2Rad21V0416101PkRep1 | 0.011868 |
| EncodeUwDnaseH7esPkRep1V2 | 0.011867 |
| EncodeHaibTfbsSknshraP300V0416102PkRep2 | 0.011864 |
| EncodeADnaseUwH7hescUniPk | 0.011858 |
| EncodeUwDnaseHgfPkRep2 | 0.011835 |
| EncodeSydhTfbsK562Cdpsc6327IggrabPk | 0.011819 |
| EncodeHaibTfbsA549Foxa2V0416102Etoh02PkRep2 | 0.011792 |
| EncodeRegTfbsClustered | 0.011778 |
| EncodeHaibTfbsK562Cbx3sc101004V0422111PkRep2 | 0.011775 |
| EncodeRegTfbsClusteredV2 | 0.011758 |
| EncodeUwHistoneH7esH3k04me3StdDiffa5dPkRep1 | 0.011757 |
| EncodeUwHistoneH7esH3k04me3StdPkRep1 | 0.011757 |
| EncodeOpenChromFaireNhekPk | 0.011743 |
| EncodeHaibTfbsGm12892Taf1V0416102PkRep2 | 0.011729 |
| EncodeHaibTfbsH1hescTead4sc101184V0422111PkRep1 | 0.011727 |
| EncodeBroadHistoneGm12878H4k20me1StdPk | 0.011726 |
| EncodeBroadHistoneHepg2H3k36me3StdPk | 0.011712 |
| EncodeUwDnaseHmvecllyPkRep1 | 0.011709 |
| EncodeHaibTfbsGm12878SrfV0416101PkRep2 | 0.011705 |
| EncodeADnaseUwHmvecllyUniPk | 0.011700 |
| EncodeOpenChromChipFibroblCtcfPkRep1 | 0.011697 |
| EncodeOpenChromChipGm12892CtcfPk | 0.011694 |
| EncodeUwDnaseHbvpPkRep1 | 0.011688 |
| EncodeSydhTfbsGm18505NfkbTnfaIggrabPk | 0.011647 |
| EncodeBroadHistoneNhaCtcfStdPk | 0.011632 |
| EncodeUwDnaseHcfaaPkRep1 | 0.011625 |
| EncodeSydhTfbsImr90Corestsc30189IggrabPk | 0.011596 |
| EncodeSydhTfbsHelas3Usf2IggmusPk | 0.011524 |
| EncodeADnaseUwHcfaaUniPk | 0.011523 |
| EncodeSydhTfbsHelas3E2f6StdPk | 0.011509 |
| EncodeOpenChromChipA549CtcfPkRep1 | 0.011497 |
| EncodeBroadHistoneHelas3H3k36me3StdPk | 0.011481 |
| EncodeUwDnaseHmvecdadPkRep1 | 0.011446 |
| EncodeADnaseUwHrgecUniPk | 0.011433 |
| EncodeUwDnaseHrgecPkRep2 | 0.011416 |
| EncodeADnaseUwHmvecdadUniPk | 0.011375 |
| EncodeADnaseUwHffUniPk | 0.011373 |
| EncodeUwDnaseHffPkRep1 | 0.011371 |
| EncodeHaibTfbsSknshraP300V0416102PkRep1 | 0.011348 |
| EncodeHaibTfbsSknshPbx3V0422111PkRep1 | 0.011345 |
| EncodeSydhTfbsGm12878Znf384hpa004051IggmusPk | 0.011339 |
| EncodeUwDnasePanc1PkRep1 | 0.011333 |
| EncodeUwDnaseNhdfneoPkRep1 | 0.011321 |
| EncodeBroadHistoneOsteoH3k79me2Pk | 0.011309 |
| EncodeADnaseUwNhdfneoUniPk | 0.011299 |
| EncodeOpenChromChipGm19238CtcfPk | 0.011288 |
| EncodeUwDnaseNhlfPkRep2 | 0.011268 |
| EncodeADnaseUwNhaUniPk | 0.011243 |
| EncodeUwDnaseNhaPkRep1 | 0.011242 |
| EncodeHaibTfbsA549Usf1Pcr1xEtoh02PkRep1 | 0.011240 |
| EncodeBroadHistoneH1hescH4k20me1StdPk | 0.011230 |
| EncodeBroadHistoneK562Rnf2Pk | 0.011224 |
| EncodeADnaseDukeIpsUniPk | 0.011220 |
| EncodeBroadHistoneK562Ezh239875StdPk | 0.011200 |
| EncodeUwDnaseH7esDiffa5dPkRep1 | 0.011193 |
| EncodeADnaseUwPanc1UniPk | 0.011185 |
| EncodeHaibTfbsGm12891Yy1sc281V0416101PkRep1 | 0.011180 |
| EncodeHaibTfbsGm12891Pu1Pcr1xPkRep2 | 0.011160 |
| EncodeHaibTfbsK562Zbtb7asc34508V0416101PkRep1 | 0.011150 |
| EncodeHaibTfbsGm12878Zbtb33Pcr1xPkRep2 | 0.011129 |
| EncodeOpenChromChipGm19240CtcfPk | 0.011118 |
| EncodeSydhTfbsHepg2JundIggrabPk | 0.011115 |
| EncodeSydhTfbsK562Mafkab50322IggrabPk | 0.011106 |
| EncodeUwDnaseNt2d1PkRep1 | 0.011091 |
| EncodeADnaseUwNt2d1UniPk | 0.011089 |
| EncodeUwDnaseHmvecllyPkRep2 | 0.011053 |
| EncodeBroadHistoneH1hescJarid1aab26049StdPk | 0.011041 |
| EncodeHaibTfbsH1hescP300V0416102PkRep2 | 0.011012 |
| EncodeUwDnaseHsmmtPkRep2 | 0.011011 |
| EncodeSydhTfbsGm18526NfkbTnfaIggrabPk | 0.011010 |
| EncodeHaibTfbsT47dCtcfsc5916V0416102Dm002p1hPkRep2 | 0.010977 |
| EncodeBroadHistoneOsteoP300kat3bPk | 0.010968 |
| EncodeBroadHistoneHepg2H4k20me1StdPk | 0.010965 |
| EncodeADnaseUwAg04450UniPk | 0.010951 |
| EncodeSydhTfbsH1hescBrca1IggrabPk | 0.010938 |
| EncodeUwDnaseAg04450PkRep1 | 0.010932 |
| EncodeOpenChromFaireGlioblaPk | 0.010920 |
| EncodeHaibTfbsA549Usf1Pcr1xEtoh02PkRep2 | 0.010902 |
| EncodeHaibTfbsH1hescUsf1Pcr1xPkRep2 | 0.010897 |
| EncodeUwDnaseT47dPkRep1 | 0.010875 |
| EncodeADnaseUwHbmecUniPk | 0.010835 |
| EncodeUwDnaseHbmecPkRep1 | 0.010835 |
| EncodeADnaseDukeOsteoblUniPk | 0.010830 |
| EncodeHaibTfbsEcc1Zbtb7aV0422111PkRep2 | 0.010826 |
| EncodeHaibTfbsSknshraCtcfV0416102PkRep2 | 0.010813 |
| EncodeSydhHistoneNt2d1H3k4me1UcdPk | 0.010804 |
| EncodeHaibTfbsA549P300V0422111Etoh02PkRep1 | 0.010800 |
| EncodeUwDnaseHuvecPkRep2 | 0.010784 |
| EncodeBroadHistoneNhaH3k36me3StdPk | 0.010777 |
| EncodeOpenChromChipGm12891CtcfPk | 0.010774 |
| EncodeHaibTfbsK562Gata2sc267Pcr1xPkRep1 | 0.010768 |
| EncodeHaibTfbsH1hescTcf12Pcr1xPkRep2 | 0.010767 |
| EncodeSydhTfbsK562CmycIfna6hStdPk | 0.010758 |
| EncodeHaibTfbsGm12878Mef2aPcr1xPkRep2 | 0.010749 |
| EncodeSydhTfbsHepg2Rad21IggrabPk | 0.010696 |
| EncodeOpenChromChipProgfibCtcfPkRep1 | 0.010691 |
| EncodeADnaseDukeLncapandrogenUniPk | 0.010644 |
| EncodeHaibTfbsH1hescE2f6V0422111PkRep1 | 0.010611 |
| EncodeHaibTfbsHepg2Hnf4gsc6558V0416101PkRep1 | 0.010597 |
| EncodeBroadHistoneH1hescEzh239875Pk | 0.010569 |
| EncodeSydhTfbsMcf10aesPol2Etoh01StdPk | 0.010566 |
| EncodeHaibTfbsA549Ctcfsc5916Pcr1xDex100nmPkRep1 | 0.010564 |
| EncodeADnaseUwNhlfUniPk | 0.010559 |
| EncodeUwDnaseNhlfPkRep1 | 0.010558 |
| EncodeUwDnaseH7esDiffa5dPkRep2 | 0.010556 |
| EncodeOpenChromChipGm19239CtcfPk | 0.010548 |
| EncodeUwDnaseHcfaaPkRep2 | 0.010538 |
| EncodeOpenChromChipGm10266CtcfPkRep1 | 0.010535 |
| EncodeSydhTfbsHelas3Baf170IggmusPk | 0.010534 |
| EncodeUwDnaseHipePkRep1 | 0.010526 |
| EncodeHaibTfbsA549Ctcfsc5916Pcr1xEtoh02PkRep1 | 0.010525 |
| EncodeHaibTfbsEcc1Cebpbsc150V0422111PkRep1 | 0.010525 |
| EncodeADnaseUwHipepicUniPk | 0.010515 |
| EncodeSydhTfbsK562Irf1Ifng30StdPk | 0.010489 |
| EncodeUwDnaseHmvecdlyadPkRep2 | 0.010477 |
| EncodeSydhTfbsGm12878Rfx5200401194IggmusPk | 0.010474 |
| EncodeSydhTfbsK562Atf3StdPk | 0.010456 |
| EncodeSydhTfbsHepg2Smc3ab9263IggrabPk | 0.010430 |
| EncodeHaibTfbsA549Atf3V0422111Etoh02PkRep1 | 0.010407 |
| EncodeBroadHistoneHsmmH4k20me1StdPk | 0.010393 |
| EncodeHaibTfbsA549Rad21V0422111PkRep1 | 0.010389 |
| EncodeHaibTfbsSknshFosl2V0422111PkRep1 | 0.010389 |
| EncodeUwDnaseH1hescPkRep1 | 0.010381 |
| EncodeOpenChromChipGm13976CtcfPkRep1 | 0.010376 |
| EncodeADnaseDukeMyometrUniPk | 0.010374 |
| EncodeADnaseDukeH9esUniPk | 0.010331 |
| EncodeUwTfbsGm12871CtcfStdPkRep2 | 0.010331 |
| EncodeSydhTfbsA549CebpbIggrabPk | 0.010325 |
| EncodeHaibTfbsSknshZbtb33V0422111PkRep2 | 0.010299 |
| EncodeHaibTfbsHepg2Zbtb7aV0416101PkRep2 | 0.010298 |
| EncodeOpenChromChipGm10248CtcfPkRep1 | 0.010293 |
| EncodeHaibTfbsA549GrPcr1xDex50nmPkRep2 | 0.010258 |
| EncodeUwDnaseHaspPkRep2 | 0.010253 |
| EncodeOpenChromFaireMrtg4016Pk | 0.010252 |
| EncodeUwDnasePrecPkRep1 | 0.010231 |
| EncodeSydhTfbsGm12878NfybIggmusPk | 0.010204 |
| EncodeADnaseUwPrecUniPk | 0.010202 |
| EncodeHaibTfbsMcf7CtcfcV0422111PkRep1 | 0.010202 |
| EncodeUwDnaseNhdfneoPkRep2 | 0.010199 |
| EncodeHaibTfbsEcc1Zbtb7aV0422111PkRep1 | 0.010196 |
| EncodeOpenChromChipGlioblaCtcfPkRep1 | 0.010181 |
| EncodeUwDnaseHsmmtPkRep1 | 0.010179 |
| EncodeSydhTfbsK562Smc3ab9263IggrabPk | 0.010160 |
| EncodeUwDnaseSknmcPkRep2 | 0.010156 |
| EncodeUwTfbsGm12874CtcfStdPkRep1 | 0.010106 |
| EncodeSydhTfbsHek293Elk4UcdPk | 0.010090 |
| EncodeHaibTfbsA549Usf1Pcr1xDex100nmPkRep2 | 0.010086 |
| EncodeSydhTfbsK562E2f4UcdPk | 0.010075 |
| EncodeOpenChromChipMcf7CtcfVehPkRep1 | 0.010068 |
| EncodeHaibTfbsGm12891Pax5c20V0416101PkRep1 | 0.010064 |
| EncodeADnaseDuke8988tUniPk | 0.010061 |
| EncodeADnaseUwHmvecdneoUniPk | 0.010010 |
| EncodeSydhTfbsGm12878Srebp1IggrabPk | 0.010008 |
| EncodeADnaseDukeMcf7hypoxiaUniPk | 0.009997 |
| EncodeUwDnaseK562Znfp5PkRep1 | 0.009996 |
| EncodeHaibTfbsGm12878Creb1sc240V0422111PkRep2 | 0.009995 |
| EncodeUwDnaseHmvecdneoPkRep1 | 0.009981 |
| EncodeHaibTfbsGm12878Egr1Pcr2xPkRep3 | 0.009956 |
| EncodeHaibTfbsA549GrPcr2xDex100nmPkRep1 | 0.009951 |
| EncodeHaibTfbsHepg2Atf3V0416101PkRep2 | 0.009951 |
| EncodeBroadHistoneNhlfEzh239875Pk | 0.009936 |
| EncodeOpenChromChipLungocCtcfPkRep1 | 0.009936 |
| EncodeUwTfbsGm12865CtcfStdPkRep1 | 0.009936 |
| EncodeBroadHistoneHepg2Ezh239875Pk | 0.009925 |
| EncodeHaibTfbsEcc1Rad21V0422111PkRep1 | 0.009923 |
| EncodeHaibTfbsA549Yy1cV0422111Etoh02PkRep2 | 0.009915 |
| EncodeHaibTfbsK562Sp1Pcr1xPkRep1 | 0.009909 |
| EncodeSydhTfbsK562Arid3asc8821IggrabPk | 0.009864 |
| EncodeHaibTfbsGm12878Pbx3Pcr1xPkRep1 | 0.009849 |
| EncodeOpenChromChipGm12878CtcfPkRep1 | 0.009844 |
| EncodeUwDnaseGm04503PkRep1 | 0.009837 |
| EncodeOpenChromChipPancreasocCtcfPkRep1 | 0.009811 |
| EncodeSydhTfbsA549CmycIggrabPk | 0.009798 |
| EncodeUwDnaseHahPkRep1 | 0.009776 |
| EncodeHaibTfbsK562Fosl1sc183V0416101PkRep1 | 0.009768 |
| EncodeADnaseUwHahUniPk | 0.009739 |
| EncodeHaibTfbsMcf7Egr1V0422111PkRep2 | 0.009738 |
| EncodeBroadHistoneK562P300StdPk | 0.009711 |
| EncodeHaibTfbsMcf7CtcfcV0422111PkRep2 | 0.009708 |
| EncodeHaibTfbsHepg2Creb1sc240V0422111PkRep2 | 0.009701 |
| EncodeHaibTfbsK562Ctcflsc98982V0416101PkRep2 | 0.009688 |
| EncodeBroadHistoneK562Suz12051317Pk | 0.009687 |
| EncodeUwDnaseSknmcPkRep1 | 0.009602 |
| EncodeOpenChromFaireMcf7HypoxlacPk | 0.009601 |
| EncodeADnaseUwSknmcUniPk | 0.009589 |
| EncodeHaibTfbsSknshraCtcfV0416102PkRep1 | 0.009556 |
| EncodeHaibTfbsMcf7SrfV0422111PkRep2 | 0.009531 |
| EncodeOpenChromChipGm13977CtcfPkRep1 | 0.009525 |
| EncodeOpenChromChipSpleenocCtcfPkRep1 | 0.009519 |
| EncodeHaibTfbsK562Bclaf101388Pcr1xPkRep2 | 0.009515 |
| EncodeHaibTfbsGm12878Zbtb33Pcr1xPkRep1 | 0.009506 |
| EncodeHaibTfbsGm12878Mef2csc13268V0416101PkRep2 | 0.009501 |
| EncodeHaibTfbsA549Pbx3V0422111PkRep2 | 0.009493 |
| EncodeSydhTfbsHuvecPol2StdPk | 0.009485 |
| EncodeHaibTfbsK562E2f6sc22823V0416102PkRep1 | 0.009482 |
| EncodeHaibTfbsK562E2f6V0416102PkRep1 | 0.009482 |
| EncodeHaibTfbsSknshNficsc81335V0422111PkRep1 | 0.009482 |
| EncodeUwDnaseA549PkRep2 | 0.009449 |
| EncodeHaibTfbsPfsk1NrsfV0416101PkRep1 | 0.009447 |
| EncodeSydhTfbsHelas3MaxStdPk | 0.009438 |
| EncodeBroadHistoneH1hescChd1a301218aStdPk | 0.009415 |
| EncodeBroadHistoneHelas3H4k20me1StdPk | 0.009410 |
| EncodeSydhTfbsHuvecGata2UcdPk | 0.009409 |
| EncodeSydhHistoneNt2d1H3k36me3bUcdPk | 0.009406 |
| EncodeUwTfbsGm12875CtcfStdPkRep2 | 0.009401 |
| EncodeHaibTfbsH1hescUsf1Pcr1xPkRep1 | 0.009371 |
| EncodeHaibTfbsSknshraRad21V0416102PkRep2 | 0.009368 |
| EncodeHaibTfbsHct116Elf1V0422111PkRep1 | 0.009359 |
| EncodeUwDnaseHmvecdlyadPkRep1 | 0.009357 |
| EncodeUwDnaseHffPkRep2 | 0.009342 |
| EncodeSydhTfbsK562CmycStdPk | 0.009335 |
| EncodeOpenChromChipK562CtcfPk | 0.009326 |
| EncodeHaibTfbsHepg2RxraPcr1xPkRep2 | 0.009322 |
| EncodeADnaseUwHeepicUniPk | 0.009316 |
| EncodeUwDnaseHeePkRep1 | 0.009312 |
| EncodeUwDnaseAg10803PkRep1 | 0.009303 |
| EncodeADnaseUwHmvecdlyadUniPk | 0.009300 |
| EncodeHaibTfbsGm12878SrfV0416101PkRep1 | 0.009279 |
| EncodeADnaseUwAg10803UniPk | 0.009271 |
| EncodeADnaseUwdukeH1hescUniPk | 0.009254 |
| EncodeSydhTfbsH1hescNrf1IggrabPk | 0.009254 |
| EncodeUwDnaseHahPkRep2 | 0.009242 |
| EncodeOpenChromChipH1hescCtcfPk | 0.009196 |
| EncodeHaibTfbsPfsk1NrsfV0416101PkRep2 | 0.009177 |
| EncodeUwDnaseGm04503PkRep2 | 0.009175 |
| EncodeHaibTfbsHelas3GabpPcr1xPkRep1 | 0.009164 |
| EncodeUwTfbsAg04449CtcfStdPkRep1 | 0.009159 |
| EncodeSydhTfbsK562Tf3c110StdPk | 0.009154 |
| EncodeUwTfbsGm12869CtcfStdPkRep1 | 0.009154 |
| EncodeUwTfbsGm12865CtcfStdPkRep2 | 0.009153 |
| EncodeHaibTfbsSknshP300V0422111PkRep1 | 0.009139 |
| EncodeOpenChromChipNhekCtcfPk | 0.009099 |
| EncodeSydhTfbsGm12878Ikzf1iknuclaStdPk | 0.009093 |
| EncodeHaibTfbsPanc1NrsfV0416101PkRep2 | 0.009086 |
| EncodeSydhTfbsHelas3Baf155IggmusPk | 0.009085 |
| EncodeBroadHistoneNhekEzh239875Pk | 0.009083 |
| EncodeUwTfbsGm12869CtcfStdPkRep2 | 0.009055 |
| EncodeUwTfbsGm12867CtcfStdPkRep1 | 0.009031 |
| EncodeUwDnaseMcf7PkRep2 | 0.009018 |
| EncodeUwDnaseHrpePkRep2 | 0.009017 |
| EncodeUwTfbsHuvecCtcfStdPkRep1 | 0.008991 |
| EncodeUwTfbsNhekCtcfStdPkRep1 | 0.008989 |
| EncodeHaibTfbsA549Elf1V0422111Etoh02PkRep1 | 0.008988 |
| EncodeUwTfbsGm12866CtcfStdPkRep1 | 0.008975 |
| EncodeUwDnaseK562Znf4c50c4PkRep2 | 0.008963 |
| EncodeUwDnaseHmvecdadPkRep2 | 0.008955 |
| EncodeSydhTfbsSknshJundIggrabPk | 0.008950 |
| EncodeUwDnaseHsmmPkRep2 | 0.008949 |
| EncodeUwTfbsGm06990CtcfStdPkRep1 | 0.008948 |
| EncodeUwDnasePanc1PkRep2 | 0.008945 |
| EncodeSydhTfbsHelas3Rad21IggrabPk | 0.008927 |
| EncodeHaibTfbsA549Zbtb33V0422111Etoh02PkRep2 | 0.008923 |
| EncodeUwTfbsGm12872CtcfStdPkRep1 | 0.008908 |
| EncodeHaibTfbsHepg2Cebpbsc150V0416101PkRep2 | 0.008894 |
| EncodeUwDnaseHffmycPkRep2 | 0.008876 |
| EncodeHaibTfbsH1hescSp4v20V0422111PkRep1 | 0.008856 |
| EncodeUwDnaseMcf7Estctrl0hPkRep1 | 0.008853 |
| EncodeOpenChromChipMcf7CtcfEstroPkRep1 | 0.008832 |
| EncodeUwDnaseAg10803PkRep2 | 0.008791 |
| EncodeUwTfbsGm12873CtcfStdPkRep2 | 0.008765 |
| EncodeUwTfbsGm12873CtcfStdPkRep1 | 0.008757 |
| EncodeUwDnaseHsmmPkRep1 | 0.008744 |
| EncodeUwTfbsGm12875CtcfStdPkRep1 | 0.008743 |
| EncodeUwTfbsGm12868CtcfStdPkRep1 | 0.008733 |
| EncodeADnaseUwdukeNhekUniPk | 0.008723 |
| EncodeHaibTfbsK562Zbtb33Pcr1xPkRep1 | 0.008722 |
| EncodeOpenChromChipGm20000CtcfPkRep1 | 0.008714 |
| EncodeUwTfbsGm12864CtcfStdPkRep1 | 0.008694 |
| EncodeUwTfbsGm12870CtcfStdPkRep1 | 0.008686 |
| EncodeUwTfbsHepg2CtcfStdPkRep1 | 0.008676 |
| EncodeOpenChromChipLncapCtcfPkRep1 | 0.008675 |
| EncodeOpenChromFaireRcc7860Pk | 0.008638 |
| EncodeHaibTfbsA549GrPcr1xDex50nmPkRep1 | 0.008630 |
| EncodeBroadHistoneDnd41H3k27me3Pk | 0.008622 |
| EncodeHaibTfbsGm12878Rad21V0416101PkRep2 | 0.008622 |
| EncodeBroadHistoneK562H3k27me3StdPk | 0.008609 |
| EncodeUwTfbsHbmecCtcfStdPkRep1 | 0.008606 |
| EncodeUwTfbsHcpeCtcfStdPkRep1 | 0.008598 |
| EncodeUwDnaseAg04450PkRep2 | 0.008597 |
| EncodeSydhTfbsHepg2Pol2PravastStdPk | 0.008588 |
| EncodeHaibTfbsHepg2Ctcfsc5916V0416101PkRep2 | 0.008587 |
| EncodeUwDnaseH7esDiffa14dPkRep2 | 0.008573 |
| EncodeOpenChromChipKidneyocCtcfPkRep1 | 0.008557 |
| EncodeUwTfbsGm12864CtcfStdPkRep2 | 0.008555 |
| EncodeSydhTfbsSknshSmc3IggrabPk | 0.008545 |
| EncodeBroadHistoneH1hescH3k36me3StdPk | 0.008535 |
| EncodeSydhTfbsK562Pol2IggmusPk | 0.008526 |
| EncodeHaibTfbsH1hescEgr1V0416102PkRep2 | 0.008509 |
| EncodeUwTfbsHmecCtcfStdPkRep1 | 0.008504 |
| EncodeHaibTfbsHepg2P300V0416101PkRep2 | 0.008503 |
| EncodeHaibTfbsHct116CtcfcV0422111PkRep2 | 0.008496 |
| EncodeUwDnaseHeePkRep2 | 0.008485 |
| EncodeUwTfbsSknshraCtcfStdPkRep2 | 0.008470 |
| EncodeHaibTfbsHepg2Rad21V0416101PkRep2 | 0.008469 |
| EncodeUwDnaseHaspPkRep1 | 0.008445 |
| EncodeUwTfbsGm12872CtcfStdPkRep2 | 0.008432 |
| EncodeUwDnaseHct116PkRep2 | 0.008423 |
| EncodeADnaseUwHaspUniPk | 0.008417 |
| EncodeUwTfbsSknshraCtcfStdPkRep1 | 0.008417 |
| EncodeSydhTfbsGm12878Stat3IggmusPk | 0.008396 |
| EncodeHaibTfbsSknshNficsc81335V0422111PkRep2 | 0.008386 |
| EncodeUwTfbsBjCtcfStdPkRep1 | 0.008369 |
| EncodeBroadHistoneK562Cbpsc369Pk | 0.008355 |
| EncodeUwTfbsGm06990CtcfStdPkRep2 | 0.008343 |
| EncodeUwTfbsGm12867CtcfStdPkRep2 | 0.008327 |
| EncodeSydhTfbsHelas3CmycStdPk | 0.008319 |
| EncodeHaibTfbsGm12878Mef2csc13268V0416101PkRep1 | 0.008314 |
| EncodeUwTfbsSaecCtcfStdPkRep1 | 0.008290 |
| EncodeUwDnaseHrpePkRep2V2 | 0.008280 |
| EncodeUwTfbsGm12871CtcfStdPkRep1 | 0.008279 |
| EncodeUwDnaseMcf7Est100nm1hPkRep1 | 0.008266 |
| EncodeUwTfbsGm12878CtcfStdPkRep2 | 0.008265 |
| EncodeSydhTfbsHepg2CebpbForsklnStdPk | 0.008264 |
| EncodeUwTfbsGm12866CtcfStdPkRep2 | 0.008260 |
| EncodeADnaseUwdukeHsmmtubeUniPk | 0.008236 |
| EncodeHaibTfbsH1hescTaf7sc101167V0416102PkRep1 | 0.008220 |
| EncodeHaibTfbsK562Rad21V0416102PkRep1 | 0.008208 |
| EncodeADnaseDukeChorionUniPk | 0.008202 |
| EncodeUwTfbsBjCtcfStdPkRep2 | 0.008200 |
| EncodeUwTfbsCaco2CtcfStdPkRep1 | 0.008195 |
| EncodeUwTfbsGm12878CtcfStdPkRep1 | 0.008194 |
| EncodeHaibTfbsSknshZbtb33V0422111PkRep1 | 0.008187 |
| EncodeBroadHistoneNhdfadH4k20me1Pk | 0.008186 |
| EncodeUwTfbsHelas3CtcfStdPkRep2 | 0.008182 |
| EncodeUwTfbsGm12874CtcfStdPkRep2 | 0.008174 |
| EncodeUwDnaseAg09319PkRep1 | 0.008170 |
| EncodeUwTfbsHcmCtcfStdPkRep2 | 0.008165 |
| EncodeUwTfbsCaco2CtcfStdPkRep2 | 0.008159 |
| EncodeSydhTfbsHelas3NfyaIggrabPk | 0.008140 |
| EncodeUwTfbsHacCtcfStdPkRep2 | 0.008133 |
| EncodeHaibTfbsHct116Yy1sc281V0416101PkRep1 | 0.008117 |
| EncodeHaibTfbsA549Ctcfsc5916Pcr1xDex100nmPkRep2 | 0.008079 |
| EncodeSydhTfbsK562Irf1Ifna6hStdPk | 0.008069 |
| EncodeHaibTfbsMcf7Rad21V0422111PkRep1 | 0.008064 |
| EncodeUwTfbsHreCtcfStdPkRep1 | 0.008061 |
| EncodeHaibTfbsA549Ctcfsc5916Pcr1xEtoh02PkRep2 | 0.008057 |
| EncodeHaibTfbsSknshTead4sc101184V0422111PkRep1 | 0.008048 |
| EncodeUwTfbsAg04449CtcfStdPkRep2 | 0.008008 |
| EncodeUwTfbsSaecCtcfStdPkRep2 | 0.007986 |
| EncodeUwTfbsHcfaaCtcfStdPkRep1 | 0.007963 |
| EncodeUwTfbsHuvecCtcfStdPkRep2 | 0.007962 |
| EncodeUwTfbsWerirb1CtcfStdPkRep1 | 0.007952 |
| EncodeUwTfbsAoafCtcfStdPkRep1 | 0.007934 |
| EncodeUwTfbsHelas3CtcfStdPkRep1 | 0.007933 |
| EncodeSydhTfbsHelas3Ap2gammaStdPk | 0.007892 |
| EncodeHaibTfbsK562Creb1sc240V0422111PkRep2 | 0.007881 |
| EncodeHaibTfbsK562Zbtb33Pcr1xPkRep2 | 0.007880 |
| EncodeBroadHistoneHuvecEzh239875Pk | 0.007872 |
| EncodeSydhTfbsHelas3Tr4StdPk | 0.007864 |
| EncodeADnaseUwAg09309UniPk | 0.007860 |
| EncodeUwDnaseAg09309PkRep1 | 0.007855 |
| EncodeHaibTfbsK562Usf1V0416101PkRep2 | 0.007833 |
| EncodeSydhTfbsHepg2Usf2IggrabPk | 0.007828 |
| EncodeBroadHistoneHmecCtcfStdPk | 0.007827 |
| EncodeUwTfbsAg09319CtcfStdPkRep1 | 0.007819 |
| EncodeHaibTfbsH1hescRad21V0416102PkRep1 | 0.007807 |
| EncodeUwTfbsHreCtcfStdPkRep2 | 0.007790 |
| EncodeOpenChromChipHelas3CtcfPk | 0.007782 |
| EncodeBroadHistoneK562NcorPk | 0.007778 |
| EncodeHaibTfbsA549E2f6V0422111PkRep2 | 0.007758 |
| EncodeUwTfbsAg09309CtcfStdPkRep1 | 0.007756 |
| EncodeHaibTfbsH1hescHdac2sc6296V0416102PkRep2 | 0.007745 |
| EncodeHaibTfbsMcf7Gata3V0422111PkRep1 | 0.007712 |
| EncodeHaibTfbsMcf7Fosl2V0422111PkRep1 | 0.007709 |
| EncodeUwTfbsHbmecCtcfStdPkRep2 | 0.007709 |
| EncodeUwTfbsHeeCtcfStdPkRep1 | 0.007668 |
| EncodeUwDnaseHffmycPkRep1 | 0.007652 |
| EncodeUwDnaseH7esDiffa14dPkRep1 | 0.007649 |
| EncodeSydhTfbsHelas3E2f1StdPk | 0.007646 |
| EncodeADnaseUwHffmycUniPk | 0.007640 |
| EncodeHaibTfbsH1hescSin3ak20Pcr1xPkRep2 | 0.007635 |
| EncodeUwDnaseLhcnm2PkRep2 | 0.007626 |
| EncodeUwTfbsWerirb1CtcfStdPkRep2 | 0.007625 |
| EncodeUwTfbsHffmycCtcfStdPkRep1 | 0.007624 |
| EncodeUwTfbsHepg2CtcfStdPkRep2 | 0.007611 |
| EncodeUwTfbsK562CtcfStdPkRep1 | 0.007599 |
| EncodeHaibTfbsSknshRxraV0422111PkRep1 | 0.007591 |
| EncodeUwTfbsA549CtcfStdPkRep2 | 0.007590 |
| EncodeHaibTfbsHct116Elf1V0422111PkRep2 | 0.007559 |
| EncodeHaibTfbsA549Pbx3V0422111PkRep1 | 0.007553 |
| EncodeUwTfbsNhdfneoCtcfStdPkRep1 | 0.007542 |
| EncodeUwTfbsHct116CtcfStdPkRep1 | 0.007534 |
| EncodeBroadHistoneNhekH3k27me3StdPk | 0.007528 |
| EncodeUwTfbsHaspCtcfStdPkRep1 | 0.007527 |
| EncodeSydhTfbsGm12878Rad21IggrabPk | 0.007525 |
| EncodeUwDnaseHrpePkRep1 | 0.007523 |
| EncodeUwTfbsAg09309CtcfStdPkRep2 | 0.007522 |
| EncodeUwTfbsHffCtcfStdPkRep1 | 0.007513 |
| EncodeBroadHistoneHuvecH4k20me1StdPk | 0.007512 |
| EncodeBroadHistoneOsteoH4k20me1Pk | 0.007507 |
| EncodeHaibTfbsMcf7Egr1V0422111PkRep1 | 0.007501 |
| EncodeBroadHistoneOsteoblH3k36me3StdPk | 0.007493 |
| EncodeUwTfbsNb4CtcfStdPkRep1 | 0.007491 |
| EncodeSydhTfbsGm12878Pol2StdPk | 0.007484 |
| EncodeBroadHistoneHuvecH3k9me1StdPk | 0.007468 |
| EncodeUwTfbsHl60CtcfStdPkRep1 | 0.007460 |
| EncodeUwTfbsAg04450CtcfStdPkRep1 | 0.007431 |
| EncodeUwDnaseHct116PkRep1 | 0.007418 |
| EncodeHaibTfbsHct116SrfV0422111PkRep1 | 0.007405 |
| EncodeADnaseUwHct116UniPk | 0.007385 |
| EncodeUwTfbsGm12872CtcfStdPkRep3 | 0.007381 |
| EncodeHaibTfbsHepg2Hnf4asc8987V0416101PkRep2 | 0.007380 |
| EncodeUwTfbsAg04450CtcfStdPkRep2 | 0.007379 |
| EncodeUwTfbsGm12873CtcfStdPkRep3 | 0.007366 |
| EncodeUwDnaseAg09319PkRep2 | 0.007361 |
| EncodeHaibTfbsMcf7Rad21V0422111PkRep2 | 0.007358 |
| EncodeUwTfbsHrpeCtcfStdPkRep2 | 0.007352 |
| EncodeHaibTfbsHepg2Foxa2sc6554V0416101PkRep2 | 0.007351 |
| EncodeHaibTfbsHepg2Zbtb7aV0416101PkRep1 | 0.007346 |
| EncodeOpenChromFaireEndometriumocPk | 0.007338 |
| EncodeUwTfbsHek293CtcfStdPkRep1 | 0.007330 |
| EncodeUwTfbsHaspCtcfStdPkRep2 | 0.007314 |
| EncodeUwTfbsHmecCtcfStdPkRep2 | 0.007287 |
| EncodeHaibTfbsA549Cebpbsc150V0422111PkRep2 | 0.007278 |
| EncodeHaibTfbsHct116Rad21V0422111PkRep1 | 0.007274 |
| EncodeHaibTfbsHepg2Fosl2V0416101PkRep2 | 0.007238 |
| EncodeUwTfbsAoafCtcfStdPkRep2 | 0.007237 |
| EncodeUwDnaseK562Znfp5PkRep2 | 0.007232 |
| EncodeSydhTfbsGm12878Srebp2IggrabPk | 0.007229 |
| EncodeADnaseUwdukeHsmmUniPk | 0.007225 |
| EncodeSydhTfbsGm12878Brca1a300IggmusPk | 0.007211 |
| EncodeUwTfbsNhdfneoCtcfStdPkRep2 | 0.007211 |
| EncodeUwTfbsNhlfCtcfStdPkRep1 | 0.007204 |
| EncodeUwDnaseAg09319PkRep1V2 | 0.007184 |
| EncodeHaibTfbsHepg2Sin3ak20Pcr1xPkRep1 | 0.007182 |
| EncodeSydhTfbsGm12878JundIggrabPk | 0.007180 |
| EncodeADnaseUwAg09319UniPk | 0.007166 |
| EncodeBroadHistoneH1hescPlu1Pk | 0.007166 |
| EncodeUwTfbsHvmfCtcfStdPkRep2 | 0.007149 |
| EncodeHaibTfbsSknshTcf12V0422111PkRep1 | 0.007142 |
| EncodeHaibTfbsK562Cebpbsc150V0422111PkRep2 | 0.007134 |
| EncodeUwTfbsHpfCtcfStdPkRep1 | 0.007128 |
| EncodeUwTfbsHpafCtcfStdPkRep1 | 0.007126 |
| EncodeADnaseUwAg04449UniPk | 0.007125 |
| EncodeSydhHistoneK562bH3k27me3bUcdPk | 0.007125 |
| EncodeSydhHistoneK562H3k27me3bUcdPk | 0.007125 |
| EncodeUwDnaseAg04449PkRep1 | 0.007122 |
| EncodeUwTfbsK562CtcfStdPkRep2 | 0.007116 |
| EncodeUwTfbsAg10803CtcfStdPkRep1 | 0.007105 |
| EncodeBroadHistoneNhdfadEzh239875Pk | 0.007093 |
| EncodeUwDnaseNhekPkRep1 | 0.007090 |
| EncodeUwTfbsGm12868CtcfStdPkRep2 | 0.007089 |
| EncodeHaibTfbsK562Taf7sc101167V0416101PkRep1 | 0.007085 |
| EncodeHaibTfbsGm12878GabpPcr2xPkRep2 | 0.007080 |
| EncodeUwDnaseNt2d1PkRep2 | 0.007068 |
| EncodeUwTfbsAg09319CtcfStdPkRep2 | 0.007064 |
| EncodeSydhTfbsK562Tal1sc12984IggmusPk | 0.007057 |
| EncodeSydhTfbsHelas3Spt20StdPk | 0.007052 |
| EncodeHaibTfbsK562Bclaf101388Pcr1xPkRep1 | 0.007022 |
| EncodeHaibTfbsHct116Rad21V0422111PkRep2 | 0.007019 |
| EncodeHaibTfbsHepg2Hnf4asc8987V0416101PkRep1 | 0.007003 |
| EncodeSydhTfbsHepg2Mafksc477IggrabPk | 0.006988 |
| EncodeHaibTfbsHepg2Foxa1sc6553V0416101PkRep2 | 0.006978 |
| EncodeUwTfbsRptecCtcfStdPkRep1 | 0.006959 |
| EncodeSydhTfbsHepg2Tr4UcdPk | 0.006950 |
| EncodeUwDnaseHrpePkRep1V2 | 0.006944 |
| EncodeSydhTfbsSknshRad21IggrabPk | 0.006934 |
| EncodeHaibTfbsGm12878BatfPcr1xPkRep1 | 0.006928 |
| EncodeUwTfbsHmfCtcfStdPkRep2 | 0.006919 |
| EncodeADnaseUwHrpepicUniPk | 0.006915 |
| EncodeHaibTfbsHct116Egr1V0422111PkRep1 | 0.006888 |
| EncodeUwDnaseK562PkRep2 | 0.006882 |
| EncodeOpenChromChipLncapCtcfAndroPkRep1 | 0.006867 |
| EncodeUwHistoneBe2cH3k04me3StdPkRep2 | 0.006866 |
| EncodeOpenChromChipMedulloCtcfPkRep1 | 0.006860 |
| EncodeUwTfbsHcmCtcfStdPkRep1 | 0.006858 |
| EncodeHaibTfbsMcf7Pmlsc71910V0422111PkRep1 | 0.006856 |
| EncodeBroadHistoneK562Cbx2Pk | 0.006847 |
| EncodeUwTfbsHffmycCtcfStdPkRep2 | 0.006841 |
| EncodeUwTfbsNhekCtcfStdPkRep2 | 0.006829 |
| EncodeSydhTfbsGm12892NfkbTnfaIggrabPk | 0.006826 |
| EncodeHaibTfbsK562SrfV0416101PkRep1 | 0.006804 |
| EncodeSydhTfbsShsy5yGata2UcdPk | 0.006803 |
| EncodeHaibTfbsEcc1Creb1sc240V0422111PkRep1 | 0.006784 |
| EncodeSydhTfbsK562Stat2Ifna30StdPk | 0.006770 |
| EncodeUwTfbsHct116CtcfStdPkRep2 | 0.006740 |
| EncodeUwDnaseAg09309PkRep2 | 0.006734 |
| EncodeBroadHistoneHmecEzh239875Pk | 0.006732 |
| EncodeHaibTfbsK562NrsfV0416102PkRep2 | 0.006730 |
| EncodeUwTfbsGm12870CtcfStdPkRep2 | 0.006724 |
| EncodeUwTfbsHvmfCtcfStdPkRep1 | 0.006705 |
| EncodeSydhTfbsImr90Chd1nb10060411IggrabPk | 0.006698 |
| EncodeHaibTfbsT47dCtcfsc5916V0416102Dm002p1hPkRep1 | 0.006672 |
| EncodeUwTfbsHpfCtcfStdPkRep2 | 0.006653 |
| EncodeUwTfbsHek293CtcfStdPkRep2 | 0.006637 |
| EncodeSydhTfbsK562Atf106325StdPk | 0.006635 |
| EncodeUwTfbsHrpeCtcfStdPkRep1 | 0.006600 |
| EncodeHaibTfbsEcc1Egr1V0422111PkRep1 | 0.006583 |
| EncodeUwTfbsGm12864CtcfStdPkRep3 | 0.006581 |
| EncodeUwTfbsWi38CtcfStdPkRep2 | 0.006569 |
| EncodeSydhTfbsHelas3JundIggrabPk | 0.006567 |
| EncodeHaibTfbsA549Usf1V0422111Etoh02PkRep1 | 0.006555 |
| EncodeHaibTfbsHepg2Zbtb33Pcr1xPkRep2 | 0.006555 |
| EncodeSydhTfbsA549Rad21IggrabPk | 0.006551 |
| EncodeBroadHistoneNhaEzh239875Pk | 0.006536 |
| EncodeUwHistoneBe2cH3k04me3StdPkRep1 | 0.006507 |
| EncodeBroadHistoneHsmmEzh239875Pk | 0.006481 |
| EncodeUwTfbsHpafCtcfStdPkRep2 | 0.006476 |
| EncodeBroadHistoneNhdfadH3k27me3StdPk | 0.006475 |
| EncodeHaibTfbsHepg2Foxa1sc101058V0416101PkRep1 | 0.006472 |
| EncodeSydhTfbsGm12878Cdpsc6327IggmusPk | 0.006436 |
| EncodeHaibTfbsA549Rad21V0422111PkRep2 | 0.006435 |
| EncodeOpenChromChipHepg2CtcfPk | 0.006391 |
| EncodeUwTfbsGm12865CtcfStdPkRep3 | 0.006384 |
| EncodeUwDnaseNhberaPkRep2 | 0.006374 |
| EncodeUwDnaseLhcnm2Diff4dPkRep1 | 0.006363 |
| EncodeHaibTfbsGm12878Atf3Pcr1xPkRep2 | 0.006325 |
| EncodeHaibTfbsHepg2P300V0416101PkRep1 | 0.006311 |
| EncodeOpenChromFaireH1hescPk | 0.006306 |
| EncodeHaibTfbsSknshYy1sc281V0422111PkRep1 | 0.006271 |
| EncodeUwTfbsGm12801CtcfStdPkRep1 | 0.006242 |
| EncodeHaibTfbsK562Six5Pcr1xPkRep2 | 0.006216 |
| EncodeUwDnaseT47dPkRep2 | 0.006215 |
| EncodeHaibTfbsHl60GabpV0422111PkRep1 | 0.006198 |
| EncodeHaibTfbsHepg2Zbtb33V0416101PkRep1 | 0.006147 |
| EncodeHaibTfbsHl60GabpV0422111PkRep2 | 0.006117 |
| EncodeHaibTfbsHepg2Cebpbsc150V0416101PkRep1 | 0.006113 |
| EncodeADnaseUwHnpcepicUniPk | 0.006110 |
| EncodeUwDnaseHnpcePkRep1 | 0.006109 |
| EncodeOpenChromFaireMrta2041Pk | 0.006107 |
| EncodeUwDnaseK562Znfe103c6PkRep2 | 0.006107 |
| EncodeUwDnaseK562Znf4c50c4PkRep1 | 0.006105 |
| EncodeUwTfbsMcf7CtcfStdPkRep1 | 0.006103 |
| EncodeHaibTfbsK562Fosl1sc183V0416101PkRep2 | 0.006090 |
| EncodeHaibTfbsHepg2Zbtb33Pcr1xPkRep1 | 0.006089 |
| EncodeBroadHistoneMonocd14ro1746H3k27me3Pk | 0.006088 |
| EncodeBroadHistoneHmecH3k36me3StdPk | 0.006078 |
| EncodeUwDnaseK562PkRep1 | 0.006078 |
| EncodeUwTfbsHacCtcfStdPkRep1 | 0.006077 |
| EncodeHaibTfbsEcc1CtcfcV0416102Dm002p1hPkRep1 | 0.006076 |
| EncodeUwDnaseWerirb1PkRep2 | 0.006071 |
| EncodeUwDnaseMcf7Estctrl0hPkRep2 | 0.006069 |
| EncodeUwDnaseHelas3PkRep1 | 0.006063 |
| EncodeUwDnaseHelas3PkRep2 | 0.006058 |
| EncodeSydhTfbsH1hescGtf2f1IggrabPk | 0.006030 |
| EncodeADnaseUwdukeHmecUniPk | 0.006025 |
| EncodeSydhTfbsK562CjunIfna30StdPk | 0.006024 |
| EncodeUwTfbsWi38CtcfStdPkRep1 | 0.006000 |
| EncodeUwTfbsHcpeCtcfStdPkRep2 | 0.005999 |
| EncodeADnaseUwBjUniPk | 0.005996 |
| EncodeUwDnaseBjPkRep1 | 0.005995 |
| EncodeUwTfbsRptecCtcfStdPkRep2 | 0.005972 |
| EncodeHaibTfbsT47dFoxa1sc6553V0416102Dm002p1hPkRep1 | 0.005966 |
| EncodeUwDnaseLncapPkRep2 | 0.005959 |
| EncodeHaibTfbsK562Hdac2sc6296V0416102PkRep2 | 0.005931 |
| EncodeBroadHistoneHelas3Ezh239875Pk | 0.005914 |
| EncodeSydhTfbsK562Rfx5IggrabPk | 0.005903 |
| EncodeSydhTfbsGm12878MaxStdPk | 0.005890 |
| EncodeUchicagoTfbsK562Enr4a1ControlPk | 0.005889 |
| EncodeSydhTfbsK562CjunStdPk | 0.005865 |
| EncodeHaibTfbsHct116CtcfcV0422111PkRep1 | 0.005847 |
| EncodeADnaseUwdukeHelas3UniPk | 0.005829 |
| EncodeHaibTfbsHepg2SrfV0416101PkRep2 | 0.005827 |
| EncodeBroadHistoneHuvecH3k27me3StdPk | 0.005823 |
| EncodeHaibTfbsMcf7Nr2f2sc271940V0422111PkRep2 | 0.005820 |
| EncodeHaibTfbsHct116Zbtb33V0416101PkRep2 | 0.005781 |
| EncodeHaibTfbsA549GrPcr2xDex100nmPkRep2 | 0.005778 |
| EncodeSydhTfbsHelas3NfybIggrabPk | 0.005776 |
| EncodeHaibTfbsGm12878Usf1Pcr2xPkRep1 | 0.005775 |
| EncodeHaibTfbsSknshTcf12V0422111PkRep2 | 0.005769 |
| EncodeSydhTfbsK562MaxStdPk | 0.005750 |
| EncodeUwTfbsA549CtcfStdPkRep1 | 0.005729 |
| EncodeHaibTfbsA549Tead4sc101184V0422111PkRep1 | 0.005726 |
| EncodeUwTfbsAg10803CtcfStdPkRep2 | 0.005723 |
| EncodeUwTfbsHmfCtcfStdPkRep1 | 0.005716 |
| EncodeHaibTfbsHepg2Usf1Pcr1xPkRep1 | 0.005707 |
| EncodeHaibTfbsH1hescEgr1V0416102PkRep1 | 0.005701 |
| EncodeHaibTfbsGm12878GabpPcr2xPkRep1 | 0.005697 |
| EncodeBroadHistoneHmecH4k20me1StdPk | 0.005675 |
| EncodeBroadHistoneA549H3k27me3Etoh02Pk | 0.005661 |
| EncodeSydhHistoneU2osH3k36me3bUcdPk | 0.005646 |
| EncodeUwDnaseRptecPkRep2 | 0.005645 |
| EncodeHaibTfbsMcf7Cebpbsc150V0422111PkRep2 | 0.005636 |
| EncodeHaibTfbsK562Ctcflsc98982V0416101PkRep1 | 0.005627 |
| EncodeHaibTfbsHct116Sp1V0422111PkRep2 | 0.005607 |
| EncodeBroadHistoneK562Cbx8Pk | 0.005591 |
| EncodeSydhTfbsK562P300sc584sc48343IggrabPk | 0.005584 |
| EncodeSydhTfbsHepg2Maffm8194IggrabPk | 0.005579 |
| EncodeBroadHistoneHsmmtH3k27me3Pk | 0.005567 |
| EncodeHaibTfbsSknshElf1V0422111PkRep1 | 0.005553 |
| EncodeBroadHistoneA549H3k09me3Etoh02Pk | 0.005542 |
| EncodeHaibTfbsMcf7Gata3V0422111PkRep2 | 0.005528 |
| EncodeBroadHistoneGm12878H3k27me3StdPkV2 | 0.005522 |
| EncodeUwDnaseMcf7Est100nm1hPkRep2 | 0.005508 |
| EncodeHaibTfbsGm12878Rad21V0416101PkRep1 | 0.005491 |
| EncodeBroadHistoneNhlfH3k27me3StdPk | 0.005488 |
| EncodeUwDnaseHmecPkRep1 | 0.005487 |
| EncodeUwTfbsMcf7CtcfStdPkRep2 | 0.005479 |
| EncodeUwDnaseHnpcePkRep2 | 0.005472 |
| EncodeHaibTfbsHelas3NrsfPcr1xPkRep2 | 0.005459 |
| EncodeHaibTfbsT47dGata3sc268V0416102Dm002p1hPkRep1 | 0.005434 |
| EncodeHaibTfbsGm12878RxraPcr1xPkRep1 | 0.005433 |
| EncodeHaibTfbsHepg2Tcf12Pcr1xPkRep1 | 0.005402 |
| EncodeSydhTfbsHuvecMaxStdPk | 0.005390 |
| EncodeHaibTfbsK562Sin3ak20V0416101PkRep1 | 0.005380 |
| EncodeUwDnaseHnpcePkRep2V2 | 0.005378 |
| EncodeSydhTfbsMcf7Znf217UcdPk | 0.005367 |
| EncodeHaibTfbsEcc1Cebpbsc150V0422111PkRep2 | 0.005361 |
| EncodeUwDnaseAg04449PkRep2 | 0.005359 |
| EncodeSydhTfbsK562Stat1Ifng6hStdPk | 0.005338 |
| EncodeHaibTfbsPanc1NrsfPcr2xPkRep1 | 0.005336 |
| EncodeSydhTfbsK562Bach1sc14700IggrabPk | 0.005331 |
| EncodeBroadHistoneNhaH3k27me3StdPk | 0.005328 |
| EncodeUwDnaseNhekPkRep2 | 0.005316 |
| EncodeSydhTfbsHepg2Mafkab50322IggrabPk | 0.005314 |
| EncodeUwDnaseK562Znfb34a8PkRep2 | 0.005301 |
| EncodeHaibTfbsH1hescAtf3V0416102PkRep1 | 0.005300 |
| EncodeSydhTfbsSknshNrf1IggrabPk | 0.005290 |
| EncodeSydhTfbsHelas3Brg1IggmusPk | 0.005269 |
| EncodeSydhTfbsK562Ini1IggmusPk | 0.005257 |
| EncodeSydhTfbsH1hescCjunIggrabPk | 0.005255 |
| EncodeBroadHistoneH1hescH3k27me3StdPk | 0.005244 |
| EncodeSydhTfbsK562MaffIggrabPk | 0.005221 |
| EncodeHaibTfbsA549Taf1V0422111Etoh02PkRep2 | 0.005218 |
| EncodeHaibTfbsA549GrPcr1xDex5nmPkRep2 | 0.005217 |
| EncodeHaibTfbsSknshElf1V0422111PkRep2 | 0.005210 |
| EncodeSydhTfbsK562Rpc155StdPk | 0.005179 |
| EncodeOpenChromChipHuvecCtcfPk | 0.005151 |
| EncodeHaibTfbsHepg2Zbtb33V0416101PkRep2 | 0.005144 |
| EncodeUwDnaseK562Znfg54a11PkRep1 | 0.005144 |
| EncodeUwTfbsHeeCtcfStdPkRep2 | 0.005117 |
| EncodeHaibMethyl450LncapSitesRep1 | 0.005106 |
| EncodeHaibTfbsHepg2Foxa1sc6553V0416101PkRep1 | 0.005102 |
| EncodeHaibTfbsH1hescNanogsc33759V0416102PkRep2 | 0.005091 |
| EncodeOpenChromFaireMrtttc549Pk | 0.005083 |
| EncodeSydhTfbsK562Stat1Ifng30StdPk | 0.005082 |
| EncodeUwDnaseMcf7PkRep1 | 0.005079 |
| EncodeSydhHistoneMcf7H3k36me3bUcdPk | 0.005062 |
| EncodeHaibTfbsHct116Atf3V0422111PkRep1 | 0.005020 |
| EncodeHaibTfbsGm12878RxraPcr1xPkRep2 | 0.004997 |
| EncodeSydhTfbsK562Tr4UcdPk | 0.004987 |
| EncodeHaibMethyl450Nt2d1SitesRep1 | 0.004979 |
| EncodeHaibTfbsH1neuronsNrsfV0422111PkRep2 | 0.004938 |
| EncodeHaibTfbsH1hescNrsfV0416102PkRep2 | 0.004919 |
| EncodeSydhTfbsGm12878CfosStdPk | 0.004907 |
| EncodeBroadHistoneHsmmH3k27me3StdPk | 0.004899 |
| EncodeHaibTfbsHepg2Zeb1V0422111PkRep1 | 0.004879 |
| EncodeSydhTfbsHek293Tcf7l2UcdPk | 0.004877 |
| EncodeHaibMethyl450Caco2SitesRep1 | 0.004876 |
| EncodeHaibMethyl450SknshraSitesRep1 | 0.004864 |
| EncodeHaibTfbsHepg2Atf3V0416101PkRep1 | 0.004853 |
| EncodeBroadHistoneA549H3k27me3Dex100nmPk | 0.004844 |
| EncodeHaibMethyl450Ecc1SitesRep1 | 0.004841 |
| EncodeOpenChromFaireNhbePk | 0.004823 |
| EncodeADnaseUwdukeMcf7UniPk | 0.004815 |
| EncodeHaibMethyl450SknmcSitesRep1 | 0.004806 |
| EncodeSydhTfbsImr90MafkIggrabPk | 0.004792 |
| EncodeHaibTfbsT47dP300V0416102Dm002p1hPkRep1 | 0.004784 |
| EncodeHaibMethyl450SknshSitesRep1 | 0.004777 |
| EncodeHaibMethyl450Helas3SitesRep1 | 0.004753 |
| EncodeSydhTfbsK562Yy1UcdPk | 0.004745 |
| EncodeADnaseUwdukeK562UniPk | 0.004736 |
| EncodeHaibMethyl450H1hescSitesRep1 | 0.004733 |
| EncodeHaibMethyl450Hek293SitesRep1 | 0.004731 |
| EncodeHaibTfbsSknshUsf1V0422111PkRep1 | 0.004725 |
| EncodeHaibTfbsK562Bcl3Pcr1xPkRep1 | 0.004716 |
| EncodeUwDnaseHmecPkRep2 | 0.004694 |
| EncodeHaibMethyl450HepatoSitesRep1 | 0.004687 |
| EncodeHaibMethyl450T47dDm002p24hSitesRep1 | 0.004679 |
| EncodeUwDnaseNhberaPkRep1 | 0.004677 |
| EncodeHaibMethyl450A549Etoh02SitesRep1 | 0.004675 |
| EncodeHaibTfbsEcc1P300V0422111PkRep2 | 0.004672 |
| EncodeSydhTfbsH1hescMafkIggrabPk | 0.004665 |
| EncodeSydhTfbsK562Nrf1IggrabPk | 0.004649 |
| EncodeHaibTfbsEcc1Rad21V0422111PkRep2 | 0.004646 |
| EncodeHaibMethyl450Hct116StanfordSitesRep1 | 0.004644 |
| EncodeSydhHistoneNt2d1H3k27me3bUcdPk | 0.004612 |
| EncodeHaibTfbsHct116Zbtb33V0416101PkRep1 | 0.004595 |
| EncodeHaibMethyl450Hct116HaibSitesRep1 | 0.004593 |
| EncodeOpenChromFairePanisletsPk | 0.004589 |
| EncodeBroadHistoneGm12878H3k27me3StdPk | 0.004576 |
| EncodeHaibMethyl450Ovcar3SitesRep1 | 0.004574 |
| EncodeHaibMethyl450Mcf7SitesRep1 | 0.004572 |
| EncodeADnaseUwRptecUniPk | 0.004567 |
| EncodeUwDnaseRptecPkRep1 | 0.004567 |
| EncodeHaibMethyl450AosmcSitesRep1 | 0.004545 |
| EncodeHaibTfbsK562Yy1V0416102PkRep1 | 0.004529 |
| EncodeUwDnaseK562Znf4g7d3PkRep2 | 0.004525 |
| EncodeHaibTfbsHct116Usf1V0422111PkRep2 | 0.004519 |
| EncodeSydhTfbsHelas3Ap2alphaStdPk | 0.004512 |
| EncodeHaibTfbsEcc1EralphaaV0416102Est10nm1hPkRep1 | 0.004500 |
| EncodeHaibMethyl450Panc1SitesRep1 | 0.004493 |
| EncodeHaibTfbsHepg2RxraPcr1xPkRep1 | 0.004482 |
| EncodeSydhTfbsHelas3Nrf1IggmusPk | 0.004482 |
| EncodeHaibMethyl450Pfsk1SitesRep1 | 0.004474 |
| EncodeHaibMethyl450Ag09309SitesRep1 | 0.004468 |
| EncodeBroadHistoneNhlfH4k20me1StdPk | 0.004445 |
| EncodeHaibMethyl450Imr90SitesRep1 | 0.004415 |
| EncodeHaibMethyl450Be2cSitesRep1 | 0.004407 |
| EncodeSydhTfbsK562Brf1StdPk | 0.004397 |
| EncodeUwDnaseA549PkRep1 | 0.004392 |
| EncodeSydhTfbsMcf7Gata3sc269UcdPk | 0.004380 |
| EncodeHaibTfbsK562Six5V0416101PkRep1 | 0.004377 |
| EncodeHaibMethyl450Hepg2SitesRep1 | 0.004362 |
| EncodeHaibMethyl450U87SitesRep1 | 0.004354 |
| EncodeHaibMethyl450Ag04450SitesRep1 | 0.004353 |
| EncodeHaibMethyl450Ag09319SitesRep1 | 0.004347 |
| EncodeHaibMethyl450NhdfneoSitesRep1 | 0.004340 |
| EncodeHaibMethyl450BjSitesRep1 | 0.004321 |
| EncodeSydhTfbsK562CjunIfna6hStdPk | 0.004318 |
| EncodeHaibTfbsEcc1CtcfcV0416102Dm002p1hPkRep2 | 0.004317 |
| EncodeHaibTfbsSknshJundV0422111PkRep1 | 0.004315 |
| EncodeHaibTfbsH1hescTcf12Pcr1xPkRep1 | 0.004314 |
| EncodeHaibMethyl450HrpeSitesRep1 | 0.004299 |
| EncodeUwDnaseK562Znfg54a11PkRep2 | 0.004285 |
| EncodeHaibTfbsEcc1EralphaaV0416102Gen1hPkRep2 | 0.004279 |
| EncodeHaibTfbsGm12878Atf3Pcr1xPkRep1 | 0.004276 |
| EncodeHaibMethyl450Ag04449SitesRep1 | 0.004272 |
| EncodeHaibMethyl450ProgfibSitesRep1 | 0.004267 |
| EncodeHaibTfbsH1hescP300V0416102PkRep1 | 0.004262 |
| EncodeHaibMethyl450JurkatSitesRep1 | 0.004249 |
| EncodeHaibMethyl450HrceSitesRep1 | 0.004248 |
| EncodeHaibMethyl450K562SitesRep1 | 0.004240 |
| EncodeHaibMethyl450RptecSitesRep1 | 0.004227 |
| EncodeSydhTfbsNt2d1Yy1UcdPk | 0.004225 |
| EncodeHaibTfbsSknshTead4sc101184V0422111PkRep2 | 0.004222 |
| EncodeBroadHistoneHsmmtEzh239875Pk | 0.004217 |
| EncodeSydhTfbsMcf7Tcf7l2UcdPk | 0.004211 |
| EncodeHaibMethyl450Mcf10aesSitesRep1 | 0.004193 |
| EncodeHaibMethyl450Mcf10aesTamSitesRep1 | 0.004187 |
| EncodeHaibMethyl450HreSitesRep1 | 0.004177 |
| EncodeHaibTfbsK562Mef2aV0416101PkRep2 | 0.004177 |
| EncodeUwDnaseK562Znfa41c6PkRep1 | 0.004174 |
| EncodeHaibMethyl450PrecSitesRep1 | 0.004158 |
| EncodeSydhTfbsH1hescRfx5200401194IggrabPk | 0.004158 |
| EncodeHaibMethyl450NhaSitesRep1 | 0.004154 |
| EncodeHaibMethyl450HmecSitesRep1 | 0.004152 |
| EncodeSydhTfbsHelas3Hae2f1StdPk | 0.004149 |
| EncodeHaibTfbsSknshGata3V0422111PkRep2 | 0.004147 |
| EncodeOpenChromFaireMcf7VehPk | 0.004144 |
| EncodeSydhTfbsGm12878ErraIggrabPk | 0.004144 |
| EncodeHaibMethyl450HpaeSitesRep1 | 0.004136 |
| EncodeHaibTfbsGm12878P300Pcr1xPkRep2 | 0.004127 |
| EncodeHaibMethyl450Ag10803SitesRep1 | 0.004124 |
| EncodeHaibMethyl450SaecSitesRep1 | 0.004121 |
| EncodeHaibMethyl450HcmSitesRep1 | 0.004115 |
| EncodeHaibMethyl450HcfSitesRep1 | 0.004113 |
| EncodeHaibMethyl450HuvecSitesRep1 | 0.004109 |
| EncodeSydhTfbsSknshP300bIggrabPk | 0.004104 |
| EncodeHaibTfbsK562Bcl3Pcr1xPkRep2 | 0.004093 |
| EncodeSydhTfbsHelas3P300sc584sc584IggrabPk | 0.004090 |
| EncodeHaibMethyl450SkmcSitesRep1 | 0.004088 |
| EncodeHaibMethyl450HipeSitesRep1 | 0.004084 |
| EncodeHaibMethyl450HaeSitesRep1 | 0.004064 |
| EncodeHaibMethyl450NhbeSitesRep1 | 0.004062 |
| EncodeHaibTfbsHelas3NrsfPcr1xPkRep1 | 0.004057 |
| EncodeHaibTfbsHct116JundV0422111PkRep1 | 0.004051 |
| EncodeHaibTfbsA549GrPcr1xDex500pmPkRep1 | 0.004044 |
| EncodeUwHistoneGm06990H3k36me3StdPkRep1 | 0.004036 |
| EncodeSydhTfbsImr90Rfx5IggrabPk | 0.004022 |
| EncodeHaibTfbsGm12878Usf1Pcr2xPkRep2 | 0.004019 |
| EncodeSydhTfbsGm12878Gcn5StdPk | 0.004017 |
| EncodeHaibMethyl450HnpceSitesRep1 | 0.004014 |
| EncodeHaibTfbsSknshGata3V0422111PkRep1 | 0.004014 |
| EncodeUwDnaseK562Znff41b2PkRep2 | 0.003963 |
| EncodeOpenChromFaireA549Pk | 0.003962 |
| EncodeHaibTfbsH1hescNrsfV0416102PkRep1 | 0.003939 |
| EncodeHaibTfbsH1hescFosl1sc183V0416102PkRep1 | 0.003938 |
| EncodeHaibTfbsSknshRxraV0422111PkRep2 | 0.003933 |
| EncodeSydhTfbsHelas3Stat3IggrabPk | 0.003928 |
| EncodeHaibMethyl450HcpeSitesRep1 | 0.003921 |
| EncodeOpenChromFaireMcf7Est10nm30mPk | 0.003904 |
| EncodeBroadHistoneK562RestPk | 0.003898 |
| EncodeHaibMethyl450HeeSitesRep1 | 0.003884 |
| EncodeSydhTfbsHepg2CjunIggrabPk | 0.003868 |
| EncodeSydhTfbsU2osSetdb1UcdPk | 0.003853 |
| EncodeADnaseUwBe2cUniPk | 0.003823 |
| EncodeUwDnaseBe2cPkRep1 | 0.003806 |
| EncodeBroadHistoneK562H3k9me3StdPk | 0.003801 |
| EncodeHaibTfbsEcc1Egr1V0422111PkRep2 | 0.003798 |
| EncodeUwTfbsBe2cCtcfStdPkRep2 | 0.003795 |
| EncodeHaibMethyl450Gm06990SitesRep1 | 0.003792 |
| EncodeUwHistoneGm12878H3k36me3StdPkRep1 | 0.003785 |
| EncodeOpenChromFaireK562NabutPk | 0.003774 |
| EncodeHaibTfbsK562Six5Pcr1xPkRep1 | 0.003768 |
| EncodeSydhTfbsHuvecCjunStdPk | 0.003768 |
| EncodeUwHistoneGm12878H3k36me3StdPkRep2 | 0.003759 |
| EncodeHaibTfbsGm12878Zeb1sc25388V0416102PkRep1 | 0.003757 |
| EncodeHaibTfbsHct116Cbx3sc101004V0422111PkRep1 | 0.003728 |
| EncodeSydhTfbsK562Bdp1StdPk | 0.003727 |
| EncodeSydhTfbsK562Usf2IggrabPk | 0.003721 |
| EncodeHaibTfbsGm12878Ets1Pcr1xPkRep1V2 | 0.003715 |
| EncodeHaibTfbsK562Taf7sc101167V0416101PkRep2 | 0.003715 |
| EncodeHaibTfbsEcc1EralphaaV0416102Est10nm1hPkRep2 | 0.003692 |
| EncodeHaibTfbsHepg2Sp2V0422111PkRep2 | 0.003685 |
| EncodeBroadHistoneCd20ro01794Ezh239875Pk | 0.003673 |
| EncodeSydhTfbsHepg2Hnf4aForsklnStdPk | 0.003672 |
| EncodeSydhTfbsA549Bhlhe40IggrabPk | 0.003644 |
| EncodeHaibTfbsGm12878SrfPcr2xPkRep1 | 0.003643 |
| EncodeSydhTfbsGm12878E2f4IggmusPk | 0.003642 |
| EncodeUwHistoneBjH3k36me3StdPkRep1 | 0.003633 |
| EncodeHaibMethyl450CmkSitesRep1 | 0.003625 |
| EncodeUwDnaseLncapPkRep1 | 0.003592 |
| EncodeHaibMethyl450Nb4SitesRep1 | 0.003587 |
| EncodeOpenChromFaireNhaPk | 0.003586 |
| EncodeOpenChromFaireK562OhureaPk | 0.003585 |
| EncodeBroadHistoneOsteoH3k27me3Pk | 0.003575 |
| EncodeHaibTfbsH1hescSix5Pcr1xPkRep2 | 0.003559 |
| EncodeHaibTfbsK562Sp1Pcr1xPkRep2 | 0.003553 |
| EncodeHaibMethyl450Gm12892SitesRep1 | 0.003531 |
| EncodeHaibTfbsSknshMef2aV0422111PkRep2 | 0.003527 |
| EncodeSydhTfbsK562Stat1Ifna6hStdPk | 0.003519 |
| EncodeUwDnaseK562Znfe103c6PkRep1 | 0.003503 |
| EncodeHaibMethyl450Gm12878SitesRep1 | 0.003464 |
| EncodeUwHistoneHuvecH3k36me3StdPkRep2 | 0.003462 |
| EncodeSydhTfbsGm12878NfkbTnfaIggrabPk | 0.003444 |
| EncodeHaibTfbsMcf7JundV0422111PkRep1 | 0.003425 |
| EncodeADnaseDukeHepatocytesUniPk | 0.003420 |
| EncodeHaibMethyl450Gm19239SitesRep1 | 0.003407 |
| EncodeUwHistoneGm06990H3k36me3StdPkRep2 | 0.003403 |
| EncodeBroadHistoneGm12878H3k9me3StdPk | 0.003396 |
| EncodeSydhTfbsK562Stat1Ifna30StdPk | 0.003393 |
| EncodeUwHistoneHuvecH3k36me3StdPkRep1 | 0.003344 |
| EncodeHaibTfbsA549Ets1V0422111Etoh02PkRep2 | 0.003338 |
| EncodeHaibTfbsSknshNrsfPcr2xPkRep1 | 0.003338 |
| EncodeUwDnaseBe2cPkRep2 | 0.003327 |
| EncodeUwHistoneSaecH3k36me3StdPkRep1 | 0.003319 |
| EncodeSydhTfbsHelas3CjunIggrabPk | 0.003316 |
| EncodeHaibTfbsHepg2Hdac2sc6296V0416101PkRep2 | 0.003273 |
| EncodeHaibTfbsHct116SrfV0422111PkRep2 | 0.003266 |
| EncodeBroadHistoneHelas3H3k27me3StdPk | 0.003260 |
| EncodeHaibTfbsK562Sp2sc643V0416102PkRep2 | 0.003251 |
| EncodeUwHistoneBjH3k36me3StdPkRep2 | 0.003249 |
| EncodeHaibTfbsH1hescSp2V0422111PkRep1 | 0.003248 |
| EncodeHaibTfbsK562Stat5asc74442V0422111PkRep2 | 0.003203 |
| EncodeHaibTfbsHct116Fosl1V0422111PkRep2 | 0.003194 |
| EncodeHaibMethyl450Gm12891SitesRep1 | 0.003192 |
| EncodeHaibTfbsHct116Fosl1V0422111PkRep1 | 0.003180 |
| EncodeHaibTfbsK562Mef2aV0416101PkRep1 | 0.003179 |
| EncodeADnaseUwdukeLncapUniPk | 0.003170 |
| EncodeHaibTfbsGm12878Six5Pcr1xPkRep2 | 0.003160 |
| EncodeUwDnaseK562Znf4g7d3PkRep1 | 0.003156 |
| EncodeUwTfbsBe2cCtcfStdPkRep1 | 0.003154 |
| EncodeBroadHistoneHsmmH3k9me3StdPk | 0.003136 |
| EncodeHaibTfbsGm12878Bcl3V0416101PkRep2 | 0.003131 |
| EncodeHaibTfbsHct116Egr1V0422111PkRep2 | 0.003131 |
| EncodeHaibTfbsT47dEralphaaV0416102Est10nm1hPkRep2 | 0.003120 |
| EncodeHaibMethyl450Hl60SitesRep1 | 0.003095 |
| EncodeSydhTfbsHepg2Irf3IggrabPk | 0.003082 |
| EncodeHaibTfbsH1hescHdac2sc6296V0416102PkRep1 | 0.003063 |
| EncodeSydhTfbsK562NfybStdPk | 0.003058 |
| EncodeHaibTfbsK562Gata2sc267Pcr1xPkRep2 | 0.003056 |
| EncodeHaibTfbsK562Sp2sc643V0416102PkRep1 | 0.003050 |
| EncodeSydhTfbsK562Znf263UcdPk | 0.003047 |
| EncodeHaibTfbsSknshGabpV0422111PkRep1 | 0.003034 |
| EncodeSydhTfbsK562Gata1UcdPk | 0.002987 |
| EncodeHaibTfbsSknshFosl2V0422111PkRep2 | 0.002968 |
| EncodeHaibTfbsHct116Atf3V0422111PkRep2 | 0.002894 |
| EncodeHaibTfbsH1hescSp2V0422111PkRep2 | 0.002863 |
| EncodeHaibTfbsH1hescRxraV0416102PkRep2 | 0.002841 |
| EncodeSydhTfbsGm12878Tr4StdPk | 0.002809 |
| EncodeHaibTfbsH1hescAtf3V0416102PkRep2 | 0.002774 |
| EncodeHaibTfbsHct116Cebpbsc150V0422111PkRep1 | 0.002763 |
| EncodeOpenChromFaireKidneyocPk | 0.002751 |
| EncodeSydhTfbsHepg2Srebp1InslnStdPk | 0.002749 |
| EncodeHaibTfbsGm12878NrsfPcr1xPkRep2 | 0.002745 |
| EncodeHaibTfbsEcc1Foxm1sc502V0422111PkRep2 | 0.002736 |
| EncodeHaibTfbsGm12891Pax5c20V0416101PkRep2 | 0.002705 |
| EncodeSydhTfbsK562NfyaStdPk | 0.002705 |
| EncodeHaibTfbsHepg2SrfV0416101PkRep1 | 0.002670 |
| EncodeHaibTfbsU87NrsfPcr2xPkRep1V2 | 0.002653 |
| EncodeUwDnaseK562Znfa41c6PkRep2 | 0.002652 |
| EncodeHaibTfbsGm12878NrsfPcr1xPkRep1 | 0.002647 |
| EncodeHaibTfbsH1hescGabpPcr1xPkRep2 | 0.002647 |
| EncodeHaibTfbsSknshJundV0422111PkRep2 | 0.002644 |
| EncodeSydhTfbsK562Kap1UcdPk | 0.002644 |
| EncodeHaibTfbsU87NrsfPcr2xPkRep1 | 0.002628 |
| EncodeHaibTfbsH1hescFosl1sc183V0416102PkRep2 | 0.002614 |
| EncodeHaibTfbsHct116JundV0422111PkRep2 | 0.002598 |
| EncodeUwHistoneH7esH3k27me3StdDiffa2dPkRep1 | 0.002588 |
| EncodeHaibTfbsH1hescSix5Pcr1xPkRep1 | 0.002584 |
| EncodeUwHistoneSaecH3k36me3StdPkRep2 | 0.002544 |
| EncodeSydhTfbsK562Gata2UcdPk | 0.002527 |
| EncodeSydhTfbsK562CjunIfng30StdPk | 0.002520 |
| EncodeHaibTfbsGm12878SrfPcr2xPkRep2 | 0.002516 |
| EncodeHaibTfbsEcc1SrfV0422111PkRep2 | 0.002511 |
| EncodeHaibTfbsHepg2Bhlhe40V0416101PkRep2 | 0.002507 |
| EncodeHaibTfbsHepg2Sp2V0422111PkRep1 | 0.002501 |
| EncodeHaibTfbsSknshMef2aV0422111PkRep1 | 0.002491 |
| EncodeUwHistoneNhekH3k36me3StdPkRep1 | 0.002443 |
| EncodeSydhTfbsK562Stat2Ifna6hStdPk | 0.002438 |
| EncodeADnaseUwdukeA549UniPk | 0.002432 |
| EncodeHaibTfbsHct116NrsfV0422111PkRep1 | 0.002427 |
| EncodeSydhTfbsK562CjunIfng6hStdPk | 0.002394 |
| EncodeUwDnaseK562Znfb34a8PkRep1 | 0.002375 |
| EncodeSydhTfbsK562Sirt6StdPk | 0.002371 |
| EncodeUwHistoneH7esH3k36me3StdDiffa2dPkRep1 | 0.002363 |
| EncodeSydhTfbsHepg2Grp20ForsklnStdPk | 0.002357 |
| EncodeBroadHistoneHsmmtH3k09me3Pk | 0.002353 |
| EncodeHaibTfbsEcc1Foxm1sc502V0422111PkRep1 | 0.002335 |
| EncodeHaibTfbsHct116Usf1V0422111PkRep1 | 0.002332 |
| EncodeUwHistoneH7esH3k36me3StdPkRep1 | 0.002324 |
| EncodeHaibTfbsHct116NrsfV0422111PkRep2 | 0.002278 |
| EncodeHaibTfbsA549GrPcr1xDex500pmPkRep2 | 0.002272 |
| EncodeUwHistoneHepg2H3k36me3StdPkRep1 | 0.002267 |
| EncodeHaibTfbsSknshFoxm1sc502V0422111PkRep2 | 0.002264 |
| EncodeSydhTfbsK562Brg1IggmusPk | 0.002263 |
| EncodeHaibTfbsPfsk1Foxp2Pcr2xPkRep2 | 0.002252 |
| EncodeHaibTfbsHepg2NrsfPcr2xPkRep1 | 0.002246 |
| EncodeHaibTfbsPanc1NrsfV0422111PkRep1 | 0.002232 |
| EncodeUwHistoneH7esH3k36me3StdDiffa5dPkRep1 | 0.002195 |
| EncodeHaibTfbsH1hescRxraV0416102PkRep1 | 0.002175 |
| EncodeUwDnaseK562Znff41b2PkRep1 | 0.002152 |
| EncodeHaibTfbsH1hescJundV0416102PkRep1 | 0.002149 |
| EncodeHaibTfbsHct116Cebpbsc150V0422111PkRep2 | 0.002145 |
| EncodeUwHistoneK562H3k36me3StdPkRep1 | 0.002135 |
| EncodeSydhTfbsK562Setdb1UcdPk | 0.002119 |
| EncodeUwHistoneH7esH3k36me3StdDiffa9dPkRep2 | 0.002080 |
| EncodeUwHistoneHreH3k27me3StdPkRep1 | 0.002071 |
| EncodeHaibTfbsHct116Cbx3sc101004V0422111PkRep2 | 0.002053 |
| EncodeSydhTfbsK562Gata1bIggmusPk | 0.002052 |
| EncodeHaibTfbsK562SrfV0416101PkRep2 | 0.002051 |
| EncodeSydhTfbsK562Rad21StdPk | 0.002051 |
| EncodeUwHistoneNhekH3k36me3StdPkRep2 | 0.002050 |
| EncodeHaibTfbsT47dEralphaaV0416102Est10nm1hPkRep1 | 0.002029 |
| EncodeUwHistoneH7esH3k27me3StdPkRep1 | 0.002029 |
| EncodeSydhTfbsGm12878Pol3StdPk | 0.002023 |
| EncodeSydhTfbsK562Nfe2StdPk | 0.001994 |
| EncodeBroadHistoneHmecH3k27me3StdPk | 0.001982 |
| EncodeHaibTfbsSknshFoxm1sc502V0422111PkRep1 | 0.001958 |
| EncodeUwHistoneH7esH3k36me3StdDiffa2dPkRep2 | 0.001955 |
| EncodeHaibTfbsMcf7Tead4sc101184V0422111PkRep1 | 0.001946 |
| EncodeSydhTfbsHelas3Irf3IggrabPk | 0.001932 |
| EncodeOpenChromFairePancreasocPk | 0.001929 |
| EncodeSydhTfbsGm12878NfyaIggmusPk | 0.001928 |
| EncodeUwHistoneK562H3k36me3StdPkRep2 | 0.001925 |
| EncodeHaibTfbsHct116Tead4sc101184V0422111PkRep1 | 0.001921 |
| EncodeHaibTfbsK562Six5V0416101PkRep2 | 0.001920 |
| EncodeUwHistoneH7esH3k27me3StdDiffa14dPkRep1 | 0.001911 |
| EncodeHaibTfbsHepg2Hnf4gsc6558V0416101PkRep2 | 0.001909 |
| EncodeSydhTfbsK562Xrcc4StdPk | 0.001873 |
| EncodeHaibTfbsPfsk1NrsfPcr2xPkRep2 | 0.001871 |
| EncodeUwHistoneH7esH3k36me3StdPkRep2 | 0.001867 |
| EncodeHaibTfbsEcc1NrsfV0422111PkRep1 | 0.001821 |
| EncodeUwHistoneH7esH3k27me3StdDiffa5dPkRep1 | 0.001804 |
| EncodeUwHistoneH7esH3k36me3StdDiffa5dPkRep2 | 0.001770 |
| EncodeHaibTfbsHepg2NrsfV0416101PkRep1 | 0.001769 |
| EncodeUwHistoneH7esH3k27me3StdPkRep2 | 0.001757 |
| EncodeOpenChromFaireFrontalcortexocPk | 0.001746 |
| EncodeSydhHistonePbmcH3k27me3bUcdPk | 0.001745 |
| EncodeHaibTfbsGm12892Pax5c20V0416101PkRep1 | 0.001740 |
| EncodeUwHistoneH7esH3k36me3StdDiffa14dPkRep2 | 0.001735 |
| EncodeHaibTfbsA549Tead4sc101184V0422111PkRep2 | 0.001733 |
| EncodeUwHistoneH7esH3k27me3StdDiffa9dPkRep1 | 0.001733 |
| EncodeBroadHistoneHelas3H3k09me3Pk | 0.001719 |
| EncodeHaibTfbsGm12878NrsfPcr2xPkRep2 | 0.001718 |
| EncodeSydhTfbsHepg2Nrf1IggrabPk | 0.001686 |
| EncodeBroadHistoneNhaH3k09me3Pk | 0.001684 |
| EncodeHaibTfbsEcc1EralphaaV0416102Gen1hPkRep1 | 0.001682 |
| EncodeHaibTfbsEcc1GrV0416102Dex100nmPkRep1 | 0.001682 |
| EncodeSydhTfbsGm12878Irf3IggmusPk | 0.001673 |
| EncodeSydhTfbsSknshRfx5IggrabPk | 0.001669 |
| EncodeUwHistoneH7esH3k27me3StdDiffa5dPkRep2 | 0.001668 |
| EncodeSydhTfbsGm12878Yy1StdPk | 0.001658 |
| EncodeUwHistoneSknshraH3k27me3StdPkRep2 | 0.001657 |
| EncodeHaibTfbsPanc1NrsfV0416101PkRep1 | 0.001643 |
| EncodeSydhTfbsHelas3Tcf7l2UcdPk | 0.001632 |
| EncodeHaibTfbsK562Trim28sc81411V0422111PkRep2 | 0.001619 |
| EncodeHaibTfbsMcf7NrsfV0422111PkRep1 | 0.001614 |
| EncodeHaibTfbsSknshP300V0422111PkRep2 | 0.001613 |
| EncodeSydhTfbsHelas3Tf3c110StdPk | 0.001609 |
| EncodeHaibTfbsT47dEralphaaPcr2xGen1hPkRep1 | 0.001599 |
| EncodeHaibTfbsMcf7Tcf12V0422111PkRep1 | 0.001579 |
| EncodeOpenChromFaireColonocPk | 0.001569 |
| EncodeUwHistoneH7esH3k27me3StdDiffa2dPkRep2 | 0.001567 |
| EncodeSydhTfbsHelas3Prdm19115IggrabPk | 0.001559 |
| EncodeUwHistoneH7esH3k27me3StdDiffa14dPkRep2 | 0.001556 |
| EncodeUwHistoneH7esH3k36me3StdDiffa9dPkRep1 | 0.001554 |
| EncodeUwHistoneAg04450H3k09me3StdPkRep1 | 0.001550 |
| EncodeSydhTfbsGm12878Nfe2sc22827StdPk | 0.001536 |
| EncodeHaibTfbsMcf7P300V0422111PkRep2 | 0.001516 |
| EncodeUwHistoneMonocd14ro1746H3k27me3StdPkRep1 | 0.001514 |
| EncodeHaibTfbsGm12878Six5Pcr1xPkRep1 | 0.001509 |
| EncodeHaibTfbsHct116Tead4sc101184V0422111PkRep2 | 0.001501 |
| EncodeUwHistoneSknshraH3k36me3StdPkRep1 | 0.001461 |
| EncodeHaibTfbsT47dJundV0422111PkRep1 | 0.001458 |
| EncodeBroadHistoneNhekH3k09me3Pk | 0.001452 |
| EncodeOpenChromFaireMedulloPk | 0.001443 |
| EncodeHaibTfbsMcf7Fosl2V0422111PkRep2 | 0.001432 |
| EncodeBroadHistoneNhlfH3k09me3Pk | 0.001422 |
| EncodeBroadHistoneH1hescH3k09me3StdPk | 0.001397 |
| EncodeUwHistoneHepg2H3k36me3StdPkRep2 | 0.001385 |
| EncodeUwHistoneH7esH3k27me3StdDiffa9dPkRep2 | 0.001350 |
| EncodeUwDnaseK562Znf2c10c5PkRep1 | 0.001340 |
| EncodeHaibTfbsGm12878Cebpbsc150V0422111PkRep2 | 0.001339 |
| EncodeHaibTfbsEcc1NrsfV0422111PkRep2 | 0.001337 |
| EncodeHaibTfbsMcf7JundV0422111PkRep2 | 0.001337 |
| EncodeUwHistoneSknshraH3k36me3StdPkRep2 | 0.001325 |
| EncodeHaibTfbsU87NrsfPcr2xPkRep2V2 | 0.001321 |
| EncodeHaibTfbsU87NrsfPcr2xPkRep2 | 0.001319 |
| EncodeHaibTfbsA549GrPcr1xDex5nmPkRep1 | 0.001314 |
| EncodeSydhTfbsShsy5yGata3sc269sc269UcdPk | 0.001291 |
| EncodeHaibTfbsPanc1NrsfV0422111PkRep2 | 0.001288 |
| EncodeBroadHistoneDnd41H3k09me3Pk | 0.001280 |
| EncodeUwHistoneBjH3k27me3StdPkRep2 | 0.001271 |
| EncodeHaibTfbsT47dEralphaaPcr2xGen1hPkRep2 | 0.001269 |
| EncodeSydhHistoneNt2d1H3k09me3UcdPk | 0.001269 |
| EncodeUwHistoneHuvecH3k27me3StdPkRep1 | 0.001262 |
| EncodeUwHistoneHuvecH3k27me3StdPkRep2 | 0.001230 |
| EncodeHaibTfbsHepg2Sp1Pcr1xPkRep2 | 0.001222 |
| EncodeHaibTfbsEcc1Foxa1sc6553V0416102Dm002p1hPkRep1 | 0.001221 |
| EncodeHaibTfbsSknmcFoxp2Pcr2xPkRep1 | 0.001218 |
| EncodeHaibTfbsHepg2Tcf12Pcr1xPkRep2 | 0.001208 |
| EncodeUwHistoneHepg2H3k27me3StdPkRep2 | 0.001199 |
| EncodeOpenChromChipH1hescCmycPk | 0.001196 |
| EncodeHaibTfbsPfsk1NrsfPcr2xPkRep1 | 0.001194 |
| EncodeUwHistoneHreH3k27me3StdPkRep2 | 0.001191 |
| EncodeBroadHistoneNhdfadH3k09me3Pk | 0.001110 |
| EncodeSydhTfbsK562Irf1Ifna30StdPk | 0.001103 |
| EncodeBroadHistoneOsteoblH3k9me3StdPk | 0.001090 |
| EncodeHaibTfbsGm12878NrsfPcr2xPkRep1 | 0.001086 |
| EncodeSydhTfbsHepg2ErraForsklnStdPk | 0.001082 |
| EncodeHaibTfbsMcf7NrsfV0422111PkRep2 | 0.001081 |
| EncodeSydhTfbsHelas3Stat1Ifng30StdPk | 0.001073 |
| EncodeHaibTfbsMcf7Foxm1sc502V0422111PkRep1 | 0.001063 |
| EncodeHaibTfbsMcf7Tead4sc101184V0422111PkRep2 | 0.001062 |
| EncodeUwHistoneGm12878H3k27me3StdPkRep2 | 0.001047 |
| EncodeSydhTfbsK562CfosStdPk | 0.001031 |
| EncodeSydhTfbsGm12878Zzz3StdPk | 0.001015 |
| EncodeUwHistoneH7esH3k36me3StdDiffa14dPkRep1 | 0.001010 |
| EncodeHaibTfbsK562Thap1sc98174V0416101PkRep1 | 0.001008 |
| EncodeHaibTfbsA549Gata3V0422111PkRep1 | 0.001006 |
| EncodeSydhTfbsHelas3CfosStdPk | 0.001001 |
| EncodeSydhTfbsPbdefetalGata1UcdPk | 0.000999 |
| EncodeHaibTfbsEcc1EraaV0416102Bpa1hPkRep2 | 0.000963 |
| EncodeUwHistoneSknshraH3k27me3StdPkRep1 | 0.000962 |
| EncodeSydhTfbsHelas3Rpc155StdPk | 0.000961 |
| EncodeUwHistoneSaecH3k27me3StdPkRep1 | 0.000943 |
| EncodeUwHistoneHelas3H3k36me3StdPkRep2 | 0.000933 |
| EncodeSydhTfbsGm12878MafkIggmusPk | 0.000932 |
| EncodeUwDnaseK562Znf2c10c5PkRep2 | 0.000897 |
| EncodeUwHistoneAg04450H3k27me3StdPkRep1 | 0.000897 |
| EncodeHaibTfbsH1hescPou5f1sc9081V0416102PkRep2 | 0.000878 |
| EncodeBroadHistoneHmecH3k09me3Pk | 0.000868 |
| EncodeUwHistoneGm06990H3k27me3StdPkRep1 | 0.000862 |
| EncodeHaibTfbsHepg2Foxa2sc6554V0416101PkRep1 | 0.000846 |
| EncodeUwHistoneHreH3k36me3StdPkRep1 | 0.000835 |
| EncodeHaibTfbsEcc1GrV0416102Dex100nmPkRep2 | 0.000811 |
| EncodeHaibTfbsH1hescPou5f1sc9081V0416102PkRep1 | 0.000804 |
| EncodeHaibTfbsHepg2Bhlhe40V0416101PkRep1 | 0.000803 |
| EncodeOpenChromFaireSmallintestineocPk | 0.000781 |
| EncodeUwHistoneBjH3k27me3StdPkRep1 | 0.000780 |
| EncodeHaibTfbsHepg2NrsfPcr2xPkRep2 | 0.000754 |
| EncodeSydhTfbsK562Pol2s2StdPk | 0.000754 |
| EncodeUwHistoneHreH3k36me3StdPkRep2 | 0.000745 |
| EncodeHaibTfbsHepg2Zeb1V0422111PkRep2 | 0.000743 |
| EncodeSydhTfbsHelas3Zzz3StdPk | 0.000735 |
| EncodeHaibTfbsH1hescSrfPcr1xPkRep2 | 0.000730 |
| EncodeHaibTfbsMcf7P300V0422111PkRep1 | 0.000722 |
| EncodeUwHistoneCaco2H3k36me3StdPkRep2 | 0.000696 |
| EncodeSydhTfbsHelas3Brf2StdPk | 0.000691 |
| EncodeHaibTfbsMcf7Tcf12V0422111PkRep2 | 0.000677 |
| EncodeSydhTfbsHelas3Bdp1StdPk | 0.000674 |
| EncodeSydhTfbsGm12878Spt20StdPk | 0.000668 |
| EncodeHaibTfbsH1hescBcl11aPcr1xPkRep1 | 0.000665 |
| EncodeBroadHistoneHuvecH3k09me3Pk | 0.000664 |
| EncodeHaibTfbsH1hescBcl11aV0416102PkRep2 | 0.000638 |
| EncodeSydhHistoneMcf7H3k09me3UcdPk | 0.000625 |
| EncodeSydhTfbsNt2d1Suz12UcdPk | 0.000613 |
| EncodeUwHistoneAg04450H3k09me3StdPkRep2 | 0.000608 |
| EncodeHaibTfbsHepg2Nr2f2sc271940V0422111PkRep2 | 0.000589 |
| EncodeBroadHistoneMonocd14ro1746H3k09me3Pk | 0.000581 |
| EncodeUwHistoneHelas3H3k36me3StdPkRep1 | 0.000528 |
| EncodeHaibTfbsK562Thap1sc98174V0416101PkRep2 | 0.000526 |
| EncodeSydhTfbsHepg2Tcf7l2UcdPk | 0.000515 |
| EncodeHaibMethylRrbsU87HaibSitesRep2 | 0.000506 |
| EncodeHaibMethylRrbsMyometrDukeSitesRep1 | 0.000505 |
| EncodeHaibMethylRrbsEcc1HaibSitesRep2 | 0.000504 |
| EncodeHaibMethylRrbsPfsk1HaibSitesRep2 | 0.000500 |
| EncodeUwHistoneSaecH3k27me3StdPkRep2 | 0.000500 |
| EncodeHaibTfbsEcc1EraaV0416102Bpa1hPkRep1 | 0.000497 |
| EncodeHaibTfbsHl60NrsfV0422111PkRep2 | 0.000495 |
| EncodeHaibMethylRrbsT47dEstradia24hhHaibSitesRep1 | 0.000491 |
| EncodeHaibMethylRrbsEcc1HaibSitesRep1 | 0.000490 |
| EncodeHaibMethylRrbsPfsk1HaibSitesRep1 | 0.000490 |
| EncodeHaibMethylRrbsU87HaibSitesRep1 | 0.000490 |
| EncodeHaibMethylRrbsT47dDm002p24hHaibSitesRep1 | 0.000483 |
| EncodeHaibMethylRrbsImr90UwSitesRep2 | 0.000478 |
| EncodeHaibMethylRrbsImr90UwstamgrowprotSitesRep2 | 0.000478 |
| EncodeHaibMethylRrbsSknshHaibSitesRep2 | 0.000477 |
| EncodeHaibMethylRrbsBcplacentauhn00189BiochainSitesRep2 | 0.000476 |
| EncodeHaibMethylRrbsBcstomach0111002BiochainSitesRep2 | 0.000476 |
| EncodeHaibMethylRrbsBcskin0111002BiochainSitesRep2 | 0.000471 |
| EncodeSydhHistoneMcf7H3k27me3bUcdPk | 0.000471 |
| EncodeUwHistoneGm12878H3k27me3StdPkRep1 | 0.000470 |
| EncodeHaibMethylRrbsBcskeletalmuscleh12817nBiochainSitesRep2 | 0.000469 |
| EncodeHaibMethylRrbsNhaDukeSitesRep2 | 0.000469 |
| EncodeHaibMethylRrbsHl60UwSitesRep1 | 0.000468 |
| EncodeHaibMethylRrbsA549Dm002p7dHaibSitesRep1 | 0.000467 |
| EncodeHaibMethylRrbsLncapUwSitesRep2 | 0.000464 |
| EncodeHaibMethylRrbsSknshHaibSitesRep1 | 0.000464 |
| EncodeHaibMethylRrbsBjUwSitesRep2 | 0.000461 |
| EncodeHaibMethylRrbsSknmcHaibSitesRep2 | 0.000459 |
| EncodeHaibMethylRrbsRptecSitesRep1 | 0.000458 |
| EncodeBroadHistoneHepg2H3k09me3Pk | 0.000457 |
| EncodeHaibMethylRrbsA549Dm002p7dHaibSitesRep2 | 0.000453 |
| EncodeHaibMethylRrbsHaeUwSitesRep1 | 0.000452 |
| EncodeHaibMethylRrbsHaeUwstamgrowprotSitesRep1 | 0.000452 |
| EncodeHaibMethylRrbsBe2cHaibSitesRep1 | 0.000451 |
| EncodeHaibMethylRrbsNhdfneoUwSitesRep2 | 0.000450 |
| EncodeHaibMethylRrbsNhdfneoUwstamgrowprotSitesRep2 | 0.000450 |
| EncodeHaibMethylRrbsMcf10aesTamStanfordSitesRep2 | 0.000449 |
| EncodeHaibMethylRrbsMcf10aesTamYalestruhlgrowprotSitesRep2 | 0.000449 |
| EncodeHaibMethylRrbsMcf10aesTamStanfordSitesRep3 | 0.000448 |
| EncodeHaibMethylRrbsHcpeUwSitesRep1 | 0.000445 |
| EncodeHaibMethylRrbsHcpeUwstamgrowprotSitesRep1 | 0.000445 |
| EncodeUwHistoneCaco2H3k36me3StdPkRep1 | 0.000445 |
| EncodeHaibMethylRrbsAg10803UwSitesRep1 | 0.000444 |
| EncodeHaibMethylRrbsAg10803UwstamgrowprotSitesRep1 | 0.000444 |
| EncodeHaibMethylRrbsHcmUwSitesRep1 | 0.000442 |
| EncodeHaibMethylRrbsHcmUwstamgrowprotSitesRep1 | 0.000442 |
| EncodeHaibMethylRrbsPanc1HaibSitesRep1 | 0.000441 |
| EncodeHaibMethylRrbsBctestisn30BiochainSitesRep2 | 0.000439 |
| EncodeHaibMethylRrbsAosmcDukeSitesRep1 | 0.000436 |
| EncodeHaibMethylRrbsHnpceUwSitesRep1 | 0.000436 |
| EncodeHaibMethylRrbsHnpceUwstamgrowprotSitesRep1 | 0.000436 |
| EncodeHaibMethylRrbsHtr8DukeSitesRep1 | 0.000436 |
| EncodeHaibMethylRrbsHrpeUwSitesRep1 | 0.000434 |
| EncodeHaibMethylRrbsHrpeUwstamgrowprotSitesRep1 | 0.000434 |
| EncodeHaibMethylRrbsHpaeUwSitesRep1 | 0.000433 |
| EncodeHaibMethylRrbsHpaeUwstamgrowprotSitesRep1 | 0.000433 |
| EncodeHaibMethylRrbsBe2cHaibSitesRep2 | 0.000432 |
| EncodeHaibMethylRrbsHl60UwSitesRep2 | 0.000432 |
| EncodeHaibMethylRrbsNt2d1UwSitesRep2 | 0.000432 |
| EncodeHaibMethylRrbsSknmcHaibSitesRep1 | 0.000432 |
| EncodeHaibMethylRrbsNhaUwSitesRep1 | 0.000431 |
| EncodeHaibMethylRrbsNhaUwstamgrowprotSitesRep1 | 0.000431 |
| EncodeHaibMethylRrbsNhdfneoUwSitesRep1 | 0.000431 |
| EncodeHaibMethylRrbsNhdfneoUwstamgrowprotSitesRep1 | 0.000431 |
| EncodeHaibMethylRrbsGm06990UwSitesRep1 | 0.000428 |
| EncodeHaibMethylRrbsPanc1HaibSitesRep2 | 0.000428 |
| EncodeHaibMethylRrbsPrecUwSitesRep1 | 0.000428 |
| EncodeHaibMethylRrbsAg09309UwSitesRep1 | 0.000427 |
| EncodeHaibMethylRrbsAg09309UwstamgrowprotSitesRep1 | 0.000427 |
| EncodeHaibMethylRrbsMcf10aesStanfordSitesRep2 | 0.000427 |
| EncodeHaibMethylRrbsMcf10aesYalestruhlgrowprotSitesRep2 | 0.000427 |
| EncodeHaibMethylRrbsBcadrenalglandh12803nBiochainSitesRep2 | 0.000424 |
| EncodeHaibMethylRrbsPanc1UwSitesRep1 | 0.000424 |
| EncodeHaibMethylRrbsAg04449UwSitesRep1 | 0.000423 |
| EncodeHaibMethylRrbsAg04449UwstamgrowprotSitesRep1 | 0.000423 |
| EncodeHaibMethylRrbsGm06990UwSitesRep2 | 0.000423 |
| EncodeHaibMethylRrbsBcskeletalmuscle0111002BiochainSitesRep2 | 0.000422 |
| EncodeHaibMethylRrbsHipeUwSitesRep1 | 0.000421 |
| EncodeHaibMethylRrbsHipeUwstamgrowprotSitesRep1 | 0.000421 |
| EncodeHaibMethylRrbsMcf10aesTamStanfordSitesRep1 | 0.000421 |
| EncodeHaibMethylRrbsMcf10aesTamYalestruhlgrowprotSitesRep1 | 0.000421 |
| EncodeHaibMethylRrbsGm12878ximatHaibSitesRep1 | 0.000420 |
| EncodeHaibMethylRrbsGm12878ximatHudsonalphagrowprotSitesRep1 | 0.000420 |
| EncodeHaibMethylRrbsHeeUwSitesRep1 | 0.000419 |
| EncodeHaibMethylRrbsHeeUwstamgrowprotSitesRep1 | 0.000419 |
| EncodeHaibMethylRrbsMcf10aesStanfordSitesRep3 | 0.000419 |
| EncodeHaibMethylRrbsBcbrainh11058nBiochainSitesRep2 | 0.000416 |
| EncodeHaibMethylRrbsBctestisn30BiochainSitesRep1 | 0.000416 |
| EncodeHaibMethylRrbsHreUwSitesRep2 | 0.000416 |
| EncodeHaibMethylRrbsHreUwstamgrowprotSitesRep2 | 0.000416 |
| EncodeHaibMethylRrbsSaecUwSitesRep1 | 0.000416 |
| EncodeHaibMethylRrbsSaecUwSitesRep2 | 0.000416 |
| EncodeHaibMethylRrbsSaecUwstamgrowprotSitesRep1 | 0.000416 |
| EncodeHaibMethylRrbsSaecUwstamgrowprotSitesRep2 | 0.000416 |
| EncodeHaibTfbsH1hescSrfPcr1xPkRep1 | 0.000416 |
| EncodeHaibTfbsMcf7Foxm1sc502V0422111PkRep2 | 0.000416 |
| EncodeHaibTfbsT47dJundV0422111PkRep2 | 0.000415 |
| EncodeHaibMethylRrbsLncapUwSitesRep1 | 0.000414 |
| EncodeHaibMethylRrbsNhbeUwSitesRep1 | 0.000413 |
| EncodeHaibMethylRrbsNhbeUwstamgrowprotSitesRep1 | 0.000413 |
| EncodeHaibMethylRrbsCmkUwSitesRep2 | 0.000412 |
| EncodeHaibMethylRrbsCmkUwstamgrowprotSitesRep2 | 0.000412 |
| EncodeHaibMethylRrbsMcf10aesStanfordSitesRep1 | 0.000412 |
| EncodeHaibMethylRrbsMcf10aesYalestruhlgrowprotSitesRep1 | 0.000412 |
| EncodeHaibMethylRrbsBclung0111002BiochainSitesRep2 | 0.000408 |
| EncodeHaibMethylRrbsAg04450UwSitesRep2 | 0.000404 |
| EncodeHaibMethylRrbsAg04450UwstamgrowprotSitesRep2 | 0.000404 |
| EncodeHaibMethylRrbsHpaeUwSitesRep2 | 0.000404 |
| EncodeHaibMethylRrbsHpaeUwstamgrowprotSitesRep2 | 0.000404 |
| EncodeHaibMethylRrbsSkmcUwSitesRep1 | 0.000404 |
| EncodeHaibMethylRrbsSkmcUwstamgrowprotSitesRep1 | 0.000404 |
| EncodeHaibMethylRrbsHek293UwSitesRep1 | 0.000402 |
| EncodeHaibMethylRrbsHek293UwstamgrowprotSitesRep1 | 0.000402 |
| EncodeHaibMethylRrbsHmecUwSitesRep1 | 0.000400 |
| EncodeHaibMethylRrbsHmecUwstamgrowprotSitesRep1 | 0.000400 |
| EncodeHaibMethylRrbsBcuterusbn0765BiochainSitesRep2 | 0.000399 |
| EncodeHaibMethylRrbsAg04450UwSitesRep1 | 0.000398 |
| EncodeHaibMethylRrbsAg04450UwstamgrowprotSitesRep1 | 0.000398 |
| EncodeSydhTfbsHepg2Srebp2PravastStdPk | 0.000398 |
| EncodeHaibMethylRrbsOsteoblDukeSitesRep1 | 0.000397 |
| EncodeHaibMethylRrbsOsteoblOpenchromgrowprotSitesRep2 | 0.000397 |
| EncodeHaibMethylRrbsBcskin0111002BiochainSitesRep1 | 0.000396 |
| EncodeHaibMethylRrbsHrceUwSitesRep2 | 0.000395 |
| EncodeHaibMethylRrbsHrceUwstamgrowprotSitesRep2 | 0.000395 |
| EncodeHaibMethylRrbsAg09319UwSitesRep2 | 0.000394 |
| EncodeHaibMethylRrbsAg09319UwstamgrowprotSitesRep2 | 0.000394 |
| EncodeHaibMethylRrbsGm19240DukeSitesRep1 | 0.000394 |
| EncodeHaibMethylRrbsGm19240OpenchromgrowprotSitesRep1 | 0.000394 |
| EncodeSydhTfbsK562Znf274m01UcdPk | 0.000393 |
| EncodeHaibMethylRrbsGm12878ximatHaibSitesRep2 | 0.000391 |
| EncodeHaibMethylRrbsGm12878ximatHudsonalphagrowprotSitesRep2 | 0.000391 |
| EncodeHaibMethylRrbsHepatocytesDukeSitesRep2 | 0.000390 |
| EncodeHaibMethylRrbsFibroblDukeSitesRep2 | 0.000389 |
| EncodeHaibMethylRrbsFibroblOpenchromgrowprotSitesRep2 | 0.000389 |
| EncodeSydhTfbsMcf7Gata3UcdPk | 0.000389 |
| EncodeHaibMethylRrbsK562HaibSitesRep2 | 0.000388 |
| EncodeHaibMethylRrbsK562HudsonalphagrowprotSitesRep2 | 0.000388 |
| EncodeHaibMethylRrbsOsteoblDukeSitesRep2 | 0.000388 |
| EncodeHaibMethylRrbsOsteoblOpenchromgrowprotSitesRep1 | 0.000388 |
| EncodeUwHistoneCaco2H3k27me3StdPkRep1 | 0.000388 |
| EncodeHaibMethylRrbsBjUwSitesRep1 | 0.000386 |
| EncodeHaibMethylRrbsGm12891HaibSitesRep2 | 0.000386 |
| EncodeHaibMethylRrbsGm12891HudsonalphagrowprotSitesRep2 | 0.000386 |
| EncodeHaibMethylRrbsBcpericardiumh12529nBiochainSitesRep2 | 0.000381 |
| EncodeSydhTfbsGm12878JundStdPk | 0.000381 |
| EncodeHaibMethylRrbsBcplacentauhn00189BiochainSitesRep1 | 0.000379 |
| EncodeHaibMethylRrbsPanc1UwSitesRep2 | 0.000379 |
| EncodeHaibMethylRrbsNb4UwSitesRep1 | 0.000378 |
| EncodeHaibMethylRrbsNb4UwstamgrowprotSitesRep1 | 0.000378 |
| EncodeHaibMethylRrbsCaco2UwSitesRep2 | 0.000377 |
| EncodeHaibMethylRrbsFibroblDukeSitesRep1 | 0.000376 |
| EncodeHaibMethylRrbsFibroblOpenchromgrowprotSitesRep1 | 0.000376 |
| EncodeHaibMethylRrbsHsmmSitesRep3 | 0.000376 |
| EncodeHaibMethylRrbsRptecSitesRep2 | 0.000375 |
| EncodeHaibMethylRrbsHrpeUwSitesRep2 | 0.000374 |
| EncodeHaibMethylRrbsHrpeUwstamgrowprotSitesRep2 | 0.000374 |
| EncodeHaibMethylRrbsSknshraUwSitesRep2 | 0.000374 |
| EncodeHaibMethylRrbsSknshraUwstamgrowprotSitesRep2 | 0.000374 |
| EncodeHaibTfbsHepg2Nr2f2sc271940V0422111PkRep1 | 0.000374 |
| EncodeHaibMethylRrbsLncapAndroDukeSitesRep2 | 0.000373 |
| EncodeHaibMethylRrbsLncapAndroOpenchromgrowprotSitesRep2 | 0.000373 |
| EncodeHaibMethylRrbsSkmcUwSitesRep2 | 0.000373 |
| EncodeHaibMethylRrbsSkmcUwstamgrowprotSitesRep2 | 0.000373 |
| EncodeHaibMethylRrbsHepatocytesDukeSitesRep1 | 0.000372 |
| EncodeHaibMethylRrbsBcadrenalglandh12803nBiochainSitesRep1 | 0.000371 |
| EncodeHaibMethylRrbsGm19239DukeSitesRep1 | 0.000371 |
| EncodeHaibMethylRrbsGm19239OpenchromgrowprotSitesRep1 | 0.000371 |
| EncodeHaibMethylRrbsT47dEstradia24hhHaibSitesRep2 | 0.000371 |
| EncodeHaibMethylRrbsAg10803UwSitesRep2 | 0.000369 |
| EncodeHaibMethylRrbsAg10803UwstamgrowprotSitesRep2 | 0.000369 |
| EncodeHaibMethylRrbsImr90UwSitesRep1 | 0.000369 |
| EncodeHaibMethylRrbsImr90UwstamgrowprotSitesRep1 | 0.000369 |
| EncodeHaibMethylRrbsUch1DukeSitesRep2 | 0.000368 |
| EncodeHaibMethylRrbsHsmmDukeSitesRep1 | 0.000367 |
| EncodeHaibMethylRrbsHsmmSitesRep1 | 0.000367 |
| EncodeHaibMethylRrbsLncapDukeSitesRep1 | 0.000367 |
| EncodeHaibMethylRrbsMelanoSitesRep1 | 0.000366 |
| EncodeHaibMethylRrbsAg09319UwSitesRep1 | 0.000365 |
| EncodeHaibMethylRrbsAg09319UwstamgrowprotSitesRep1 | 0.000365 |
| EncodeHaibMethylRrbsHrceUwSitesRep1 | 0.000365 |
| EncodeHaibMethylRrbsHrceUwstamgrowprotSitesRep1 | 0.000365 |
| EncodeHaibMethylRrbsT47dDm002p24hHaibSitesRep2 | 0.000365 |
| EncodeHaibMethylRrbsBcskeletalmuscle0111002BiochainSitesRep1 | 0.000364 |
| EncodeHaibMethylRrbsCmkUwSitesRep1 | 0.000364 |
| EncodeHaibMethylRrbsCmkUwstamgrowprotSitesRep1 | 0.000364 |
| EncodeHaibMethylRrbsNhaUwSitesRep2 | 0.000364 |
| EncodeHaibMethylRrbsNhaUwstamgrowprotSitesRep2 | 0.000364 |
| EncodeHaibMethylRrbsAg09309UwSitesRep2 | 0.000363 |
| EncodeHaibMethylRrbsAg09309UwstamgrowprotSitesRep2 | 0.000363 |
| EncodeHaibMethylRrbsGm12878HaibSitesRep2 | 0.000362 |
| EncodeHaibMethylRrbsGm12878HudsonalphagrowprotSitesRep2 | 0.000362 |
| EncodeHaibMethylRrbsAosmcDukeSitesRep2 | 0.000361 |
| EncodeHaibMethylRrbsBcleftventriclen41BiochainSitesRep2 | 0.000361 |
| EncodeHaibMethylRrbsHepg2DukeSitesRep1 | 0.000361 |
| EncodeHaibMethylRrbsHepg2OpenchromgrowprotSitesRep1 | 0.000361 |
| EncodeHaibMethylRrbsBcskeletalmuscleh12817nBiochainSitesRep1 | 0.000360 |
| EncodeHaibMethylRrbsNb4UwSitesRep2 | 0.000360 |
| EncodeHaibMethylRrbsNb4UwstamgrowprotSitesRep2 | 0.000360 |
| EncodeHaibMethylRrbsProgfibSitesRep2 | 0.000360 |
| EncodeHaibMethylRrbsBcstomach0111002BiochainSitesRep1 | 0.000359 |
| EncodeHaibMethylRrbsHct116StanfordSitesRep2 | 0.000359 |
| EncodeHaibMethylRrbsHct116YalegrowprotSitesRep2 | 0.000359 |
| EncodeHaibMethylRrbsCaco2UwSitesRep1 | 0.000357 |
| EncodeHaibMethylRrbsBcpericardiumh12529nBiochainSitesRep1 | 0.000356 |
| EncodeHaibMethylRrbsHcfUwSitesRep1 | 0.000356 |
| EncodeHaibMethylRrbsHcfUwstamgrowprotSitesRep1 | 0.000356 |
| EncodeHaibMethylRrbsSknshraUwSitesRep1 | 0.000356 |
| EncodeHaibMethylRrbsSknshraUwstamgrowprotSitesRep1 | 0.000356 |
| EncodeHaibMethylRrbsGm12892HaibSitesRep2 | 0.000354 |
| EncodeHaibMethylRrbsGm12892HudsonalphagrowprotSitesRep2 | 0.000354 |
| EncodeHaibMethylRrbsBcbreast0203015BiochainSitesRep2 | 0.000353 |
| EncodeHaibMethylRrbsGm19239DukeSitesRep2 | 0.000353 |
| EncodeHaibMethylRrbsGm19239OpenchromgrowprotSitesRep2 | 0.000353 |
| EncodeHaibMethylRrbsHct116StanfordSitesRep1 | 0.000353 |
| EncodeHaibMethylRrbsHct116YalegrowprotSitesRep1 | 0.000353 |
| EncodeHaibMethylRrbsBcleukocyteuhn00204BiochainSitesRep1 | 0.000352 |
| EncodeHaibMethylRrbsBcleftventriclen41BiochainSitesRep1 | 0.000351 |
| EncodeHaibMethylRrbsNt2d1UwSitesRep1 | 0.000349 |
| EncodeHaibMethylRrbsNt2d1UwstamgrowprotSitesRep2 | 0.000349 |
| EncodeHaibMethylRrbsJurkatUwSitesRep1 | 0.000348 |
| EncodeHaibMethylRrbsJurkatUwstamgrowprotSitesRep1 | 0.000348 |
| EncodeHaibMethylRrbsOvcar3UwSitesRep2 | 0.000348 |
| EncodeHaibMethylRrbsHepg2HaibSitesRep2 | 0.000347 |
| EncodeHaibMethylRrbsHepg2HudsonalphagrowprotSitesRep2 | 0.000347 |
| EncodeHaibMethylRrbsHek293StanfordSitesRep2 | 0.000345 |
| EncodeHaibMethylRrbsHek293YalegrowprotSitesRep2 | 0.000345 |
| EncodeHaibMethylRrbsHmecUwSitesRep2 | 0.000345 |
| EncodeHaibMethylRrbsHmecUwstamgrowprotSitesRep2 | 0.000345 |
| EncodeHaibMethylRrbsK562HaibSitesRep1 | 0.000344 |
| EncodeHaibMethylRrbsK562HudsonalphagrowprotSitesRep1 | 0.000344 |
| EncodeHaibMethylRrbsHcpeUwSitesRep2 | 0.000343 |
| EncodeHaibMethylRrbsHcpeUwstamgrowprotSitesRep2 | 0.000343 |
| EncodeHaibMethylRrbsMcf7RandshrnaDukeSitesRep1 | 0.000343 |
| EncodeHaibMethylRrbsProgfibSitesRep1 | 0.000341 |
| EncodeHaibMethylRrbsGm12891HaibSitesRep1 | 0.000340 |
| EncodeHaibMethylRrbsGm12891HudsonalphagrowprotSitesRep1 | 0.000340 |
| EncodeHaibMethylRrbsMcf7CtcfshrnaDukeSitesRep2 | 0.000340 |
| EncodeHaibMethylRrbsGm19240DukeSitesRep2 | 0.000339 |
| EncodeHaibMethylRrbsGm19240OpenchromgrowprotSitesRep2 | 0.000339 |
| EncodeHaibMethylRrbsBcbrainh11058nBiochainSitesRep1 | 0.000338 |
| EncodeHaibMethylRrbsBcbreast0203015BiochainSitesRep1 | 0.000338 |
| EncodeHaibMethylRrbsHcfUwSitesRep2 | 0.000338 |
| EncodeHaibMethylRrbsHcfUwstamgrowprotSitesRep2 | 0.000338 |
| EncodeHaibMethylRrbsHcmUwSitesRep2 | 0.000338 |
| EncodeHaibMethylRrbsHcmUwstamgrowprotSitesRep2 | 0.000338 |
| EncodeHaibMethylRrbsNhbeUwSitesRep2 | 0.000337 |
| EncodeHaibMethylRrbsNhbeUwstamgrowprotSitesRep2 | 0.000337 |
| EncodeHaibMethylRrbsMcf7RandshrnaDukeSitesRep2 | 0.000335 |
| EncodeHaibMethylRrbsHeeUwSitesRep2 | 0.000334 |
| EncodeHaibMethylRrbsHeeUwstamgrowprotSitesRep2 | 0.000334 |
| EncodeHaibMethylRrbsBcuterusbn0765BiochainSitesRep1 | 0.000333 |
| EncodeHaibMethylRrbsHepg2DukeSitesRep2 | 0.000333 |
| EncodeHaibMethylRrbsHepg2OpenchromgrowprotSitesRep2 | 0.000333 |
| EncodeHaibMethylRrbsNt2d1StanfordSitesRep1 | 0.000333 |
| EncodeHaibMethylRrbsNt2d1YalegrowprotSitesRep1 | 0.000333 |
| EncodeHaibMethylRrbsMcf7UwSitesRep1 | 0.000332 |
| EncodeHaibMethylRrbsMcf7UwstamgrowprotSitesRep1 | 0.000332 |
| EncodeHaibMethylRrbsH1hescHaibSitesRep2 | 0.000331 |
| EncodeHaibMethylRrbsH1hescHudsonalphagrowprotSitesRep2 | 0.000331 |
| EncodeHaibMethylRrbsHipeUwSitesRep2 | 0.000331 |
| EncodeHaibMethylRrbsHipeUwstamgrowprotSitesRep2 | 0.000331 |
| EncodeHaibMethylRrbsMcf7DukeSitesRep1 | 0.000331 |
| EncodeHaibMethylRrbsMcf7OpenchromgrowprotSitesRep1 | 0.000331 |
| EncodeHaibMethylRrbsMcf7DukeSitesRep2 | 0.000330 |
| EncodeHaibMethylRrbsMcf7OpenchromgrowprotSitesRep2 | 0.000330 |
| EncodeHaibMethylRrbsBckidney0111002BiochainSitesRep2 | 0.000329 |
| EncodeHaibMethylRrbsHtr8DukeSitesRep3 | 0.000328 |
| EncodeHaibMethylRrbsLncapAndroDukeSitesRep1 | 0.000328 |
| EncodeHaibMethylRrbsLncapAndroOpenchromgrowprotSitesRep1 | 0.000328 |
| EncodeHaibMethylRrbsJurkatUwSitesRep2 | 0.000327 |
| EncodeHaibMethylRrbsJurkatUwstamgrowprotSitesRep2 | 0.000327 |
| EncodeHaibMethylRrbsPanisletsSitesRep5 | 0.000326 |
| EncodeHaibMethylRrbsHsmmDukeSitesRep3 | 0.000324 |
| EncodeHaibMethylRrbsHreUwSitesRep1 | 0.000323 |
| EncodeHaibMethylRrbsHreUwstamgrowprotSitesRep1 | 0.000323 |
| EncodeHaibMethylRrbsHsmmfshdDukeSitesRep1 | 0.000323 |
| EncodeHaibMethylRrbsBclung0111002BiochainSitesRep1 | 0.000319 |
| EncodeHaibMethylRrbsHsmmtubefshdDukeSitesRep3 | 0.000319 |
| EncodeHaibMethylRrbsLncapDukeSitesRep2 | 0.000319 |
| EncodeSydhTfbsH1hescSuz12UcdPk | 0.000319 |
| EncodeHaibMethylRrbsBcpancreash12817nBiochainSitesRep2 | 0.000318 |
| EncodeHaibMethylRrbsHsmmDukeSitesRep2 | 0.000318 |
| EncodeHaibMethylRrbsHsmmSitesRep2 | 0.000318 |
| EncodeHaibMethylRrbsHelas3HaibSitesRep1 | 0.000317 |
| EncodeHaibMethylRrbsHelas3HudsonalphagrowprotSitesRep1 | 0.000317 |
| EncodeHaibMethylRrbsHsmmtDukeSitesRep3 | 0.000316 |
| EncodeHaibMethylRrbsHelas3HaibSitesRep2 | 0.000315 |
| EncodeHaibMethylRrbsHelas3HudsonalphagrowprotSitesRep2 | 0.000315 |
| EncodeHaibMethylRrbsHsmmfshdDukeSitesRep3 | 0.000314 |
| EncodeHaibMethylRrbsHsmmtDukeSitesRep2 | 0.000314 |
| EncodeHaibMethylRrbsHsmmtOpenchromgrowprotSitesRep2 | 0.000314 |
| EncodeHaibMethylRrbsHsmmtubefshdDukeSitesRep2 | 0.000314 |
| EncodeHaibMethylRrbsBcliver0111002BiochainSitesRep2 | 0.000313 |
| EncodeHaibMethylRrbsPanisletsSitesRep4 | 0.000313 |
| EncodeHaibMethylRrbsHepg2HaibSitesRep1 | 0.000312 |
| EncodeHaibMethylRrbsHepg2HudsonalphagrowprotSitesRep1 | 0.000312 |
| EncodeHaibMethylRrbsMcf7StanfordSitesRep1 | 0.000312 |
| EncodeHaibMethylRrbsMcf7YalegrowprotSitesRep1 | 0.000312 |
| EncodeHaibMethylRrbsGm12878HaibSitesRep1 | 0.000311 |
| EncodeHaibMethylRrbsGm12878HudsonalphagrowprotSitesRep1 | 0.000311 |
| EncodeHaibMethylRrbsGm12892HaibSitesRep1 | 0.000311 |
| EncodeHaibMethylRrbsGm12892HudsonalphagrowprotSitesRep1 | 0.000311 |
| EncodeHaibMethylRrbsMcf7CtcfshrnaDukeSitesRep1 | 0.000310 |
| EncodeHaibMethylRrbsAg04449UwSitesRep2 | 0.000309 |
| EncodeHaibMethylRrbsAg04449UwstamgrowprotSitesRep2 | 0.000309 |
| EncodeHaibMethylRrbsBcleukocyteuhn00204BiochainSitesRep2 | 0.000309 |
| EncodeHaibMethylRrbsHsmmtubefshdDukeSitesRep1 | 0.000308 |
| EncodeHaibMethylRrbsHaeUwSitesRep2 | 0.000307 |
| EncodeHaibMethylRrbsHaeUwstamgrowprotSitesRep2 | 0.000307 |
| EncodeHaibMethylRrbsHsmmtDukeSitesRep1 | 0.000307 |
| EncodeHaibMethylRrbsHsmmtOpenchromgrowprotSitesRep1 | 0.000307 |
| EncodeHaibMethylRrbsHek293UwSitesRep2 | 0.000306 |
| EncodeHaibMethylRrbsHek293UwstamgrowprotSitesRep2 | 0.000306 |
| EncodeHaibMethylRrbsNhaDukeSitesRep1 | 0.000306 |
| EncodeUwHistoneNhekH3k27me3StdPkRep2 | 0.000306 |
| EncodeHaibMethylRrbsNt2d1StanfordSitesRep2 | 0.000305 |
| EncodeHaibMethylRrbsNt2d1YalegrowprotSitesRep2 | 0.000305 |
| EncodeHaibMethylRrbsHnpceUwSitesRep2 | 0.000303 |
| EncodeHaibMethylRrbsHnpceUwstamgrowprotSitesRep2 | 0.000303 |
| EncodeHaibMethylRrbsPanisletsSitesRep3 | 0.000302 |
| EncodeHaibMethylRrbsOvcar3UwSitesRep1 | 0.000301 |
| EncodeHaibMethylRrbsPrecUwSitesRep2 | 0.000300 |
| EncodeHaibMethylRrbsMcf7UwSitesRep2 | 0.000298 |
| EncodeHaibMethylRrbsMcf7UwstamgrowprotSitesRep2 | 0.000298 |
| EncodeHaibMethylRrbsHsmmfshdDukeSitesRep2 | 0.000295 |
| EncodeHaibMethylRrbsMyometrDukeSitesRep2 | 0.000294 |
| EncodeSydhTfbsK562Setdb1MnasedUcdPk | 0.000292 |
| EncodeHaibMethylRrbsMelanoSitesRep3 | 0.000291 |
| EncodeHaibMethylRrbsUch1DukeSitesRep1 | 0.000290 |
| EncodeHaibMethylRrbsMcf7StanfordSitesRep2 | 0.000289 |
| EncodeHaibMethylRrbsMcf7YalegrowprotSitesRep2 | 0.000289 |
| EncodeHaibTfbsT47dEraaV0416102Bpa1hPkRep1 | 0.000283 |
| EncodeHaibMethylRrbsHek293StanfordSitesRep1 | 0.000282 |
| EncodeHaibMethylRrbsHek293YalegrowprotSitesRep1 | 0.000282 |
| EncodeUwHistoneNhekH3k27me3StdPkRep1 | 0.000279 |
| EncodeSydhTfbsHelas3Brf1StdPk | 0.000272 |
| EncodeHaibTfbsT47dEraaV0416102Bpa1hPkRep2 | 0.000271 |
| EncodeHaibTfbsEcc1Foxa1sc6553V0416102Dm002p1hPkRep2 | 0.000268 |
| EncodeHaibMethylRrbsBcliver0111002BiochainSitesRep1 | 0.000266 |
| EncodeHaibMethylRrbsH1hescHaibSitesRep1 | 0.000266 |
| EncodeHaibMethylRrbsH1hescHudsonalphagrowprotSitesRep1 | 0.000266 |
| EncodeHaibMethylRrbsMelanoSitesRep2 | 0.000265 |
| EncodeHaibMethylRrbsBcpancreash12817nBiochainSitesRep1 | 0.000259 |
| EncodeHaibMethylRrbsBckidney0111002BiochainSitesRep1 | 0.000252 |
| EncodeHaibMethylRrbsPanisletsSitesRep1 | 0.000243 |
| EncodeUwHistoneHelas3H3k27me3StdPkRep2 | 0.000242 |
| EncodeHaibMethylRrbsPanisletsSitesRep2 | 0.000226 |
| EncodeHaibTfbsHepg2Tead4sc101184V0422111PkRep2 | 0.000185 |
| EncodeSydhTfbsHelas3Znf274UcdPk | 0.000176 |
| EncodeUwHistoneHmecH3k27me3StdPkRep1 | 0.000176 |
| EncodeSydhTfbsHepg2CebpzIggrabPk | 0.000156 |
| EncodeUwHistoneGm06990H3k27me3StdPkRep2 | 0.000155 |
| EncodeUwHistoneCaco2H3k27me3StdPkRep2 | 0.000128 |
| EncodeHaibTfbsA549Foxa1V0416102Dex100nmPkRep2 | 0.000122 |
| EncodeUwHistoneK562H3k27me3StdPkRep1 | 0.000109 |
| EncodeHaibTfbsA549Foxa1V0416102Dex100nmPkRep1 | 0.000098 |
| EncodeUwHistoneK562H3k27me3StdPkRep2 | 0.000092 |
| EncodeSydhHistonePbmcH3k9me3UcdPk | 0.000085 |
| EncodeSydhHistoneU2osH3k9me3UcdPk | 0.000084 |
| EncodeHaibTfbsPanc1NrsfPcr2xPkRep2 | 0.000068 |
| EncodeSydhTfbsGm08714Znf274UcdPk | 0.000029 |
| EncodeSydhTfbsNt2d1Znf274UcdPk | 0.000017 |
| EncodeSydhTfbsHepg2Srebp1PravastStdPk | 0.000011 |
| EncodeHaibTfbsH1hescNanogsc33759V0416102PkRep1 | 0.000009 |
| EncodeSydhTfbsH1hescZnf274m01UcdPk | 0.000006 |
| EncodeSydhTfbsGm12878Znf274StdPk | 0.000000 |
| EncodeSydhTfbsHepg2Znf274UcdPk | 0.000000 |
| EncodeSydhTfbsK562Znf274UcdPk | 0.000000 |
| EncodeUwHistoneHelas3H3k27me3StdPkRep1 | 0.000000 |
| EncodeUwHistoneHepg2H3k27me3StdPkRep1 | 0.000000 |
