# Supplementary material for: The DEK oncoprotein binds to highly and ubiquitously expressed genes with a dual role in their transcriptional regulation
Source: Mol Cancer. 2014 Sep 12;13:215. doi: 10.1186/1476-4598-13-215 (PMC4175287; doi:10.1186/1476-4598-13-215)
Supplement: Supplementary file 3 — Additional file 3: Table S3: Correlation between the binding pattern of DEK and those of other factors in myeloid cells. Complete list of the 201 ChIP-seq experiments in the Encode database that were performed in myeloid cells, ordered from that with the most to that with the least similar binding pattern to that of DEK. (DOCX 23 KB) [file 12943_2014_1416_MOESM3_ESM.docx]

**Additional file 3: Table S3.** **Correlation between the binding pattern of DEK and those of other factors in myeloid cells.** Complete list of the 201 ChIP-seq experiments in the Encode database that were performed in myeloid cells, ordered from that with the most to that with the least similar binding pattern to that of DEK.

| TRACK | | SIMILARITY SCORE | |
| --- | --- | --- | --- |
|  | |  | |
|  | |  | |
| EncodeHaibTfbsHl60Pol24h8V0422111PkRep2 | 0,089874 | |  |
| EncodeHaibTfbsHl60Pol24h8V0422111PkRep1 | 0,071680 | |  |
| EncodeHaibTfbsK562MaxV0416102PkRep2 | 0,064209 | |  |
| EncodeHaibTfbsK562Hey1Pcr1xPkRep1 | 0,056659 | |  |
| EncodeHaibTfbsK562Pol2V0416101PkRep2 | 0,056443 | |  |
| EncodeHaibTfbsHl60Pu1V0422111PkRep1 | 0,054145 | |  |
| EncodeHaibTfbsK562Pol2V0416101PkRep1 | 0,053004 | |  |
| EncodeHaibTfbsK562Pol24h8V0416101PkRep2 | 0,052455 | |  |
| EncodeHaibTfbsK562Cbx3sc101004V0422111PkRep1 | 0,051743 | |  |
| EncodeHaibTfbsK562Hey1Pcr1xPkRep2 | 0,050767 | |  |
| EncodeHaibTfbsK562Elf1sc631V0416102PkRep2 | 0,049904 | |  |
| EncodeHaibTfbsK562Pmlsc71910V0422111PkRep2 | 0,048707 | |  |
| EncodeHaibTfbsK562Taf1V0416101PkRep1 | 0,046753 | |  |
| EncodeHaibTfbsK562Yy1V0416102PkRep2 | 0,045770 | |  |
| EncodeHaibTfbsK562MaxV0416102PkRep1 | 0,045685 | |  |
| EncodeHaibTfbsK562Tead4sc101184V0422111PkRep2 | 0,043648 | |  |
| EncodeHaibTfbsK562Yy1sc281V0416101PkRep1 | 0,042456 | |  |
| EncodeHaibTfbsK562Yy1V0416101PkRep1 | 0,042456 | |  |
| EncodeHaibTfbsK562Nr2f2sc271940V0422111PkRep2 | 0,042307 | |  |
| EncodeSydhTfbsK562Ccnt2StdPk | 0,041932 | |  |
| EncodeSydhTfbsNb4Pol2StdPk | 0,041524 | |  |
| EncodeHaibTfbsK562Pol24h8V0416101PkRep1 | 0,041329 | |  |
| EncodeSydhTfbsK562Hmgn3StdPk | 0,040950 | |  |
| EncodeHaibTfbsK562GabpV0416101PkRep2 | 0,039954 | |  |
| EncodeHaibTfbsK562Taf1V0416101PkRep2 | 0,038745 | |  |
| EncodeSydhTfbsK562CmycIfng30StdPk | 0,038055 | |  |
| EncodeSydhTfbsK562Pol2StdPk | 0,037595 | |  |
| EncodeHaibTfbsK562Elf1sc631V0416102PkRep1 | 0,035325 | |  |
| EncodeSydhTfbsNb4MaxStdPk | 0,034893 | |  |
| EncodeSydhTfbsNb4CmycStdPk | 0,034668 | |  |
| EncodeHaibTfbsK562Trim28sc81411V0422111PkRep1 | 0,033748 | |  |
| EncodeSydhTfbsK562Mazab85725IggrabPk | 0,032525 | |  |
| EncodeSydhTfbsK562CmycIggrabPk | 0,032339 | |  |
| EncodeSydhTfbsK562Bhlhe40nb100IggrabPk | 0,031386 | |  |
| EncodeSydhTfbsK562Pol2Ifng30StdPk | 0,031211 | |  |
| EncodeHaibTfbsK562E2f6sc22823V0416102PkRep2 | 0,030508 | |  |
| EncodeHaibTfbsK562E2f6V0416102PkRep2 | 0,030508 | |  |
| EncodeSydhTfbsK562Pol2Ifng6hStdPk | 0,030152 | |  |
| EncodeSydhTfbsK562JundIggrabPk | 0,028958 | |  |
| EncodeHaibTfbsK562Egr1V0416101PkRep1 | 0,028799 | |  |
| EncodeHaibTfbsHl60Pu1V0422111PkRep2 | 0,028519 | |  |
| EncodeSydhTfbsK562Hcfc1nb10068209IggrabPk | 0,028050 | |  |
| EncodeSydhTfbsK562Gtf2bStdPk | 0,027745 | |  |
| EncodeHaibTfbsK562Pmlsc71910V0422111PkRep1 | 0,027639 | |  |
| EncodeSydhTfbsK562MaxIggrabPk | 0,027638 | |  |
| EncodeHaibTfbsK562Sin3ak20V0416101PkRep2 | 0,027306 | |  |
| EncodeHaibTfbsK562Ets1V0416101PkRep2 | 0,027195 | |  |
| EncodeSydhTfbsK562TbpIggmusPk | 0,027175 | |  |
| EncodeHaibTfbsK562GabpV0416101PkRep1 | 0,026410 | |  |
| EncodeSydhTfbsK562CjunIggrabPk | 0,025590 | |  |
| EncodeHaibTfbsK562Egr1V0416101PkRep2 | 0,025458 | |  |
| EncodeHaibTfbsK562Tead4sc101184V0422111PkRep1 | 0,025337 | |  |
| EncodeSydhTfbsK562Corestsc30189IggrabPk | 0,025178 | |  |
| EncodeHaibTfbsK562Cebpbsc150V0422111PkRep1 | 0,024352 | |  |
| EncodeSydhTfbsK562Znfmizdcp1ab65767IggrabPk | 0,023731 | |  |
| EncodeHaibTfbsK562Zbtb7asc34508V0416101PkRep2 | 0,023164 | |  |
| EncodeHaibTfbsK562Pu1Pcr1xPkRep1 | 0,023066 | |  |
| EncodeHaibTfbsK562Hdac2sc6296V0416102PkRep1 | 0,023052 | |  |
| EncodeSydhTfbsK562Gtf2f1ab28179IggrabPk | 0,022709 | |  |
| EncodeSydhTfbsK562Pol2Ifna6hStdPk | 0,022362 | |  |
| EncodeHaibTfbsK562Ets1V0416101PkRep1 | 0,022107 | |  |
| EncodeHaibTfbsK562Nr2f2sc271940V0422111PkRep1 | 0,021931 | |  |
| EncodeSydhTfbsK562Tblr1ab24550IggrabPk | 0,021403 | |  |
| EncodeSydhTfbsK562Chd2ab68301IggrabPk | 0,021274 | |  |
| EncodeSydhTfbsK562P300IggrabPk | 0,020958 | |  |
| EncodeSydhTfbsK562Pol2s2IggrabPk | 0,020950 | |  |
| EncodeSydhTfbsK562Znf143IggrabPk | 0,020891 | |  |
| EncodeSydhTfbsK562Pol2Ifna30StdPk | 0,020663 | |  |
| EncodeSydhTfbsK562Mxi1af4185IggrabPk | 0,020576 | |  |
| EncodeSydhTfbsK562Ubtfsab1404509IggmusPk | 0,020246 | |  |
| EncodeSydhTfbsK562E2f6UcdPk | 0,019845 | |  |
| EncodeHaibTfbsK562CtcfcPcr1xPkRep1V2 | 0,019752 | |  |
| EncodeSydhTfbsK562Corestab24166IggrabPk | 0,019198 | |  |
| EncodeHaibTfbsK562Stat5asc74442V0422111PkRep1 | 0,018074 | |  |
| EncodeHaibTfbsHl60NrsfV0422111PkRep1 | 0,017946 | |  |
| EncodeSydhTfbsK562Znf384hpa004051IggrabPk | 0,017416 | |  |
| EncodeHaibTfbsK562NrsfV0416102PkRep1 | 0,016883 | |  |
| EncodeHaibTfbsK562CtcfcPcr1xPkRep1 | 0,016777 | |  |
| EncodeSydhTfbsK562Irf1Ifng6hStdPk | 0,016763 | |  |
| EncodeHaibTfbsK562Cebpdsc636V0422111PkRep1 | 0,016609 | |  |
| EncodeSydhTfbsK562Tblr1nb600270IggrabPk | 0,016377 | |  |
| EncodeSydhTfbsK562Zc3h11anb10074650IggrabPk | 0,015195 | |  |
| EncodeHaibTfbsK562Cebpdsc636V0422111PkRep2 | 0,014691 | |  |
| EncodeHaibTfbsK562Usf1V0416101PkRep1 | 0,014658 | |  |
| EncodeHaibTfbsK562Atf3V0416101PkRep2 | 0,014565 | |  |
| EncodeSydhTfbsK562CmycIfna30StdPk | 0,014452 | |  |
| EncodeHaibTfbsK562Rad21V0416102PkRep2 | 0,014162 | |  |
| EncodeSydhTfbsK562Elk112771IggrabPk | 0,014150 | |  |
| EncodeHaibTfbsK562Atf3V0416101PkRep1 | 0,013967 | |  |
| EncodeSydhTfbsK562CebpbIggrabPk | 0,013739 | |  |
| EncodeSydhTfbsK562CmycIfng6hStdPk | 0,013721 | |  |
| EncodeSydhTfbsK562NelfeStdPk | 0,013330 | |  |
| EncodeSydhTfbsK562Ubfsc13125IggmusPk | 0,013274 | |  |
| EncodeHaibTfbsK562Creb1sc240V0422111PkRep1 | 0,013075 | |  |
| EncodeSydhTfbsK562CtcfbIggrabPk | 0,012968 | |  |
| EncodeHaibTfbsK562Pu1Pcr1xPkRep2 | 0,012953 | |  |
| EncodeHaibTfbsK562Yy1sc281V0416101PkRep2 | 0,012909 | |  |
| EncodeHaibTfbsK562Yy1V0416101PkRep2 | 0,012909 | |  |
| EncodeHaibTfbsK562CtcfcPcr1xPkRep2 | 0,012480 | |  |
| EncodeSydhTfbsK562Cdpsc6327IggrabPk | 0,011819 | |  |
| EncodeHaibTfbsK562Cbx3sc101004V0422111PkRep2 | 0,011775 | |  |
| EncodeHaibTfbsK562Zbtb7asc34508V0416101PkRep1 | 0,011150 | |  |
| EncodeSydhTfbsK562Mafkab50322IggrabPk | 0,011106 | |  |
| EncodeHaibTfbsK562Gata2sc267Pcr1xPkRep1 | 0,010768 | |  |
| EncodeSydhTfbsK562CmycIfna6hStdPk | 0,010758 | |  |
| EncodeSydhTfbsK562Irf1Ifng30StdPk | 0,010489 | |  |
| EncodeSydhTfbsK562Atf3StdPk | 0,010456 | |  |
| EncodeSydhTfbsK562Smc3ab9263IggrabPk | 0,010160 | |  |
| EncodeSydhTfbsK562E2f4UcdPk | 0,010075 | |  |
| EncodeHaibTfbsK562Sp1Pcr1xPkRep1 | 0,009909 | |  |
| EncodeSydhTfbsK562Arid3asc8821IggrabPk | 0,009864 | |  |
| EncodeHaibTfbsK562Fosl1sc183V0416101PkRep1 | 0,009768 | |  |
| EncodeHaibTfbsK562Ctcflsc98982V0416101PkRep2 | 0,009688 | |  |
| EncodeHaibTfbsK562Bclaf101388Pcr1xPkRep2 | 0,009515 | |  |
| EncodeHaibTfbsK562E2f6sc22823V0416102PkRep1 | 0,009482 | |  |
| EncodeHaibTfbsK562E2f6V0416102PkRep1 | 0,009482 | |  |
| EncodeSydhTfbsK562CmycStdPk | 0,009335 | |  |
| EncodeSydhTfbsK562Tf3c110StdPk | 0,009154 | |  |
| EncodeHaibTfbsK562Zbtb33Pcr1xPkRep1 | 0,008722 | |  |
| EncodeSydhTfbsK562Pol2IggmusPk | 0,008526 | |  |
| EncodeHaibTfbsK562Rad21V0416102PkRep1 | 0,008208 | |  |
| EncodeSydhTfbsK562Irf1Ifna6hStdPk | 0,008069 | |  |
| EncodeHaibTfbsK562Creb1sc240V0422111PkRep2 | 0,007881 | |  |
| EncodeHaibTfbsK562Zbtb33Pcr1xPkRep2 | 0,007880 | |  |
| EncodeHaibTfbsK562Usf1V0416101PkRep2 | 0,007833 | |  |
| EncodeHaibTfbsK562Cebpbsc150V0422111PkRep2 | 0,007134 | |  |
| EncodeHaibTfbsK562Taf7sc101167V0416101PkRep1 | 0,007085 | |  |
| EncodeSydhTfbsK562Tal1sc12984IggmusPk | 0,007057 | |  |
| EncodeHaibTfbsK562Bclaf101388Pcr1xPkRep1 | 0,007022 | |  |
| EncodeHaibTfbsK562SrfV0416101PkRep1 | 0,006804 | |  |
| EncodeSydhTfbsK562Stat2Ifna30StdPk | 0,006770 | |  |
| EncodeHaibTfbsK562NrsfV0416102PkRep2 | 0,006730 | |  |
| EncodeSydhTfbsK562Atf106325StdPk | 0,006635 | |  |
| EncodeHaibTfbsK562Six5Pcr1xPkRep2 | 0,006216 | |  |
| EncodeHaibTfbsHl60GabpV0422111PkRep1 | 0,006198 | |  |
| EncodeHaibTfbsHl60GabpV0422111PkRep2 | 0,006117 | |  |
| EncodeHaibTfbsK562Fosl1sc183V0416101PkRep2 | 0,006090 | |  |
| EncodeSydhTfbsK562CjunIfna30StdPk | 0,006024 | |  |
| EncodeHaibTfbsK562Hdac2sc6296V0416102PkRep2 | 0,005931 | |  |
| EncodeSydhTfbsK562Rfx5IggrabPk | 0,005903 | |  |
| EncodeSydhTfbsK562CjunStdPk | 0,005865 | |  |
| EncodeSydhTfbsK562MaxStdPk | 0,005750 | |  |
| EncodeHaibTfbsK562Ctcflsc98982V0416101PkRep1 | 0,005627 | |  |
| EncodeSydhTfbsK562P300sc584sc48343IggrabPk | 0,005584 | |  |
| EncodeHaibTfbsK562Sin3ak20V0416101PkRep1 | 0,005380 | |  |
| EncodeSydhTfbsK562Stat1Ifng6hStdPk | 0,005338 | |  |
| EncodeSydhTfbsK562Bach1sc14700IggrabPk | 0,005331 | |  |
| EncodeSydhTfbsK562Ini1IggmusPk | 0,005257 | |  |
| EncodeSydhTfbsK562MaffIggrabPk | 0,005221 | |  |
| EncodeSydhTfbsK562Rpc155StdPk | 0,005179 | |  |
| EncodeSydhTfbsK562Stat1Ifng30StdPk | 0,005082 | |  |
| EncodeSydhTfbsK562Tr4UcdPk | 0,004987 | |  |
| EncodeSydhTfbsK562Yy1UcdPk | 0,004745 | |  |
| EncodeHaibTfbsK562Bcl3Pcr1xPkRep1 | 0,004716 | |  |
| EncodeSydhTfbsK562Nrf1IggrabPk | 0,004649 | |  |
| EncodeHaibTfbsK562Yy1V0416102PkRep1 | 0,004529 | |  |
| EncodeSydhTfbsK562Brf1StdPk | 0,004397 | |  |
| EncodeHaibTfbsK562Six5V0416101PkRep1 | 0,004377 | |  |
| EncodeSydhTfbsK562CjunIfna6hStdPk | 0,004318 | |  |
| EncodeHaibTfbsK562Mef2aV0416101PkRep2 | 0,004177 | |  |
| EncodeHaibTfbsK562Bcl3Pcr1xPkRep2 | 0,004093 | |  |
| EncodeHaibTfbsK562Six5Pcr1xPkRep1 | 0,003768 | |  |
| EncodeSydhTfbsK562Bdp1StdPk | 0,003727 | |  |
| EncodeSydhTfbsK562Usf2IggrabPk | 0,003721 | |  |
| EncodeHaibTfbsK562Taf7sc101167V0416101PkRep2 | 0,003715 | |  |
| EncodeHaibTfbsK562Sp1Pcr1xPkRep2 | 0,003553 | |  |
| EncodeSydhTfbsK562Stat1Ifna6hStdPk | 0,003519 | |  |
| EncodeSydhTfbsK562Stat1Ifna30StdPk | 0,003393 | |  |
| EncodeHaibTfbsK562Sp2sc643V0416102PkRep2 | 0,003251 | |  |
| EncodeHaibTfbsK562Stat5asc74442V0422111PkRep2 | 0,003203 | |  |
| EncodeHaibTfbsK562Mef2aV0416101PkRep1 | 0,003179 | |  |
| EncodeSydhTfbsK562NfybStdPk | 0,003058 | |  |
| EncodeHaibTfbsK562Gata2sc267Pcr1xPkRep2 | 0,003056 | |  |
| EncodeHaibTfbsK562Sp2sc643V0416102PkRep1 | 0,003050 | |  |
| EncodeSydhTfbsK562Znf263UcdPk | 0,003047 | |  |
| EncodeSydhTfbsK562Gata1UcdPk | 0,002987 | |  |
| EncodeSydhTfbsK562NfyaStdPk | 0,002705 | |  |
| EncodeSydhTfbsK562Kap1UcdPk | 0,002644 | |  |
| EncodeSydhTfbsK562Gata2UcdPk | 0,002527 | |  |
| EncodeSydhTfbsK562CjunIfng30StdPk | 0,002520 | |  |
| EncodeSydhTfbsK562Stat2Ifna6hStdPk | 0,002438 | |  |
| EncodeSydhTfbsK562CjunIfng6hStdPk | 0,002394 | |  |
| EncodeSydhTfbsK562Sirt6StdPk | 0,002371 | |  |
| EncodeSydhTfbsK562Brg1IggmusPk | 0,002263 | |  |
| EncodeSydhTfbsK562Setdb1UcdPk | 0,002119 | |  |
| EncodeSydhTfbsK562Gata1bIggmusPk | 0,002052 | |  |
| EncodeHaibTfbsK562SrfV0416101PkRep2 | 0,002051 | |  |
| EncodeSydhTfbsK562Rad21StdPk | 0,002051 | |  |
| EncodeSydhTfbsK562Nfe2StdPk | 0,001994 | |  |
| EncodeHaibTfbsK562Six5V0416101PkRep2 | 0,001920 | |  |
| EncodeSydhTfbsK562Xrcc4StdPk | 0,001873 | |  |
| EncodeHaibTfbsK562Trim28sc81411V0422111PkRep2 | 0,001619 | |  |
| EncodeSydhTfbsK562Irf1Ifna30StdPk | 0,001103 | |  |
| EncodeSydhTfbsK562CfosStdPk | 0,001031 | |  |
| EncodeHaibTfbsK562Thap1sc98174V0416101PkRep1 | 0,001008 | |  |
| EncodeSydhTfbsK562Pol2s2StdPk | 0,000754 | |  |
| EncodeHaibTfbsK562Thap1sc98174V0416101PkRep2 | 0,000526 | |  |
| EncodeHaibTfbsHl60NrsfV0422111PkRep2 | 0,000495 | |  |
| EncodeSydhTfbsK562Znf274m01UcdPk | 0,000393 | |  |
| EncodeSydhTfbsK562Setdb1MnasedUcdPk | 0,000292 | |  |
| EncodeSydhTfbsK562Znf274UcdPk | 0,000000 | |  |
