# Supplementary material for: The DEK oncoprotein binds to highly and ubiquitously expressed genes with a dual role in their transcriptional regulation
Source: Mol Cancer. 2014 Sep 12;13:215. doi: 10.1186/1476-4598-13-215 (PMC4175287; doi:10.1186/1476-4598-13-215)
Supplement: Supplementary file 4 — Additional file 4: Table S4: DEK binding correlates with RNA polymerase II binding in hematopoietic datasets. All ChIP-seq experiments in the four hematopoietic datasets in the Encode database were scored and ranked based on their similarity with the DEK binding pattern. In all four datasets, the binding patterns of RNA polymerase II were among the most similar to that of DEK. (DOCX 14 KB) [file 12943_2014_1416_MOESM4_ESM.docx]

**Additional file 4: Table S4. DEK binding correlates with RNA polymerase II binding in hematopoietic datasets.** All ChIP-seq experiments in the four hematopoietic datasets in the Encode database were scored and ranked based on their similarity with the DEK binding pattern. In all four datasets, the binding patterns of RNA polymerase II were among the most similar to that of DEK.

| CELL LINE | | DATASET | | RANKS OF POL2 TRACKS | | NUMBER OF TRACKS | |
| --- | --- | --- | --- | --- | --- | --- | --- |
|  | |  | |  | |  | |
|  | |  | |  | |  | |
|  | NB4 |  | SYDH | #1 |  | 3 |  |
|  | HL60 |  | HAIB | #1, #2 |  | 8 |  |
|  | K562 |  | HAIB | #3, #4 |  | 93 |  |
|  | K562 |  | SYDH | #4 |  | 97 |  |
|  |  |  |  |  |  |  |  |
