# Supplementary material for: The DEK oncoprotein binds to highly and ubiquitously expressed genes with a dual role in their transcriptional regulation
Source: Mol Cancer. 2014 Sep 12;13:215. doi: 10.1186/1476-4598-13-215 (PMC4175287; doi:10.1186/1476-4598-13-215)
Supplement: Supplementary file 5 — Additional file 5: Table S5: DEK binds to commonly expressed genes. Complete list of the 1172 analyzed transcription factors, based on their binding to genes that are commonly expressed across different tissues. For each transcription factor, the overlap with each of the other binding patterns in the Encode database was calculated. The resulting ranks of the POL2 binding patterns were extracted and the median rank was calculated, representing the similarity between the binding pattern of the transcription factor and that of POL2 across all the different cell types represented in Encode. DEK was found to be one of the transcription factors with the highest similarity, demonstrating that DEK binds to genes that are commonly expressed across different cell types. (DOCX 61 KB) [file 12943_2014_1416_MOESM5_ESM.docx]

**Additional file 5: Table S5.** **DEK binds to commonly expressed genes.** Complete list of the 1172 analyzed transcription factors, based on their binding to genes that are commonly expressed across different tissues. For each transcription factor, the overlap with each of the other binding patterns in the Encode database was calculated. The resulting ranks of the POL2 binding patterns were extracted and the median rank was calculated, representing the similarity between the binding pattern of the transcription factor and that of POL2 across all the different cell types represented in Encode. DEK was found to be one of the transcription factors with the highest similarity, demonstrating that DEK binds to genes that are commonly expressed across different cell types.

| TRACK | MEDIAN RANK OF ALL POL2 TRACKS |
| --- | --- |
|  |  |
|  |  |
| EncodeHaibTfbsPfsk1Pol24h8V0416101PkRep2 | 57 |
| EncodeSydhTfbsA549Pol2s2IggrabPk | 58 |
| EncodeSydhTfbsGm12891Pol2IggmusPk | 59 |
| EncodeSydhTfbsK562Pol2Ifna30StdPk | 59 |
| EncodeSydhTfbsGm12878Pol2StdPk | 63 |
| EncodeSydhTfbsGm12878Pol2IggmusPk | 63 |
| EncodeSydhTfbsHuvecPol2StdPk | 65 |
| EncodeSydhTfbsK562Pol2Ifna6hStdPk | 66 |
| EncodeSydhTfbsHepg2Pol2PravastStdPk | 72 |
| EncodeSydhTfbsPbdePol2UcdPk | 74 |
| EncodeHaibTfbsHl60Pol24h8V0422111PkRep1 | 75 |
| EncodeSydhTfbsGm12892Pol2IggmusPk | 75 |
| EncodeSydhTfbsHelas3Pol2StdPk | 75 |
| EncodeSydhTfbsGm18505Pol2IggmusPk | 76 |
| EncodeSydhTfbsGm19193Pol2IggmusPk | 80 |
| EncodeSydhTfbsHct116Pol2UcdPk | 81 |
| EncodeSydhTfbsK562Pol2s2IggrabPk | 81 |
| EncodeSydhTfbsGm19099Pol2IggmusPk | 82 |
| EncodeSydhTfbsK562Pol2Ifng30StdPk | 83 |
| EncodeHaibTfbsA549Bcl3V0422111Etoh02PkRep1 | 83 |
| EncodeHaibTfbsH1hescTaf7sc101167V0416102PkRep2 | 83 |
| EncodeHaibTfbsHuvecPol2Pcr1xPkRep2 | 84 |
| EncodeSydhTfbsGm18526Pol2IggmusPk | 85 |
| EncodeSydhTfbsK562Pol2Ifng6hStdPk | 85 |
| EncodeSydhTfbsGm10847Pol2IggmusPk | 91 |
| EncodeHaibTfbsK562Bcl3Pcr1xPkRep2 | 91 |
| EncodeSydhTfbsGm18951Pol2IggmusPk | 91 |
| EncodeSydhTfbsImr90Chd1nb10060411IggrabPk | 93 |
| EncodeHaibTfbsH1hescAtf2sc81188V0422111PkRep2 | 94 |
| EncodeSydhTfbsRajiPol2UcdPk | 94 |
| EncodeHaibTfbsK562Pmlsc71910V0422111PkRep1 | 96 |
| EncodeSydhTfbsK562Pol2StdPk | 96 |
| EncodeSydhTfbsNb4Pol2StdPk | 96 |
| EncodeSydhTfbsGm15510Pol2IggmusPk | 97 |
| EncodeHaibTfbsA549Pbx3V0422111PkRep1 | 98 |
| EncodeSydhTfbsHek293Pol2StdPk | 99 |
| EncodeHaibTfbsEcc1Pol2V0416102Dm002p1hPkRep1 | 101 |
| EncodeSydhTfbsImr90Pol2IggrabPk | 101 |
| EncodeHaibTfbsGm12891Taf1Pcr1xPkRep2 | 103 |
| DEK | 104 |
| EncodeHaibTfbsGm12878Nfatc1sc17834V0422111PkRep2 | 107 |
| EncodeSydhTfbsHepg2Pol2ForsklnStdPk | 107 |
| EncodeHaibTfbsEcc1Taf1V0422111PkRep2 | 108 |
| EncodeHaibTfbsGm12892Taf1V0416102PkRep2 | 108 |
| EncodeSydhTfbsImr90Rfx5IggrabPk | 108 |
| EncodeSydhTfbsSknshRfx5IggrabPk | 108 |
| EncodeHaibTfbsGm12878Nfatc1sc17834V0422111PkRep1 | 109 |
| EncodeHaibTfbsU87Pol24h8V0416101PkRep1 | 112 |
| EncodeSydhTfbsGm12878Gcn5StdPk | 112 |
| EncodeHaibTfbsA549Taf1V0422111Etoh02PkRep1 | 114 |
| EncodeHaibTfbsK562Bclaf101388Pcr1xPkRep2 | 114 |
| EncodeHaibTfbsGm12891Pou2f2Pcr1xPkRep2 | 116 |
| EncodeHaibTfbsHct116Sin3ak20V0422111PkRep1 | 116 |
| EncodeHaibTfbsMcf7Pmlsc71910V0422111PkRep1 | 116 |
| EncodeHaibTfbsK562Bclaf101388Pcr1xPkRep1 | 116 |
| EncodeHaibTfbsA549Bcl3V0422111Etoh02PkRep2 | 117 |
| EncodeHaibTfbsGm12891Taf1Pcr1xPkRep1 | 117 |
| EncodeSydhTfbsHepg2Pol2s2IggrabPk | 118 |
| EncodeHaibTfbsHepg2Mbd4sc271530V0422111PkRep2 | 118 |
| EncodeHaibTfbsMcf7Taf1V0422111PkRep1 | 119 |
| EncodeSydhTfbsHelas3Gcn5StdPk | 119 |
| EncodeHaibTfbsK562Bcl3Pcr1xPkRep1 | 120 |
| EncodeSydhTfbsH1hescChd1a301218aIggrabPk | 120 |
| EncodeHaibTfbsGm12878Taf1Pcr1xPkRep2 | 121 |
| EncodeHaibTfbsHuvecPol2Pcr1xPkRep1 | 121 |
| EncodeHaibTfbsH1neuronsTaf1V0422111PkRep1 | 121 |
| EncodeHaibTfbsH1neuronsTaf1V0422111PkRep2 | 121 |
| EncodeHaibTfbsMcf7Taf1V0422111PkRep2 | 123 |
| EncodeSydhTfbsSknshNrf1IggrabPk | 123 |
| EncodeHaibTfbsHct116Sin3ak20V0422111PkRep2 | 124 |
| EncodeHaibTfbsHepg2Taf1Pcr2xPkRep2 | 125 |
| EncodeHaibTfbsH1hescTaf7sc101167V0416102PkRep1 | 126 |
| EncodeHaibTfbsEcc1Taf1V0422111PkRep1 | 127 |
| EncodeHaibTfbsHepg2Taf1Pcr2xPkRep1 | 127 |
| EncodeSydhTfbsK562Gtf2bStdPk | 127 |
| EncodeSydhTfbsGm15510NfkbTnfaIggrabPk | 128 |
| EncodeSydhTfbsK562Znf263UcdPk | 128 |
| EncodeHaibTfbsK562Taf1V0416101PkRep2 | 129 |
| EncodeSydhTfbsImr90Mazab85725IggrabPk | 129 |
| EncodeHaibTfbsPanc1Sin3ak20V0416101PkRep1 | 130 |
| EncodeSydhTfbsK562CmycIfng6hStdPk | 130 |
| EncodeHaibTfbsGm12892Taf1V0416102PkRep1 | 131 |
| EncodeHaibTfbsH1neuronsNrsfV0422111PkRep1 | 131 |
| EncodeHaibTfbsSknshTaf1V0416101PkRep1 | 131 |
| EncodeHaibTfbsMcf7Pmlsc71910V0422111PkRep2 | 132 |
| EncodeHaibTfbsPfsk1Taf1V0416101PkRep1 | 132 |
| EncodeHaibTfbsA549Pbx3V0422111PkRep2 | 132 |
| EncodeHaibTfbsK562Creb1sc240V0422111PkRep2 | 133 |
| EncodeHaibTfbsMcf7GabpV0422111PkRep2 | 134 |
| EncodeHaibTfbsK562Taf7sc101167V0416101PkRep2 | 135 |
| EncodeSydhTfbsK562NelfeStdPk | 135 |
| EncodeHaibTfbsHct116Zbtb33V0416101PkRep1 | 136 |
| EncodeHaibTfbsH1neuronsNrsfV0422111PkRep2 | 136 |
| EncodeSydhTfbsK562Pol2IggmusPk | 136 |
| EncodeSydhTfbsHelas3E2f1StdPk | 137 |
| EncodeSydhTfbsHelas3Nrf1IggmusPk | 137 |
| EncodeHaibTfbsA549Zbtb33V0422111Etoh02PkRep2 | 138 |
| EncodeHaibTfbsGm12878Mta3sc81325V0422111PkRep1 | 139 |
| EncodeHaibTfbsSknshYy1sc281V0422111PkRep1 | 139 |
| EncodeSydhTfbsHepg2Nrf1IggrabPk | 139 |
| EncodeSydhTfbsHelas3Hae2f1StdPk | 139 |
| EncodeHaibTfbsH1hescTaf1V0416102PkRep2 | 140 |
| EncodeSydhTfbsA549CmycIggrabPk | 140 |
| EncodeHaibTfbsSknshGabpV0422111PkRep1 | 141 |
| EncodeHaibTfbsSknshGabpV0422111PkRep2 | 141 |
| EncodeHaibTfbsSknshZbtb33V0422111PkRep1 | 141 |
| EncodeSydhTfbsImr90Mxi1IggrabPk | 141 |
| EncodeSydhTfbsGm12878ErraIggrabPk | 142 |
| EncodeSydhTfbsK562Yy1UcdPk | 142 |
| EncodeHaibTfbsH1hescAtf2sc81188V0422111PkRep1 | 142 |
| EncodeHaibTfbsK562Taf1V0416101PkRep1 | 143 |
| EncodeSydhTfbsK562CmycIfna6hStdPk | 143 |
| EncodeHaibTfbsHepg2Creb1sc240V0422111PkRep2 | 143 |
| EncodeSydhTfbsSknshMxi1IggrabPk | 143 |
| EncodeHaibTfbsK562Creb1sc240V0422111PkRep1 | 144 |
| EncodeSydhTfbsK562CmycStdPk | 144 |
| EncodeSydhTfbsK562Stat2Ifna30StdPk | 144 |
| EncodeHaibTfbsPanc1Sin3ak20V0416101PkRep2 | 145 |
| EncodeSydhTfbsK562E2f4UcdPk | 146 |
| EncodeSydhTfbsHelas3Ini1IggmusPk | 146 |
| EncodeSydhTfbsGm10847NfkbTnfaIggrabPk | 147 |
| EncodeHaibTfbsHepg2Creb1sc240V0422111PkRep1 | 147 |
| EncodeHaibTfbsHl60GabpV0422111PkRep1 | 148 |
| EncodeHaibTfbsSknshZbtb33V0422111PkRep2 | 148 |
| EncodeSydhTfbsNt2d1Yy1UcdPk | 148 |
| EncodeHaibTfbsH1hescPol24h8V0416102PkRep2 | 149 |
| EncodeHaibTfbsHl60GabpV0422111PkRep2 | 150 |
| EncodeHaibTfbsSknshElf1V0422111PkRep1 | 150 |
| EncodeSydhTfbsGm12891NfkbTnfaIggrabPk | 150 |
| EncodeSydhTfbsK562Stat2Ifna6hStdPk | 150 |
| EncodeSydhTfbsMcf10aesPol2Etoh01StdPk | 151 |
| EncodeSydhTfbsMcf7Hae2f1UcdPk | 151 |
| EncodeHaibTfbsGm12878Pmlsc71910V0422111PkRep1 | 151 |
| EncodeHaibTfbsGm12891Yy1sc281V0416101PkRep1 | 151 |
| EncodeHaibTfbsGm12878GabpPcr2xPkRep2 | 152 |
| EncodeSydhTfbsGm12892NfkbTnfaIggrabPk | 152 |
| EncodeSydhTfbsGm18505NfkbTnfaIggrabPk | 152 |
| EncodeHaibTfbsSknshYy1sc281V0422111PkRep2 | 152 |
| EncodeSydhTfbsHelas3Pol2s2IggrabPk | 152 |
| EncodeHaibTfbsPanc1Pol24h8V0416101PkRep1 | 153 |
| EncodeHaibTfbsHepg2Yy1sc281V0416101PkRep2 | 154 |
| EncodeHaibTfbsGm12878GabpPcr2xPkRep1 | 154 |
| EncodeHaibTfbsHct116Elf1V0422111PkRep2 | 154 |
| EncodeHaibTfbsHepg2MaxV0422111PkRep2 | 155 |
| EncodeHaibTfbsGm12878Elf1sc631V0416101PkRep1 | 155 |
| EncodeHaibTfbsHelas3Taf1Pcr1xPkRep1 | 155 |
| EncodeHaibTfbsGm12878Bclaf101388V0416101PkRep2 | 156 |
| EncodeHaibTfbsGm12878SrfPcr2xPkRep2 | 156 |
| EncodeHaibTfbsHct116Egr1V0422111PkRep2 | 156 |
| EncodeHaibTfbsK562Yy1V0416101PkRep2 | 156 |
| EncodeHaibTfbsK562Yy1sc281V0416101PkRep2 | 156 |
| EncodeHaibTfbsGm12878Bcl3V0416101PkRep1 | 156 |
| EncodeHaibTfbsH1hescE2f6V0422111PkRep2 | 157 |
| EncodeHaibTfbsHct116Egr1V0422111PkRep1 | 157 |
| EncodeHaibTfbsHct116SrfV0422111PkRep1 | 157 |
| EncodeHaibTfbsHepg2GabpPcr2xPkRep2 | 157 |
| EncodeHaibTfbsK562Sin3ak20V0416101PkRep2 | 157 |
| EncodeHaibTfbsK562Taf7sc101167V0416101PkRep1 | 157 |
| EncodeHaibTfbsSknshElf1V0422111PkRep2 | 157 |
| EncodeHaibTfbsHepg2MaxV0422111PkRep1 | 157 |
| EncodeHaibTfbsA549Elf1V0422111Etoh02PkRep1 | 158 |
| EncodeHaibTfbsA549GabpV0422111Etoh02PkRep2 | 158 |
| EncodeHaibTfbsGm12878Creb1sc240V0422111PkRep2 | 158 |
| EncodeSydhTfbsK562CmycIfna30StdPk | 158 |
| EncodeHaibTfbsGm12892Yy1V0416101PkRep2 | 159 |
| EncodeHaibTfbsHepg2GabpPcr2xPkRep1 | 159 |
| EncodeHaibTfbsMcf7Sin3ak20V0422111PkRep2 | 159 |
| EncodeSydhTfbsK562Nrf1IggrabPk | 159 |
| EncodeSydhTfbsMcf10aesPol2TamStdPk | 159 |
| EncodeHaibTfbsA549Six5V0422111Etoh02PkRep2 | 160 |
| EncodeHaibTfbsH1hescTaf1V0416102PkRep1 | 160 |
| EncodeHaibTfbsPfsk1Taf1V0416101PkRep2 | 160 |
| EncodeHaibTfbsGm12892Pol2V0416102PkRep2 | 160 |
| EncodeHaibTfbsHct116MaxV0422111PkRep2 | 160 |
| EncodeHaibTfbsA549E2f6V0422111PkRep1 | 161 |
| EncodeHaibTfbsGm12878Egr1Pcr2xPkRep3 | 161 |
| EncodeHaibTfbsA549Pol2Pcr2xDex100nmPkRep1 | 161 |
| EncodeHaibTfbsGm12878Stat5asc74442V0422111PkRep1 | 162 |
| EncodeHaibTfbsGm12878Taf1Pcr1xPkRep1 | 162 |
| EncodeHaibTfbsHepg2Cebpdsc636V0416101PkRep1 | 163 |
| EncodeHaibTfbsMcf7SrfV0422111PkRep1 | 163 |
| EncodeSydhTfbsHepg2Srebp1InslnStdPk | 163 |
| EncodeHaibTfbsA549Taf1V0422111Etoh02PkRep2 | 164 |
| EncodeSydhTfbsGm12878MafkIggmusPk | 164 |
| EncodeSydhTfbsK562E2f6UcdPk | 164 |
| EncodeHaibTfbsGm12878SrfPcr2xPkRep1 | 164 |
| EncodeHaibTfbsGm12891Pol2Pcr1xPkRep2 | 164 |
| EncodeSydhTfbsHepg2Tr4UcdPk | 164 |
| EncodeHaibTfbsEcc1Creb1sc240V0422111PkRep1 | 165 |
| EncodeHaibTfbsHct116MaxV0422111PkRep1 | 165 |
| EncodeSydhTfbsH1hescNrf1IggrabPk | 165 |
| EncodeHaibTfbsK562Cebpdsc636V0422111PkRep2 | 165 |
| EncodeSydhTfbsGm12878Sin3anb6001263IggmusPk | 166 |
| EncodeHaibTfbsA549Yy1cV0422111Etoh02PkRep2 | 167 |
| EncodeHaibTfbsH1hescCreb1sc240V0422111PkRep2 | 167 |
| EncodeSydhTfbsGm12878JundIggrabPk | 167 |
| EncodeSydhTfbsK562Setdb1UcdPk | 167 |
| EncodeSydhTfbsK562Stat1Ifna6hStdPk | 167 |
| EncodeHaibTfbsMcf7MaxV0422111PkRep2 | 168 |
| EncodeSydhTfbsHelas3E2f4StdPk | 168 |
| EncodeSydhTfbsK562Stat1Ifng6hStdPk | 168 |
| EncodeHaibTfbsA549Sin3ak20V0422111Etoh02PkRep2 | 169 |
| EncodeHaibTfbsGm12891Pou2f2Pcr1xPkRep1 | 169 |
| EncodeHaibTfbsK562Sin3ak20V0416101PkRep1 | 169 |
| EncodeHaibTfbsHct116Elf1V0422111PkRep1 | 169 |
| EncodeHaibTfbsK562Ets1V0416101PkRep1 | 169 |
| EncodeHaibTfbsHepg2Pol2Pcr2xPkRep1 | 170 |
| EncodeHaibTfbsA549Zbtb33V0422111Etoh02PkRep1 | 171 |
| EncodeHaibTfbsHepg2Zeb1V0422111PkRep1 | 171 |
| EncodeSydhTfbsGm12878Brca1a300IggmusPk | 171 |
| EncodeSydhTfbsGm19099NfkbTnfaIggrabPk | 171 |
| EncodeSydhTfbsK562Stat1Ifna30StdPk | 171 |
| EncodeHaibTfbsEcc1Zbtb7aV0422111PkRep1 | 171 |
| EncodeSydhTfbsGm19193NfkbTnfaIggrabPk | 171 |
| exp1_peaks | 172 |
| EncodeSydhTfbsGm12878E2f4IggmusPk | 172 |
| EncodeSydhTfbsGm12878Pol2s2IggmusPk | 172 |
| EncodeHaibTfbsGm12878Ets1Pcr1xPkRep1V2 | 173 |
| EncodeHaibTfbsHepg2Zbtb33V0416101PkRep1 | 173 |
| EncodeHaibTfbsMcf7GabpV0422111PkRep1 | 173 |
| EncodeSydhTfbsH1hescRfx5200401194IggrabPk | 173 |
| EncodeSydhTfbsHelas3Elk4UcdPk | 173 |
| EncodeHaibTfbsPfsk1Sin3ak20V0416101PkRep1 | 174 |
| EncodeSydhTfbsHelas3Hcfc1nb10068209IggrabPk | 174 |
| EncodeHaibTfbsA549Sin3ak20V0422111Etoh02PkRep1 | 175 |
| EncodeHaibTfbsPanc1Pol24h8V0416101PkRep2 | 175 |
| EncodeSydhTfbsGm18526NfkbTnfaIggrabPk | 175 |
| EncodeHaibTfbsHuvecPol24h8V0416101PkRep1 | 175 |
| EncodeSydhTfbsHelas3E2f6StdPk | 175 |
| EncodeHaibTfbsGm12878Creb1sc240V0422111PkRep1 | 176 |
| EncodeHaibTfbsHl60NrsfV0422111PkRep1 | 176 |
| EncodeHaibTfbsK562Yy1V0416102PkRep1 | 176 |
| EncodeHaibTfbsK562Zbtb7asc34508V0416101PkRep1 | 176 |
| EncodeHaibTfbsGm12878Foxm1sc502V0422111PkRep2 | 176 |
| EncodeHaibTfbsEcc1Yy1sc281V0422111PkRep1 | 177 |
| EncodeHaibTfbsEcc1Zbtb7aV0422111PkRep2 | 177 |
| EncodeHaibTfbsMcf7Egr1V0422111PkRep1 | 177 |
| EncodeHaibTfbsK562Thap1sc98174V0416101PkRep1 | 178 |
| EncodeHaibTfbsMcf7Elf1V0422111PkRep2 | 178 |
| EncodeSydhTfbsGm18951NfkbTnfaIggrabPk | 179 |
| EncodeHaibTfbsGm12878Cebpbsc150V0422111PkRep1 | 180 |
| EncodeHaibTfbsPfsk1Foxp2Pcr2xPkRep1 | 180 |
| EncodeHaibTfbsEcc1Egr1V0422111PkRep1 | 180 |
| EncodeHaibTfbsK562GabpV0416101PkRep2 | 180 |
| EncodeHaibTfbsK562Pmlsc71910V0422111PkRep2 | 180 |
| EncodeSydhTfbsHek293Elk4UcdPk | 180 |
| EncodeHaibTfbsGm12892Pol24h8V0416102PkRep2 | 181 |
| EncodeHaibTfbsHepg2Zbtb33V0416101PkRep2 | 181 |
| EncodeHaibTfbsK562Trim28sc81411V0422111PkRep1 | 181 |
| EncodeHaibTfbsA549E2f6V0422111PkRep2 | 182 |
| EncodeHaibTfbsGm12892Pax5c20V0416101PkRep2 | 182 |
| EncodeHaibTfbsH1hescSp4v20V0422111PkRep1 | 182 |
| EncodeHaibTfbsGm12878Bcl3V0416101PkRep2 | 182 |
| EncodeSydhTfbsGm12878Tr4StdPk | 183 |
| EncodeHaibTfbsH1hescGabpPcr1xPkRep2 | 183 |
| EncodeHaibTfbsH1hescPol2V0416102PkRep1 | 183 |
| EncodeSydhTfbsHelas3Tr4StdPk | 184 |
| EncodeHaibTfbsMcf7SrfV0422111PkRep2 | 184 |
| EncodeHaibTfbsA549GabpV0422111Etoh02PkRep1 | 185 |
| EncodeHaibTfbsMcf7Elf1V0422111PkRep1 | 185 |
| EncodeHaibTfbsU87Pol24h8V0416101PkRep1V2 | 185 |
| EncodeHaibTfbsGm12878Ets1Pcr1xPkRep2V2 | 186 |
| EncodeHaibTfbsGm12878Pou2f2Pcr1xPkRep3 | 186 |
| EncodeHaibTfbsK562Zbtb33Pcr1xPkRep2 | 186 |
| EncodeHaibTfbsK562Thap1sc98174V0416101PkRep2 | 186 |
| EncodeHaibTfbsHepg2Mbd4sc271530V0422111PkRep1 | 187 |
| EncodeSydhTfbsHct116Tcf7l2UcdPk | 187 |
| EncodeHaibTfbsEcc1MaxV0422111PkRep1 | 188 |
| EncodeHaibTfbsH1hescSrfPcr1xPkRep1 | 188 |
| EncodeHaibTfbsGm12892Pol24h8V0416102PkRep1 | 189 |
| EncodeHaibTfbsHct116SrfV0422111PkRep2 | 189 |
| EncodeHaibTfbsGm12878Ets1Pcr1xPkRep1 | 190 |
| EncodeHaibTfbsGm12878Ets1Pcr1xPkRep2 | 190 |
| EncodeHaibTfbsEcc1Yy1sc281V0422111PkRep2 | 191 |
| EncodeHaibTfbsGm12878Pol24h8Pcr1xPkRep2 | 193 |
| EncodeHaibTfbsGm12878Pol2Pcr2xPkRep2 | 193 |
| EncodeHaibTfbsHelas3Taf1Pcr1xPkRep2 | 193 |
| EncodeSydhTfbsK562NfyaStdPk | 193 |
| EncodeHaibTfbsH1hescSin3ak20Pcr1xPkRep2 | 194 |
| EncodeHaibTfbsH1hescSp2V0422111PkRep1 | 194 |
| EncodeSydhTfbsHepg2ErraForsklnStdPk | 194 |
| EncodeHaibTfbsGm12878SrfV0416101PkRep1 | 195 |
| EncodeHaibTfbsA549Ets1V0422111Etoh02PkRep2 | 195 |
| EncodeHaibTfbsGm12878Zbtb33Pcr1xPkRep1 | 195 |
| EncodeSydhTfbsHepg2Srebp2PravastStdPk | 195 |
| EncodeHaibTfbsHepg2Sp2V0422111PkRep2 | 196 |
| EncodeHaibTfbsSknshSin3ak20V0416101PkRep1 | 196 |
| EncodeHaibTfbsA549Ets1V0422111Etoh02PkRep1 | 196 |
| EncodeSydhTfbsK562Znf274m01UcdPk | 196 |
| EncodeHaibTfbsA549Atf3V0422111Etoh02PkRep2 | 197 |
| EncodeHaibTfbsA549Yy1cV0422111Etoh02PkRep1 | 197 |
| EncodeHaibTfbsMcf7Egr1V0422111PkRep2 | 197 |
| EncodeSydhTfbsH1hescCtbp2UcdPk | 197 |
| EncodeSydhTfbsHuvecMaxStdPk | 197 |
| EncodeSydhTfbsK562Irf1Ifng6hStdPk | 198 |
| EncodeSydhTfbsK562Xrcc4StdPk | 198 |
| EncodeHaibTfbsGm12878Egr1V0416101PkRep2 | 198 |
| EncodeHaibTfbsGm12878Zbtb33Pcr1xPkRep2 | 198 |
| EncodeHaibTfbsHct116Yy1sc281V0416101PkRep1 | 198 |
| EncodeHaibTfbsK562Sp2sc643V0416102PkRep1 | 198 |
| EncodeHaibTfbsH1hescE2f6V0422111PkRep1 | 199 |
| EncodeSydhTfbsK562Stat1Ifng30StdPk | 199 |
| EncodeHaibTfbsEcc1SrfV0422111PkRep2 | 199 |
| EncodeHaibTfbsGm12891Pol24h8Pcr1xPkRep2 | 199 |
| EncodeHaibTfbsGm12891Pol24h8Pcr1xPkRep1 | 200 |
| EncodeHaibTfbsK562Stat5asc74442V0422111PkRep1 | 200 |
| EncodeHaibTfbsEcc1Egr1V0422111PkRep2 | 201 |
| EncodeHaibTfbsHepg2Yy1sc281V0416101PkRep1 | 201 |
| EncodeSydhTfbsGm12878Srebp1IggrabPk | 201 |
| EncodeHaibTfbsK562Zbtb33Pcr1xPkRep1 | 202 |
| EncodeHaibTfbsSknmcPol24h8V0416101PkRep2 | 202 |
| EncodeSydhTfbsGm12878NfyaIggmusPk | 202 |
| EncodeHaibTfbsH1hescSp4v20V0422111PkRep2 | 203 |
| EncodeHaibTfbsK562Sp2sc643V0416102PkRep2 | 204 |
| EncodeHaibTfbsK562Pol2V0416101PkRep2 | 204 |
| EncodeHaibTfbsGm12878Six5Pcr1xPkRep1 | 205 |
| EncodeHaibTfbsGm12878Zeb1sc25388V0416102PkRep2 | 205 |
| EncodeHaibTfbsHct116Usf1V0422111PkRep1 | 205 |
| EncodeHaibTfbsHepg2Zbtb33Pcr1xPkRep2 | 205 |
| EncodeHaibTfbsGm12878Pol2Pcr2xPkRep1 | 205 |
| EncodeHaibTfbsHepg2Hey1V0416101PkRep1 | 205 |
| EncodeSydhTfbsK562NfybStdPk | 206 |
| EncodeHaibTfbsHepg2Sp2V0422111PkRep1 | 206 |
| EncodeHaibTfbsEcc1SrfV0422111PkRep1 | 207 |
| EncodeHaibTfbsGm12891Yy1sc281V0416101PkRep2 | 207 |
| EncodeHaibTfbsH1hescEgr1V0416102PkRep1 | 208 |
| EncodeHaibTfbsA549Elf1V0422111Etoh02PkRep2 | 208 |
| EncodeHaibTfbsEcc1Creb1sc240V0422111PkRep2 | 210 |
| EncodeHaibTfbsH1hescCreb1sc240V0422111PkRep1 | 210 |
| EncodeHaibTfbsGm12878Usf1Pcr2xPkRep2 | 210 |
| EncodeHaibTfbsSknshUsf1V0422111PkRep1 | 210 |
| EncodeHaibTfbsHelas3GabpPcr1xPkRep2 | 211 |
| EncodeHaibTfbsHepg2Elf1sc631V0416101PkRep1 | 211 |
| EncodeHaibTfbsK562Hey1Pcr1xPkRep1 | 211 |
| EncodeHaibTfbsH1hescEgr1V0416102PkRep2 | 212 |
| EncodeSydhTfbsGm12878NfkbTnfaIggrabPk | 212 |
| EncodeSydhTfbsHepg2Srebp1PravastStdPk | 212 |
| EncodeHaibTfbsA549JundV0416102Etoh02PkRep2 | 212 |
| EncodeHaibTfbsK562GabpV0416101PkRep1 | 212 |
| EncodeHaibTfbsSknshraYy1sc281V0416102PkRep1 | 212 |
| EncodeHaibTfbsH1hescGabpPcr1xPkRep1 | 213 |
| EncodeHaibTfbsHelas3Pol2Pcr1xPkRep2 | 214 |
| EncodeSydhTfbsHelas3Brf2StdPk | 214 |
| EncodeHaibTfbsGm12878Stat5asc74442V0422111PkRep2 | 214 |
| EncodeSydhTfbsK562MaxStdPk | 215 |
| EncodeHaibTfbsA549Six5V0422111Etoh02PkRep1 | 215 |
| EncodeSydhTfbsK562Tf3c110StdPk | 215 |
| EncodeHaibTfbsGm12878Pmlsc71910V0422111PkRep2 | 216 |
| EncodeHaibTfbsK562Mef2aV0416101PkRep2 | 216 |
| EncodeHaibTfbsK562Pol24h8V0416101PkRep1 | 216 |
| EncodeHaibTfbsK562Six5V0416101PkRep2 | 217 |
| EncodeHaibTfbsK562E2f6V0416102PkRep1 | 218 |
| EncodeHaibTfbsK562E2f6sc22823V0416102PkRep1 | 218 |
| EncodeSydhTfbsHelas3NfybIggrabPk | 218 |
| EncodeSydhTfbsGm12878Yy1StdPk | 219 |
| EncodeHaibTfbsGm12878Usf1Pcr2xPkRep1 | 220 |
| EncodeHaibTfbsH1hescYy1sc281V0416102PkRep2 | 220 |
| EncodeSydhTfbsHepg2TbpIggrabPk | 220 |
| EncodeHaibTfbsGm12878Nficsc81335V0422111PkRep2 | 221 |
| EncodeHaibTfbsGm12891Pax5c20V0416101PkRep1 | 221 |
| EncodeHaibTfbsH1hescSrfPcr1xPkRep2 | 221 |
| EncodeHaibTfbsK562Pol24h8V0416101PkRep2 | 221 |
| EncodeHaibTfbsGm12878Pol24h8Pcr1xPkRep1 | 222 |
| EncodeHaibTfbsGm12878Pou2f2Pcr1xPkRep1 | 222 |
| EncodeHaibTfbsHct116Zbtb33V0416101PkRep2 | 222 |
| EncodeHaibTfbsHepg2Zbtb33Pcr1xPkRep1 | 222 |
| EncodeSydhTfbsHelas3TbpIggrabPk | 222 |
| EncodeSydhTfbsK562Tr4UcdPk | 222 |
| EncodeHaibTfbsGm12892Pol2V0416102PkRep1 | 223 |
| EncodeHaibTfbsHepg2Cebpdsc636V0416101PkRep2 | 223 |
| EncodeHaibTfbsK562Ets1V0416101PkRep2 | 223 |
| EncodeSydhTfbsHepg2Hnf4aForsklnStdPk | 223 |
| EncodeHaibTfbsGm12878Egr1V0416101PkRep1 | 224 |
| EncodeSydhTfbsGm12878Spt20StdPk | 224 |
| EncodeHaibTfbsHepg2Pol24h8V0416102PkRep2 | 224 |
| EncodeHaibTfbsA549Usf1Pcr1xDex100nmPkRep2 | 225 |
| EncodeHaibTfbsHelas3GabpPcr1xPkRep1 | 225 |
| EncodeHaibTfbsK562SrfV0416101PkRep1 | 225 |
| EncodeHaibTfbsSknshSin3ak20V0416101PkRep2 | 225 |
| EncodeSydhTfbsK562CfosStdPk | 225 |
| EncodeHaibTfbsGm12878Pbx3Pcr1xPkRep1 | 226 |
| EncodeHaibTfbsSknshMaxV0422111PkRep1 | 226 |
| EncodeSydhTfbsGm12878Zzz3StdPk | 226 |
| EncodeSydhTfbsA549MaxIggrabPk | 226 |
| EncodeHaibTfbsK562Pol2V0416101PkRep1 | 227 |
| EncodeHaibTfbsK562Hey1Pcr1xPkRep2 | 228 |
| EncodeHaibTfbsSknshTaf1V0416101PkRep2 | 228 |
| EncodeHaibTfbsK562Elf1sc631V0416102PkRep1 | 229 |
| EncodeSydhTfbsHelas3Irf3IggrabPk | 229 |
| EncodeSydhTfbsHelas3NfyaIggrabPk | 229 |
| EncodeSydhTfbsK562Atf3StdPk | 229 |
| EncodeHaibTfbsA549Usf1V0422111Etoh02PkRep1 | 230 |
| EncodeHaibTfbsGm12878Atf3Pcr1xPkRep2 | 230 |
| EncodeHaibTfbsH1hescSp1Pcr1xPkRep2 | 231 |
| EncodeHaibTfbsSknshFoxm1sc502V0422111PkRep2 | 231 |
| EncodeHaibTfbsH1hescAtf3V0416102PkRep2 | 231 |
| EncodeHaibTfbsA549Tcf12V0422111Etoh02PkRep1 | 232 |
| EncodeHaibTfbsHct116Usf1V0422111PkRep2 | 232 |
| EncodeHaibTfbsK562Yy1V0416101PkRep1 | 232 |
| EncodeHaibTfbsK562Yy1sc281V0416101PkRep1 | 232 |
| EncodeHaibTfbsMcf7Hdac2sc6296V0422111PkRep1 | 232 |
| EncodeHaibTfbsHepg2Atf3V0416101PkRep2 | 233 |
| EncodeSydhTfbsA549Bhlhe40IggrabPk | 233 |
| EncodeHaibTfbsSknshUsf1V0422111PkRep2 | 234 |
| EncodeHaibTfbsSknshraYy1sc281V0416102PkRep2 | 234 |
| EncodeSydhTfbsHepg2CebpzIggrabPk | 234 |
| EncodeHaibTfbsHepg2Hey1V0416101PkRep2 | 235 |
| EncodeHaibTfbsH1hescSin3ak20Pcr1xPkRep1 | 235 |
| EncodeHaibTfbsK562Sp1Pcr1xPkRep1 | 236 |
| EncodeHaibTfbsEcc1Usf1V0422111PkRep1 | 236 |
| EncodeHaibTfbsSknmcPol24h8V0416101PkRep1 | 237 |
| EncodeSydhTfbsGm12878Irf3IggmusPk | 237 |
| EncodeSydhTfbsHelas3CmycStdPk | 237 |
| EncodeHaibTfbsSknshNrsfPcr2xPkRep2 | 238 |
| EncodeSydhTfbsK562Elk112771IggrabPk | 238 |
| EncodeHaibTfbsHepg2NrsfV0416101PkRep2 | 238 |
| EncodeHaibTfbsSknshPol24h8V0416101PkRep2 | 238 |
| EncodeHaibTfbsA549Usf1Pcr1xEtoh02PkRep1 | 240 |
| EncodeHaibTfbsHepg2Elf1sc631V0416101PkRep2 | 240 |
| EncodeSydhTfbsHepg2Irf3IggrabPk | 240 |
| EncodeHaibTfbsMcf7JundV0422111PkRep1 | 240 |
| EncodeHaibTfbsPfsk1Sin3ak20V0416101PkRep2 | 240 |
| EncodeSydhTfbsHepg2Hsf1ForsklnStdPk | 241 |
| EncodeHaibTfbsH1hescSp2V0422111PkRep2 | 241 |
| EncodeUchicagoTfbsK562Ehdac8ControlPk | 241 |
| EncodeHaibTfbsK562Cebpdsc636V0422111PkRep1 | 242 |
| EncodeSydhTfbsGm12878Nrf1IggmusPk | 242 |
| EncodeHaibTfbsGm12878Atf2sc81188V0422111PkRep2 | 242 |
| EncodeHaibTfbsGm12878SrfV0416101PkRep2 | 242 |
| EncodeSydhTfbsGm12878CfosStdPk | 242 |
| EncodeHaibTfbsHl60Pu1V0422111PkRep2 | 244 |
| EncodeHaibTfbsH1hescSp1Pcr1xPkRep1 | 245 |
| EncodeSydhTfbsK562Gtf2f1ab28179IggrabPk | 247 |
| EncodeHaibTfbsK562Mef2aV0416101PkRep1 | 248 |
| EncodeHaibTfbsSknshMaxV0422111PkRep2 | 249 |
| EncodeHaibTfbsEcc1Foxm1sc502V0422111PkRep2 | 250 |
| EncodeUchicagoTfbsK562Enr4a1ControlPk | 250 |
| EncodeHaibTfbsA549Creb1sc240V0416102Dex100nmPkRep2 | 250 |
| EncodeHaibTfbsHepg2SrfV0416101PkRep1 | 250 |
| EncodeSydhTfbsPbdeGata1UcdPk | 250 |
| EncodeHaibTfbsA549MaxV0422111PkRep2 | 251 |
| EncodeHaibTfbsH1hescPol24h8V0416102PkRep1 | 251 |
| EncodeHaibTfbsHelas3Pol2Pcr1xPkRep1 | 251 |
| EncodeHaibTfbsGm12891Pol2Pcr1xPkRep1 | 251 |
| EncodeHaibTfbsHl60Pol24h8V0422111PkRep2 | 251 |
| EncodeHaibTfbsEcc1Usf1V0422111PkRep2 | 252 |
| EncodeHaibTfbsH1hescP300V0416102PkRep2 | 252 |
| EncodeHaibTfbsGm12878Elf1sc631V0416101PkRep2 | 253 |
| EncodeHaibTfbsH1hescBcl11aV0416102PkRep2 | 253 |
| EncodeHaibTfbsK562Usf1V0416101PkRep2 | 253 |
| EncodeHaibTfbsK562Sp1Pcr1xPkRep2 | 254 |
| EncodeHaibTfbsH1hescSix5Pcr1xPkRep1 | 255 |
| EncodeSydhTfbsHelas3Zzz3StdPk | 255 |
| EncodeHaibTfbsGm12891Pax5c20V0416101PkRep2 | 256 |
| EncodeSydhTfbsGm12878Srebp2IggrabPk | 256 |
| EncodeSydhTfbsHepg2Brca1a300IggrabPk | 256 |
| EncodeSydhTfbsK562Usf2IggrabPk | 256 |
| EncodeHaibTfbsGm12878Tcf3Pcr1xPkRep2 | 256 |
| EncodeSydhTfbsK562CjunIggrabPk | 256 |
| EncodeHaibTfbsA549Usf1Pcr1xEtoh02PkRep2 | 257 |
| EncodeHaibTfbsHepg2SrfV0416101PkRep2 | 257 |
| EncodeHaibTfbsEcc1MaxV0422111PkRep2 | 259 |
| EncodeSydhTfbsHelas3Baf155IggmusPk | 259 |
| EncodeHaibTfbsA549NrsfV0422111Etoh02PkRep2 | 259 |
| EncodeHaibTfbsGm12878RxraPcr1xPkRep1 | 259 |
| EncodeSydhTfbsK562Irf1Ifna30StdPk | 260 |
| EncodeHaibTfbsPfsk1Pol24h8V0416101PkRep1 | 260 |
| EncodeSydhTfbsHek293Tcf7l2UcdPk | 261 |
| EncodeSydhTfbsHelas3Tcf7l2UcdPk | 261 |
| EncodeSydhTfbsNt2d1Suz12UcdPk | 261 |
| EncodeHaibTfbsHepg2Zbtb7aV0416101PkRep2 | 261 |
| EncodeHaibTfbsGm12878Yy1sc281Pcr1xPkRep2 | 262 |
| EncodeSydhTfbsHepg2Grp20ForsklnStdPk | 262 |
| EncodeSydhTfbsPbdefetalGata1UcdPk | 262 |
| EncodeHaibTfbsEcc1Foxm1sc502V0422111PkRep1 | 263 |
| EncodeHaibTfbsHct116Sp1V0422111PkRep1 | 263 |
| EncodeHaibTfbsHepg2Sin3ak20Pcr1xPkRep1 | 263 |
| EncodeSydhTfbsK562Gata1UcdPk | 265 |
| EncodeHaibTfbsGm12892Yy1V0416101PkRep1 | 265 |
| EncodeSydhTfbsGm12878Chd1a301218aIggmusPk | 265 |
| EncodeHaibTfbsMcf7Sin3ak20V0422111PkRep1 | 266 |
| EncodeHaibTfbsH1hescMaxV0422111PkRep1 | 267 |
| EncodeHaibTfbsA549Usf1Pcr1xDex100nmPkRep1 | 268 |
| EncodeHaibTfbsH1hescSix5Pcr1xPkRep2 | 269 |
| EncodeHaibTfbsGm12878P300Pcr1xPkRep1 | 271 |
| EncodeHaibTfbsA549Usf1V0422111Etoh02PkRep2 | 272 |
| EncodeHaibTfbsU87Pol24h8V0416101PkRep2V2 | 272 |
| EncodeHaibTfbsHct116Pol24h8V0416101PkRep1 | 272 |
| EncodeHaibTfbsGm12878Mef2aPcr1xPkRep1 | 273 |
| EncodeHaibTfbsH1hescHdac2sc6296V0416102PkRep2 | 273 |
| EncodeHaibTfbsSknshNrsfV0416101PkRep2 | 273 |
| EncodeHaibTfbsGm12878Mef2aPcr1xPkRep2 | 276 |
| EncodeHaibTfbsH1hescBcl11aPcr1xPkRep1 | 276 |
| EncodeHaibTfbsHepg2Atf3V0416101PkRep1 | 276 |
| EncodeHaibTfbsMcf7Foxm1sc502V0422111PkRep1 | 276 |
| EncodeHaibTfbsH1hescP300V0416102PkRep1 | 277 |
| EncodeSydhTfbsGm12878NfybIggmusPk | 277 |
| EncodeSydhTfbsK562Gata2UcdPk | 277 |
| EncodeSydhTfbsHelas3Ap2gammaStdPk | 278 |
| EncodeHaibTfbsGm12878Zeb1sc25388V0416102PkRep1 | 280 |
| EncodeSydhTfbsHepg2CebpbForsklnStdPk | 280 |
| EncodeHaibTfbsSknshraUsf1sc8983V0416102PkRep2 | 281 |
| EncodeHaibTfbsA549NrsfV0422111Etoh02PkRep1 | 283 |
| EncodeHaibTfbsHepg2Mybl2sc81192V0422111PkRep1 | 283 |
| EncodeHaibTfbsGm12878Bclaf101388V0416101PkRep1 | 284 |
| EncodeHaibTfbsHepg2Hnf4gsc6558V0416101PkRep2 | 284 |
| EncodeHaibTfbsK562Atf3V0416101PkRep1 | 285 |
| EncodeHaibTfbsEcc1Cebpbsc150V0422111PkRep1 | 285 |
| EncodeHaibTfbsPanc1NrsfV0416101PkRep2 | 286 |
| EncodeHaibTfbsK562Six5V0416101PkRep1 | 286 |
| EncodeHaibTfbsA549GrPcr2xDex100nmPkRep1 | 287 |
| EncodeHaibTfbsHepg2Pol2Pcr2xPkRep2 | 288 |
| EncodeSydhTfbsHelas3Elk112771IggrabPk | 288 |
| EncodeHaibTfbsSknshraUsf1sc8983V0416102PkRep1 | 289 |
| EncodeHaibTfbsEcc1Pol2V0416102Dm002p1hPkRep2 | 290 |
| EncodeSydhTfbsK562Setdb1MnasedUcdPk | 290 |
| EncodeSydhTfbsHelas3Stat1Ifng30StdPk | 291 |
| EncodeHaibTfbsH1hescJundV0416102PkRep1 | 291 |
| EncodeHaibTfbsA549Creb1sc240V0416102Dex100nmPkRep1 | 292 |
| EncodeHaibTfbsHct116JundV0422111PkRep1 | 292 |
| EncodeSydhTfbsGm08714Znf274UcdPk | 292 |
| EncodeHaibTfbsU87Pol24h8V0416101PkRep2 | 293 |
| EncodeHaibTfbsK562Hdac2sc6296V0416102PkRep2 | 294 |
| EncodeSydhTfbsHepg2Usf2IggrabPk | 294 |
| EncodeHaibTfbsHepg2Fosl2V0416101PkRep1 | 294 |
| EncodeHaibTfbsPfsk1Foxp2Pcr2xPkRep2 | 294 |
| EncodeSydhTfbsH1hescBrca1IggrabPk | 298 |
| EncodeHaibTfbsK562Stat5asc74442V0422111PkRep2 | 299 |
| EncodeHaibTfbsH1hescAtf3V0416102PkRep1 | 299 |
| EncodeHaibTfbsGm12892Pax5c20V0416101PkRep1 | 301 |
| EncodeHaibTfbsH1hescUsf1Pcr1xPkRep2 | 301 |
| EncodeHaibTfbsK562Six5Pcr1xPkRep2 | 301 |
| EncodeHaibTfbsPfsk1NrsfV0416101PkRep2 | 301 |
| EncodeHaibTfbsA549Atf3V0422111Etoh02PkRep1 | 301 |
| EncodeHaibTfbsSknshPol24h8V0416101PkRep1 | 302 |
| EncodeSydhTfbsHepg2Bhlhe40cIggrabPk | 302 |
| EncodeSydhTfbsGm12878MaxStdPk | 302 |
| EncodeSydhTfbsHelas3MaxStdPk | 303 |
| EncodeSydhTfbsHelas3Baf170IggmusPk | 304 |
| EncodeHaibTfbsH1hescJundV0416102PkRep2 | 304 |
| EncodeSydhTfbsGm12878Rfx5200401194IggmusPk | 304 |
| EncodeHaibTfbsEcc1P300V0422111PkRep1 | 305 |
| EncodeHaibTfbsMcf7MaxV0422111PkRep1 | 305 |
| EncodeSydhTfbsGm12878WhipIggmusPk | 306 |
| EncodeSydhTfbsHelas3Ap2alphaStdPk | 307 |
| EncodeHaibTfbsGm12878Cebpbsc150V0422111PkRep2 | 308 |
| EncodeHaibTfbsHepg2Pol24h8V0416102PkRep1 | 308 |
| EncodeHaibTfbsA549MaxV0422111PkRep1 | 309 |
| EncodeHaibTfbsGm12878Mta3sc81325V0422111PkRep2 | 309 |
| EncodeHaibTfbsHct116Pol24h8V0416101PkRep2 | 309 |
| EncodeHaibTfbsHct116Sp1V0422111PkRep2 | 310 |
| EncodeSydhTfbsSknshJundIggrabPk | 310 |
| EncodeSydhTfbsK562Kap1UcdPk | 311 |
| EncodeHaibTfbsGm12878Mef2csc13268V0416101PkRep2 | 311 |
| EncodeHaibTfbsGm12878Pbx3Pcr1xPkRep2 | 311 |
| EncodeHaibTfbsGm12878Runx3sc101553V0422111PkRep2 | 312 |
| EncodeHaibTfbsK562Trim28sc81411V0422111PkRep2 | 313 |
| EncodeSydhTfbsMcf10aesCmycEtoh01HvdPk | 313 |
| EncodeHaibTfbsHepg2Nficsc81335V0422111PkRep2 | 313 |
| EncodeSydhTfbsK562Nfe2StdPk | 313 |
| EncodeHaibTfbsGm12878Six5Pcr1xPkRep2 | 314 |
| EncodeHaibTfbsH1hescUsf1Pcr1xPkRep1 | 315 |
| EncodeHaibTfbsA549JundV0416102Etoh02PkRep1 | 316 |
| EncodeHaibTfbsA549Gata3V0422111PkRep2 | 316 |
| EncodeHaibTfbsGm12878P300Pcr1xPkRep2 | 317 |
| EncodeHaibTfbsH1hescPol2V0416102PkRep2 | 317 |
| EncodeHaibTfbsHepg2Zbtb7aV0416101PkRep1 | 317 |
| EncodeHaibTfbsHepg2Sin3ak20Pcr1xPkRep2 | 318 |
| EncodeHaibTfbsH1hescMaxV0422111PkRep2 | 318 |
| EncodeHaibTfbsH1hescYy1sc281V0416102PkRep1 | 318 |
| EncodeHaibTfbsPfsk1NrsfPcr2xPkRep2 | 319 |
| EncodeHaibTfbsGm12878Sp1Pcr1xPkRep1 | 320 |
| EncodeHaibTfbsK562MaxV0416102PkRep2 | 320 |
| EncodeSydhTfbsMcf10aesE2f4TamHvdPk | 320 |
| EncodeHaibTfbsHepg2Nficsc81335V0422111PkRep1 | 320 |
| EncodeHaibTfbsT47dP300V0416102Dm002p1hPkRep2 | 320 |
| EncodeHaibTfbsK562Six5Pcr1xPkRep1 | 321 |
| EncodeHaibTfbsA549Pol2Pcr2xEtoh02PkRep2 | 322 |
| EncodeSydhTfbsK562P300sc584sc48343IggrabPk | 322 |
| EncodeHaibTfbsGm12878Runx3sc101553V0422111PkRep1 | 323 |
| EncodeHaibTfbsGm12878Bcl11aPcr1xPkRep2 | 323 |
| EncodeHaibTfbsGm12878Pu1Pcr1xPkRep3 | 323 |
| EncodeHaibTfbsHuvecPol24h8V0416101PkRep2 | 325 |
| EncodeSydhTfbsHepg2Pol2IggrabPk | 326 |
| EncodeHaibTfbsHct116Yy1sc281V0416101PkRep2 | 328 |
| EncodeHaibTfbsHepg2Mybl2sc81192V0422111PkRep2 | 328 |
| EncodeHaibTfbsK562Usf1V0416101PkRep1 | 328 |
| EncodeSydhTfbsPanc1Tcf7l2UcdPk | 330 |
| EncodeHaibTfbsA549Pol2Pcr2xDex100nmPkRep2 | 331 |
| EncodeHaibTfbsGm12878Pax5n19Pcr1xPkRep1 | 331 |
| EncodeHaibTfbsK562SrfV0416101PkRep2 | 331 |
| EncodeSydhTfbsGm12878Nfe2sc22827StdPk | 332 |
| EncodeHaibTfbsHepg2Nr2f2sc271940V0422111PkRep1 | 333 |
| EncodeHaibTfbsK562Cbx3sc101004V0422111PkRep2 | 334 |
| EncodeHaibTfbsA549Pol2Pcr2xEtoh02PkRep1 | 336 |
| EncodeSydhTfbsH1hescTbpIggrabPk | 336 |
| EncodeSydhTfbsHepg2Chd2ab68301IggrabPk | 337 |
| EncodeHaibTfbsPfsk1NrsfV0416101PkRep1 | 338 |
| EncodeHaibTfbsHelas3NrsfPcr1xPkRep2 | 339 |
| EncodeHaibTfbsSknshJundV0422111PkRep2 | 340 |
| EncodeSydhTfbsGm12878Ikzf1iknuclaStdPk | 340 |
| EncodeSydhTfbsH1hescUsf2IggrabPk | 340 |
| EncodeSydhTfbsK562Mazab85725IggrabPk | 340 |
| EncodeSydhTfbsNt2d1Znf274UcdPk | 340 |
| EncodeHaibTfbsGm12878Pou2f2Pcr1xPkRep2 | 341 |
| EncodeSydhTfbsH1hescSuz12UcdPk | 343 |
| EncodeHaibTfbsGm12878RxraPcr1xPkRep2 | 344 |
| EncodeHaibTfbsSknshFoxm1sc502V0422111PkRep1 | 346 |
| EncodeHaibTfbsSknshJundV0422111PkRep1 | 347 |
| EncodeSydhTfbsHelas3Prdm19115IggrabPk | 348 |
| EncodeHaibTfbsMcf7Hdac2sc6296V0422111PkRep2 | 348 |
| EncodeHaibTfbsEcc1Cebpbsc150V0422111PkRep2 | 349 |
| EncodeSydhTfbsGm12878TbpIggmusPk | 349 |
| EncodeHaibTfbsHct116JundV0422111PkRep2 | 349 |
| EncodeHaibTfbsHct116Cbx3sc101004V0422111PkRep1 | 350 |
| EncodeHaibTfbsSknshPbx3V0422111PkRep2 | 351 |
| EncodeHaibTfbsGm12878Atf3Pcr1xPkRep1 | 351 |
| EncodeHaibTfbsGm12878Irf4sc6059Pcr1xPkRep1 | 352 |
| EncodeSydhTfbsHelas3Tf3c110StdPk | 352 |
| EncodeSydhTfbsK562Corestsc30189IggrabPk | 352 |
| EncodeHaibTfbsSknshTead4sc101184V0422111PkRep1 | 353 |
| EncodeHaibTfbsA549Sp1V0422111Etoh02PkRep2 | 353 |
| EncodeHaibTfbsGm12878Pu1Pcr1xPkRep2 | 356 |
| EncodeHaibTfbsHct116Cebpbsc150V0422111PkRep1 | 356 |
| EncodeHaibTfbsHepg2Tcf12Pcr1xPkRep1 | 359 |
| EncodeHaibTfbsH1hescFosl1sc183V0416102PkRep2 | 361 |
| EncodeHaibTfbsGm12878Tcf12Pcr1xPkRep2 | 361 |
| EncodeHaibTfbsGm12878Mef2csc13268V0416101PkRep1 | 362 |
| EncodeSydhTfbsGm12878Ebf1sc137065StdPk | 362 |
| EncodeSydhTfbsHepg2Mxi1StdPk | 362 |
| EncodeHaibTfbsHepg2Bhlhe40V0416101PkRep1 | 364 |
| EncodeHaibTfbsT47dJundV0422111PkRep2 | 364 |
| EncodeHaibTfbsA549P300V0422111Etoh02PkRep2 | 365 |
| EncodeSydhTfbsK562CmycIggrabPk | 365 |
| EncodeSydhTfbsImr90Corestsc30189IggrabPk | 366 |
| EncodeSydhTfbsNb4MaxStdPk | 366 |
| EncodeSydhTfbsK562CjunStdPk | 366 |
| EncodeHaibTfbsEcc1NrsfV0422111PkRep1 | 367 |
| EncodeHaibTfbsK562Hdac2sc6296V0416102PkRep1 | 368 |
| EncodeSydhTfbsSknshP300bIggrabPk | 369 |
| EncodeHaibTfbsH1neuronsPol24h8V0422111PkRep1 | 370 |
| EncodeSydhTfbsGm12878Elk112771IggmusPk | 370 |
| EncodeHaibTfbsGm12878Ebfsc137065Pcr1xPkRep1 | 372 |
| EncodeHaibTfbsK562Egr1V0416101PkRep2 | 372 |
| EncodeHaibTfbsK562Yy1V0416102PkRep2 | 372 |
| EncodeHaibTfbsGm12878Atf2sc81188V0422111PkRep1 | 373 |
| EncodeSydhTfbsMcf10aesCmycTam14hHvdPk | 373 |
| EncodeHaibTfbsH1neuronsPol24h8V0422111PkRep2 | 374 |
| EncodeSydhTfbsHelas3Mxi1af4185IggrabPk | 374 |
| EncodeHaibTfbsGm12878Sp1Pcr1xPkRep2 | 375 |
| EncodeHaibTfbsHepg2Hnf4gsc6558V0416101PkRep1 | 376 |
| EncodeHaibTfbsT47dEralphaaV0416102Est10nm1hPkRep2 | 377 |
| EncodeSydhTfbsK562Pol2s2StdPk | 377 |
| EncodeHaibTfbsEcc1EraaV0416102Bpa1hPkRep2 | 380 |
| EncodeHaibTfbsHepg2Usf1Pcr1xPkRep1 | 380 |
| EncodeHaibTfbsA549P300V0422111Etoh02PkRep1 | 380 |
| EncodeSydhTfbsGm12878Usf2IggmusPk | 380 |
| EncodeHaibTfbsEcc1EralphaaV0416102Est10nm1hPkRep1 | 381 |
| EncodeHaibTfbsK562Nr2f2sc271940V0422111PkRep2 | 382 |
| EncodeHaibTfbsSknmcFoxp2Pcr2xPkRep2 | 382 |
| EncodeHaibTfbsSknshNrsfV0416101PkRep1 | 382 |
| EncodeHaibTfbsEcc1Nficsc81335V0422111PkRep1 | 382 |
| EncodeSydhTfbsK562JundIggrabPk | 384 |
| EncodeHaibTfbsGm12878Irf4sc6059Pcr1xPkRep2 | 384 |
| EncodeHaibTfbsGm12878Pax5n19Pcr1xPkRep2 | 384 |
| EncodeSydhTfbsK562Brg1IggmusPk | 384 |
| EncodeSydhTfbsK562Gata1bIggmusPk | 385 |
| EncodeHaibTfbsGm12878Ebfsc137065Pcr1xPkRep2 | 385 |
| EncodeHaibTfbsGm12878Tcf3Pcr1xPkRep1 | 386 |
| EncodeSydhTfbsGm12878Stat3IggmusPk | 387 |
| EncodeSydhTfbsMcf10aesStat3Etoh01StdPk | 387 |
| EncodeSydhTfbsK562Bach1sc14700IggrabPk | 389 |
| EncodeHaibTfbsA549Gata3V0422111PkRep1 | 391 |
| EncodeHaibTfbsMcf7NrsfV0422111PkRep1 | 392 |
| EncodeHaibTfbsGm12878Bcl11aPcr1xPkRep1 | 392 |
| EncodeHaibTfbsT47dEralphaaPcr2xGen1hPkRep1 | 393 |
| EncodeHaibTfbsK562Tead4sc101184V0422111PkRep1 | 393 |
| EncodeSydhTfbsK562Hmgn3StdPk | 394 |
| EncodeSydhTfbsK562Ubfsc13125IggmusPk | 394 |
| EncodeHaibTfbsH1hescFosl1sc183V0416102PkRep1 | 394 |
| EncodeHaibTfbsT47dEraaV0416102Bpa1hPkRep2 | 395 |
| EncodeSydhTfbsK562Chd2ab68301IggrabPk | 395 |
| EncodeSydhTfbsK562Mxi1af4185IggrabPk | 395 |
| EncodeSydhTfbsK562Ccnt2StdPk | 396 |
| EncodeSydhTfbsK562Bhlhe40nb100IggrabPk | 397 |
| EncodeHaibTfbsEcc1Tead4sc101184V0422111PkRep1 | 397 |
| EncodeHaibTfbsK562NrsfV0416102PkRep1 | 398 |
| EncodeSydhTfbsHelas3CebpbIggrabPk | 398 |
| EncodeHaibTfbsGm12891Pu1Pcr1xPkRep2 | 398 |
| EncodeSydhTfbsH1hescChd2IggrabPk | 400 |
| EncodeSydhTfbsHelas3Chd2IggrabPk | 400 |
| EncodeSydhTfbsK562CebpbIggrabPk | 400 |
| EncodeHaibTfbsMcf7Tcf12V0422111PkRep1 | 401 |
| EncodeSydhTfbsK562CmycIfng30StdPk | 401 |
| EncodeHaibTfbsHepg2Hdac2sc6296V0416101PkRep2 | 402 |
| EncodeHaibTfbsGm12878Pax5c20Pcr1xPkRep2 | 403 |
| EncodeHaibTfbsK562Zbtb7asc34508V0416101PkRep2 | 403 |
| EncodeSydhTfbsHek293tZnf263UcdPk | 403 |
| EncodeHaibTfbsEcc1EralphaaV0416102Est10nm1hPkRep2 | 404 |
| EncodeHaibTfbsGm12878NrsfPcr1xPkRep1 | 404 |
| EncodeSydhTfbsK562Rpc155StdPk | 404 |
| EncodeHaibTfbsEcc1GrV0416102Dex100nmPkRep2 | 405 |
| EncodeHaibTfbsHepg2Tcf12Pcr1xPkRep2 | 407 |
| EncodeUchicagoTfbsK562EjundControlPk | 408 |
| EncodeHaibTfbsGm12878Nficsc81335V0422111PkRep1 | 409 |
| EncodeHaibTfbsU87NrsfPcr2xPkRep2V2 | 409 |
| EncodeSydhTfbsMcf7Tcf7l2UcdPk | 409 |
| EncodeHaibTfbsT47dP300V0416102Dm002p1hPkRep1 | 410 |
| EncodeSydhTfbsK562TbpIggmusPk | 410 |
| EncodeHaibTfbsK562E2f6V0416102PkRep2 | 411 |
| EncodeHaibTfbsK562E2f6sc22823V0416102PkRep2 | 411 |
| EncodeHaibTfbsGm12878BatfPcr1xPkRep2 | 411 |
| EncodeHaibTfbsHepg2Hnf4asc8987V0416101PkRep1 | 411 |
| EncodeHaibTfbsGm12878Yy1sc281Pcr1xPkRep1 | 412 |
| EncodeHaibTfbsK562Elf1sc631V0416102PkRep2 | 412 |
| EncodeSydhTfbsGm12878Mxi1IggmusPk | 413 |
| EncodeHaibTfbsHepg2Hnf4asc8987V0416101PkRep2 | 414 |
| EncodeSydhTfbsGm12878MaxIggmusPk | 415 |
| EncodeHaibTfbsSknshPbx3V0422111PkRep1 | 416 |
| EncodeSydhTfbsNb4CmycStdPk | 417 |
| EncodeHaibTfbsK562Egr1V0416101PkRep1 | 418 |
| EncodeHaibTfbsEcc1EralphaaV0416102Gen1hPkRep1 | 418 |
| EncodeSydhTfbsHelas3Brca1a300IggrabPk | 418 |
| EncodeSydhTfbsHelas3MaxIggrabPk | 421 |
| EncodeHaibTfbsT47dEralphaaPcr2xGen1hPkRep2 | 422 |
| EncodeHaibTfbsK562Atf3V0416101PkRep2 | 423 |
| EncodeSydhTfbsK562Ubtfsab1404509IggmusPk | 424 |
| EncodeHaibTfbsHepg2RxraPcr1xPkRep1 | 424 |
| EncodeHaibTfbsK562Fosl1sc183V0416101PkRep1 | 425 |
| EncodeSydhTfbsHepg2MaxIggrabPk | 425 |
| EncodeHaibTfbsGm12878Pax5c20Pcr1xPkRep1 | 425 |
| EncodeSydhTfbsK562Irf1Ifng30StdPk | 425 |
| EncodeSydhTfbsK562Znf384hpa004051IggrabPk | 425 |
| EncodeHaibTfbsSknmcFoxp2Pcr2xPkRep1 | 427 |
| EncodeSydhTfbsHepg2Mazab85725IggrabPk | 427 |
| EncodeHaibTfbsGm12878NrsfPcr1xPkRep2 | 429 |
| EncodeSydhTfbsGm12878Chd2ab68301IggmusPk | 429 |
| EncodeHaibTfbsHepg2Sp1Pcr1xPkRep2 | 429 |
| EncodeSydhTfbsK562Irf1Ifna6hStdPk | 429 |
| EncodeHaibTfbsHepg2Sp1Pcr1xPkRep1 | 430 |
| EncodeSydhTfbsK562Hcfc1nb10068209IggrabPk | 430 |
| EncodeHaibTfbsHepg2Cebpbsc150V0416101PkRep1 | 431 |
| EncodeHaibTfbsSknshP300V0422111PkRep1 | 431 |
| EncodeSydhTfbsGm12878Mazab85725IggmusPk | 432 |
| EncodeHaibTfbsHepg2JundPcr1xPkRep2 | 433 |
| EncodeHaibTfbsMcf7Foxm1sc502V0422111PkRep2 | 433 |
| EncodeSydhTfbsK562Znf274UcdPk | 434 |
| EncodeHaibTfbsHepg2Bhlhe40V0416101PkRep2 | 434 |
| EncodeHaibTfbsT47dEralphaaV0416102Est10nm1hPkRep1 | 435 |
| EncodeSydhTfbsHepg2CebpbIggrabPk | 435 |
| EncodeHaibTfbsEcc1EraaV0416102Bpa1hPkRep1 | 435 |
| EncodeHaibTfbsK562Cebpbsc150V0422111PkRep2 | 436 |
| EncodeSydhTfbsHelas3Mazab85725IggrabPk | 436 |
| EncodeHaibTfbsT47dJundV0422111PkRep1 | 438 |
| EncodeHaibTfbsA549GrPcr2xDex100nmPkRep2 | 438 |
| EncodeSydhTfbsH1hescSin3anb6001263IggrabPk | 438 |
| EncodeSydhTfbsMcf10aesStat3TamStdPk | 438 |
| EncodeHaibTfbsHl60Pu1V0422111PkRep1 | 439 |
| EncodeHaibTfbsMcf7Nr2f2sc271940V0422111PkRep2 | 439 |
| EncodeHaibTfbsGm12878Tcf12Pcr1xPkRep1 | 440 |
| EncodeSydhTfbsHelas3Spt20StdPk | 440 |
| EncodeHaibTfbsHct116Cebpbsc150V0422111PkRep2 | 441 |
| EncodeHaibTfbsU87NrsfPcr2xPkRep2 | 441 |
| EncodeHaibTfbsGm12878Foxm1sc502V0422111PkRep1 | 441 |
| EncodeSydhTfbsH1hescMxi1IggrabPk | 443 |
| EncodeSydhTfbsK562Tblr1nb600270IggrabPk | 443 |
| EncodeHaibTfbsHepg2JundPcr1xPkRep1 | 444 |
| EncodeSydhTfbsK562Sirt6StdPk | 444 |
| EncodeSydhTfbsH1hescBach1sc14700IggrabPk | 445 |
| EncodeSydhTfbsHelas3Brg1IggmusPk | 445 |
| EncodeSydhTfbsK562P300IggrabPk | 445 |
| EncodeHaibTfbsA549GrPcr1xDex500pmPkRep1 | 445 |
| EncodeHaibTfbsEcc1EralphaaV0416102Gen1hPkRep2 | 447 |
| EncodeSydhTfbsH1hescCjunIggrabPk | 447 |
| EncodeHaibTfbsHepg2Tead4sc101184V0422111PkRep1 | 447 |
| EncodeHaibTfbsMcf7P300V0422111PkRep1 | 447 |
| EncodeHaibTfbsT47dEraaV0416102Bpa1hPkRep1 | 447 |
| EncodeHaibTfbsA549Sp1V0422111Etoh02PkRep1 | 448 |
| EncodeSydhTfbsImr90CebpbIggrabPk | 448 |
| EncodeHaibTfbsEcc1Tcf12V0422111PkRep2 | 448 |
| EncodeHaibTfbsK562MaxV0416102PkRep1 | 449 |
| EncodeHaibTfbsA549Cebpbsc150V0422111PkRep2 | 450 |
| EncodeHaibTfbsK562Cbx3sc101004V0422111PkRep1 | 451 |
| EncodeHaibTfbsK562Tead4sc101184V0422111PkRep2 | 453 |
| EncodeSydhTfbsGm12878Bhlhe40cIggmusPk | 453 |
| EncodeSydhTfbsHelas3Rpc155StdPk | 453 |
| EncodeSydhTfbsK562Bdp1StdPk | 453 |
| EncodeHaibTfbsEcc1Nficsc81335V0422111PkRep2 | 454 |
| EncodeHaibTfbsEcc1P300V0422111PkRep2 | 455 |
| EncodeSydhTfbsGm12878Stat1StdPk | 455 |
| EncodeHaibTfbsHepg2Usf1Pcr1xPkRep2 | 455 |
| EncodeHaibTfbsK562Nr2f2sc271940V0422111PkRep1 | 457 |
| EncodeSydhTfbsGm12878P300sc584IggmusPk | 458 |
| EncodeSydhTfbsGm12878Znf143166181apStdPk | 459 |
| EncodeHaibTfbsH1hescTead4sc101184V0422111PkRep2 | 459 |
| EncodeHaibTfbsEcc1Tead4sc101184V0422111PkRep2 | 460 |
| EncodeSydhTfbsGm12878Pol3StdPk | 461 |
| EncodeHaibTfbsMcf7Tead4sc101184V0422111PkRep2 | 461 |
| EncodeSydhTfbsK562CjunIfng30StdPk | 462 |
| EncodeSydhTfbsH1hescGtf2f1IggrabPk | 464 |
| EncodeHaibTfbsSknshMef2aV0422111PkRep1 | 465 |
| EncodeSydhTfbsGm12878Corestsc30189IggmusPk | 465 |
| EncodeSydhTfbsHepg2Rfx5200401194IggrabPk | 465 |
| EncodeSydhTfbsSknshSmc3IggrabPk | 465 |
| EncodeHaibTfbsEcc1Tcf12V0422111PkRep1 | 465 |
| EncodeSydhTfbsGm12878Tblr1ab24550IggmusPk | 465 |
| EncodeHaibTfbsMcf7Nr2f2sc271940V0422111PkRep1 | 466 |
| EncodeSydhTfbsK562Znfmizdcp1ab65767IggrabPk | 467 |
| EncodeHaibTfbsK562Pu1Pcr1xPkRep2 | 467 |
| EncodeHaibTfbsSknshRxraV0422111PkRep1 | 467 |
| EncodeHaibTfbsK562Cebpbsc150V0422111PkRep1 | 468 |
| EncodeSydhTfbsHepg2P300sc582IggrabPk | 468 |
| EncodeHaibTfbsK562Ctcflsc98982V0416101PkRep1 | 469 |
| EncodeHaibTfbsH1hescHdac2sc6296V0416102PkRep1 | 470 |
| EncodeSydhTfbsGm12878Znf274StdPk | 470 |
| EncodeHaibTfbsMcf7JundV0422111PkRep2 | 471 |
| EncodeSydhTfbsHelas3Bdp1StdPk | 471 |
| EncodeSydhTfbsHelas3Zkscan1hpa006672IggrabPk | 471 |
| EncodeSydhTfbsGm12878JundStdPk | 471 |
| EncodeHaibTfbsHct116Atf3V0422111PkRep1 | 474 |
| EncodeSydhTfbsGm12878P300bStdPk | 474 |
| EncodeSydhTfbsSknshCtcfbIggrabPk | 474 |
| EncodeHaibTfbsSknshTead4sc101184V0422111PkRep2 | 475 |
| EncodeHaibTfbsGm12891Pu1Pcr1xPkRep1 | 476 |
| EncodeHaibTfbsMcf7Tcf12V0422111PkRep2 | 476 |
| EncodeHaibTfbsMcf7Gata3V0422111PkRep2 | 477 |
| EncodeHaibTfbsEcc1NrsfV0422111PkRep2 | 479 |
| EncodeSydhTfbsK562CjunIfna6hStdPk | 479 |
| EncodeHaibTfbsA549GrPcr1xDex5nmPkRep2 | 482 |
| EncodeHaibTfbsGm12878Pu1Pcr1xPkRep1 | 482 |
| EncodeSydhTfbsHelas3Gtf2f1ab28179IggrabPk | 482 |
| EncodeHaibTfbsMcf7P300V0422111PkRep2 | 483 |
| EncodeSydhTfbsK562Tblr1ab24550IggrabPk | 483 |
| EncodeSydhTfbsHuvecCjunStdPk | 483 |
| EncodeSydhTfbsK562MaxIggrabPk | 484 |
| EncodeSydhTfbsK562Corestab24166IggrabPk | 484 |
| EncodeHaibTfbsA549Cebpbsc150V0422111PkRep1 | 485 |
| EncodeHaibTfbsK562Gata2sc267Pcr1xPkRep2 | 486 |
| EncodeHaibTfbsHepg2P300V0416101PkRep2 | 486 |
| EncodeHaibTfbsA549Foxa1V0416102Dex100nmPkRep1 | 488 |
| EncodeSydhTfbsSknshRad21IggrabPk | 492 |
| EncodeHaibTfbsGm12878BatfPcr1xPkRep1 | 493 |
| EncodeHaibTfbsH1hescPou5f1sc9081V0416102PkRep2 | 493 |
| EncodeSydhTfbsH1hescCmycIggrabPk | 493 |
| EncodeHaibTfbsA549Tcf12V0422111Etoh02PkRep2 | 494 |
| EncodeHaibTfbsSknshRxraV0422111PkRep2 | 494 |
| EncodeSydhTfbsHelas3CjunIggrabPk | 494 |
| EncodeSydhTfbsHepg2Tcf7l2UcdPk | 494 |
| EncodeHaibTfbsMcf7Cebpbsc150V0422111PkRep2 | 496 |
| EncodeHaibTfbsH1hescNanogsc33759V0416102PkRep2 | 496 |
| EncodeHaibTfbsA549GrPcr1xDex50nmPkRep1 | 497 |
| EncodeHaibTfbsPanc1NrsfV0422111PkRep2 | 499 |
| EncodeHaibTfbsA549GrPcr1xDex50nmPkRep2 | 499 |
| EncodeSydhTfbsHepg2Corestsc30189IggrabPk | 500 |
| EncodeSydhTfbsHek293Kap1UcdPk | 500 |
| EncodeSydhTfbsHelas3Corestsc30189IggrabPk | 500 |
| EncodeSydhTfbsHelas3Brf1StdPk | 501 |
| EncodeHaibTfbsHepg2Zeb1V0422111PkRep2 | 501 |
| EncodeHaibTfbsA549GrPcr1xDex5nmPkRep1 | 502 |
| EncodeSydhTfbsGm12878P300IggmusPk | 502 |
| EncodeSydhTfbsK562Rfx5IggrabPk | 503 |
| EncodeHaibTfbsSknshP300V0422111PkRep2 | 503 |
| EncodeHaibTfbsHelas3NrsfPcr1xPkRep1 | 504 |
| EncodeSydhTfbsMcf10aesStat3Etoh01cStdPk | 505 |
| EncodeHaibTfbsK562Ctcflsc98982V0416101PkRep2 | 508 |
| EncodeSydhTfbsMcf10aesStat3Tam112hHvdPk | 509 |
| EncodeHaibTfbsHepg2Cebpbsc150V0416101PkRep2 | 510 |
| EncodeHaibTfbsEcc1GrV0416102Dex100nmPkRep1 | 511 |
| EncodeHaibTfbsGm12878NrsfPcr2xPkRep1 | 511 |
| EncodeSydhTfbsK562CjunIfng6hStdPk | 514 |
| EncodeHaibTfbsA549Foxa1V0416102Dex100nmPkRep2 | 515 |
| EncodeSydhTfbsK562Ini1IggmusPk | 515 |
| EncodeHaibTfbsSknshNficsc81335V0422111PkRep2 | 515 |
| EncodeSydhTfbsH1hescCebpbIggrabPk | 516 |
| EncodeHaibTfbsEcc1Foxa1sc6553V0416102Dm002p1hPkRep2 | 517 |
| EncodeSydhTfbsHelas3Rfx5200401194IggrabPk | 517 |
| EncodeSydhTfbsH1hescJundIggrabPk | 519 |
| EncodeHaibTfbsHepg2Foxa2sc6554V0416101PkRep1 | 520 |
| EncodeHaibTfbsA549Tead4sc101184V0422111PkRep1 | 522 |
| EncodeHaibTfbsHepg2Hdac2sc6296V0416101PkRep1 | 522 |
| EncodeHaibTfbsSknshMef2aV0422111PkRep2 | 522 |
| EncodeSydhTfbsHelas3Stat3IggrabPk | 522 |
| EncodeSydhTfbsHelas3MafkIggrabPk | 523 |
| EncodeSydhTfbsMcf10aesCfosTamHvdPk | 524 |
| EncodeSydhTfbsHelas3CfosStdPk | 524 |
| EncodeHaibTfbsMcf7Fosl2V0422111PkRep2 | 526 |
| EncodeSydhTfbsK562Mafkab50322IggrabPk | 526 |
| EncodeSydhTfbsHelas3Tcf7l2c9b92565UcdPk | 528 |
| EncodeHaibTfbsK562Gata2sc267Pcr1xPkRep1 | 529 |
| EncodeHaibTfbsHepg2NrsfV0416101PkRep1 | 530 |
| EncodeHaibTfbsSknshNficsc81335V0422111PkRep1 | 530 |
| EncodeUchicagoTfbsK562EfosControlPk | 531 |
| EncodeSydhTfbsMcf7Gata3sc269UcdPk | 535 |
| EncodeHaibTfbsA549Tead4sc101184V0422111PkRep2 | 537 |
| EncodeSydhTfbsA549CebpbIggrabPk | 537 |
| EncodeSydhTfbsH1hescZnf274m01UcdPk | 537 |
| EncodeSydhTfbsHelas3Znf143IggrabPk | 537 |
| EncodeHaibTfbsHct116Fosl1V0422111PkRep1 | 538 |
| EncodeSydhTfbsHepg2Mafksc477IggrabPk | 538 |
| EncodeSydhTfbsK562Znf143IggrabPk | 538 |
| EncodeSydhTfbsMcf10aesStat3Etoh01bStdPk | 539 |
| EncodeHaibTfbsK562Fosl1sc183V0416101PkRep2 | 541 |
| EncodeSydhTfbsK562Zc3h11anb10074650IggrabPk | 544 |
| EncodeSydhTfbsU2osKap1UcdPk | 544 |
| EncodeSydhTfbsK562Brf1StdPk | 545 |
| EncodeHaibTfbsEcc1Foxa1sc6553V0416102Dm002p1hPkRep1 | 546 |
| EncodeHaibTfbsHct116CtcfcV0422111PkRep1 | 549 |
| EncodeSydhTfbsH1hescZnf143IggrabPk | 549 |
| EncodeHaibTfbsK562Pu1Pcr1xPkRep1 | 550 |
| EncodeHaibTfbsHepg2NrsfPcr2xPkRep2 | 550 |
| EncodeHaibTfbsMcf7Fosl2V0422111PkRep1 | 551 |
| EncodeSydhTfbsK562Tal1sc12984IggmusPk | 551 |
| EncodeHaibTfbsHepg2Tead4sc101184V0422111PkRep2 | 553 |
| EncodeHaibTfbsT47dCtcfsc5916V0416102Dm002p1hPkRep1 | 556 |
| EncodeRegTfbsClusteredV2 | 558 |
| EncodeRegTfbsClustered | 559 |
| EncodeSydhTfbsHelas3Smc3ab9263IggrabPk | 560 |
| EncodeSydhTfbsGm12878Znf384hpa004051IggmusPk | 560 |
| EncodeUchicagoTfbsK562Egata2ControlPk | 560 |
| EncodeHaibTfbsGm12878NrsfPcr2xPkRep2 | 562 |
| EncodeSydhTfbsA549CtcfbIggrabPk | 562 |
| EncodeHaibTfbsHepg2RxraPcr1xPkRep2 | 563 |
| EncodeSydhTfbsMcf10aesCfosEtoh01HvdPk | 564 |
| EncodeSydhTfbsK562Rad21StdPk | 565 |
| EncodeHaibTfbsHepg2Foxa1sc101058V0416101PkRep1 | 566 |
| EncodeHaibTfbsHepg2Nr2f2sc271940V0422111PkRep2 | 568 |
| EncodeHaibTfbsK562NrsfV0416102PkRep2 | 569 |
| EncodeSydhTfbsGm12878Rad21IggrabPk | 569 |
| EncodeSydhTfbsK562Atf106325StdPk | 569 |
| EncodeHaibTfbsT47dCtcfsc5916V0416102Dm002p1hPkRep2 | 570 |
| EncodeSydhTfbsHepg2Mafkab50322IggrabPk | 570 |
| EncodeSydhTfbsHelas3Usf2IggmusPk | 572 |
| EncodeHaibTfbsPanc1NrsfPcr2xPkRep1 | 572 |
| EncodeSydhTfbsMcf7Znf217UcdPk | 574 |
| EncodeSydhTfbsGm12878Cdpsc6327IggmusPk | 576 |
| EncodeHaibTfbsMcf7NrsfV0422111PkRep2 | 577 |
| EncodeHaibTfbsEcc1CtcfcV0416102Dm002p1hPkRep2 | 578 |
| EncodeSydhTfbsK562CjunIfna30StdPk | 578 |
| EncodeSydhTfbsHepg2CjunIggrabPk | 579 |
| EncodeHaibTfbsA549Foxa2V0416102Etoh02PkRep1 | 580 |
| EncodeHaibTfbsA549Fosl2V0422111Etoh02PkRep2 | 582 |
| EncodeSydhTfbsImr90MafkIggrabPk | 582 |
| EncodeSydhTfbsHuvecCfosUcdPk | 583 |
| EncodeSydhTfbsHepg2Arid3anb100279IggrabPk | 583 |
| EncodeHaibTfbsPanc1NrsfPcr2xPkRep2 | 586 |
| EncodeSydhTfbsU2osSetdb1UcdPk | 586 |
| EncodeSydhTfbsHuvecGata2UcdPk | 587 |
| EncodeUchicagoTfbsK562EjunbControlPk | 594 |
| EncodeHaibTfbsHct116CtcfcV0422111PkRep2 | 594 |
| EncodeHaibTfbsSknshTcf12V0422111PkRep1 | 595 |
| EncodeSydhTfbsH1hescMafkIggrabPk | 595 |
| EncodeHaibTfbsHct116Fosl1V0422111PkRep2 | 596 |
| EncodeHaibTfbsHct116Rad21V0422111PkRep1 | 597 |
| EncodeSydhTfbsHelas3JundIggrabPk | 598 |
| EncodeHaibTfbsU87NrsfPcr2xPkRep1V2 | 599 |
| EncodeHaibTfbsA549Ctcfsc5916Pcr1xDex100nmPkRep2 | 602 |
| EncodeSydhTfbsHepg2JundIggrabPk | 602 |
| EncodeSydhTfbsHepg2Maffm8194IggrabPk | 603 |
| EncodeHaibTfbsH1hescNrsfV0416102PkRep2 | 605 |
| EncodeHaibTfbsA549Fosl2V0422111Etoh02PkRep1 | 609 |
| EncodeHaibTfbsH1hescTcf12Pcr1xPkRep2 | 609 |
| EncodeHaibTfbsU87NrsfPcr2xPkRep1 | 610 |
| EncodeSydhTfbsMcf10aesCfosTam112hHvdPk | 610 |
| EncodeUwTfbsGm12801CtcfStdPkRep1 | 610 |
| EncodeHaibTfbsHepg2NrsfPcr2xPkRep1 | 614 |
| EncodeHaibTfbsHepg2Foxa1sc101058V0416101PkRep2 | 615 |
| EncodeHaibTfbsSknshFosl2V0422111PkRep2 | 616 |
| EncodeHaibTfbsH1hescNrsfV0416102PkRep1 | 617 |
| EncodeSydhTfbsHelas3P300sc584sc584IggrabPk | 617 |
| EncodeHaibTfbsHepg2Fosl2V0416101PkRep2 | 618 |
| EncodeHaibTfbsSknshGata3V0422111PkRep1 | 620 |
| EncodeHaibTfbsSknshTcf12V0422111PkRep2 | 621 |
| EncodeHaibTfbsSknshFosl2V0422111PkRep1 | 621 |
| EncodeHaibTfbsEcc1CtcfcV0416102Dm002p1hPkRep1 | 622 |
| EncodeHaibTfbsMcf7Gata3V0422111PkRep1 | 627 |
| EncodeHaibTfbsT47dGata3sc268V0416102Dm002p1hPkRep1 | 632 |
| EncodeHaibTfbsA549Ctcfsc5916Pcr1xEtoh02PkRep1 | 633 |
| EncodeHaibTfbsMcf7Cebpbsc150V0422111PkRep1 | 634 |
| EncodeHaibTfbsHepg2P300V0416101PkRep1 | 634 |
| EncodeSydhTfbsK562Cdpsc6327IggrabPk | 635 |
| EncodeSydhTfbsGm12878Ctcfsc15914c20StdPk | 636 |
| EncodeHaibTfbsHct116Atf3V0422111PkRep2 | 639 |
| EncodeSydhTfbsH1hescMaxUcdPk | 639 |
| EncodeHaibTfbsT47dGata3sc268V0416102Dm002p1hPkRep2 | 641 |
| EncodeSydhTfbsShsy5yGata2UcdPk | 643 |
| EncodeHaibTfbsH1hescTcf12Pcr1xPkRep1 | 644 |
| EncodeHaibTfbsA549Foxa2V0416102Etoh02PkRep2 | 649 |
| EncodeHaibTfbsHct116Cbx3sc101004V0422111PkRep2 | 651 |
| EncodeHaibTfbsPfsk1NrsfPcr2xPkRep1 | 651 |
| EncodeHaibTfbsA549Ctcfsc5916Pcr1xEtoh02PkRep2 | 652 |
| EncodeHaibTfbsPanc1NrsfV0422111PkRep1 | 652 |
| EncodeHaibTfbsH1hescTead4sc101184V0422111PkRep1 | 653 |
| EncodeHaibTfbsSknshraP300V0416102PkRep1 | 653 |
| EncodeHaibTfbsT47dFoxa1sc6553V0416102Dm002p1hPkRep2 | 653 |
| EncodeHaibTfbsK562CtcfcPcr1xPkRep1V2 | 654 |
| EncodeSydhTfbsK562CtcfbIggrabPk | 656 |
| EncodeSydhTfbsA549Rad21IggrabPk | 660 |
| EncodeHaibTfbsHct116NrsfV0422111PkRep2 | 663 |
| EncodeHaibTfbsSknshraP300V0416102PkRep2 | 665 |
| EncodeHaibTfbsH1hescCtcfsc5916V0416102PkRep2 | 666 |
| EncodeHaibTfbsHct116NrsfV0422111PkRep1 | 666 |
| EncodeSydhTfbsH1hescRad21IggrabPk | 666 |
| EncodeHaibTfbsSknshGata3V0422111PkRep2 | 668 |
| EncodeHaibTfbsMcf7Tead4sc101184V0422111PkRep1 | 670 |
| EncodeUwTfbsAg04449CtcfStdPkRep1 | 670 |
| EncodeSydhTfbsK562MaffIggrabPk | 672 |
| EncodeHaibTfbsHepg2Foxa2sc6554V0416101PkRep2 | 674 |
| EncodeHaibTfbsSknshraRad21V0416102PkRep1 | 676 |
| EncodeSydhTfbsImr90Rad21IggrabPk | 677 |
| EncodeHaibTfbsHepg2Foxa1sc6553V0416101PkRep1 | 678 |
| EncodeUwTfbsGm12870CtcfStdPkRep2 | 681 |
| EncodeSydhTfbsMcf10aesCfosTam14hHvdPk | 687 |
| EncodeHaibTfbsH1hescRxraV0416102PkRep2 | 688 |
| EncodeHaibTfbsK562CtcfcPcr1xPkRep1 | 688 |
| EncodeHaibTfbsK562CtcfcPcr1xPkRep2 | 688 |
| EncodeUwTfbsGm12867CtcfStdPkRep2 | 688 |
| EncodeHaibTfbsHepg2Foxa1sc6553V0416101PkRep2 | 690 |
| EncodeUwTfbsGm12866CtcfStdPkRep2 | 690 |
| EncodeHaibTfbsGm12878Rad21V0416101PkRep1 | 693 |
| EncodeSydhTfbsK562Arid3asc8821IggrabPk | 693 |
| EncodeHaibTfbsH1hescRad21V0416102PkRep2 | 694 |
| EncodeUwTfbsGm12871CtcfStdPkRep2 | 695 |
| EncodeUwTfbsGm12869CtcfStdPkRep2 | 695 |
| EncodeHaibTfbsMcf7CtcfcV0422111PkRep1 | 697 |
| EncodeSydhTfbsK562Smc3ab9263IggrabPk | 698 |
| EncodeUwTfbsHcpeCtcfStdPkRep1 | 699 |
| EncodeHaibTfbsH1hescRxraV0416102PkRep1 | 702 |
| EncodeHaibTfbsA549GrPcr1xDex500pmPkRep2 | 704 |
| EncodeSydhTfbsMcf7Gata3UcdPk | 704 |
| EncodeUwTfbsHepg2CtcfStdPkRep1 | 704 |
| EncodeUwTfbsSknshraCtcfStdPkRep1 | 705 |
| EncodeUwTfbsHbmecCtcfStdPkRep1 | 707 |
| EncodeUwTfbsSknshraCtcfStdPkRep2 | 710 |
| EncodeHaibTfbsSknshraCtcfV0416102PkRep1 | 710 |
| EncodeUwTfbsWi38CtcfStdPkRep2 | 710 |
| EncodeHaibTfbsHct116Rad21V0422111PkRep2 | 711 |
| EncodeSydhTfbsImr90CtcfbIggrabPk | 712 |
| EncodeSydhTfbsGm12878Smc3ab9263IggmusPk | 712 |
| EncodeHaibTfbsA549Rad21V0422111PkRep1 | 713 |
| EncodeHaibTfbsEcc1Rad21V0422111PkRep2 | 713 |
| EncodeUwTfbsGm12875CtcfStdPkRep2 | 714 |
| EncodeUwTfbsWerirb1CtcfStdPkRep1 | 714 |
| EncodeHaibTfbsHl60NrsfV0422111PkRep2 | 715 |
| EncodeHaibTfbsSknshraCtcfV0416102PkRep2 | 715 |
| EncodeUwTfbsHmecCtcfStdPkRep1 | 720 |
| EncodeUwTfbsGm12868CtcfStdPkRep2 | 724 |
| EncodeHaibTfbsT47dFoxa1sc6553V0416102Dm002p1hPkRep1 | 724 |
| EncodeUwTfbsGm12874CtcfStdPkRep1 | 727 |
| EncodeUwTfbsBjCtcfStdPkRep1 | 730 |
| EncodeHaibTfbsK562Rad21V0416102PkRep2 | 730 |
| EncodeUwTfbsNhekCtcfStdPkRep1 | 733 |
| EncodeUwTfbsHl60CtcfStdPkRep1 | 735 |
| EncodeUwTfbsHelas3CtcfStdPkRep2 | 737 |
| EncodeUwTfbsGm12865CtcfStdPkRep1 | 738 |
| EncodeUwTfbsHuvecCtcfStdPkRep1 | 740 |
| EncodeUwTfbsHek293CtcfStdPkRep1 | 741 |
| EncodeUwTfbsGm12878CtcfStdPkRep1 | 742 |
| EncodeUwTfbsGm12866CtcfStdPkRep1 | 742 |
| EncodeUwTfbsGm12868CtcfStdPkRep1 | 742 |
| EncodeUwTfbsGm12872CtcfStdPkRep1 | 745 |
| EncodeUwTfbsGm12865CtcfStdPkRep2 | 747 |
| EncodeUwTfbsSaecCtcfStdPkRep2 | 747 |
| EncodeUwTfbsGm12867CtcfStdPkRep1 | 748 |
| EncodeHaibTfbsMcf7Rad21V0422111PkRep1 | 748 |
| EncodeUwTfbsAg04449CtcfStdPkRep2 | 749 |
| EncodeUwTfbsSaecCtcfStdPkRep1 | 749 |
| EncodeUwTfbsGm12870CtcfStdPkRep1 | 750 |
| EncodeUwTfbsAg09319CtcfStdPkRep1 | 751 |
| EncodeUwTfbsGm12871CtcfStdPkRep1 | 751 |
| EncodeUwTfbsK562CtcfStdPkRep1 | 753 |
| EncodeUwTfbsCaco2CtcfStdPkRep1 | 754 |
| EncodeHaibTfbsHct116Tead4sc101184V0422111PkRep1 | 755 |
| EncodeUwTfbsGm12873CtcfStdPkRep1 | 755 |
| EncodeUwTfbsAg09309CtcfStdPkRep2 | 755 |
| EncodeUwTfbsNhdfneoCtcfStdPkRep1 | 755 |
| EncodeUwTfbsHacCtcfStdPkRep2 | 756 |
| EncodeUwTfbsA549CtcfStdPkRep2 | 758 |
| EncodeHaibTfbsHepg2Ctcfsc5916V0416101PkRep2 | 759 |
| EncodeUwTfbsGm12869CtcfStdPkRep1 | 759 |
| EncodeUwTfbsHcmCtcfStdPkRep2 | 762 |
| EncodeUwTfbsGm12873CtcfStdPkRep3 | 762 |
| EncodeSydhTfbsHepg2Rad21IggrabPk | 763 |
| EncodeUwTfbsHelas3CtcfStdPkRep1 | 763 |
| EncodeUwTfbsGm12864CtcfStdPkRep1 | 764 |
| EncodeHaibTfbsSknshNrsfPcr2xPkRep1 | 767 |
| EncodeUwTfbsAoafCtcfStdPkRep1 | 767 |
| EncodeUwTfbsGm12878CtcfStdPkRep2 | 768 |
| EncodeUwTfbsBjCtcfStdPkRep2 | 768 |
| EncodeUwTfbsGm12875CtcfStdPkRep1 | 769 |
| EncodeHaibTfbsMcf7Rad21V0422111PkRep2 | 772 |
| EncodeHaibTfbsHepg2Ctcfsc5916V0416101PkRep1 | 774 |
| EncodeUwTfbsNhdfneoCtcfStdPkRep2 | 774 |
| EncodeUwTfbsHffCtcfStdPkRep1 | 775 |
| EncodeHaibTfbsMcf7CtcfcV0422111PkRep2 | 775 |
| EncodeUwTfbsAg09309CtcfStdPkRep1 | 776 |
| EncodeUwTfbsHpafCtcfStdPkRep1 | 776 |
| EncodeUwTfbsGm12873CtcfStdPkRep2 | 777 |
| EncodeUwTfbsHaspCtcfStdPkRep2 | 777 |
| EncodeUwTfbsHcfaaCtcfStdPkRep1 | 777 |
| EncodeUwTfbsHacCtcfStdPkRep1 | 777 |
| EncodeUwTfbsHmfCtcfStdPkRep2 | 778 |
| EncodeUwTfbsGm06990CtcfStdPkRep2 | 779 |
| EncodeUwTfbsHuvecCtcfStdPkRep2 | 779 |
| EncodeUwTfbsWi38CtcfStdPkRep1 | 779 |
| EncodeUwTfbsGm06990CtcfStdPkRep1 | 781 |
| EncodeHaibTfbsH1hescPou5f1sc9081V0416102PkRep1 | 782 |
| EncodeHaibTfbsHepg2Rad21V0416101PkRep1 | 782 |
| EncodeUwTfbsGm12864CtcfStdPkRep2 | 783 |
| EncodeHaibTfbsA549Ctcfsc5916Pcr1xDex100nmPkRep1 | 784 |
| EncodeUwTfbsAg04450CtcfStdPkRep1 | 784 |
| EncodeUwTfbsGm12874CtcfStdPkRep2 | 785 |
| EncodeUwTfbsHeeCtcfStdPkRep1 | 787 |
| EncodeUwTfbsAg09319CtcfStdPkRep2 | 787 |
| EncodeUwTfbsAg10803CtcfStdPkRep1 | 788 |
| EncodeUwTfbsGm12872CtcfStdPkRep2 | 788 |
| EncodeUwTfbsHaspCtcfStdPkRep1 | 788 |
| EncodeUwTfbsAg04450CtcfStdPkRep2 | 788 |
| EncodeUwTfbsNhlfCtcfStdPkRep1 | 788 |
| EncodeUwTfbsBe2cCtcfStdPkRep1 | 789 |
| EncodeUwTfbsHreCtcfStdPkRep1 | 790 |
| EncodeHaibTfbsGm12878Rad21V0416101PkRep2 | 791 |
| EncodeSydhTfbsHelas3Rad21IggrabPk | 791 |
| EncodeUwTfbsAoafCtcfStdPkRep2 | 791 |
| EncodeSydhTfbsHepg2Znf274UcdPk | 792 |
| EncodeHaibTfbsEcc1Rad21V0422111PkRep1 | 792 |
| EncodeUwTfbsCaco2CtcfStdPkRep2 | 792 |
| EncodeUwTfbsHvmfCtcfStdPkRep2 | 794 |
| EncodeUwTfbsGm12865CtcfStdPkRep3 | 794 |
| EncodeUwTfbsHpfCtcfStdPkRep1 | 794 |
| EncodeUwTfbsHreCtcfStdPkRep2 | 795 |
| EncodeUwTfbsGm12872CtcfStdPkRep3 | 796 |
| EncodeUwTfbsWerirb1CtcfStdPkRep2 | 798 |
| EncodeUwTfbsRptecCtcfStdPkRep1 | 799 |
| EncodeUwTfbsK562CtcfStdPkRep2 | 800 |
| EncodeHaibTfbsA549Rad21V0422111PkRep2 | 801 |
| EncodeUwTfbsMcf7CtcfStdPkRep1 | 801 |
| EncodeUwTfbsHek293CtcfStdPkRep2 | 803 |
| EncodeHaibTfbsHct116Tead4sc101184V0422111PkRep2 | 804 |
| EncodeUwTfbsHrpeCtcfStdPkRep2 | 804 |
| EncodeHaibTfbsSknshraRad21V0416102PkRep2 | 805 |
| EncodeUwTfbsHmecCtcfStdPkRep2 | 809 |
| EncodeUwTfbsHbmecCtcfStdPkRep2 | 809 |
| EncodeUwTfbsHpafCtcfStdPkRep2 | 811 |
| EncodeHaibTfbsK562Rad21V0416102PkRep1 | 814 |
| EncodeUwTfbsHcpeCtcfStdPkRep2 | 814 |
| EncodeUwTfbsHepg2CtcfStdPkRep2 | 815 |
| EncodeUwTfbsA549CtcfStdPkRep1 | 816 |
| EncodeUwTfbsAg10803CtcfStdPkRep2 | 818 |
| EncodeUwTfbsHvmfCtcfStdPkRep1 | 819 |
| EncodeUwTfbsHct116CtcfStdPkRep1 | 820 |
| EncodeUwTfbsHrpeCtcfStdPkRep1 | 820 |
| EncodeUwTfbsHcmCtcfStdPkRep1 | 821 |
| EncodeUwTfbsBe2cCtcfStdPkRep2 | 822 |
| EncodeUwTfbsHpfCtcfStdPkRep2 | 823 |
| EncodeUwTfbsHffmycCtcfStdPkRep2 | 823 |
| EncodeHaibTfbsHepg2Rad21V0416101PkRep2 | 827 |
| EncodeSydhTfbsHepg2Smc3ab9263IggrabPk | 827 |
| EncodeUwTfbsHffmycCtcfStdPkRep1 | 828 |
| EncodeSydhTfbsShsy5yGata3sc269sc269UcdPk | 834 |
| EncodeUwTfbsHct116CtcfStdPkRep2 | 835 |
| EncodeHaibTfbsH1hescCtcfsc5916V0416102PkRep1 | 837 |
| EncodeUwTfbsRptecCtcfStdPkRep2 | 843 |
| EncodeUwTfbsGm12864CtcfStdPkRep3 | 848 |
| EncodeUwTfbsMcf7CtcfStdPkRep2 | 848 |
| EncodeHaibTfbsPanc1NrsfV0416101PkRep1 | 852 |
| EncodeUwTfbsNb4CtcfStdPkRep1 | 852 |
| EncodeUwTfbsNhekCtcfStdPkRep2 | 857 |
| EncodeUwTfbsHeeCtcfStdPkRep2 | 859 |
| EncodeUwTfbsHmfCtcfStdPkRep1 | 866 |
| EncodeSydhTfbsHelas3Znf274UcdPk | 869 |
| EncodeHaibTfbsH1hescNanogsc33759V0416102PkRep1 | 878 |
| EncodeHaibTfbsH1hescRad21V0416102PkRep1 | 892 |
