# Supplementary material for: The DEK oncoprotein binds to highly and ubiquitously expressed genes with a dual role in their transcriptional regulation
Source: Mol Cancer. 2014 Sep 12;13:215. doi: 10.1186/1476-4598-13-215 (PMC4175287; doi:10.1186/1476-4598-13-215)
Supplement: Supplementary file 6 — Additional file 6: Table S6: DEK affects the expression of genes involved in multiple cellular functions. Gene set enrichment analysis of the changes in gene expression following knockdown of DEK, showing that the genes deregulated upon DEK knockdown are involved in multiple cellular processes that are also enriched for genes shown in Table 1 to be bound by DEK. Positive enrichment scores correspond to enrichment among the genes downregulated by DEK knockdown and negative enrichment scores correspond to enrichment among the genes upregulated by DEK knockdown. FDR denotes the false discovery rate. (DOCX 15 KB) [file 12943_2014_1416_MOESM6_ESM.docx]

**Additional file 6: Table S6. DEK affects the expression of genes involved in multiple cellular functions.** Gene set enrichment analysis of the changes in gene expression following knockdown of DEK, showing that the genes deregulated upon DEK knockdown are involved in multiple cellular processes that are also enriched for genes shown in Table 1 to be bound by DEK. Positive enrichment scores correspond to enrichment among the genes downregulated by DEK knockdown and negative enrichment scores correspond to enrichment among the genes upregulated by DEK knockdown. FDR denotes the false discovery rate.

| GENE ONTOLOGY TERM | ENRICHMENT SCORE | FDR |
| --- | --- | --- |
|  |  |  |
|  |  |  |
| Neutral amino acid transport | -2.95 | 0.000 |
| Heme metabolic process | 2.33 | 0.000 |
| NLS-bearing substrate import into the nucleus | -2.14 | 0.000 |
| Covalent chromatin modification | 1.95 | 0.006 |
| Oligosaccharide metabolic process | 1.93 | 0.006 |
| Response to virus | 1.87 | 0.017 |
| Histone modification | 1.84 | 0.021 |
| Cell polarity | -1.96 | 0.033 |
| Cell cycle S phase | 1.77 | 0.040 |
| Regulation of cell shape | -1.97 | 0.040 |
| Mitochondrial transport | 1.74 | 0.049 |
|  |  |  |
