# Supplementary material for: The DEK oncoprotein binds to highly and ubiquitously expressed genes with a dual role in their transcriptional regulation
Source: Mol Cancer. 2014 Sep 12;13:215. doi: 10.1186/1476-4598-13-215 (PMC4175287; doi:10.1186/1476-4598-13-215)
Supplement: Supplementary file 8 — Additional file 8: Datasets used for the network analysis. (DOCX 34 KB) [file 12943_2014_1416_MOESM8_ESM.docx]

Additional file 8

Supplementary Methods

**Network analysis**

The network analysis was performed on data from samples in the following datasets.

Balgobind, B. V., M. M. Van den Heuvel-Eibrink, R. X. De Menezes, D. Reinhardt, I. H. Hollink, S. T. Arentsen-Peters, E. R. van Wering, G. J. Kaspers, J. Cloos, E. S. de Bont, J. M. Cayuela, A. Baruchel, C. Meyer, R. Marschalek, J. Trka, J. Stary, H. B. Beverloo, R. Pieters, C. M. Zwaan and M. L. den Boer. "Evaluation of Gene Expression Signatures Predictive of Cytogenetic and Molecular Subtypes of Pediatric Acute Myeloid Leukemia." *Haematologica* 96, no. 2 (2011): 221-30.

Haferlach, C., C. Mecucci, S. Schnittger, A. Kohlmann, M. Mancini, A. Cuneo, N. Testoni, G. Rege-Cambrin, A. Santucci, M. Vignetti, P. Fazi, M. P. Martelli, T. Haferlach and B. Falini. "Aml with Mutated Npm1 Carrying a Normal or Aberrant Karyotype Show Overlapping Biologic, Pathologic, Immunophenotypic, and Prognostic Features." *Blood* 114, no. 14 (2009): 3024-32.

Klein, H. U., C. Ruckert, A. Kohlmann, L. Bullinger, C. Thiede, T. Haferlach and M. Dugas. "Quantitative Comparison of Microarray Experiments with Published Leukemia Related Gene Expression Signatures." *BMC Bioinformatics* 10, (2009): 422.

Kohlmann, A., T. J. Kipps, L. Z. Rassenti, J. R. Downing, S. A. Shurtleff, K. I. Mills, A. F. Gilkes, W. K. Hofmann, G. Basso, M. C. Dell'orto, R. Foa, S. Chiaretti, J. De Vos, S. Rauhut, P. R. Papenhausen, J. M. Hernandez, E. Lumbreras, A. E. Yeoh, E. S. Koay, R. Li, W. M. Liu, P. M. Williams, L. Wieczorek and T. Haferlach. "An International Standardization Programme Towards the Application of Gene Expression Profiling in Routine Leukaemia Diagnostics: The Microarray Innovations in Leukemia Study Prephase." *Br J Haematol* 142, no. 5 (2008): 802-7.

Li, L., M. Li, C. Sun, L. Francisco, S. Chakraborty, M. Sabado, T. McDonald, J. Gyorffy, K. Chang, S. Wang, W. Fan, J. Li, L. P. Zhao, J. Radich, S. Forman, S. Bhatia and R. Bhatia. "Altered Hematopoietic Cell Gene Expression Precedes Development of Therapy-Related Myelodysplasia/Acute Myeloid Leukemia and Identifies Patients at Risk." *Cancer Cell* 20, no. 5 (2011): 591-605.

Metzeler, K. H., M. Hummel, C. D. Bloomfield, K. Spiekermann, J. Braess, M. C. Sauerland, A. Heinecke, M. Radmacher, G. Marcucci, S. P. Whitman, K. Maharry, P. Paschka, R. A. Larson, W. E. Berdel, T. Buchner, B. Wormann, U. Mansmann, W. Hiddemann, S. K. Bohlander, C. Buske, Cancer, B. Leukemia Group and A. M. L. Cooperative Group German. "An 86-Probe-Set Gene-Expression Signature Predicts Survival in Cytogenetically Normal Acute Myeloid Leukemia." *Blood* 112, no. 10 (2008): 4193-201.

Miesner, M., C. Haferlach, U. Bacher, T. Weiss, K. Macijewski, A. Kohlmann, H. U. Klein, M. Dugas, W. Kern, S. Schnittger and T. Haferlach. "Multilineage Dysplasia (Mld) in Acute Myeloid Leukemia (Aml) Correlates with Mds-Related Cytogenetic Abnormalities and a Prior History of Mds or Mds/Mpn but Has No Independent Prognostic Relevance: A Comparison of 408 Cases Classified as "Aml Not Otherwise Specified" (Aml-Nos) or "Aml with Myelodysplasia-Related Changes" (Aml-Mrc)." *Blood* 116, no. 15 (2010): 2742-51.

Mills, K. I., A. Kohlmann, P. M. Williams, L. Wieczorek, W. M. Liu, R. Li, W. Wei, D. T. Bowen, H. Loeffler, J. M. Hernandez, W. K. Hofmann and T. Haferlach. "Microarray-Based Classifiers and Prognosis Models Identify Subgroups with Distinct Clinical Outcomes and High Risk of Aml Transformation of Myelodysplastic Syndrome." *Blood* 114, no. 5 (2009): 1063-72.

Payton, J. E., N. R. Grieselhuber, L. W. Chang, M. Murakami, G. K. Geiss, D. C. Link, R. Nagarajan, M. A. Watson and T. J. Ley. "High Throughput Digital Quantification of Mrna Abundance in Primary Human Acute Myeloid Leukemia Samples." *J Clin Invest* 119, no. 6 (2009): 1714-26.

Pigazzi, M., R. Masetti, S. Bresolin, A. Beghin, A. Di Meglio, S. Gelain, L. Trentin, E. Baron, M. Giordan, A. Zangrando, B. Buldini, A. Leszl, M. C. Putti, C. Rizzari, F. Locatelli, A. Pession, G. Te Kronnie and G. Basso. "Mll Partner Genes Drive Distinct Gene Expression Profiles and Genomic Alterations in Pediatric Acute Myeloid Leukemia: An Aieop Study." *Leukemia* 25, no. 3 (2011): 560-3.

Taskesen, E., L. Bullinger, A. Corbacioglu, M. A. Sanders, C. A. Erpelinck, B. J. Wouters, S. C. van der Poel-van de Luytgaarde, F. Damm, J. Krauter, A. Ganser, R. F. Schlenk, B. Lowenberg, R. Delwel, H. Dohner, P. J. Valk and K. Dohner. "Prognostic Impact, Concurrent Genetic Mutations, and Gene Expression Features of Aml with Cebpa Mutations in a Cohort of 1182 Cytogenetically Normal Aml Patients: Further Evidence for Cebpa Double Mutant Aml as a Distinctive Disease Entity." *Blood* 117, no. 8 (2011): 2469-75.

Tomasson, M. H., Z. Xiang, R. Walgren, Y. Zhao, Y. Kasai, T. Miner, R. E. Ries, O. Lubman, D. H. Fremont, M. D. McLellan, J. E. Payton, P. Westervelt, J. F. DiPersio, D. C. Link, M. J. Walter, T. A. Graubert, M. Watson, J. Baty, S. Heath, W. D. Shannon, R. Nagarajan, C. D. Bloomfield, E. R. Mardis, R. K. Wilson and T. J. Ley. "Somatic Mutations and Germline Sequence Variants in the Expressed Tyrosine Kinase Genes of Patients with De Novo Acute Myeloid Leukemia." *Blood* 111, no. 9 (2008): 4797-808.

Verhaak, R. G., B. J. Wouters, C. A. Erpelinck, S. Abbas, H. B. Beverloo, S. Lugthart, B. Lowenberg, R. Delwel and P. J. Valk. "Prediction of Molecular Subtypes in Acute Myeloid Leukemia Based on Gene Expression Profiling." *Haematologica* 94, no. 1 (2009): 131-4.

Wouters, B. J., B. Lowenberg, C. A. Erpelinck-Verschueren, W. L. van Putten, P. J. Valk and R. Delwel. "Double Cebpa Mutations, but Not Single Cebpa Mutations, Define a Subgroup of Acute Myeloid Leukemia with a Distinctive Gene Expression Profile That Is Uniquely Associated with a Favorable Outcome." *Blood* 113, no. 13 (2009): 3088-91.

Yamashita, Y., K. Minoura, T. Taya, S. I. Fujiwara, K. Kurashina, H. Watanabe, Y. L. Choi, M. Soda, H. Hatanaka, M. Enomoto, S. Takada and H. Mano. "Analysis of Chromosome Copy Number in Leukemic Cells by Different Microarray Platforms." *Leukemia* 21, no. 6 (2007): 1333-7.
